# Large-scale evidence for logarithmic effects of word predictability on reading time

**DOI:** 10.1073/pnas.2307876121

**Published:** 2024-02-29

**Authors:** Cory Shain, Clara Meister, Tiago Pimentel, Ryan Cotterell, Roger Levy

**Affiliations:** ^a^Department of Brain & Cognitive Sciences, Massachusetts Institute of Technology, Cambridge, MA 02139; ^b^Department of Computer Science, Institute for Machine Learning, ETH Zürich, Zürich 8092, Schweiz; ^c^Department of Computer Science and Technology, University of Cambridge, Cambridge CB3 0FD, United Kingdom

**Keywords:** language, prediction, reading, nonlinear regression, human language processing

## Abstract

During real-time language comprehension, our minds rapidly decode complex meanings from sequences of words. The difficulty of doing so is known to be related to words’ contextual predictability, but what cognitive processes do these predictability effects reflect? In one view, predictability effects reflect facilitation due to anticipatory processing of words that are predictable from context. This view predicts a linear effect of predictability on processing demand. In another view, predictability effects reflect the costs of probabilistic inference over sentence interpretations. This view predicts either a logarithmic or a superlogarithmic effect of predictability on processing demand, depending on whether it assumes pressures toward a uniform distribution of information over time. The empirical record is currently mixed. Here, we revisit this question at scale: We analyze six reading datasets, estimate next-word probabilities with diverse statistical language models, and model reading times using recent advances in nonlinear regression. Results support a logarithmic effect of word predictability on processing difficulty, which favors probabilistic inference as a key component of human language processing.

## Significance

The difficulty of comprehending words in context is related to their predictability, but what cognitive processes do these predictability effects reflect? In one view, predictability effects reflect facilitation due to anticipatory processing of predictable words. This view predicts a linear effect of predictability on processing demand. In another view, predictability effects reflect the costs of probabilistic inference over sentence interpretations. This view predicts either a logarithmic or a superlogarithmic effect of predictability on processing demand, depending on whether it assumes pressures toward a uniform distribution of information over time. Here, we investigate this question by applying recent advances in nonlinear regression to diverse datasets of human reading. Results support a logarithmic effect of word predictability on processing difficulty.

Comprehending language involves continuously integrating new input with context in order to rapidly form an interpretation of the meanings of the utterances we hear and read. Precisely how the mind achieves this goal is unknown, but a wealth of prior studies offer an important clue: The difficulty of processing a word is related to its predictability in context. This claim is supported by diverse evidence, including self-paced reading (1–3), eye-tracking during reading (4–6), electrophysiology (7–9), and neuroimaging (10–12), using both naturalistic stimuli (4) and stimuli specifically designed to manipulate predictability (3). But what cognitive processes do predictability effects reflect? The answer to this question is tied to a major open debate about the cognitive architecture of human language comprehension (1, 3, 13–15).

Some contend that predictability effects reflect facilitation due to anticipatory processing (e.g., lexical retrieval and structural integration) of future words, e.g., refs. 3 and 16. In this facilitation view, the primary work of sentence processing is to build a mental representation of language structure and meaning, with processing demand proportional to the difficulty of the cognitive operations required to build this representation (e.g., recognizing words, retrieving their representations from the mental lexicon, and integrating those representations into existing syntactic and semantic structures). Prediction facilitates this process by allowing the processor to deal with some of this burden in advance when words are highly predictable from context, thus making more efficient use of processing resources. This view thus predicts a linear effect of contextual probability: A word can be partially processed in advance in proportion to the probability with which it can be correctly guessed in a serial processor (see e.g., refs. 1 and 6 for discussion) or in proportion to the processor resources probabilistically allocated to it in a parallel processor (3). A consequence of the facilitation view is that predictability effects should be driven primarily by highly predictable words, since these are the words for which predictions are likely to be correct and can therefore confer a substantive benefit. Small absolute differences in low probability should have little practical impact on processing demand, since little advance processing is possible. In the limit of total prediction failure (i.e., encountering a word with contextual probability 0), processing simply proceeds without any anticipatory benefit, resulting in no facilitation.

Others contend that predictability effects primarily reflect a processing cost, namely, the cost of probabilistic inference. This cost view draws from information theory in framing prediction as an intrinsic feature of a generative, probabilistic mental processor whose primary work is incremental probabilistic inference over a vast (even infinite) space of possible analyses of the unfolding sentence (17, 18). In this view, an interpretation is a probability distribution, and processing demand is determined by the size of the change in the interpretation: In particular, the Kullback–Leibler (KL) divergence between the interpreter states before and after observing a word. This divergence can be shown to be equivalent to the surprisal (negative log probability, also known as *Shannon information*) of a word in context (18). Thus, this position predicts a logarithmic effect of contextual predictability (or, equivalently, a linear effect of surprisal) on processing difficulty (for discussion of possible mechanisms underlying this predicted logarithmic relationship, see e.g., refs. 1, 19, and 20). A consequence of the cost view is that predictability effects should be driven primarily by small absolute differences in low probability, since these differences are large on a logarithmic (surprisal) scale. In the limit of total prediction failure, catastrophic processing failure (infinite processing cost) ensues—by consequence, under this view, next-word probability is assumed to never be truly zero.

A variant of the cost view is the uniform information density (UID) hypothesis (21, 22), in which probabilistic inference trades off with a bias against word-by-word variation in surprisal (thus smoothing processing load over time). While some versions of the cost view (like surprisal theory e.g., ref. 18), are indifferent to the temporal arrangement of information in the linguistic message, the uid view posits additional pressures toward a more even distribution of information over time, in service of communicative efficiency (23). To the extent that these hypothesized pressures derive from constraints on comprehenders’ information processing, one natural basis for UID pressures would be a superlogarithmic relationship between contextual probability and processing cost: If highly surprising words (i.e., spikes in information content) are disproportionately difficult to process, uniform information density is favored (13). Although early UID proposals did not specify a processing mechanism, recent work has shown that some inferential processing algorithms have superlogarithmic time complexity in predictability, thus potentially grounding UID pressures in comprehension processes (14).

**Fig. 1.**
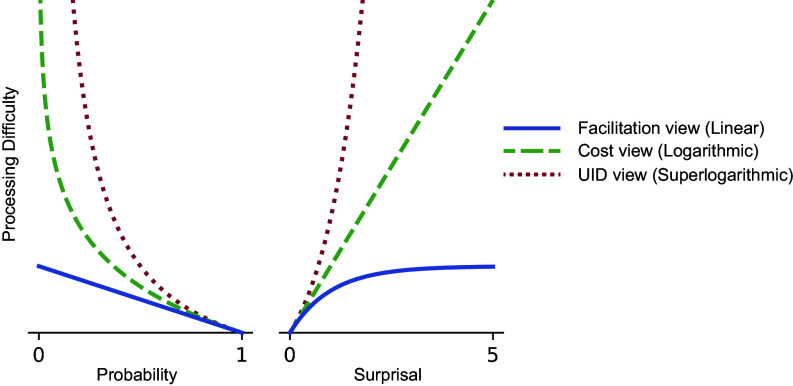
Expected relationships between word predictability (x-axis) and processing demand (y-axis) according to the facilitation, cost, and uid views of predictability effects in language comprehension. Hypothesized effects are represented on both a probability scale (*Left*) and a surprisal scale (*Right*). If, per the facilitation view, prediction serves to facilitate advance processing of highly predictable words, then processing gains will be proportional to probability. By contrast, the cost and uid views derive predictability effects from a process that updates a probability distribution over sentence interpretations, where the cost of this update is logarithmic or superlogarithmic on word predictability. Thus, as shown in the *Left* plot, both the cost and uid views predict rapidly increasing (asymptotically infinite) processing demand as probability goes to 0, and differ in the rate of this predicted increase. Equivalently, as shown in the *Right* plot, the facilitation view predicts a plateau in processing cost as surprisal increases, whereas the cost and uid views respectively predict a linear or superlinear increase in processing cost as a function of surprisal.

The hypothesized relationships between predictability and processing demand under each of these three views are schematized in Fig. 1, which shows all three sets of predictions both on a probability scale (*Left*) and a surprisal scale (*Right*). As shown, the facilitation view (blue) predicts a linear fall-off in processing demand as predictability drops to zero. On a surprisal scale, this prediction appears as a plateau in which the slope of the change in processing demand decreases rapidly on surprisal. By contrast, the cost view (green) predicts a skyrocketing increase in processing demand as predictability drops to zero, since surprisal is climbing to infinity. On a surprisal scale, this prediction appears as a straight line. The uid view predicts an even steeper increase in processing difficulty (red). The uid view is most easily differentiated from the cost view on a surprisal scale, where, as shown, the slope of the change in processing demand also increases on surprisal.

The facilitation, cost, and uid views thus make testably different predictions about the relationship between word predictability and processing demand. However, the empirical record on this question is currently mixed, with some studies reporting a linear predictability effect (3), others reporting a logarithmic predictability effect (1, 15, 24, 25), and still others reporting a superlogarithmic predictability effect (13, 14). These differences in results plausibly derive from methodological differences, some of which concern experimental design. For example, a key challenge in studying the construct of human subjective predictability is that it is not observable and must be approximated using a model of contextual probability, and studies differ in how they implement this approximation. For example, Smith and Levy (1) quantified contextual word probabilities using statistical language models, whereas Brothers and Kuperberg (3) used probabilities derived from a cloze task (26) in which humans predicted the next word based on preceding context. The advantages of cloze estimates are that i) they directly reflect human subjective probabilities and ii) they have been shown to be superior to corpus-based estimates in predicting human reading patterns (27); although both of these purported advantages are under debate (*Discussion* and *SI Appendix*, 1). The disadvantage of cloze estimates is the inherent practical difficulty in accurately estimating degrees of low contextual probability—millions of samples per context would be needed to reach the precision of statistical language models. Unfortunately, these are precisely the probabilities that most strongly differentiate the empirical predictions of the hypotheses reviewed above.

Studies also show design differences in their use of constructed vs. naturalistic language materials. Brothers and Kuperberg used constructed materials, which they justify in light of the problems for causal inference presented by observational (naturalistic) data. However, these inferential gains come at the cost of i) limited coverage of the critical low-probability interval of the contextual probability spectrum, ii) data loss due to restricted focus on a critical region, rather than word-by-word modeling, and iii) ecological validity (see also, e.g., refs. 28, 29, 30, 31, *SI Appendix*, 1). In addition, the theoretically predicted patterns should at minimum hold in observational data, even if the existence of such patterns is insufficient to establish causal effects. Perhaps in light of these considerations, most other studies of the functional form of predictability effects use naturalistic data, e.g., refs. 1, 13, 14, 15, and 25.

Design differences aside, all previous studies share a reliance on standard analysis methods that enforce implausible simplifying assumptions when applied to complex continuous-time processes like language comprehension. These assumptions include linearity and/or additivity of effects, discrete-time dynamics (i.e., spillover effects at the word level), time-invariance, and constant error. All of these assumptions are likely unwarranted for human language comprehension, and a failure to account for their violations can substantially influence effect estimates and hypothesis tests, especially in naturalistic data (32, 33). Although some studies (1, 25), relax the linearity assumption through generalized additive models GAMs (34), which can flexibly infer nonlinear effects, they still rely on implausible dynamical and distributional assumptions (i.e., a homoscedastic, additive, discrete-time stationary model).

In light of these concerns, we revisit the functional form of word predictability effects by analyzing the largest collection of naturalistic reading data to date (six large-scale public English-language datasets with a combined total of over 2.2 million data points across three different reading modalities), combining recent advances in statistical language modeling with statistical analyses based on the recently introduced continuous-time deconvolutional regressive neural network (CDRNN, refs. 32 and 33). In brief, CDRNNs leverage the power of deep learning to infer a highly expressive impulse response function (IRF) that relates features of fixated words to measured reading times as a function of their distance in continuous time. For example, the fitted model will contain an estimate of how a given surprisal value at a given fixated word will affect reading behavior 500 ms in the future, thus directly taking into account the possibility of nonlinear and continuously delayed effects. The architecture of CDRNNs allows them to relax all of the aforementioned simplifying assumptions: Predictors can exert arbitrary nonlinear and interactive influences on the response, the response function can change over the course of the experiment (nonstationarity), and the predictors can influence all parameters of the predictive distribution, not just the mean (heteroscedasticity). CDRNNs thus provide a more flexible analysis approach that substantially improves fit to reading behavior (32, 33).

To anticipate our results, even though CDRNNs are expressive enough to learn any of the functional forms discussed above, they emergently discover a logarithmic effect of word predictability, as predicted by the cost view (17, 18). Detailed model comparisons show that this logarithmic effect is better supported by our results than either the linear effect predicted by the facilitation view or the superlogarithmic effect predicted by the uid view.

## Results

We evaluate predictability effects in six publicly available naturalistic reading datasets: The Brown self-paced reading (SPR) dataset (1), the Dundee eye-tracking (ET) dataset (35), the monolingual English version of the GECO eye-tracking dataset (36), the Natural Stories self-paced reading dataset (37), the Natural Stories Maze dataset (38), and the Provo eye-tracking dataset (39). In each case, the critical response variable is how long participants spent reading each word in a running text (for supplemental analyses of predictability effects on word skipping in the three eye-tracking datasets, *SI Appendix*, 2).

We consider word predictability estimates derived from diverse statistical language models, computational models that define a probability distribution over the next word given its linguistic context. Specifically, we consider an *n*-gram model that predicts the next word from a table of counts of word sequences in a text corpus (40), a probabilistic context-free grammar (PCFG) model that predicts the next word given a set of hypotheses about the sentence’s structure (syntactic tree, (41)), and three pre-trained deep neural network language models based on the transformer architecture (42): GPT-2(-small) (43), GPT-J (44), and GPT-3 (45).

We analyze these data using continuous-time deconvolutional regressive neural networks (32, 33), controlling for numerous perceptual, motor, and linguistic variables as well as participant and item effects in a mixed model design (CDRNNs recover expected effects of our word length and frequency controls; see *SI Appendix*, 3). To shed light on the functional form of word predictability effects, we consider not only models that can find an unconstrained function of word surprisal (f(surp)) but also models that are constrained to be linear in either probability (prob) or some fixed power of surprisal (surp^1/2^, surp^3/4^, surp^1^, surp^4/3^, or surp^2^).

As in prior work (1, 14, 25), part of our analysis rests on visualization of the model-estimated relationship between predictability and processing cost. However, we go beyond these visual impressions and compare model performance on a held-out portion of each dataset under different assumptions about the nature of predictability effects. All statistical comparisons are based on pre-trained CDRNNs’ performance on data not seen in training, directly grounding hypothesis tests in how well models generalize.

For further details about the experimental tasks and materials, datasets, language models, regression analyses, and statistical testing protocols, see *Materials and Methods*. For simplicity, unless otherwise specified, we report comparisons that aggregate across all datasets considered in this study. Complete results tables for all statistical tests conducted in this study (including results on individual datasets) are given in *SI Appendix*, 4.

### What Is the Estimated Shape of Predictability Effects?.

**Fig. 2.**
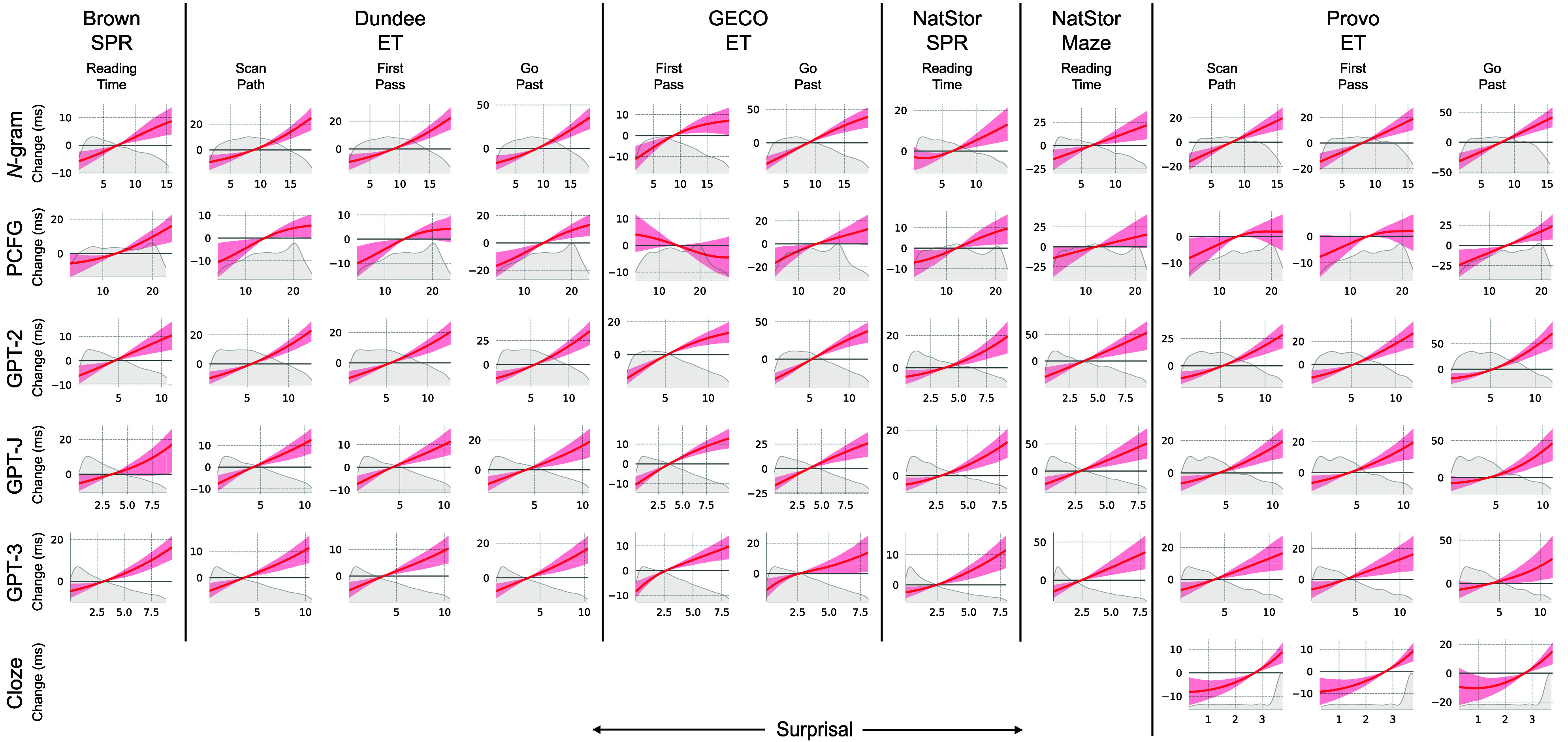
CDRNN-estimated functional form of surprisal (x-axis) effects on reading times (y-axis) across language model types (*n*-gram, PCFG, GPT-2, GPT-J, GPT-3, and human cloze) with no delay (i.e. at the surprising word). Plots cover the interdecile range of values in each training dataset (for plots covering the full empirical range, see *SI Appendix*, Fig. S9). Kernel density plots show the distribution of surprisal values in the training data over the plotted range.

We first establish qualitative impressions about the functional form of predictability effects by visualizing the estimates from the unconstrained f(surp) CDRNN models. Estimates for the effect of word surprisal on fixations to that word (i.e., at no delay) are plotted across language models and datasets in Fig. 2 (for visualization of these effects over time following stimulus onset, see *SI Appendix*, 5). With one exception (PCFG surprisal effects on GECO first pass reading times), all estimates show the expected positive relationship between surprisal and reading time (in fact, PCFG surprisal in GECO first pass reading times also shows a positive surprisal effect, albeit at longer latencies; see *SI Appendix*, 5 for visualizations and *SI Appendix*, 6 for additional discussion). Furthermore, estimates are primarily consistent with a logarithmic predictability (linear surprisal) effect. They are inconsistent with a linear predictability effect, according to which processing cost should essentially not vary beyond about four nats of surprisal (around 2% predictability). Although there are hints of superlogarithmicity (superlinear surprisal effects) in some configurations (e.g., *n*-gram effects on Dundee scan path durations) and of sublogarithmicity (sublinear surprisal effects) in others (e.g., GPT-2 effects on GECO first pass durations), the uncertainty interval covers a logarithmic effect in nearly all cases. In *SI Appendix*, 7, we also show that CDRNN models tend to recover a logarithmic predictability effect when provided with predictability measures on a linear or superlogarithmic scale. This outcome is at odds with some recent reports of superlogarithmic effects in a subset of these data, e.g., refs. 13 and 14. They are likewise at odds with recent claims that better language models find more strongly superlogarithmic effects (14)—in our results, estimates using a much larger model (GPT-3) are not systematically more superlogarithmic than estimates using smaller models with worse perplexity like GPT-2 (*SI Appendix*, 8). We return to these divergences from prior work in *Discussion*.

### Are Predictability Effects Robust in Naturalistic Reading?.

**Fig. 3.**
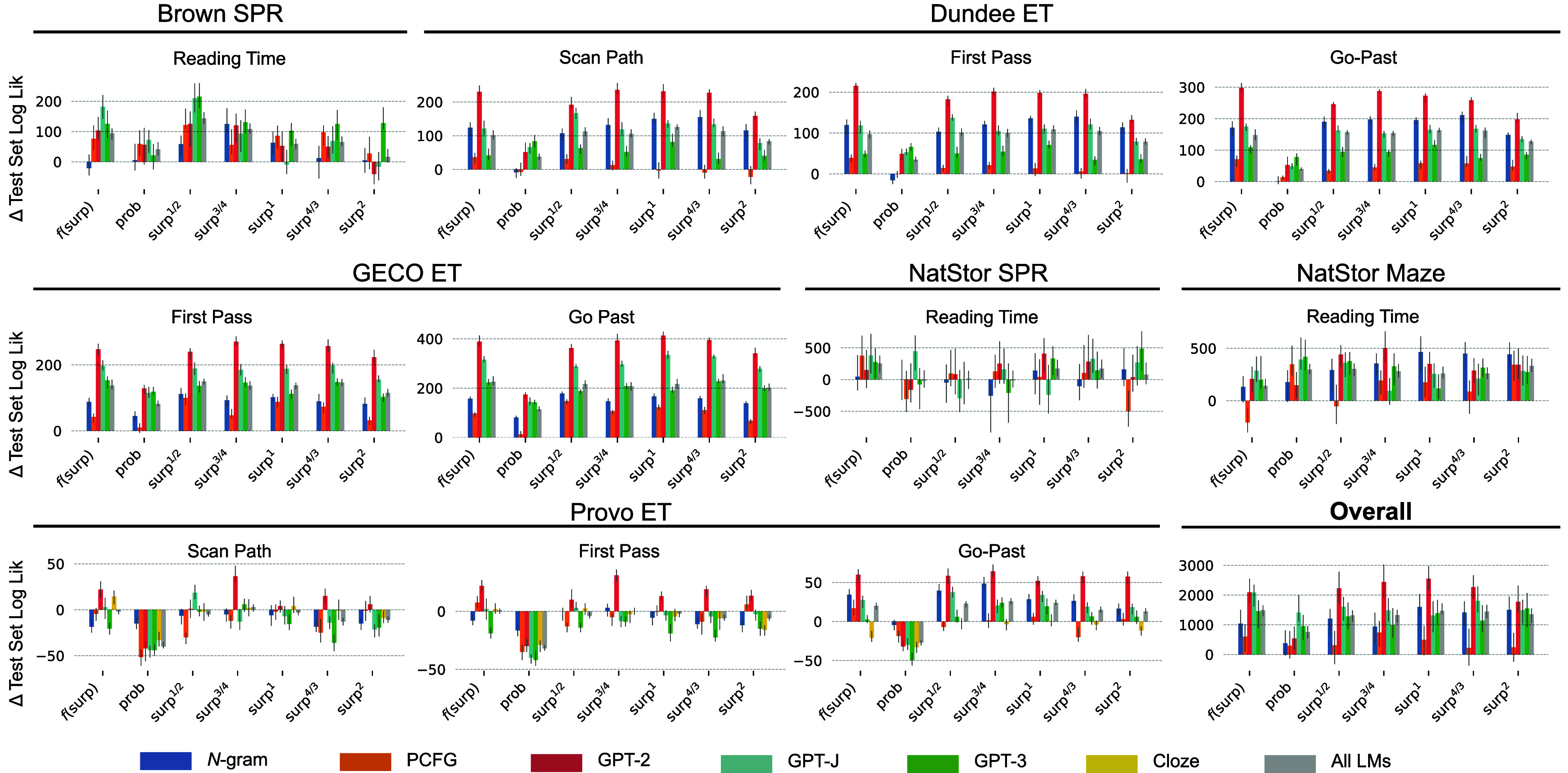
Change in test set log likelihood as a function of i) language model and ii) predictability-cost function, relative to a baseline model containing no predictability measure. Predictability-cost functions include the main CDRNN model that enforces no constraints on functional form (f(surp)), along with models assuming a linear effect of word probability (prob) and models assuming a linear effect on some exponent of surprisal (from surp^1/2^ to surp^2^). Bars represent the median pairwise likelihood difference between the models of the critical and baseline ensembles (10 models each, resulting in 100 likelihood differences per bar). Error bars show 95% bootstrapped CIs of the median pairwise likelihood difference.

We now confirm that our analyses replicate numerous prior findings of predictability effects in reading, e.g., refs. 1, 4, 25, and 46 inter alia. To this end, patterns of fit of pre-trained CDRNN models to unseen data are visualized in Fig. 3, which shows the median change in out-of-sample test-set likelihood relative to a baseline containing no predictability variable. The primary models of interest—f(surp)—use unconstrained (possibly nonlinear) functions of surprisal. The f(surp) model for each statistical language model is significant over a baseline model with no predictability effect, as is the f(surp) model for all language models in aggregate, supporting a generalizable effect of word predictability. Moreover, the more constrained models prob, surp^1/2^, surp^3/4^, surp^1^, surp^4/3^, and surp^2^ are also significant over the baseline, indicating that this finding does not critically depend on assumptions about functional form. We thus find strong evidence that reading behavior is modulated by predictability in context, consistent with much prior work.

### Which Language Model Best Estimates Human Subjective Surprisal?.

We next evaluate differences in psychometric quality (predictive fit to reading times) across language models. The numerically best performing language model overall is GPT-2(-small), which significantly outperforms all other language models in the f(surp) configuration except GPT-J (Fig. 3), and which shows especially pronounced performance gains over other models in the more constrained configurations surp^3/4^ and surp^1^. The finding that GPT-2-small substantially outperforms GPT-3 is striking given that GPT-3 has over 1,000 times more parameters than GPT-2-small, is trained on much more data, and has better overall perplexity (see the surprisal density plots in Fig. 2; perplexities by language model and dataset are provided in *SI Appendix*, 9). This result suggests that previously reported correlations between the linguistic and psychometric performance of language models (25, 47) may not hold for more recent large transformer language models, and instead suggests limitations on the benefits of language model perplexity for modeling human subjective word probabilities (48). Given these performance differences, although we consider results across language models in the remainder of this article, we place special emphasis on results derived from GPT-2, since these most reliably characterize reading behavior overall.

### Main Question: Is Processing Difficulty Linear, Logarithmic, or Superlogarithmic on Word Predictability?.

We now turn to the statistical analyses that bear on our core question, using out-of-sample model performance to assess hypothesized functional forms of predictability effects. As shown in Fig. 3, we compare the performance of the unconstrained f(surp) models to that of models constrained to respect some fixed predictability-cost function, namely models that are linear on raw probability (prob, as predicted by the facilitation view) and on powers of surprisal (surp^1/2^, surp^3/4^, surp^1^, surp^4/3^, and surp^2^), where the surp^1^ model instantiates the logarithmic pattern predicted by the cost view and the surp^4/3^ and surp^2^ models instantiate superlogarithmic patterns consistent with the uid view. The surp^1/2^ and surp^3/4^ models instantiate sublogarithmic effects and are included for completeness, even though no existing theory predicts these functional forms.

**Fig. 4.**
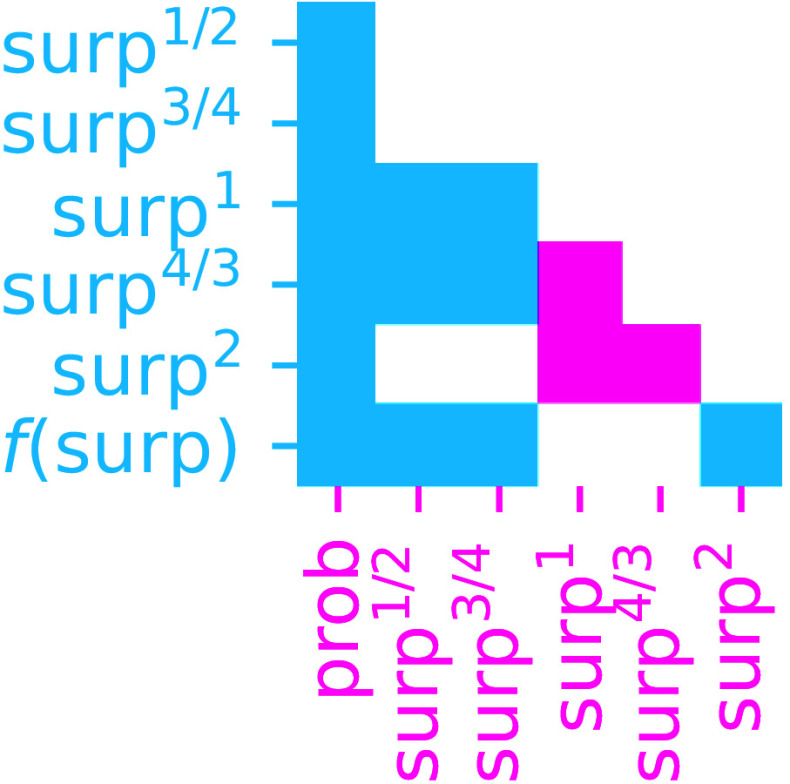
Results of statistical comparisons across all datasets and language models between pairs of assumed forms for the effect of word predictability. For a given pair, cyan indicates that the model on the row has significantly better test set performance than the model on the column, magenta indicates that the model on the column significantly outperforms the model on the row, and white indicates no significant difference. Only the lower triangle is shown. Tests use false discovery rate correction for multiple comparisons (49) across all tests. See *SI Appendix*, Fig. S4 for results by dataset and language model.

Overall results across language models and datasets (Fig. 4; see *SI Appendix*, 4 for full testing results by model and dataset) indicate i) that prob significantly under-performs all surprisal-based models, ii) that surp^1^ is the best performing constrained model overall, significantly outperforming both sublogarithmic models (surp^1/2^ and surp^3/4^) and superlogarithmic models (surp^4/3^ and surp^2^), and iii) that there is no significant advantage of unconstrained f(surp) models over surp^1^ models constrained to have a logarithmic predictability effect. There is thus no systematic evidence in our study that predictability effects are anything other than logarithmic, and, of the constrained models, the logarithmic effect fits the data better than either the linear effect predicted by the cost view or the superlogarithmic effect predicted by the uid view. Results from this large-scale investigation therefore favor a logarithmic predictability effect.

One logical possibility is that both kinds of processes (anticipatory facilitation and inferential cost) act simultaneously, giving rise to a superposition of linear and logarithmic effects on predictability. This view has been advocated by a prior study of predictability effects on event-related potentials in electrophysiology experiments (50), which supported additive linear and logarithmic predictability effects. This position predicts a sharper fall-off in processing demand in the low-surprisal interval due to additional linear facilitation at highly predictable words. Might a similar pattern hold in reading data? Visual estimates in Fig. 2 do not appear to support this hypothesis. To address this hypothesis directly, we focus on GPT-2 (the language model with the strongest overall psychometric performance) and fit models that contain strictly linear predictors for one or both of GPT-2 probability and GPT-2 surprisal. We then compare the generalization performance of the model containing both GPT-2 probability and GPT-2 surprisal to that of the models containing only one or the other (“Prob vs. Surp” of *SI Appendix*, Tables S5–S8). We only find significant contributions of GPT-2 probability (linear effect) above and beyond GPT-2 surprisal (logarithmic effect) in two datasets (Natural Stories SPR and Natural Stories Maze). However, in the largest of these (Natural Stories SPR), the GPT-2 probability effect does not go in the predicted direction: More probable words are associated with longer reading times (*SI Appendix*, Fig. S7). Thus, although the overall contribution of GPT-2 probability over GPT-2 surprisal alone across datasets is significant (*SI Appendix*, Table S9), this significance is driven largely by the opposite pattern from that predicted by the facilitation view. We therefore do not find evidence to support the additive linear and logarithmic effects of word predictability reported by ref. 50, a difference that could be due to modality differences (reading in our study vs. electrophysiology in theirs). Instead, overall results are primarily consistent with logarithmic predictability effects alone.

Nonetheless, there are potentially important differences in testing outcomes between individual datasets and language models, as visualized in *SI Appendix*, Fig. S4. For example, superlogarithmic models tend to show stronger performance in the Natural Stories SPR dataset: Aggregating across language models, surp^4/3^ outperforms surp^1^. In addition, across all datasets, the larger transformer language models (GPT-J and GPT-3) favor a superlogarithmic model over a logarithmic one: surp^4/3^ outperforms surp^1^ using GPT-J predictability estimates, and surp^2^ outperforms surp^1^ using GPT-3 predictability estimates. Both of the outcomes above (superlogarithmic effects in Natural Stories SPR and a bias toward superlogarithmicity in larger language models) are consistent with recent findings from ref. 14. However, they should be interpreted with caution, since i) we find no evidence that these dataset-specific superlogarithmicities are characteristic of reading in general (across our entire sample; see *SI Appendix*, 8 for in-depth discussion of this point), and ii) the GPT-J and GPT-3 language models perform worse in overall psychometric comparisons than GPT-2 (especially in the critical surp^1^ condition; Fig. 3), which does not exhibit a bias toward superlogarithmicity. The GPT-J and GPT-3 patterns are therefore a questionable basis for claims about predictability effects in humans, in the absence of similar patterns in better-performing GPT-2 models. In addition, GPT-3 in particular was trained on a large web corpus that is not publicly available. Given that all the reading stimuli in these experiments are available online, it is plausible that GPT-3 was trained on some or all of these texts, which could artificially reduce its surprisal estimates for them, especially for highly surprising words that contribute large training gradients. This could give rise to artifactual superlogarithmicities when using GPT-3 surprisal estimates to predict human reading times, since compression in the high-surprisal regime will lead to steeper increases in processing cost if the underlying cost function is logarithmic on human subjective probabilities. Therefore, although these exceptions are noteworthy and warrant future research, the overall pattern emerging from our study is most favorable to logarithmic predictability effects.

### Do Results Change under Cloze Estimates of Word Predictability?.

The results reported here derive from statistical language models that perform next-word prediction. However, the current experimental gold standard measure of word predictability is the cloze task (26) in which predicted next-word continuations given a context are collected from human participants, e.g., refs. 3, 6, 27, 46, and 51, 52, 53. Because cloze estimates are human-derived, they avoid potential confounds due to mismatch between statistically estimated and human subjective next-word probabilities. Indeed, the use of statistical rather than cloze predictability estimates has been cited as a criticism of prior work on the functional form of word predictability effects [ref. 3; see *SI Appendix*, 1 for extended discussion]. However, some have argued that the cloze task may measure different cognitive processes than those that underlie real-time language comprehension (27, 52), and there is currently debate as to whether cloze estimates perform better (27, 51, 53) or worse (54, 55) than statistical language models as estimators of human processing difficulty (*SI Appendix*, 1).

Thoroughly investigating this question in our current experimental setup is prohibitive, since it would require word-by-word cloze distributions for all the large naturalistic texts in this study (including an entire novel in the case of the GECO dataset). However, the Provo dataset was fully cloze-normed as part of its design (6). We therefore use Provo to address two questions: i) Do results depend critically on the use of statistical predictability estimates, and ii) how does our best statistical language model (GPT-2) perform relative to cloze?

Regarding (i), estimates using cloze surprisal for the Provo dataset are plotted in Fig. 2. As shown, estimates are if anything more strongly superlogarithmic than estimates using any of the statistical language models, and the f(surp) model significantly outperforms the prob model for all duration types. Despite the visually superlogarithmic estimates in Fig. 2, the performance profile for cloze is similar to that of other predictability estimates (Fig. 3), with peak performance from logarithmic (surp^1^) or slightly sublogarithmic (surp^3/4^) models, and worse performance from either superlogarithmic model (surp^4/3^ and surp^2^). Thus, results under cloze remain most consistent with logarithmic predictability effects.

Regarding (ii), GPT-2 surprisal numerically outperforms cloze surprisal in all comparisons, significantly so for first pass and go-past durations (Fig. 3). This outcome suggests that at the scales of training corpus, language model architecture, and cloze norm dataset size investigated here, the benefits of artificial model-based surprisal estimation (e.g., differentiating among degrees of low probability, or capturing variability that may be under-represented by cloze distributions, see *SI Appendix*, 1) may now outweigh whatever disadvantages model-based estimates might have in principle relative to cloze norms, at least for naturalistic-text datasets (54, 55).

## Discussion

In this study, we revisited a longstanding question about predictive processing during language comprehension, namely, what is the functional form of predictability effects on measures of incremental comprehension difficulty? We evaluated five statistical language models (*n*-gram, PCFG, GPT-2, GPT-J, and GPT-3 models) on six large-scale reading datasets using recent advances in nonlinear regression modeling for naturalistic language processing data (CDRNNs, (32, 33)). Unlike most prior work on this question (cf., 13), our statistical tests are based exclusively on out-of-sample model fit, thus grounding the outcomes of tests in the generalizability of effects.

Results favor a logarithmic effect of word predictability (linear effect of word surprisal, 1) compared to a linear (3) or superlogarithmic (13, 14) effect. Nonlinear CDRNN models of human reading emergently discover estimates consistent with a logarithmic predictability effect, improve upon models constrained to have a linear or superlogarithmic predictability effect, and generally do not improve upon models constrained to have a logarithmic predictability effect. Similarly, models constrained to have a logarithmic effect generally outperform models constrained to have a linear effect, as well as models constrained to have slightly sublogarithmic or superlogarithmic effects. Supplementary analyses (*SI Appendix*, 7) suggest that this logarithmic effect of word predictability is not due to an inductive bias of the CDRNN model. Moreover, when we reanalyze data from Brothers and Kuperberg (3)—the strongest current counterevidence supporting linear rather than logarithmic predictability effects—we find (*SI Appendix*, 1) that GPT-2 predictability estimates instead favor a logarithmic over a linear effect and fit Brothers and Kuperberg’s self-paced reading data as well as the cloze estimates used in the original study.

Our findings have implications for current understanding of the cognitive processes that give rise to predictability effects, favoring the view that predictability effects primarily reflect the cost of probabilistic inference (17) over the view that predictability effects primarily reflect anticipatory facilitation (3). Furthermore, our results do not support the hypothesis that processing demand is superlogarithmic in predictability, which might give rise to uniform information density pressures (13, 14, 22).

In making this claim, we stress that we have used the term facilitation more narrowly than it is sometimes used in the field: By “facilitation view,” we are referring specifically to theories of a linear form for the predictability–cost relationship whereby predictability effects are driven primarily by highly predictable words, rather than the more general idea that contextually preactivated words are read more quickly. Our findings agree with the construal of predictable words as “facilitated” in this more general sense: When surprisal is low, the cost view predicts fast reading (because the inferential update is small).

### Implications for Theories of Language Comprehension.

A common stance among psycholinguists is that prediction serves a largely facilitatory (3)—and possibly optional (56)—role in a comprehension process dominated by the demands of incrementally assembling an ever-richer representation of sentence structure and meaning. These hypothesized demands include lexical retrieval (57) and syntactic integration (58), and successful prediction might allow the processor to discharge these demands early and thus use computational resources more efficiently. As word predictability nears zero, the processor gets little of this anticipatory benefit and converges to a wait-and-see mode. This view predicts little difference for processing between next-word probabilities of e.g., P=0.001 vs. P=0.0001: In both cases, the full processing burden will fall on the word itself. Our results challenge this facilitation view, instead showing large changes in processing cost due to small absolute differences within the low probability regime.

Rather, our results support an information-theoretic view (17, 18) in which a major driver of processing cost is probabilistic inference over a (possibly vast) space of interpretations of the unfolding sentence (possibly including syntactic parses, predicate logic, and any other cognitively-relevant form of sentence representation). Under this view, an interpretation is a probability distribution over this representation space, and words with small absolute differences in probability can have large differences in the size of the update they require to the interpretation distribution, due to the logarithmic form of the KL divergence between the interpreter states before and after observing a word. Our results bear out this prediction by supporting a linear increase in reading latencies as a function of this logarithmic divergence (surprisal), thereby supporting the cost view that prediction is not merely an aide to comprehension, but an inherent consequence of what it means to comprehend.

The importance of probabilistic inference draws support from computational parsing algorithms, the design of which is dominated by the problem of finding (rather than assembling) the correct analysis of a sentence e.g., refs. 59–67. Computationally implemented approaches thus suggest that the problem of local ambiguity in sentence interpretation goes well beyond the garden-path constructions and attachment ambiguities that have largely preoccupied psycholinguistic treatments of this problem (68, 69, 70, 71, 72, 73, 74, 75, 76, 77, 78), and may instead be the primary obstacle to successful comprehension (79, 80, 81). It is therefore not surprising to find evidence that probabilistic inference may also be a major preoccupation of the human language comprehension system.

That said, two points of clarification must be emphasized. First, our claims are not at odds with the notion of preactivation per se, but only with a facilitatory construal of its influence on processing cost. Diverse experimental evidence supports the hypothesis that predictable linguistic units are represented in the mind and brain before they are encountered (82–87). Probabilistic inference is perfectly compatible with this evidence, since the candidate interpretations among which the processor allocates probability mass might contain information about as-yet unobserved material. Our study simply constrains the hypothesis space around how these representations influence incremental processing demand.

Second, our claims are compatible with the existence of other, surprisal-independent determinants of incremental processing demand. In other words, our claims do not entail commitment to a strong view of surprisal as the sole causal bottleneck between representations and processing demand (c.f., 18). Experiments have identified diverse surprisal-independent influences on processing demand, including lexical (88, 89) and repetition (90) priming, word frequency (91, 92), dependency locality (93, 94), and garden path constructions (95). Whether all such influences can be reconciled with surprisal theory is currently unclear (for recent attempts to address some of them, see refs. 96 and 97). But the results of our study are orthogonal to this issue: We are not claiming that surprisal is the only determinant of processing difficulty, only that it is an important one, and that predictability effects in natural reading cannot be reduced to mere facilitation at highly predictable words. As a result, we argue that mechanisms of probabilistic inference should feature prominently in theories of language comprehension, regardless of any other constraints on constructing sentence representations in memory.

One potential challenge for the cost view that we have advocated is a well-replicated finding that invalid parafoveal preview (i.e., replacing words near the current fixation with other words or random characters) eliminates predictability effects in early eye movement measures [first fixation duration and first pass duration, e.g., refs. 98–100. This finding has been taken to indicate that, at least in early measures, predictability primarily affects only the earliest stages of lexical processing, when visual cues to word identity are poorly resolved in the parafovea and must be supplemented by top–down predictive signals (100). This interpretation is hard to reconcile with our construal of predictability effects as primarily reflecting high-level structural and semantic inference. Although we cannot address this concern empirically since all of our data used valid preview, we offer three comments. First, three of our six datasets used self-paced designs that have no preview (but still show strong predictability effects), and the same experimental studies above found that predictability effects were preserved under invalid preview in later measures go-past durations and N400 amplitudes (99, 100). Thus, predictability effects register consistently in later measures that plausibly reflect high-level inferential processing. Second, our finding of predictability effects (under valid preview) in early eye movement measures like scan path and first past durations may reflect inferential processing that began during parafoveal preview and continues after fixation (some models of surprisal effects, e.g., Smith and Levy (1), assume that inferences are continuously updated, rather than than being strictly post-lexical, which is consistent with inference during preview). Invalid preview would delay the start of such processing, potentially pushing predictability effects outside the time window within which they would normally be captured by earlier measures (but preserving them in later ones). Third, one interpretational challenge for studies that manipulate preview validity is that the parafovea provides incorrect information about the future realization of the text. Although participants are usually not conscious of the preview validity manipulation, invalid preview could still send signals to the language processing system that predictions are incorrect with unusual frequency (when in fact they are not). This could result in a strategic adaptation in which the processing system relies less on prediction (or, put information-theoretically, generates more entropic predictions), thereby attenuating predictability effects. The high-cloze (i.e., very predictable) items typically used in these experimental studies may be especially susceptible to such an attenuation, since they encourage strong predictions that are temporarily disconfirmed parafoveally. Current evidence about preview validity may therefore be compatible with the view of predictability effects we have advocated, although the discussion above offers many opportunities for follow-up study.

Our results also discriminate between extant information-theoretic models of language comprehension by favoring the logarithmic effect of word predictability predicted by standard surprisal theory (17, 18) over the superlogarithmic effect that has been hypothesized to give rise to pressures toward uniform information density (13, 14); although there are visually apparent superlogarithmicities in some model estimates (Fig. 2 and *SI Appendix*, Fig. S8), superlogarithmic models generally underperform logarithmic or sublogarithmic ones (Fig. 3). Our results nonetheless highlight the challenge of discriminating between fine differences in hypothesized functional form on the basis of reading data, even at scale. Despite some statistically significant advantages of a logarithmic effect shape, we tend to find a broad range of near-equivalence in model performance across the sublogarithmic–superlogarithmic spectrum, with variation across language models and datasets as to the precise peak of this continuum (Fig. 3). Since UID does not make precise claims about how strong superlogarithmicity should be (and is thus consistent with an arbitrarily diminishing exponent on log probability), it may not be possible to rule out UID pressures on the basis of this kind of data. Our evidence is simply more consistent with a logarithmic than a superlogarithmic effect of word predictability on reading times, while placing some constraints on the strength of superlogarithmicity (e.g., squared surprisal is likely too strongly superlogarithmic).

Why do our results differ from those reported in other recent studies using partially overlapping data (13, 14)? With respect to Meister et al. (13), the strongest evidence for superlogarithmicity came from offline acceptability judgments; the evidence from online reading measures was more equivocal. The relationship between online and offline measures of comprehension difficulty is currently poorly understood, and we leave this discrepancy to future investigation. With respect to Hoover et al. (14), their claims of superlogarithmicity are based on visual estimates (and descriptive statistics derived from those estimates) from models fitted only to the Natural Stories SPR dataset. Our results in fact partially replicate theirs, since estimates tend to be visually superlogarithmic in Natural Stories SPR (especially over the long right tail of surprisal values, see *SI Appendix*, Fig. S9), and a slightly superlogarithmic model (surp^4/3^) outperforms a logarithmic one on that dataset, aggregating over all language models. However, this outcome appears to be largely restricted to Natural Stories SPR and does not generalize to a broader sample of reading data. Furthermore, a recent study of predictability effects across languages (15) obtained strongly logarithmic estimates (with little hint of superlogarithmicity) in ten non-English languages. In the absence of reasons to think that Natural Stories SPR is an especially reliable source of evidence on this question (see *SI Appendix*, 8 for counterarguments), our results suggest that the pattern reported by Hoover et al. may not be characteristic of reading in general.

### Implications for Statistical Modeling of Human Subjective Word Probabilities.

Our results additionally differentiate computational models of human next-word prediction. Surprisal estimates from GPT-2(-small) (43) substantially outperform surprisal estimates from *n*-gram, PCFG, GPT-J, and GPT-3 models. GPT-2 therefore appears to reside in a “Goldilocks” region of psychometric performance between language models that are too constrained on the one hand (*n*-gram and PCFG models) and too powerful on the other (GPT-J and GPT-3). This outcome challenges the notion that previously reported correlations between the linguistic and psychometric performance of language models (25, 47, 101) will extrapolate to models of ever-increasing size, complexity, and quantity of training data (48). Instead, the task of using language model predictions to estimate human reading times may be akin to tasks in natural language processing that show an “inverse scaling” property, whereby task performance is inversely related to model size (102–104). This result has both methodological and scientific implications. From a methodological standpoint, bigger is not always better; the selection of a language model for psycholinguistic research may need to consider additional dimensions (beyond perplexity). From a scientific standpoint, homing in on classes of models that best mimic human processing patterns offers the opportunity for new insights into the learning and processing mechanisms that underlie human language abilities (9, 105), a direction that we leave to future work.

In addition, our results also bear on the widespread perception of cloze norms as the gold standard method for estimating human next-word predictability. Prior work has raised theoretical concerns about this perception, arguing that cloze predictions may reflect distinct cognitive processes from those recruited during real-time language comprehension (27, 106). Relatedly, some recent studies have found cloze estimates to underperform model-based predictability estimates in predicting human language processing measures (54, 55). Our results accord with these prior findings by showing that, when used as estimators of human reading effort, surprisal values from GPT-2 are, on average, at or beyond parity with cloze norms (based on the Provo dataset). Although additional research is needed to characterize the relative strengths of statistical vs. cloze predictability estimates in specific cases, our results suggest that the use of statistical predictability estimates, especially those from incremental transformer language models like GPT-2, should not generally be viewed as a design weakness relative to cloze norms in studies of language processing (see *SI Appendix*, 1 for extended discussion).

Although this comparison between GPT-2 and cloze may seem purely methodological, it is in fact bound up in our core theoretical question about the cognitive sources of word predictability effects. This is because of the asymmetric importance assigned by the facilitation vs. cost views to low-probability events, for which the cloze task (under realistic sample sizes) provides poor quality estimates. Under a facilitation (linear predictability) view, the main drivers of predictability effects are high-probability words. If this view is correct, then accurately estimating degrees of low probability is of little consequence, and cloze is the preferred estimator. Under a cost (logarithmic predictability, i.e., surprisal) view, the main drivers of predictability effects are low-probability words, since small absolute differences in low predictability can correspond to large differences in surprisal. If this view is correct, then accurately estimating degrees of low probability is essential, and cloze is not the preferred estimator. Therefore, one consequence of the cost view is that accurately estimating fine-grained differences in low probability (via e.g., GPT-2) should be more important than accurately estimating human subjective probabilities within the high-probability regime (via cloze). Our results support this position.

### Conclusion.

In conclusion, using recent advances in computational language modeling and time series analysis, and using diverse large-scale naturalistic reading datasets, our results support a logarithmic effect of word predictability on processing difficulty (1), and therefore support probabilistic inference as a core component of human language comprehension.

## Materials and Methods

### Data.

The datasets considered in this study span three modalities: self-paced reading, the Maze task, and eye-tracking during reading. In a self-paced reading task, participants are presented with texts in which words or characters are occluded until the participant reveals them one-by-one in left-to-right order by pressing a button. In a Maze task (107), like in a self-paced reading task, participants press buttons to progress word-by-word through a text. However, at each word position in the text, participants are presented with a forced choice between the true next word and a distractor, and they are tasked with selecting the correct continuation. In an eye-tracking during reading task, texts are presented on a screen to participants who read naturally, and their sequence of fixations to words in the text is recorded by an eye tracker.

The self-paced reading and Maze tasks yield a single word-by-word dependent variable: reading time (or reaction time, RT), that is, the time elapsed between stimulus presentation (a word in self-paced reading or a forced-choice decision in Maze) and pressing a button to indicate a decision (to reveal the next word in self-paced reading or to choose the continuation in Maze). Modeling eye movements during free reading is more challenging because the eyes do not progress linearly through the textual sequence of words. Studies of eye-tracking during reading have used a variety of measures derived from the reading record, each with a somewhat different cognitive interpretation (see e.g. ref. 108 for review).

In this study, we consider three different measures of fixation duration:

- Scan path duration, e.g., ref. 109. Time elapsed from when the eyes enter any word region from either the left or the right to when they next enter a different word region (either to the left or to the right), regardless of whether the fixation is a part of a regressive eye movement. This definition of scan path duration sums across all consecutive fixations to the same word region, since we do not wish to treat consecutive fixations to the same word as distinct events (a word should likely not influence our analyses three times more for having been viewed by three consecutive fixations rather than one). Under this definition (and unlike the first pass and go-past durations discussed below), a given experimental participant can have more than one observation associated with a given word token in the text (when words are refixated). For example, if a word sequence ABC is fixated in the order ACBBC, the scan path record will contain a sequence of four events: the duration of the fixation to A, followed by the duration of the first fixation to C, followed by the summed durations of the fixations to B, followed by the duration of the second fixation to C. Scan path durations thus encode the entire sequence of word fixations in time rather than textual order, including fixations that are part of regressive eye movements (e.g., refixations and fixations to words that were skipped in the initial pass). Regressive and nonregressive scan path events are distinguished in our analyses by a binary indicator variable (*SI Appendix*, 10).
- First pass duration, e.g., ref. 108. Time elapsed from when the eyes first enter a word region from the left to when they enter a different word region (either to the left or to the right). The sequence of first pass durations excludes all regressive eye movements, such that refixations or fixations to words that were skipped in the initial pass are not modeled.
- Go-past duration, e.g., ref. 98. Time elapsed from when the eyes first enter a word region from the left to when they enter a different word region to its right (including all intervening regressive fixations). Like first pass durations, the sequence of go-past durations excludes all regressive eye movements, such that refixations or fixations to words that were skipped in the initial pass are not modeled (except indirectly via their influence on go-past durations for words that were fixated in the initial pass).

Scan path and first pass durations are both early measures, restricted to the fixation duration of a single word (108). They differ only in whether regressive eye movements are included (scan path) or discarded (first pass). Go-past duration is a late measure designed to capture all processing (including regressive eye movements) involved in moving beyond the current “frontier” in progressing through the text.

In all eye tracking datasets except the GECO dataset (see below), a stimulus “event” is considered to be any fixation to a word region in the text. Thus, the full sequence of fixations before entering a target word region, regressive or nonregressive, is used to predict all three types of fixation duration at that region. Note that this differs from standard regression analyses of first-pass and go-past durations in eye-tracking data, which typically discard the full sequence of fixations and only consider the linear sequence of words. The ability to recruit the full scan path record to predict all response variables is an advantage of the deconvolutional regression approach described below.

In all datasets, following prior analyses of the Dundee and Natural Stories SPR datasets (109), we partition the data into training, validation, and test splits (approximately 50, 25, and 25%, respectively) using modular arithmetic on a split variable i, defined as a function of participant index p and sentence index s:

[1] $$\begin{array}{c} i=(s+p)\;\mathrm{mod}\;4, \end{array}$$

where datapoints are cycled into training if i∈{0,1}, validation if i=2, and test if i=3. Models are only fitted to data from the training set. Validation data are used for tuning and early stopping, following ref. 33. Test data are only used for statistical comparisons between models. Per ref. 109, to enable valid deconvolution, all data partitioning and filtering (see below) are applied only to the response vectors (the modeled reading times). The entire predictor matrix (sequence of word fixation features) is retained in all models.

The preprocessed datasets are available at https://osf.io/6wvqe/. For instructions on reproducing our preprocessing pipeline for the reading data, see https://github.com/coryshain/cdr.

#### Brown SPR.

The Brown SPR dataset (1) contains self-paced reading data from 35 participants reading short (292-902 word) passages from the Brown dataset of American English (110). The data can be accessed online at https://github.com/wilcoxeg/neural-networks-read-times.

The dataset contains a total of 450 sentences, 7,188 words, and 136,907 responses. Following established protocol for Natural Stories SPR (another self-paced reading dataset, described below), we remove sentence boundaries and RTs that were less than 100 ms or greater than 3,000 ms.

#### Dundee ET.

The Dundee ET dataset (111) contains eye-tracking data from 10 participants who read newspaper articles from The Independent on a computer monitor. The data can be accessed online at https://github.com/wilcoxeg/neural-networks-read-times.

The dataset contains a total of 2,388 sentences, 51,501 words, and 408,439 distinct fixations to word regions on the screen. The responses in the Dundee dataset are filtered to exclude fixations following large outlier saccades (>20 words in either direction), based on the assumption that such outliers reflect track loss or inattention, rather than language processing. Following prior work, e.g., ref. 108, we also remove fixations to words adjacent to a screen, line, or sentence boundary, as well as fixations interrupted by blinks.

#### GECO ET.

The GECO ET dataset (36) contains eye-tracking data from participants who read *The Mysterious Affair at Styles* by Agatha Christie on a computer monitor. The full dataset contains data from 19 Dutch-English bilinguals who read the first half of the novel in either Dutch or English and the second half in the other language, along with data from 14 English monolinguals who read the entire novel in English. Because the computational language models used in this study are English-specific, here we only used the data from the 14 monolingual English readers. Unlike the other ET datasets analyzed in this study, the GECO dataset does not provide the full scan path record, but only a distilled format that contains first pass and go-past times by word. Thus, in the case of GECO, we do not analyze scan path durations, and we treat each fixated word in textual order as a stimulus “event” (rather than individual fixations) for the purposes of deconvolution. The data can be accessed online at https://expsy.ugent.be/downloads/geco/.

The portion of the dataset that we analyzed contains a total of 5,300 sentences, 56,440 words, and 374,179 events. Following the Dundee protocol (above), the responses in the GECO dataset are filtered to exclude fixations following large outlier saccades (>20 words in either direction) and fixations to sentence boundaries (screen and line boundaries were not annotated).

#### Natural stories SPR.

The Natural Stories SPR dataset (37) contains crowd-sourced self-paced reading responses from 178 participants to 10 naturally occurring narrative or nonfiction pieces modified in order to over-represent rare words and syntactic constructions without compromising perceived naturalness. The stimuli are thus designed to reflect the typical conditions of story comprehension, while subtly taxing the language processing system. The data can be accessed online at https://github.com/languageMIT/naturalstories.

The dataset contains a total of 485 sentences, 10,256 words, and 1,013,377 responses. Following previous work, e.g., ref. 109, RTs are removed if they are less than 100 ms or greater than 3,000 ms, if they are to words adjacent to a sentence boundary, if participants answered less than 5/8 comprehension questions correctly, or if, subject to the aforementioned constraints, participants have fewer than 100 RTs.

#### Natural stories maze.

The Natural Stories Maze dataset (38) contains crowd-sourced Maze task responses from 95 participants to the same materials as in the Natural Stories SPR dataset above, using a recently developed technique (A-Maze) to generate high-quality forced-choice alternatives for long naturalistic passages (112). The data can be accessed online at https://github.com/vboyce/amaze-natural-stories.

The dataset contains a total of 97,527 responses (the textual statistics are the same as Natural Stories SPR above). Following ref. 38, RTs are removed if they are less than 100 ms or greater than 5,000 ms, if they are to words adjacent to a sentence boundary, or if the subject responded incorrectly (i.e., selected the wrong continuation). Inattentive subjects (defined as subjects with lower than 80% accuracy) are also removed.

#### Provo ET.

The Provo ET dataset (39) contains eye-tracking data from 84 participants who read 55 short (39 to 62 word) passages from various online sources on a computer monitor. The data can be accessed online at https://osf.io/sjefs/.

The dataset contains a total of 134 sentences, 2,745 words, and 213,224 distinct fixations to word regions on the screen. Following the Dundee protocol (above), responses are filtered to exclude fixations following large outlier saccades (>20 words in either direction), fixations to words adjacent to a sentence boundary (screen and line boundaries were not annotated), and fixations interrupted by blinks.

### Surprisal Estimates.

We obtain the surprisal estimates used in our experiments from three different families of language models. First, we consider surprisal estimates derived from an *n*-gram model, a simple count-based method that estimates word probabilities by interpolating over prefix counts up to a fixed length, estimated from large text corpora. Many prior studies have reported *n*-gram effects in human language processing, (e.g., refs. 1, 12, and 113 inter alia). We compute *n*-gram surprisal values using a 5-gram model estimated on the WikiText-103 dataset (114)—a large, popular language modeling dataset extracted from Wikipedia—with Kneser–Essen–Ney smoothing (115). Model parameters are estimated using the KenLM (116) library with default hyperparameter settings.

Second, we consider surprisal estimates from a probabilistic context-free grammar (PCFG) parser, which conditions its next-word predictions on hypotheses about the syntactic structure of the sentence, rather than on the preceding word sequence. Although incremental generative parsers generally perform poorly as language models due to their highly constrained representation of context, recent work has shown that they perform unexpectedly well as models of measures of sentence processing (48). Our PCFG (41) is trained on a generalized categorial grammar reannotation (117) of the Penn Treebank (118).

Third, we consider surprisal estimates from large autoregressive language models based on the transformer architecture (42), namely GPT-2(-small) (43), GPT-J (44), and GPT-3 (45). These models generate next-word predictions via a deep neural network transform of the linguistic context (preceding word sequence). Recent work has shown strong alignment between autoregressive transformer representations and measures of human sentence processing, both behavioral (101) and neural (119). GPT-2 is a 124M parameter model that has been open-sourced through the Hugging Face library (120). We generate GPT-2 surprisals using the default Hugging Face implementation of GPT-2 (GPT-2-small). At the time we conducted this study, GPT-J was among the largest fully open-source transformer language models, with 6B parameters. Open-source models are a critical asset to repeatable science since their weights and training data are available for direct inspection, and the inclusion of GPT-J therefore allows us to incorporate more recent advances in language modeling since the release of GPT-2 without compromising replicability. GPT-3 is a large (175B parameter) proprietary commercial language model trained on proprietary data, and its weights have not been publicly released. At the time we conducted this study, access to GPT-3 surprisal estimates was only available through a paid service. Considering GPT-3 surprisal allows us to explore more recent advances in language modeling, at the expense of full replicability given the reliance on a proprietary model. In this study, we use GPT-3-davinci-002.

Before computing the GPT-2 and GPT-J surprisal estimates, text from all corpora is pre-processed using the Moses decoder (http://www.statmt.org/moses/) tokenizer and punctuation normalizer. Capitalization is kept intact. No text preprocessing is used for GPT-3. Note that additional tokenization is performed internally by the tokenizers associated with each of the neural models (likewise provided either by the Hugging Face library for GPT-2 and GPT-J and by the OpenAI API for GPT-3). Because of these tokenization protocols, transformer language models sometimes predict at the level of subwords. To align surprisal values from transformers to word tokens, we therefore sum surprisal values across tokens within each word to generate a word-level value. This procedure is licensed by the chain rule. Texts were entered to each model in their entirety when possible (except in the case of the PCFG, which requires sentence-tokenized text). In cases where text length exceeded the maximum allowed by the model, we used a sliding window approach guaranteeing at least 200 words of context per prediction. Code for reproducing our *n*-gram, GPT-2, and GPT-J estimates is available at https://github.com/rycolab/revisiting-uid. Code for reproducing the PCFG and GPT-3 estimates is available at https://osf.io/6wvqe/.

Models also include a number of control predictors described in *SI Appendix*, 10; see *SI Appendix*, 11 for detailed model formulae. The preprocessed datasets, including all control and surprisal predictors, are available at https://osf.io/6wvqe/.

### Analysis.

#### Continuous-time deconvolutional regression.

All analyses use continuous-time deconvolutional regressive neural networks (CDRNNs; 32, 33); see *SI Appendix*, 12 for a formal definition of the regression model. In brief, CDRNNs convolve the recent history of predictors (word features) in the experiment with continuous-time filters generated by deep neural networks in order to parameterize the distribution over the response (e.g., scan path duration) at a point in time. CDRNNs thus implicitly estimate continuous-time impulse response functions (IRFs) representing the effect of an impulse (a word) on the response (comprehension difficulty) at some delay. The properties of these IRFs can be queried using a combination of perturbation analysis (121) and Monte Carlo dropout (122), enabling interpretation of a black box deep neural model. Unlike standard approaches to time series regression like linear mixed-effects models (LMEs; 123) and generalized additive models (GAMs; 34), CDRNNs simultaneously relax assumptions that the IRF is discrete-time, linear, and stationary (time-invariant), all within a distributional regression framework, e.g., ref. 124 that captures stimulus-driven effects on all parameters of the distribution over the dependent measure, not just its expected value. Critically, CDRNNs can be constrained to enforce linearity for certain predictors, permitting statistical evaluation of nonlinearity by comparing the fit of models that relax or enforce it. Full description of the CDRNN approach can be found in ref. 33. CDRNN implementation details used in this study are described in *SI Appendix*, 13. Code for reproducing all analyses in this study can be found at https://github.com/coryshain/cdr. See *SI Appendix*, 14 for evidence that more commonly used generalized additive models (GAMs) yield similar results to our own.

#### Response distribution.

Because the distribution of reading times is known to be heavily right skewed, (e.g., ref. 8), we assume an exGaussian response distribution, (see e.g., refs. 5 and 125 for evidence that the exGaussian provides a strong distributional fit to human sentence reading). The exGaussian has three parameters: location (μ), dispersion (σ), and skewness (τ), where location, dispersion, and skewness all increase on their respective parameters. The quantity of interest targeted in this study is the influence of word probability estimates on the mean of this predictive distribution, where the mean depends linearly on the location and skewness parameters:

[2] $$\begin{array}{c} E_{F(\mu ,\sigma ,\tau )}\left(X\right)=\mu +\tau . \end{array}$$

Thus, a linear influence of surprisal on either μ or τ will yield a linear influence of surprisal on the mean of the response distribution. See *SI Appendix*, 15 for evidence both that assuming an exGaussian response substantially improves model fit over assuming a normal response and that similar findings to our main results still obtain when assuming normally distributed reading times.

#### Baseline models.

The main CDRNN models in this study are fully nonlinear on surprisal and can thus find any functional form (f(surp)) to a range of control models. The baseline model contains no predictability effect of any kind and thus provides a reference for the overall effect of including a predictability measure. The prob model is constrained to be linear on probability, rather than surprisal, as predicted by some theories, e.g., ref. 3. The surp^1/2^, surp^3/4^, surp^1^, surp^4/3^, and surp^2^ are constrained to be linear on some power of surprisal (denoted in superscript) and thus represent a cline of functional forms for the predictability effect, from sublogarithmic (surp^1/2^) to logarithmic (surp^1^) to superlogarithmic (surp^2^).

#### Statistical procedure.

Statistical testing within our continuous-time deconvolutional framework relies on out-of-sample model comparison: Models instantiating the null vs. alternative hypotheses are trained on a portion of the data (training set), and conditional likelihoods from these models over an unseen portion of the data (test set) are statistically compared in order to determine whether the model instantiating the alternative hypothesis generalizes better than the model instantiating the null hypothesis (109). All results reported in this study are based in ensembles of 10 models, which reduces variability in effect estimation and predictive performance due to stochastic initialization and optimization. Following ref. 33, ensembles are compared using paired permutation tests of out-of-sample conditional likelihood. Full details of the testing protocol are described in *SI Appendix*, 13.

## Supplementary Material

Appendix 01 (PDF)

## Data Availability

Previously published data were used for this work (1, 35–39).
