# Supplementary material for: Large-scale evidence for logarithmic effects of word predictability on reading time
Source: Proc Natl Acad Sci U S A. 2024 Feb 29;121(10):e2307876121. doi: 10.1073/pnas.2307876121 (PMC10927576; doi:10.1073/pnas.2307876121)
Supplement: Supplementary file 1 — Appendix 01 (PDF) [file pnas.2307876121.sapp.pdf]

PNAS

Supporting Information for

Large-scale evidence for logarithmic effects of word predictability on reading time

Cory Shain, Clara Meister, Tiago Pimentel, Ryan Cotterell, and Roger Levy

Corresponding Author: Cory Shain.
E-mail: cshain@mit.edu

This PDF file includes:

Supporting text
Figs. S1 to S11
Tables S1 to S15
SI References

Contents

1 Revisiting the Linear Predictability Effects of Brothers & Kuperberg (2021) **3**
A Background. 3
B Methods. 4
C Results. 4
C.1 What Is the Estimated Shape of Predictability Effects? 4
C.2 Is Processing Difficulty Linear or Logarithmic on Word Predictability in Brothers and Kuperberg's Experiments? 6
C.3 How Do Cloze-Based and Model-Based Predictability Estimates Differ? 8
D Discussion. 9

2 Analysis of Word Skipping Behavior **13**
A Methods. 13
B Results. 13
C Discussion. 16

3 Visualization of Word Length and Frequency Effects **16**

4 Full Significance Testing Results **18**

5 Full IRF Surface Plots **24**

6 Direct Comparison between GPT-2 and PCFG Language Models **28**

7 Are Logarithmic Predictability Effects Recovered from a Non-Logarithmic Scale? **28**

8 Comparison to Hoover et al. (2023) **29**

9 Language Model Perplexity **31**

10 Statistical Controls **33**

11 Model Formulae **33**

12 CDRNN Model Definition **34**

13 CDRNN Implementation and Statistical Procedure **35**

14 Revisiting Our Main Findings Using Generalized Additive Models (GAMs) **36**

15 Reanalysis Using Normal Error **44**

Supporting Information Text

1. Revisiting the Linear Predictability Effects of Brothers & Kuperberg (2021)

The strongest current empirical support for linear (rather than our claimed logarithmic) predictability effects on processing difficulty comes from Brothers and Kuperberg (1), who report a striking convergence of evidence from three independent experiments. Brothers and Kuperberg's study is carefully implemented, with two key design features differentiating their approach from most other work on this question (including ours, but also 2–5): (i) they used a normed predictability manipulation in a critical region of constructed stimulus sentences (i.e., an *experimental* or *controlled* study) rather than word-by-word modeling of responses to uncontrolled text (i.e., *naturalistic*, *corpus*, *observational*, or *correlational* studies, such as ours), and (ii) they used large-sample ($n \approx 90$) cloze-based predictability estimates in these critical regions, rather than model-based predictability estimates. These design features naturally travel together: word-by-word cloze-norming of free text is a large and rarely-attempted undertaking (though see 6). Thus, barring an unusually large investment of resources, cloze-norming is for practical purposes out of reach for naturalistic studies, but feasible for experimental studies, which have a relatively small number of critical regions to norm.

Citing (7–10), Brothers and Kuperberg argue that these design features are an advantage over studies that use (i) naturalistic stimuli and (ii) model-based statistical predictability estimates (including our study, by extension). They place special emphasis on (i), arguing in the article (and at length in an appendix) that naturalistic studies of language processing are especially susceptible to the correlation-causation problem: uncontrolled texts exhibit extensive covariation in linguistic features like length, frequency, predictability, syntactic complexity, semantic properties, etc., the underlying causes of which are still poorly understood. Naturalistic studies by definition lack experimental control for these confounds, and controlling for all plausible confounds statistically is difficult (or even impossible) given the complexity of the system. Furthermore, implemented statistical models require many assumptions about the set of relevant control variables and about the relationships among both the control variables and the response; these assumptions may lack strong theoretical or empirical support. These concerns are argued to make causal inference inherently less reliable from correlational studies than from experimental studies. Importantly in light of these arguments, across three experiments (self-paced reading, picture naming, and a meta-analysis of controlled studies of predictability effects) Brothers and Kuperberg found a linear rather than a logarithmic form for the predictability–cost relationship. Is the logarithmic effect found by our study (and others) merely an artifact of uncontrolled confounds in naturalistic reading, as suggested by Brothers and Kuperberg?

Here we address this concern by reanalyzing data from Brothers and Kuperberg's two key experiments (self-paced reading and picture naming) and attempting to nuance the arguments outlined above. In brief, (1) we show that Brothers and Kuperberg's experimental data are more equivocal than the impression given by the original study, supporting a logarithmic (rather than linear) predictability effect when modeled using GPT-2 (the language model with the best psychometric performance in our own study), and (2) we argue that Brothers and Kuperberg's advocacy for their design is somewhat selective: considering the full range of relative strengths and weaknesses for e.g., experimental vs. naturalistic studies and cloze vs. model-based predictability estimates results in a more ambivalent picture, without one unambiguously more reliable design.

A. Background. Brothers and Kuperberg constructed 648 stimulus sentences (9 to 16 words long, mean 13 words) representing low-cloze (less predictable), moderate-cloze, and high-cloze (more predictable) contexts for each of 216 critical words. The critical word was always followed by at least two additional words. An example item set is given below, with the critical word **glasses** in bold:

**High-cloze:**	Her vision is terrible and she has to wear **glasses** in class.
**Moderate-cloze:**	She looks very different when she has to wear **glasses** in class.
**Low-cloze:**	Her mother was adamant that she has to wear **glasses** in class.

As the example makes clear, stimuli were constructed to read like natural sentences while carefully matching on length as well as left and right context of the critical word. Participants in all experiments saw each target word in at most one condition. Cloze probabilities for the critical word of each stimulus were acquired in a crowd-sourced cloze completion task with about 90 completions per context, yielding an average resolution of $p = 1/90 \approx 0.01$ for the resulting predictability estimates. These stimuli were then used in a crowd-sourced self-paced reading (SPR) experiment (**Experiment 1**, $n = 216$) in which the dependent variable was summed reading time over a 3-word critical region starting at the critical word (the two additional words were included to capture spillover effects). A subset of 84 of these items (all with nouns as the critical word) were also used in an in-person cross-modal picture naming experiment (**Experiment 2**, $n = 36$) in which participants listened to an audio recording of the sentence prefix (up to but not including the critical word) being read aloud and were tasked with naming a picture of the critical word presented at a delay of 250ms (here the dependent variable was naming response time). As Brothers and Kuperberg acknowledge (p. 6), the naming experiment differs more than the SPR experiment from the normal conditions of word-by-word reading: it is an offline, explicit task that plausibly engages distinct cognitive processes (e.g., conscious reflection, visual object recognition, articulation) from those engaged in incremental language comprehension. It was included in the original study as a conceptual replication of the SPR experiment. Data from both experiments were analyzed using generalized additive models (GAMs; 11) to infer the form of the predictability–cost relationship from data and using linear mixed-effects models (LMEs; 12) to test prior hypotheses about this form (namely, linear vs. logarithmic). Brothers and Kuperberg also conducted a meta-analysis (**Study 3**) of 8 experiments across 5 prior studies in which they

made inferences about the functional form of the predictability–cost relationship from condition-wise effect sizes (i.e., for high-, moderate-, and low-cloze conditions). We refer readers to Brothers and Kuperberg's study for full methodological details.

Brothers and Kuperberg's findings converged across experiments to support a linear over a logarithmic predictability effect. In both the SPR and naming experiments, GAM-estimated smoothing splines for the relationship between predictability and response time were approximately linear in cloze probability and sublogarithmic in cloze surprisal (our analyses reproduce this finding, see the top row of **Figure S1A**), and LME models showed better fit to the data when cloze was represented linearly rather than logarithmically (our analyses also reproduce this finding, see rows "$\text{Cloze}_{\text{SURP}^1}$ vs. $\text{Cloze}_{\text{PROB}}$" and "$\text{Cloze}_{\text{PROB}}$+$\text{Cloze}_{\text{SURP}^1}$ vs. $\text{Cloze}_{\text{SURP}^1}$" of **Table S1**). And in the meta-analysis, they found that the relative effect sizes reported for high-, moderate-, and low-cloze conditions in prior studies of predictability effects were more consistent with a linear than a logarithmic predictability effect. The authors made the data from these experiments and many of the analysis scripts publicly available: https://osf.io/b9kns/. We refer readers to Brothers and Kuperberg's study for full results.

B. Methods. Here we use Brothers and Kuperberg's public data release to revisit findings from the SPR and naming experiments. We leave the meta-analysis aside since the data release includes only aggregate measures, not the item-level stimulus and response data needed to e.g., reanalyze the data using model-based predictability estimates. Because the SPR task is more similar to incremental language comprehension than the cross-modal picture naming task (see above), we focus primarily on the SPR data, with the naming data included in our reanalysis for completeness. We follow Brothers and Kuperberg in using GAMs implemented by the `mgcv` package in `R` (11) to estimate the functional form of predictability effects and LMEs implemented by the `lme4` package in `R` (12) for statistical comparisons between prior hypotheses (e.g., linear vs. logarithmic predictability effects). We chose these methods over the CDRNNs used in our main study both to improve comparability to Brothers and Kuperberg's results and because the public data release lacks word-level timing information; as a result, continuous-time deconvolution is not possible.

However, we go beyond the original study in two key ways. *First*, we use GAMs to additionally estimate predictability effects under other predictability models, not just cloze:

1. The **trigram** predictability estimates used as a control in Brothers and Kuperberg's original study.

2. **GPT-2** predictability estimates computed by applying the same procedures from our main study to the stimuli used by Brothers and Kuperberg.

3. **GPT-2-region** predictability, i.e., the product of probabilities (sum of surprisals) assigned by GPT-2 to the entire 3-word critical region analyzed in the SPR experiment. This variant is motivated by concern that responses in the critical region may be driven not only by spillover from the first (critical) word in the region, but also by the surprisals of the remaining words in the region (the critical region is lexically matched within item sets but not across them). Note that this measure only applies to the SPR experiment, since participants in the naming experiment did not hear the critical word or any words that followed it.

Because Brothers and Kuperberg have not as of this writing released code for their GAM models, we approximate their implementation based on the description in their article (our implementation can be found in our public repository: https://github.com/coryshain/cdr). We closely reproduce their visualizations (**Figure S1A**).

Second, we perform statistical comparisons using out-of-sample likelihood, as in our main analyses. By contrast, the key comparison in Brothers and Kuperberg (linear vs. logarithmic cloze effects) is based on numerical differences in in-sample likelihood. We use 5-fold cross-validation to estimate an LME on 4 folds of data and use the estimated model to assign a likelihood to the held-out fold. Roughly equally-sized folds were created by cycling stimulus sentences into folds based on modular arithmetic (mod 5) applied to their numerical IDs (these IDs are unrelated to order of presentation, which was randomized). Differences in cross-validated out-of-sample likelihood are permutation-tested for significance, allowing us to perform both non-nested comparisons (e.g., linear vs. logarithmic effects of some predictability estimate) and nested comparisons (e.g., the unique contribution of adding a logarithmic effect to a model containing a linear one).

Following Brothers and Kuperberg, we only include the key predictability variable(s) in each model. Follow-up analyses with additional controls (critical word length, summed critical region word length, critical word unigram surprisal, summed critical region unigram surprisal, critical word position in the sentence, and mean semantic distance of the critical word from content words in the preceding context) had little impact either on visualizations or measures of model fit, and we do not report on them further. All models include random intercepts by participant and item (richer random effects structures—e.g., with random slopes by participant for each predictor—led to frequent convergence errors).

About 15% of the time, the critical word in an item was not produced by any participant in the norming experiment, resulting in a cloze probability of 0. Because the logarithm (and thus, the surprisal) of cloze probability for these items is undefined, we follow Brothers and Kuperberg in assigning half a response (out of an average of 90 responses per item) to these items, resulting in a minimum cloze probability of about 0.01 and a maximum cloze surprisal of about 5.2.

C. Results.

C.1. What Is the Estimated Shape of Predictability Effects? Results from the GAM models are visualized in **Figure S1A**. Estimates using cloze probability/surprisal (top row of the figure) closely match those reported by Brothers and Kuperberg and support a linear predictability effect: despite the flexibility of GAM regression, models find a clear straight-line effect of cloze probability

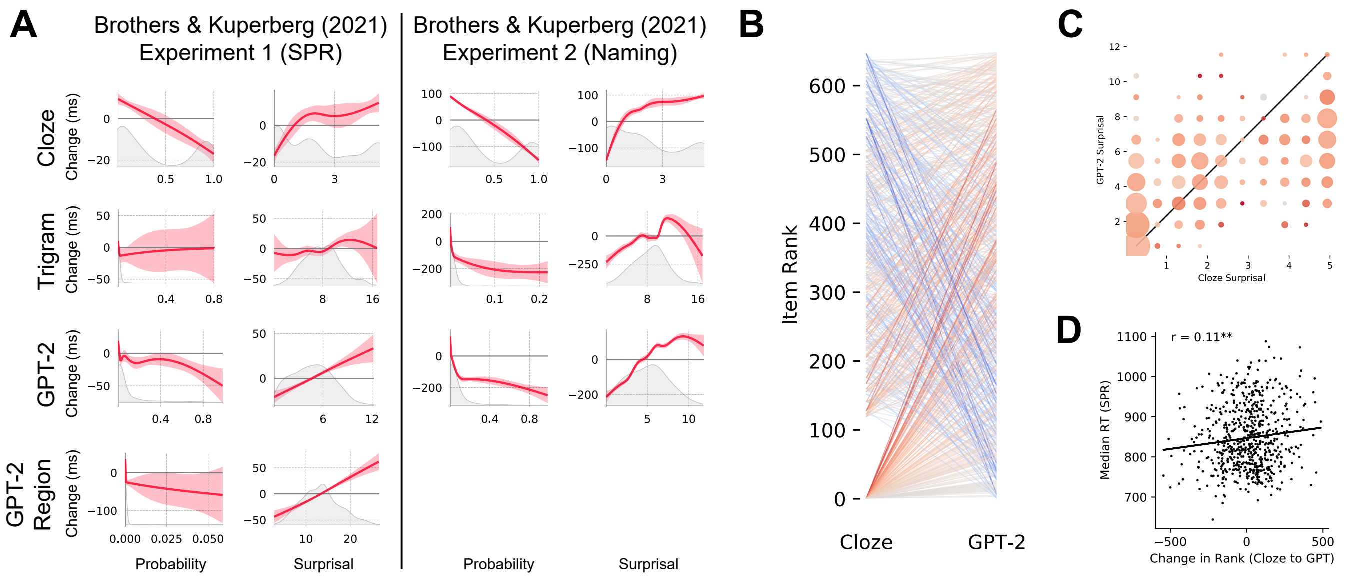

Fig. S1. A. GAM-estimated functional form of effects in Brothers' & Kuperberg's (1) Experiment 1 (SPR) and Experiment 2 (cross-modal picture naming) across language model types (human cloze and trigram from the original study, GPT-2 predictability of the critical word, and GPT-2 predictability of the whole 3-word critical region). Plots cover the full empirical range of predictor values in each training dataset. Kernel density plots show the distribution of predictor values in the training data over the plotted range. Uncertainty intervals show the $\pm$ 2 standard errors used as a default in plots from `mgcv` (unlike CDRNN plots, which show 95% credible intervals). **B.** Change in rank for Brothers' and Kuperberg's 648 stimuli as a function of cloze probability (left) vs. GPT-2 probability (right). Color reflects the slope of the change in rank (red for positive, blue for negative). **C.** The distribution of items and reading latencies in Brothers and Kuperberg's SPR experiment as a function of cloze surprisal vs. GPT-2 surprisal. Point size represents the number of items that fall into the corresponding region of the cloze surprisal vs. GPT-2 surprisal coordinate space. Point color represents the median RT of those items in the SPR experiment (darker red indexes longer RTs). **D.** Median RT by item in Brothers and Kuperberg's SPR experiment as a function of the item's rank change in surprisal as estimated by GPT-2, relative to cloze.

on response times in both experiments, and, when given surprisal-scale cloze values instead, models find the characteristic "plateau" at high surprisal values, as expected when a linear predictability effect is projected onto a logarithmic scale (**Figure 1**). However, results using model-based predictability estimates (bottom three rows of the figure) show a strikingly different pattern. In the SPR experiment, GPT-2-based surprisal effects are strongly linear (trigram effects are too uncertain to be informative). In the naming experiment, model-based surprisal effects (including from Brothers and Kuperberg's own trigram measures) are mostly consistent with a linear effect, except with a downward curve at high surprisal values where there are few datapoints (see the superposed density plots of **Figure S1A**); GAM models sometimes show a similar downward curve at high surprisal values in our own data (**Figure S10**). Despite this downward curve, model-based estimates for the naming experiment are inconsistent with a linear predictability effect. For example, the estimated GPT-2 effect is essentially logarithmic (linear in surprisal) over almost 10 orders of magnitude on a natural log scale (from $p = 1$ to approximately $p = 0.0005$), an interval that contains most of the data. Furthermore, when we examine trigram- and GPT-2-based effects estimated on a probability scale (left column of **Figure S1A**), both experiments show the characteristic spike in estimated processing cost as probability approaches zero from the right, as expected when a logarithmic predictability effect is projected onto a linear scale (**Figure 1**). The upshot of these visualizations is that the disagreement between Brothers and Kuperberg's findings of linearity and the logarithmic findings of other studies (such as ours) is not due primarily to misalignment between experimental and naturalistic approaches, but instead due primarily to the choice of predictability model: Brothers and Kuperberg's conclusions from these experiments depend critically on using cloze to represent predictability, whereas model-based analyses of the same data support a logarithmic pattern.

This could be because models underestimate the degree to which high-cloze words are predictable to humans, as Brothers and Kuperberg argue (pp. 5–6). We agree that this is true in some cases, but it is not the whole story, at least for the (full-context) GPT-2 model. *First*, GPT-2 does capture some high-cloze items (as shown by the fact that some GPT-2 probabilities fall near 1). *Second*, cloze may *overestimate* humans' certainty about future words during real-time comprehension due to task-specific strategic effects (13), such as a prototypicality bias (14), that may suppress variance in (explicit) cloze responses relative to (implicit) expectations during comprehension. *Third*, as shown in **Figure S1B**, GPT-2 does not merely suppress cloze probabilities (which would result in stable item ranks between cloze and GPT-2), nor does it fail to capture human cloze intuitions altogether (Spearman rank correlation between cloze and GPT-2 probabilities for these stimuli is 0.55). Instead, the relationship is complex, with much shared structure between GPT-2 and human cloze, but also qualitative differences *in both directions*, with both (a) many high-cloze items given low probabilities by GPT-2 (consistent with Brothers and Kuperberg's framing), but also (b) many low-cloze items given high probabilities by GPT-2, as shown by the many red lines representing items with cloze near zero that GPT-2 predicts with high probability. GPT-2's rank changes confer a modeling benefit relative to cloze, as shown in **Figure S1D**, which plots the median RT by item in Brothers and Kuperberg's SPR experiment as a function of the item's rank change in surprisal from cloze to GPT-2. As shown, there is a weak but significant trend whereby items that are ranked as more surprising by GPT-2 relative to cloze also take longer to read. Furthermore, when we bin items by their cloze vs. GPT-2 surprisal values **Figure S1C**, we find that some items in the middle of the cloze surprisal range have both long reading times (dark red color) and high GPT-2 surprisal. This is important because moderate-surprisal items are critical to function estimation: if the predictability model overestimates the predictability of some items, this could push items with longer reading times leftward along the surprisal scale, potentially giving rise to a more plateau-like effect as predicted by the FACILITATION view (**Figure 1**).

Thus, we find evidence that GPT-2 contains potentially important signal about human subjective predictability during incremental language comprehension that may not be fully captured by cloze estimates. In **C.3** we present more detailed qualitative analyses of these differences.

C.2. Is Processing Difficulty Linear or Logarithmic on Word Predictability in Brothers and Kuperberg's Experiments? We now evaluate how the patterns visualized in **Figure S1A** are reflected in model fit to unseen response time data, as summarized in **Table S1**. Predictability effects are generally significant over baseline ($\emptyset$) across experiments and predictability measures, with the key exception in the SPR study of the trigram estimates provided in the data release. In other words, predictability effects are indeed robust in these experiments, but Brothers and Kuperberg's trigram estimates are a poor proxy for response times (especially in SPR), which perhaps contributed to their failure to explain variance over cloze in Brothers and Kuperberg's SPR analyses. This limitation is addressed by GPT-2, which provides a strong fit to response times in both experiments. Regarding comparisons between linear and logarithmic variants of the same predictability measures, results replicate Brothers and Kuperberg's key findings even using our more stringent held-out tests: in both experiments, cloze probability outperforms cloze surprisal, and cloze probability contributes significantly when added to a model containing cloze surprisal alone, whereas cloze surprisal does not contribute significantly when added to a model containing cloze probability alone. Thus, as in the visualizations above, cloze-based results support a linear predictability effect. However, model-based results again show the opposite pattern: across experients and predictability models, a surprisal scale numerically outperforms a probability scale, significantly so in GPT-2-based comparisons. Moreover, in GPT-2-based models for both experiments, surprisal contributes significantly when added to a model containing probability alone, but probability does not contribute significantly when added to a model containing surprisal alone.

Which of these patterns is to be believed? Perhaps Brothers and Kuperberg are right that cloze is a better approximation of human subjective predictability than corpus estimates, and therefore that results based on cloze should be trusted more. The SPR results undermine this position: although cloze probability outperforms trigram surprisal as in Brothers and Kuperberg's own analyses, there is no significant difference in fit between cloze probability and either GPT-2 or GPT-2-Region. By contrast,

	Comparison	SPR Experiment ΔLL	SPR Experiment p	Naming Experiment ΔLL	Naming Experiment p
Overall	Cloze$_{\text{PROB}}$ vs. $\emptyset$	**83**	**0.0012**	**1477**	**0.0006**
	Cloze$_{\text{SURP}^1}$ vs. $\emptyset$	**62**	**0.0012**	**1077**	**0.0006**
	Trigram$_{\text{PROB}}$ vs. $\emptyset$	-4	—	**44**	**0.0006**
	Trigram$_{\text{SURP}^1}$ vs. $\emptyset$	2	1.0000	**46**	**0.0038**
	GPT-2$_{\text{PROB}}$ vs. $\emptyset$	**37**	**0.0007**	**402**	**0.0005**
	GPT-2$_{\text{SURP}^1}$ vs. $\emptyset$	**74**	**0.0007**	**836**	**0.0005**
	GPT-2-Region$_{\text{PROB}}$ vs. $\emptyset$	-1	—		
	GPT-2-Region$_{\text{SURP}^1}$ vs. $\emptyset$	**73**	**0.0007**		
Probability vs. Surprisal	Cloze$_{\text{SURP}^1}$ vs. Cloze$_{\text{PROB}}$	**-21**	**0.0107**	**-400**	**0.0011**
	Trigram$_{\text{SURP}^1}$ vs. Trigram$_{\text{PROB}}$	6	0.1506	2	1.0000
	GPT-2$_{\text{SURP}^1}$ vs. GPT-2$_{\text{PROB}}$	**37**	**0.0012**	**434**	**0.0007**
	GPT-2-Region$_{\text{SURP}^1}$ vs. GPT-2-Region$_{\text{PROB}}$	**74**	**0.0012**		
	Cloze$_{\text{PROB}}$-Cloze$_{\text{SURP}^1}$ vs. Cloze$_{\text{SURP}^1}$	**20**	**0.0107**	**395**	**0.0007**
	Cloze$_{\text{PROB}}$-Cloze$_{\text{SURP}^1}$ vs. Cloze$_{\text{PROB}}$	-1	—	-5	—
	Trigram$_{\text{PROB}}$-Trigram$_{\text{SURP}^1}$ vs. Trigram$_{\text{SURP}^1}$	-2	—	5	1.0000
	Trigram$_{\text{PROB}}$-Trigram$_{\text{SURP}^1}$ vs. Trigram$_{\text{PROB}}$	5	0.6184	7	1.0000
	GPT-2$_{\text{PROB}}$-GPT-2$_{\text{SURP}^1}$ vs. GPT-2$_{\text{SURP}^1}$	-2	—	18	0.0600
	GPT-2$_{\text{PROB}}$-GPT-2$_{\text{SURP}^1}$ vs. GPT-2$_{\text{PROB}}$	**35**	**0.0012**	**452**	**0.0007**
	GPT-2-Region$_{\text{PROB}}$+GPT-2-Region$_{\text{SURP}^1}$ vs. GPT-2-Region$_{\text{SURP}^1}$	-24	—		
	GPT-2-Region$_{\text{PROB}}$+GPT-2-Region$_{\text{SURP}^1}$ vs. GPT-2-Region$_{\text{PROB}}$	**50**	**0.0107**		
Cloze vs. Other	Trigram$_{\text{SURP}^1}$ vs. Cloze$_{\text{PROB}}$	**-81**	**0.0015**	**-1431**	**0.0005**
	Cloze$_{\text{PROB}}$+Trigram$_{\text{SURP}^1}$ vs. Cloze$_{\text{PROB}}$	-4	—	4	0.2979
	GPT-2$_{\text{SURP}^1}$ vs. Cloze$_{\text{PROB}}$	-9	0.9841	**-641**	**0.0005**
	Cloze$_{\text{PROB}}$+GPT-2$_{\text{SURP}^1}$ vs. Cloze$_{\text{PROB}}$	4	0.6076	**16**	**0.0276**
	GPT-2-Region$_{\text{SURP}^1}$ vs. Cloze$_{\text{PROB}}$	-10	1.0000		
	Cloze$_{\text{PROB}}$+GPT-2-Region$_{\text{SURP}^1}$ vs. Cloze$_{\text{PROB}}$	0	1.0000		

Table S1. Testing Results on Data from Brothers' & Kuperberg's (1) Experiment 1 (SPR) and Experiment 2 (cross-modal picture naming). Results of key statistical comparisons using permutation tests of the difference in average cross-validated log likelihood (ΔLL) between linear mixed effects (LME) models that assume some predictability estimate and functional form (PROB is linear in probability and SURP^1 is linear in surprisal). Boldface indicates statistical significance. In directional tests where one hypothesis subsumes the other (e.g., PROB vs. $\emptyset$, since a nonlinear surprisal effect subsumes a linear one), dashes (—) indicate failure of the alternative hypothesis (left) to improve over the null hypothesis (right). Comparisons involving GPT-2-Region are left empty in the column for the naming experiment, where this measure is not relevant. Otherwise, cells are color coded using cyan to indicate that the hypothesis on the left outperforms the one on the right, and using magenta to indicate that the hypothesis on the right outperforms the one on the left. All p-values are corrected for false discovery rate (15) across all tests within family of tests (delimited by single horizontal lines) for each experiment. Key comparisons are highlighted.

Rank change	Critical word	Stimulus
-545	leaves	Bill raked up all the leaves in front of his condo.
-511	spider	The web had been spun by the large spider on our porch.
-500	ring	Before proposing to his girlfriend he would need a ring with a big diamond.
-493	match	The gentleman lit the candle using a single match which was surprising.
-443	star	The hopeful girl wished upon a star up in the sky.
-441	beak	My son saw the bird pecking at the soil using its beak and claws.
-437	yearbook	To record some high school memories, he had everyone sign his yearbook after graduation.
-425	grace	Before eating dinner, the family held hands to say grace around the table.
-418	elves	In Santa's workshop, the toys are made by the elves on Christmas Eve.
-413	frame	To display the beautiful photo, she ordered an elegant frame made of silver.
486	bloom	We started watering the yard because the plants had begun to bloom out back.
456	wood	At the ancient burial site they found weapons made of wood with smooth edges.
449	narrow	They decided not to go rafting because the river was too narrow and cramped.
446	island	The rescue workers just learned that the captain had died on the island in Bermuda.
437	skin	The factory produces noxious chemicals that can damage your skin quite badly.
437	leather	For her project, she bought lots of black leather and tough fabric.
422	bed	There was a large pile of dirty towels on the bed in the guestroom.
384	quiet	The neighborhood at the top of the tall hill was very quiet and peaceful.
375	attic	The accountant put all of the old documents in the attic at home.
373	chess	They were learning another important rule of chess at Victor's house.

Table S2. Top 10 items from Brothers and Kuperberg (1) with the largest rank decrease in probability from cloze to GPT-2 (top) and top 10 items with the largest rank increase in probability from cloze to GPT-2 (bottom).

cloze probability significantly outperforms GPT-2 surprisal on the naming data. However, picture naming is an offline task that is considerably more similar to a cloze task than it is to word-by-word reading as reflected in SPR and the experiments analyzed in our main study. It is thus perhaps not surprising that cloze provides a better fit to such data, and the relevance of picture naming latencies to online language processing is less direct than SPR or eye-tracking. In light of other findings (including our own, but see also 16, 17) that cloze sometimes provides a poorer fit to human data than recent language models like GPT-2, as well as arguments that there may be important differences in the cognitive mechanisms that underlie the cloze task vs. moment-by-moment language processing (18), the ambivalent results shown in **Table S1** do not convincingly support the notion that cloze is a systematically more reliable proxy (vs. statistical models) for human subjective predictability during real-time language comprehension.

C.3. How Do Cloze-Based and Model-Based Predictability Estimates Differ? Given these divergent results using cloze-based vs. model-based predictability, we now ask whether the two predictability estimates differ in systematic ways. The kernel densities shown in **Figure S1A** shed some light on this question. As shown, the cloze probability/surprisal values for items in these studies tend to fall (by design) into a roughly trimodal distribution corresonding to the high-, moderate-, and low-cloze conditions (the moderate- and low-cloze conditions collapse in probability space to a single large mode in these visualizations), with substantial spread of empirical support across the full spectrum of attested cloze values. Thus, according to the cloze estimates, Brothers and Kuperberg's experimental manipulation successfully varies predictability relatively evenly. The model-based probability/surprisal values are distributed differently: they are strongly concentrated toward low values in probability space and moderate values in surprisal space, with little representation of the extremes of the surprisal continuum.

To build qualitative intuitions about these differences, in **Table S2** we present the top 10 items with the largest rank decrease in probability from cloze to GPT-2 as well as the top 10 items with the largest rank increase in probability from cloze to GPT-2. The rank decrease items (high-cloze items that were assigned low probability by GPT-2) indeed seem like failure cases, i.e., items with a strong next-word candidate that the model should have anticipated but did not, perhaps due to failures of idiomatic knowledge (e.g., "say grace") or world knowledge (e.g., that "proposing" in this context refers to a romantic gesture involving a ring) that humans generally have. To better understand what might be happening in these cases, in **Table S3** we present the top 10 highest probability continuations for each of these items according to GPT-2, with their associated probabilities. As shown, the model assigns implausibly low probability to the high-cloze continuation, and many of the top 10 predictions are nonsensical (e.g., *Bill raked up all the* ***headlines***) or demonstrate misunderstanding (e.g., *To record some high school memories, he had everyone sign his* ***name***, which suggests failure to understand the fact that one does not normally sign someone else's name).

Nevertheless, even in these failure cases, the model shows sensitivity to phenomena that are unlikely to be produced in a cloze task but are plausible in ordinary texts and thus might register in human expectations during reading. These include:

- **Coordination.** E.g.:
  - *The web had been spun by the large* ***and***
  - *To display the beautiful photo, she ordered an elegant* ***,*** (here, the generated comma plausibly delimits coordinated adjectives, suggesting that the model may be entertaining this possibility)

- **Modifiers.** E.g.:
  - *My son saw the bird pecking at the soil using its **own***
  - *In Santa's workshop, the toys are made by the **same***
- **Metaphors.** E.g.,:
  - *The web had been spun by the large **multinational***
- **Effects of extrasentential context.** GPT-2 often receives truncated text in training and must contend with the possibility that relevant context is missing to an extent that human cloze participants may not (since they are aware that they are study participants and the sentences have no context). E.g.:
  - *Before proposing to his girlfriend he would need a **passport*** (e.g., perhaps the partners live in different countries)

These examples illustrate the diversity of linguistic inputs that a general-purpose sentence comprehension system must contend with during typical reading, diversity that models must also contend with in training but that cloze productions may not represent well. In light of this diversity, distributing probability mass across many such continuations, even for "high cloze" experimental items, may often be optimal, both for machines and plausibly human readers. Greater tolerance of diverse outcomes could even underlie the cases of rank *increase* (i.e., prescience) in **Table S2**, where GPT-2 assigns unexpectedly high probability to low-cloze but semantically plausible continuations (although these cases could also of course arise by chance). In any case, the results above suggest that model-based estimates are not only higher resolution than cloze (as discussed in the main article), but also qualitatively different due not only to their weaknesses but also plausibly to their strengths relative to cloze norms as models of human prediction during language comprehension.

D. Discussion. Here we revisited the data and arguments presented in Brothers and Kuperberg (1), the strongest extant evidence favoring linear over logarithmic predictability effects during incremental language comprehension. We showed that both of their key experimental findings are contingent on analysis choices, namely, the use of cloze norms rather than statistical language models to represent predictability. When their experimental data are reanalyzed using statistical language models, results favor logarithmic over linear predictability effects. Thus, even Brothers and Kuperberg's controlled experiment replicates our own claimed logarithmic predictability effect when analyzed using methods more similar to those we have advocated. Although direct support is infeasible due to limitations of the public data release, the same discrepancy might also hold in Brothers and Kuperberg's meta-analysis, which is based exclusively on cloze estimates. We further showed no statistical difference in fit to reading times between cloze probability and GPT-2 surprisal in Brothers and Kuperberg's SPR experiment, which is the experimental task that most closely mimics incremental sentence comprehension (Brothers and Kuperberg's other experiment is picture naming, which is an explicit, offline, cloze-like task that differs in potentially critical ways from incremental sentence comprehension). The ambivalence of this statistical outcome undermines a key pillar supporting Brothers and Kuperberg's interpretation of their data, which turns on the use of cloze. This concern is reinforced by our main finding from the Provo corpus that cloze estimates are neither linear in predictability nor strong psychometrically—they are significantly outperformed as reading models by GPT-2. Other recent studies have reached a similar conclusion (16, 17). Finally, we qualitatively analyzed the items with the strongest discrepancy between cloze and GPT-2 predictability and identified not only clear failure cases in which GPT-2 assigns implausibly low predictability to high-cloze items, but also plausible advantages of model-based estimates in representing the full diversity of continuations in ordinary texts, diversity which is likely also relevant to human expectations during sentence comprehension. In light of these considerations, we do not believe there is strong reason to favor linear cloze-based over logarithmic model-based interpretations of Brothers and Kuperberg's data, and thus we do not consider their study to be strong counterevidence to our claims that predictability effects are more logarithmic than linear.

Although we have argued that the linear predictability effects reported in Brothers and Kuperberg's study are due primarily to analysis choices rather than the use of experimental vs. observational data (and that under different, similarly justifiable analyses our two studies in fact agree), we would additionally like to address their argument (elaborated in their Appendix B) that correlational studies like ours are inherently less reliable than experimental ones like theirs. Their argument (and those they cite in its defense, namely 7, 8) invokes an uncontroversial principle of scientific inference: correlation does not imply causation, and thus well-designed experimental studies (e.g., randomized controlled trials) are strictly more informative about causation than comparable correlational studies, assuming that a pure manipulation is feasible. They demonstrated the practical significance of this issue for the study of predictability effects with an elegant proof-of-concept analysis: they replaced the dependent variable in their SPR experiment (reading time) with mean lexical decision times of the critical words in an independent experiment (19). These lexical decision times can have no causal relation to the trigram predictability of critical words in Brothers and Kuperberg's experiment, since the lexical decision experiment used different stimuli in a different task; nonetheless, they found a significant effect of trigram log probability (over and above unigram log probability, a more straightforwardly causal influence on lexical decision times with which trigram probability covaries) on lexical decision times in a linear model. They attribute this to *residual confounding*, i.e., systematic covariation between a non-causal variable and *measurement error* of a causal variable, leading to a spurious effect. Mitigating such issues can require deep domain knowledge, but even with such knowledge, it is often impossible to completely eliminate the possibility of systematic uncontrolled confounds that could bias inferences. In Brothers and Kuperberg's view, naturalistic studies' systematic tendency to find logarithmic

predictability effects is driven by residual confounding with word frequency, which is known to have a logarithmic effect on response times.

However, there is a less nefarious issue that can produce a similar result: correlation between the non-causal variable and random noise in a specific experimental sample. Although standard tests account for variance in the effect estimate, the fact remains that the degree of overfitting of an arbitrary model to an arbitrary finite dataset cannot be known in advance. Fortunately, this issue can be straightforwardly addressed using out-of-sample testing procedures like those we have used throughout this study. And it turns out that these procedures fix the spurious trigram finding above: when we replace Brothers and Kuperberg's in-sample test with the 5-fold cross-validation procedure used in our analyses of their SPR and naming experiments, unigram log probability remains significant over trigram log probability as a predictor of lexical decision times ($p < 0.001$) but trigram log probability is not significant over unigram log probability ($p = 0.309$). This outcome does not invalidate Brothers and Kuperberg's general concern: we agree that correlation does not imply causation and that confounds can influence results, including in studies of word predictability. Our point is that the practical significance of this concern can be reduced by careful analysis design.

In addition, we see little external support for Brothers and Kuperberg's position that apparent logarithmicity in the predictability effects found by other studies is driven by poor control of frequency effects. Not only do the same logarithmic patterns emerge in their own data when reanalyzed with model-based predictability estimates as shown above (Brothers and Kupergberg's design holds critical words—and thus, frequencies—constant while varying predictability within each item set, leaving no frequency-related variance within a set for GPT-2 surprisal to correlate with), but naturalistic studies regularly include statistical frequency controls with diverse (and increasingly accurate) implementations. For example, our own study estimates frequency from the 3.5-billion word Gigaword 3 corpus (20), and a related study (21) used frequency estimates from the Open Web Text corpus (22), which is about an order of magnitude larger than Gigaword, to directly compare log frequency and GPT-2 surprisal effects, finding additive effects of similar magnitude for both variables across diverse naturalistic datasets (see also 23, for review of convergent findings from experimental studies). Other studies reporting logarithmic effects in English have also used strong frequency controls derived from other text corpora (3, 5), and a recent study showed strongly logarithmic predictability effects in naturalistic reading data from English and 10 other languages under large-scale frequency controls (24). Although Brothers and Kuperberg's claim cannot be directly falsified, we find it implausible that errors in estimating human subjective frequency are so systematically and similarly correlated with surprisal across such diverse training corpora, languages, and statistical language models that they spuriously drive the large and growing body of findings that favor logarithmic over linear predictability effects.

Moreover, it is not obvious that the correlation-causation problem applies uniquely or even especially to naturalistic studies relative to experimental studies for the domain of language research, because a pure manipulation of the theoretically relevant variable (e.g., predictability) is usually not possible. The manipulation usually must be cached out as a manipulation of *language* (e.g., words, morphemes, or syntactic structures), and linguistic units are complex objects with myriad poorly understood relationships not only to diverse and often collinear linguistic variables (frequency, predictability, syntactic category, age-of-acquisition, orthographic neighborhood, lexical semantics, etc.) but also to sensory systems by which words and their referents are perceived, to conceptual systems by which words and sentences interface with knowledge representations, and to executive systems that use these knowledge representations in order to control behavior. A linguistic manipulation therefore plucks at a potentially vast web of interrelated cognitive phenomena. Brothers and Kuperberg's own lexical decision demonstration illustrates the generality of this issue: even controlled studies of lexical decision times must contend with the possibility that the critical variable may covary with errors in any attempts at experimental or statistical control, potentially giving rise to spurious results. These potential confounds must be anticipated and addressed in experimental studies, just as they must be in naturalistic ones. In psycholinguistics, the type of a study (experimental or correlational) may be less informative about its reliability for causal inference than the quality of its experimental and statistical design.

More broadly, Brothers and Kupergberg's discussion of the relative strengths of experimental and naturalistic studies in psycholinguistics may be somewhat selective. Their discussion does not acknowledge known inferential challenges for experimental studies in cognitive science and psycholinguistics that have been treated at length elsewhere (25–31), especially item and task effects. In brief, although an experimental study licenses narrow causal inferences about a specific set of items presented in a specific task (e.g., specific high and low cloze items in a picture naming task), it does not necessarily license broad causal inferences about the theoretical construct of interest (e.g., prediction during incremental sentence processing), in part because of the complexity of language itself (item effects, see the preceding paragraph) and in part because humans adapt flexibly to the experimental setting, and in so doing may draw on novel or heuristic task-specific processing strategies (task effects). In other words, it may matter how closely a given language processing experiment resembles things that people do with language in the real world. People regularly read connected texts for content, but they rarely read dozens of isolated sentences for a comprehension assessment, and the cognitive strategies they use in the latter setting may differ qualitatively from those they use in the former. This concern is reinforced by neuroscientific evidence that domain-general executive regions of the brain, which are largely dormant during passive language comprehension, come online strongly when language comprehension is layered with other tasks (32). Systematic differences with respect to which regions of the brain are engaged between an experiment (a task) and an inferential target (the human language comprehension system) could give rise to systematic differences in measurable responses (e.g., reading behavior). There is thus reason to suspect that experimental studies may be prone to task effects in ways that naturalistic studies plausibly are not (30). This is not to say that experimental studies are uninformative (members of our author team also do experimental work), but rather to establish that experimental studies

of language processing also have inferential limitations that are distinct from but perhaps equally serious to those faced by naturalistic studies.

In other words, experimental and naturalistic studies of human language processing have largely complementary strengths and weaknesses. Experimental studies support strong (narrow) causal inferences but may have poor ecological validity (relevance to language processing in general), whereas naturalistic studies have strong ecological validity but only support causal inferences indirectly and in proportion to the degree to which their statistical design rules out other causal factors. Moreover, each approach is more naturally suited to some questions than others. Many experiments' scientific value lies precisely in their *un*naturalness: deliberately implausible designs can be used to test theoretical predictions about events that rarely or never occur in the real world (33). Experimental studies are well suited to address such questions, and naturalistic studies are not. However, for questions about the normal response of a system to events that regularly occur (e.g., word predictability effects in reading), naturalistic studies can offer substantial advantages in terms of power and ecological validity, and the causality gap with experimental studies can be narrowed using careful statistical design (30). We therefore agree with the spirit of Brothers and Kuperberg's position (citing 7) that "claims made from regression analysis techniques should not be accepted until confirmed via controlled experimental techniques" (p. 9). But we also think that a balanced perspective acknowledges the converse, at least for questions that are amenable to naturalistic investigation. More generally, we think psycholinguistic science would benefit from an emphasis on both establishing consensus around convergent findings and understanding divergent findings across well-designed studies that differ in methodological details, including along the experimental-naturalistic axis. We believe such a consensus is emerging around the functional form of predictability effects on incremental processing demand.

Critical word:	leaves	
Cloze probability:	0.93333	
GPT-2 probability:	0.00002	
Prefix:	Bill raked up all the	
Top-10 GPT-2 predictions:	Token	p
	fuss	0.0373
	money	0.0231
	headlines	0.0208
	talk	0.0131
	right	0.0121
	wrong	0.0093
	hype	0.0093
	way	0.0085
	controversy	0.0082
	media	0.0080

Critical word:	beak	
Cloze probability:	0.98876	
GPT-2 probability:	0.00241	
Prefix:	My son saw the bird pecking at the soil using its	
Top-10 GPT-2 predictions:	Token	p
	tail	0.0543
	claws	0.0389
	wings	0.0365
	tal	0.0279
	nose	0.0277
	long	0.0271
	mouth	0.0234
	own	0.0184
	tiny	0.0178
	eyes	0.0143

Critical word:	spider	
Cloze probability:	0.93333	
GPT-2 probability:	0.00020	
Prefix:	The web had been spun by the large	
Top-10 GPT-2 predictions:	Token	p
	-	0.0686
	media	0.0462
	corporations	0.0352
	multinational	0.0268
	,	0.0238
	and	0.0222
	companies	0.0194
	tech	0.0179
	number	0.0128
	internet	0.0108

Critical word:	yearbook	
Cloze probability:	0.83516	
GPT-2 probability:	0.00014	
Prefix:	To record some high school memories, he had everyone sign his	
Top-10 GPT-2 predictions:	Token	p
	name	0.3214
	own	0.0264
	aut	0.0246
	letter	0.0176
	first	0.0134
	papers	0.0107
	diploma	0.0099
	"	0.0098
	book	0.0077
	daughter	0.0070

Critical word:	ring	
Cloze probability:	0.93333	
GPT-2 probability:	0.00026	
Prefix:	Before proposing to his girlfriend he would need a	
Top-10 GPT-2 predictions:	Token	p
	permit	0.0300
	driver	0.0197
	lawyer	0.0178
	passport	0.0158
	doctor	0.0145
	"	0.0142
	car	0.0121
	job	0.0115
	lot	0.0109
	divorce	0.0105

Critical word:	grace	
Cloze probability:	0.68539	
GPT-2 probability:	0.00004	
Prefix:	Before eating dinner, the family held hands to say	
Top-10 GPT-2 predictions:	Token	p
	goodbye	0.3330
	hello	0.1649
	"	0.0809
	,	0.0710
	thank	0.0512
	farewell	0.0404
	thanks	0.0368
	good	0.0353
	:	0.0146
	a	0.0116

Critical word:	match	
Cloze probability:	1.00000	
GPT-2 probability:	0.00139	
Prefix:	The gentleman lit the candle using a single	
Top-10 GPT-2 predictions:	Token	p
	candle	0.0740
	hand	0.0556
	-	0.0316
	finger	0.0281
	piece	0.0228
	strand	0.0217
	screw	0.0156
	,	0.0148
	brush	0.0137
	stick	0.0132

Critical word:	elves	
Cloze probability:	1.00000	
GPT-2 probability:	0.00326	
Prefix:	In Santa's workshop, the toys are made by the	
Top-10 GPT-2 predictions:	Token	p
	same	0.0689
	kids	0.0187
	children	0.0179
	company	0.0164
	team	0.0159
	Santa	0.0131
	artist	0.0126
	local	0.0122
	crafts	0.0122
	people	0.0101

Critical word:	star	
Cloze probability:	0.96000	
GPT-2 probability:	0.00164	
Prefix:	The hopeful girl wished upon a	
Top-10 GPT-2 predictions:	Token	p
	man	0.0452
	young	0.0186
	stranger	0.0161
	friend	0.0142
	boy	0.0107
	bright	0.0105
	white	0.0092
	god	0.0090
	father	0.0089
	girl	0.0080

Critical word:	frame	
Cloze probability:	0.93333	
GPT-2 probability:	0.00118	
Prefix:	To display the beautiful photo, she ordered an elegant	
Top-10 GPT-2 predictions:	Token	p
	,	0.0438
	dress	0.0297
	black	0.0294
	and	0.0280
	white	0.0267
	set	0.0153
	red	0.0149
	silver	0.0111
	pair	0.0088
	gown	0.0084

Table S3. Top-10 next-token predictions from GPT-2 for Brothers and Kuperberg's (1) items with the largest rank decrease from cloze to GPT-2 probability. GPT-2 uses subword tokenization, which means that candidate outputs include punctuation, white space, control characters, and partial words.

2. Analysis of Word Skipping Behavior

Our study targets the theoretical construct of language *processing difficulty*, for which we (following much prior work) take reading times to be a reliable experimental proxy. Our main analyses therefore focus exclusively on measures of reading time. We have thus far not considered a related phenomenon from the literature on eye-tracking during reading: *word skipping*, when the eyes entirely skip (do not land on) a word during the first pass through a text (34–38).

There are three reasons why word skipping behavior is not included in our main analyses. *First*, skipping words is not possible in self-paced experimental paradigms such as those used for the Brown, Natural Stories SPR, and Natural Stories Maze datasets. Thus, skipping is only relevant to a subset of the data we wish to analyze. *Second*, the predictions of theories of processing difficulty are less straightforward *a priori* for skipping probabilities than they are for reading times. For example, under all theories considered here, predictability effects are thought to derive from the degree of match between predicted and observed words. But what effect might predictability have on the decision to observe (i.e., fixate) a word in the first place? How to extend theories of processing difficulty to such questions is not immediately clear (see below for discussion). *Third*, and perhaps consequently, prior work on the functional form of predictability effects in reading has focused heavily on measures of reading time (1–3, 5), and we have chosen to follow this precedent. Nonetheless, motivated by prior work that found predictability effects on word skipping (e.g., 37, 39–41), here we report supplementary analyses of the effect of predictability on word skipping in our three eye-tracking datasets (the Dundee, GECO, and Provo datasets).

A. Methods. Our analyses of word skipping use the same CDRNN design for each dataset as our analyses of fixation duration, with the following changes:

- Words that were skipped during first-pass reading are added as events to the predictor and response matrices of the CDRNN models (by contrast, our main analyses only consider fixated words).
- The response variable is a boolean indicator for whether a word was skipped during first-pass reading (words that were initially skipped and subsequently fixated during a regressive eye movement are treated as skipped). The response is modeled as binomially distributed rather than exGaussian. The IRF thus describes the expected change in the logit (log-odds) of first-pass skipping as a function of continuous delay in time.
- The *saccade length* predictor (i.e., the length in words of the incoming saccade) is removed from all models. This is because *saccade length* is not well defined for skipped words, which (by virtue of not having been fixated) have no incoming saccade. Although in principle this could be addressed by setting *saccade length* to 0 for skipped words, *saccade length* $= 0$ would then be a near-perfect decision rule for whether a word was skipped (the only other fixations with this value are the first fixation in a text). This decision rule is non-linear but learnable by a neural network (e.g., a CDRNN). Thus, including *saccade length* in the model could trivialize the regression problem by providing a single covariate with near-perfect separation of the target classes (skipped vs. fixated), with potentially harmful downstream consequences for estimates of our effect of interest (predictability).

One challenge for modeling word skipping in our continuous-time deconvolutional framework is the necessity of timestamping skipped words, which are in a sense non-events since they are never fixated. Whereas fixations are naturally timestamped by their onsets (as in our main analyses), what timestamps should be assigned to unfixated words? Here we adopt the approach of assigning the timestamp of the *immediately following* first-pass fixation to all intervening skipped words, since this is the time point at which the skipped words first *could* have been fixated (but in fact were not). This timestamp is then used to determine the delay between responses (fixated or skipped words) and predictors (preceding fixated or skipped words up to and including the current response), which in turn parameterizes the IRF in the CDRNN model. For simplicity, we only consider predictability according to the best-performing language model from the reading time analyses (GPT-2-small) in three functional forms: $f(\text{SURP})$, PROB, and SURP^1.

B. Results. CDRNN-estimated predictability effects on word skipping are visualized in **Figure S2** (middle and bottom rows), along with word length effects for reference (top row) given the well-established tendency for shorter words to be skipped more frequently (35, 37, 42, 43). As shown, CDRNN analyses find the expected decrease in skipping probability for longer words, suggesting that our logistic CDRNN models are sensitive enough to recover a known effect. Estimated predictability effects on word skipping are substantially weaker. As shown in the middle row of **Figure S2**, the Dundee dataset shows no visible relationship between a word's predictability and its probability of being skipped, whereas the GECO and Provo datasets show small estimated increases in skipping rate for less surprising words. The surface plots on the bottom row of **Figure S2** suggest that the peak predictability effect may in fact be delayed in time: although the predictability of a word has a weak association with its own skipping probability (at delay 0s), it has a stronger association with the skipping probability of *subsequent* words within a short time window (delays between 0s and about 0.3s).

Model comparisons on the held-out test set (**Table S4**) support the generalizability of these effects. In 8 of 9 comparisons, models of word skipping that contain a predictability effect ($f(\text{SURP})$, PROB, and SURP^1) significantly outperform those that do not ($\emptyset$); the only exception is the PROB model on the GECO dataset. Thus, our analyses support prior claims that predictability is associated with word skipping (39). However, as indicated by the delayed effects shown in **Figure S2**, the bulk of these effects appear to be effects of the predictability of *prior* words on the skipping probability of the current word, with considerably

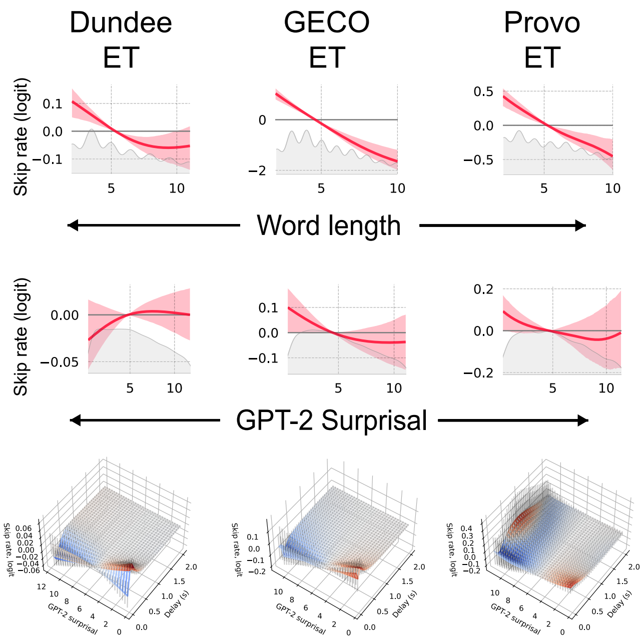

Fig. S2. CDRNN-based analysis of word skipping in eye-tracking datasets (Dundee, GECO, and Provo datasets). **Top:** Functional form of word length (x-axis) effects on the log-odds of skipping (y-axis) with no delay (i.e. at the current word). **Middle:** Functional form of GPT-2 surprisal (x-axis) effects on the log-odds of skipping (y-axis) with no delay (i.e. at the surprising word). **Bottom:** Surface plots showing the estimated change in the log-odds of skipping (vertical axis) from observing a word with a given surprisal value (left axis) at a given delay (in seconds) from word onset (right axis). All plots cover the interdecile range of predictor values in each training dataset. Uncertainty intervals represent 95% variational Bayesian credible intervals. Kernel density plots show the distribution of predictor values in the training data over the plotted range. Word length densities appear to oscillate because word length is integer-valued (number of characters).

	Comparison	Dundee ΔLL	Dundee p	GECO ΔLL	GECO p	Provo ΔLL	Provo p
GPT-2	GPT-2$_{f(\text{SURP})}$ vs. $\emptyset$	**18**	**0.0005**	**49**	**0.0004**	**496**	**0.0002**
	GPT-2$_{\text{PROB}}$ vs. $\emptyset$	**17**	**0.0005**	-7	—	**586**	**0.0002**
	GPT-2$_{\text{SURP}^1}$ vs. $\emptyset$	**12**	**0.0005**	**35**	**0.0004**	**301**	**0.0002**
	GPT-2$_{f(\text{SURP})}$ vs. GPT-2$_{\text{PROB}}$	0	1.0000	**56**	**0.0004**	-90	—
	GPT-2$_{f(\text{SURP})}$ vs. GPT-2$_{\text{SURP}^1}$	6	0.0979	**14**	**0.0247**	**195**	**0.0002**
	GPT-2$_{\text{SURP}^1}$ vs. GPT-2$_{\text{PROB}}$	-5	0.0979	**42**	**0.0004**	**-285**	**0.0002**

Table S4. Testing Results for Word Skipping on Eye-Tracking Datasets. Results of key statistical comparisons between models of word skipping based on permutation tests of the difference in average test set likelihood between the alternative and null CDRNN model in each comparison (ΔLL). Subscripts indicate the assumed functional form of predictability effects: $f(\text{SURP})$ (nonlinear in surprisal), and PROB (linear in probability). Boldface indicates statistical significance. In directional tests where one hypothesis subsumes the other (e.g., $f(\text{SURP})$ vs. SURP^1, since a nonlinear surprisal effect subsumes a linear one), dashes (—) indicate failure of the alternative hypothesis (left) to improve over the null hypothesis (right). Otherwise, cells are color coded using cyan to indicate that the hypothesis on the left outperforms the one on the right, and using magenta to indicate that the hypothesis on the right outperforms the one on the left. All p-values are corrected for false discovery rate (15) across all tests within each dataset.

weaker evidence that a word's predictability affects its own skipping probability. We leave further investigation of this finding to future research.

Results regarding the functional form relating predictability to skipping probability are less clear. The f(SURP) model finds a roughly linear relationship between surprisal and the log-odds of skipping, especially at the peak predictability effect (bottom row of **SI S4**), but the f(SURP) model outperforms the more constrained PROB and SURP^1 models in half of the comparisons, suggesting that the optimal form for the predictability-cost relationship may be other than strictly linear (PROB) or strictly logarithmic (SURP^1). Direct comparisons between the PROB and SURP^1 models are also equivocal: neither model is significant over the other in the Dundee dataset, the SURP^1 model is significant over the PROB model in the GECO dataset, and the PROB model is significant over the SURP^1 model in the Provo dataset. Thus, results do little to clarify the optimal form of the function relating word predictability to the log-odds of skipping, and we leave further investigation of this question to future research.

C. Discussion. Using CDRNN analyses of word skipping in our three eye-tracking datasets, we showed a significant effect of predictability on skipping that is relatively weak on the (un)predictable word itself but stronger on subsequent words that follow closely in time. Statistical comparisons between linear and logarithmic predictability effects were inconclusive, with some evidence that neither of these assumed forms may be optimal for word skipping (since the unconstrained f(SURP) model often outperformed them). What do these word skipping results reveal about our core scientific question: the influence of predictability on processing demand? The answer depends on why there is a relationship between word predictability and word skipping behavior in the first place. Prior theorizing about this relationship has focused on one of two potential causes. The first potential cause is parafoveal preview (39). Words that are not yet in foveal attention are still processed to some extent (44), as revealed e.g., by studies that show effects of preview validity (45–48), by studies that show indirect effects of skipped words (49), and by studies that show effects of the content of words in the parafovea on reading behavior (50, though see 10). Parafoveal preview may provide enough information to partially (dis)confirm predictions, which may in turn allow predictability to influence whether readers skip parafoveally accessed words. Assuming a preview-based mechanism, both the FACILITATION and COST views are consistent with an effect of predictability on skipping probability: when the parafovea provides enough information to determine that a subsequent word is strongly activated by context (FACILITATION view) or contributes little information (COST view), the reader may choose to skip it. With respect to the finer-grained question about the quantitative form of the relationship between predictability and skipping probability, additional theoretical commitments are needed as to precisely how the processing costs of (parafoveally accessed) words affect the skipping decision. In particular, although processing costs are in principle unbounded, skipping probability is constrained to the interval $[0, 1]$ and therefore cannot scale logarithmically on predictability as a matter of mathematical definition. The expected functional form will therefore depend on the choice of nonlinearity (e.g., log, logit) used to map between processing costs and skipping probabilities. This choice in turn depends on principles of elegance or mechanism that must be exposited by theorists. To our knowledge, this is uncharted theoretical territory, and developing such a theory is beyond the scope of our study.

The second potential cause is partial collinearity between predictability and *entropy*, an information-theoretic measure of the degree of spread of the predictive distribution over words given context (38). Lower entropy indexes greater concentration of probability mass on a smaller number of outcomes. Entropy is thus a formalization of the experimental construct of *contextual constraint* and plausibly influences the skipping decision: skipping may be more likely when there is greater certainty (lower entropy) in the distribution over the upcoming word. Entropy in natural texts is positively correlated with surprisal (51); that is, when language models are more certain about the identity of a word (prior to observing it), they tend to be less surprised by the word (after observing it). Thus, an association between predictability and skipping rate could emerge indirectly by correlation with entropy. Indeed, one recent study found no effect of surprisal on skipping rates once entropy was taken into account (52). Assuming an entropy-based mechanism (i.e., disregarding parafoveal preview), neither the FACILITATION nor the COST views predicts an entropy-independent predictability effect: without access to the identity of the predicted word, the degree of prediction (mis)match cannot be computed, nor used to guide the skipping decision.

In summary, word skipping behavior does not to our knowledge differentiate the classes of theories at issue in our study. Both the FACILITATION and COST views make broadly similar predictions about skipping behavior (predictability effects on skipping under a preview-based mechanism, no unique predictability effects on skipping under an entropy-based mechanism) without clear differences in their commitments as to the functional form relating predictability to skipping probability (under a preview-based mechanisms). Our findings support the existence of a predictability effect on skipping probability but locate this predictability effect primarily on the skipping rate of *subsequent words*, an outcome that does not appear to be anticipated by either theory. Thus, these word skipping analyses are largely orthogonal to our core claims about predictability and processing demand. They primarily serve to explore a dimension (skipping rate) of the predictability-cost relationship which is under-studied in naturalistic settings, and which warrants further theoretical analysis.

3. Visualization of Word Length and Frequency Effects

Although the primary construct of interest in our study is word predictability, reading times are also known to be influenced by other factors, especially word length (53) and frequency (54). For this reason, our own statistical models include length and unigram surprisal (negative normalized log frequency) as control covariates (see **SI 10**). To establish continuity with prior work, in this section we visualize the estimates for these length and frequency predictors in models that simultaneously estimate effects of GPT-2 surprisal (along with other control predictors). Results are shown in **Figure S3**. As expected, reading times increase across datasets for both longer words and less frequent words (higher unigram surprisal). These effects are similar in

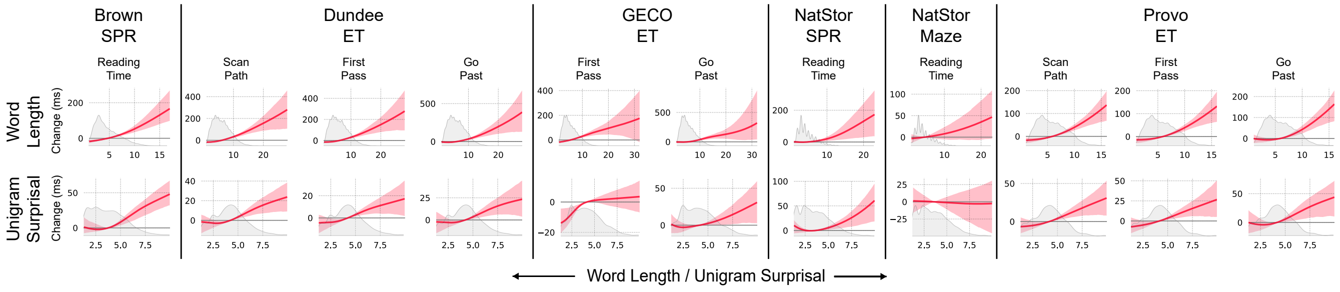

Fig. S3. CDRNN-estimated functional form of word length and unigram surprisal (negative log frequency) effects with no delay (i.e. at the current word). Plots cover the full empirical range of predictor values in each training dataset. Kernel density plots show the distribution of predictor values in the training data over the plotted range.

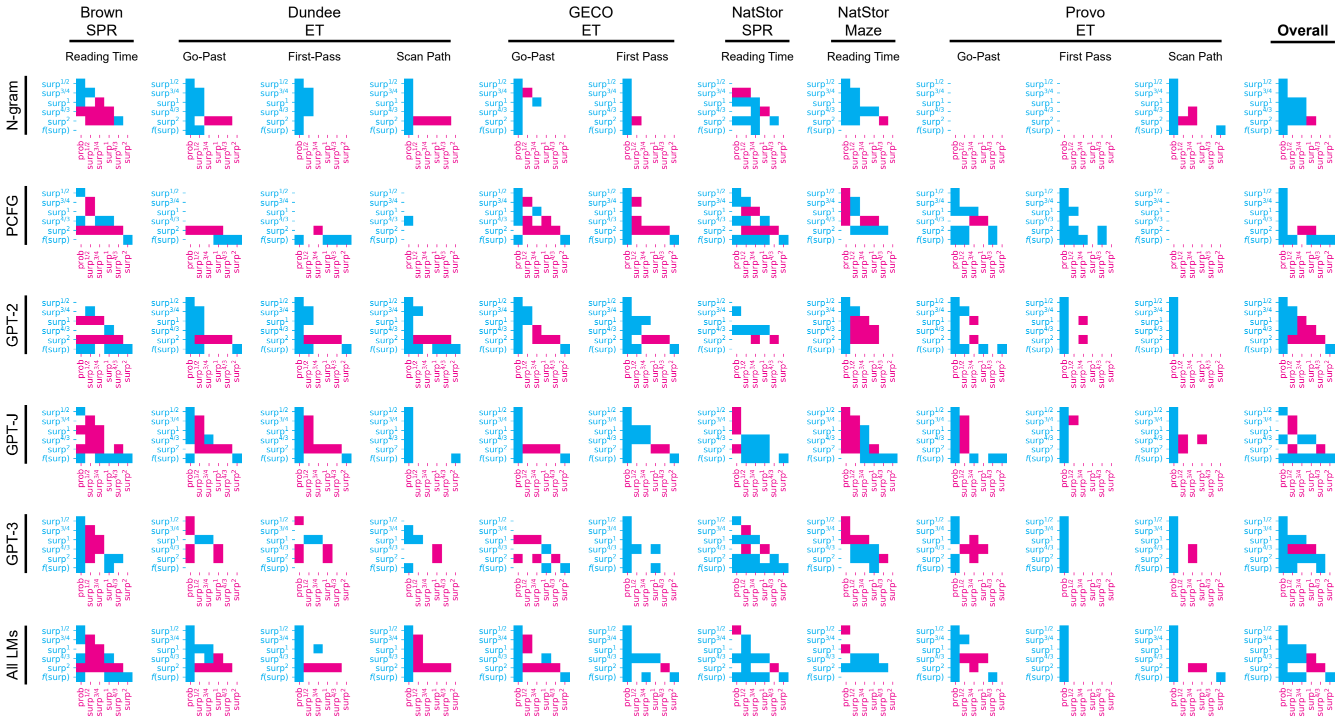

Fig. S4. Performance comparison across datasets and language models between pairs of assumed forms for the effect of word predictability. For a given pair, cyan indicates that the model on the row has significantly better test set performance than the model on the column, magenta indicates that the model on the column significantly outperforms the model on the row, and white indicates no significant difference. Only the lower triangle is shown. Note that comparisons to the unconstrained $f(\textsc{surp})$ model are treated as directional (since $f(\textsc{surp})$ subsumes the hypothesis space of all other models), and thus comparisons in which another model numerically outperforms $f(\textsc{surp})$ on the test set are treated as non-significant. Tests use false discovery rate correction for multiple comparisons (15) within the set of comparisons covered by each cell of the figure. Overall results across language models and datasets (lower right subplot) indicate that PROB significantly under-performs surprisal-based models, that $\textsc{surp}^1$ significantly outperforms superlogarithmic models ($\textsc{surp}^{3/4}$ and $\textsc{surp}^2$), and that $\textsc{surp}^1$ is the best performing constrained model overall.

size to those obtained for our critical surprisal measures (**Figure S9**). Thus, our CDRNN-based analyses recover other known signatures of processing difficulty, beyond predictability effects.

Word length, frequency, and predictability covary in language, and the relationship (and degree of separability) between these variables is of central theoretical concern; for example, the degree to which frequency and predictability effects are dissociable has been tied to theoretical debates about the nature of sentence comprehension that are closely related to the FACILITATION and COST views targeted by our study (23). Full investigation of these relationships is beyond the scope of this work, but a recent study used similar methods (CDRNN analyses of naturalistic reading) to investigate the relationship between frequency and predictability effects and found evidence supporting an additive dissociation between frequency and predictability in naturalistic reading (21), as has been claimed based on experimental designs (23, 55). This outcome suggests bounds on the role of probabilistic inference in sentence interpretation by pointing to the existence of inference-independent lexical retrieval difficulty (23), although inference-based explanations for this finding may exist (see 21).

4. Full Significance Testing Results

Tables S5–S9 provide the full results of all statistical tests conducted in this study. **Figure S4** visualizes the results of key comparisons between hypothesized forms for the predictability-cost relationship, broken out by individual models and datasets. The bottom right subplot is identical to **Figure 4** of the main article, which shows results of tests that aggregate across all datasets and language models.

Group	Comparison	Brown (SPR) ΔLL	Brown (SPR) p	NatStor (SPR) ΔLL	NatStor (SPR) p	NatStor (Maze) ΔLL	NatStor (Maze) p
n-gram	n-gram$_{f(\text{SURP})}$ vs. $\emptyset$	-20	—	43	1.0000	15	1.0000
	n-gram$_{f(\text{SURP})}$ vs. n-gram$_{\text{PROB}}$	-11	—	40	1.0000	-134	—
	n-gram$_{f(\text{SURP})}$ vs. n-gram$_{\text{SURP}^{1/2}}$	-98	—	107	0.2520	-192	—
	n-gram$_{f(\text{SURP})}$ vs. n-gram$_{\text{SURP}^{3/4}}$	-137	—	**504**	**0.0006**	-300	—
	n-gram$_{f(\text{SURP})}$ vs. n-gram$_{\text{SURP}^{1}}$	-82	—	-230	—	-334	—
	n-gram$_{f(\text{SURP})}$ vs. n-gram$_{\text{SURP}^{4/3}}$	20	0.3784	-19	—	-375	—
	n-gram$_{f(\text{SURP})}$ vs. n-gram$_{\text{SURP}^{2}}$	-39	—	-293	—	-326	—
	n-gram$_{\text{PROB}}$ vs. $\emptyset$	-9	—	3	1.0000	**149**	**0.0005**
	n-gram$_{\text{SURP}^{1/2}}$ vs. $\emptyset$	**78**	**0.0006**	-65	—	**207**	**0.0005**
	n-gram$_{\text{SURP}^{3/4}}$ vs. $\emptyset$	**116**	**0.0006**	-461	—	**315**	**0.0005**
	n-gram$_{\text{SURP}^{1}}$ vs. $\emptyset$	**62**	**0.0006**	**273**	**0.0006**	**349**	**0.0005**
	n-gram$_{\text{SURP}^{4/3}}$ vs. $\emptyset$	-40	—	62	1.0000	**390**	**0.0005**
	n-gram$_{\text{SURP}^{2}}$ vs. $\emptyset$	18	0.4223	**336**	**0.0006**	**341**	**0.0005**
	n-gram$_{\text{SURP}^{1/2}}$ vs. n-gram$_{\text{PROB}}$	**87**	**0.0006**	-67	1.0000	**58**	**0.0073**
	n-gram$_{\text{SURP}^{3/4}}$ vs. n-gram$_{\text{PROB}}$	**125**	**0.0006**	**-464**	**0.0006**	**166**	**0.0005**
	n-gram$_{\text{SURP}^{1}}$ vs. n-gram$_{\text{PROB}}$	**71**	**0.0006**	**270**	**0.0006**	**200**	**0.0005**
	n-gram$_{\text{SURP}^{4/3}}$ vs. n-gram$_{\text{PROB}}$	**-31**	**0.0234**	59	1.0000	**241**	**0.0005**
	n-gram$_{\text{SURP}^{2}}$ vs. n-gram$_{\text{PROB}}$	27	0.0669	**333**	**0.0006**	**192**	**0.0005**
	n-gram$_{\text{SURP}^{3/4}}$ vs. n-gram$_{\text{SURP}^{1/2}}$	**38**	**0.0049**	**-397**	**0.0006**	**109**	**0.0005**
	n-gram$_{\text{SURP}^{1}}$ vs. n-gram$_{\text{SURP}^{1/2}}$	-16	0.6168	**338**	**0.0006**	**142**	**0.0005**
	n-gram$_{\text{SURP}^{4/3}}$ vs. n-gram$_{\text{SURP}^{1/2}}$	**-118**	**0.0006**	127	0.1453	**183**	**0.0005**
	n-gram$_{\text{SURP}^{2}}$ vs. n-gram$_{\text{SURP}^{1/2}}$	**-59**	**0.0006**	**400**	**0.0006**	**134**	**0.0005**
	n-gram$_{\text{SURP}^{1}}$ vs. n-gram$_{\text{SURP}^{3/4}}$	**-55**	**0.0006**	**734**	**0.0006**	33	0.3215
	n-gram$_{\text{SURP}^{4/3}}$ vs. n-gram$_{\text{SURP}^{3/4}}$	**-157**	**0.0006**	**524**	**0.0006**	**75**	**0.0005**
	n-gram$_{\text{SURP}^{2}}$ vs. n-gram$_{\text{SURP}^{3/4}}$	**-98**	**0.0006**	**797**	**0.0006**	26	0.9934
	n-gram$_{\text{SURP}^{4/3}}$ vs. n-gram$_{\text{SURP}^{1}}$	**-102**	**0.0006**	**-211**	**0.0006**	**41**	**0.0250**
	n-gram$_{\text{SURP}^{2}}$ vs. n-gram$_{\text{SURP}^{1}}$	**-43**	**0.0006**	62	1.0000	-8	1.0000
	n-gram$_{\text{SURP}^{2}}$ vs. n-gram$_{\text{SURP}^{4/3}}$	**59**	**0.0006**	**273**	**0.0006**	**-49**	**0.0399**
	n-gram$_{\text{SURP}^{<1}}$ vs. n-gram$_{\text{SURP}^{1}}$	**37**	**0.0012**	**-545**	**0.0006**	**-94**	**0.0005**
	n-gram$_{\text{SURP}^{>1}}$ vs. n-gram$_{\text{SURP}^{1}}$	**-69**	**0.0006**	-88	0.3359	**41**	**0.0303**
	n-gram$_{\text{SURP}^{<1}}$ vs. n-gram$_{\text{SURP}^{\geq 1}}$	**73**	**0.0006**	**-464**	**0.0006**	**-113**	**0.0005**
	n-gram$_{\text{SURP}^{>1}}$ vs. n-gram$_{\text{SURP}^{\leq 1}}$	**-85**	**0.0006**	**182**	**0.0006**	**81**	**0.0005**
PCFG	PCFG$_{f(\text{SURP})}$ vs. $\emptyset$	**81**	**0.0006**	**408**	**0.0006**	-107	—
	PCFG$_{f(\text{SURP})}$ vs. PCFG$_{\text{PROB}}$	12	1.0000	**926**	**0.0006**	-457	—
	PCFG$_{f(\text{SURP})}$ vs. PCFG$_{\text{SURP}^{1/2}}$	-37	—	**327**	**0.0006**	-78	—
	PCFG$_{f(\text{SURP})}$ vs. PCFG$_{\text{SURP}^{3/4}}$	25	0.1315	**182**	**0.0026**	-268	—
	PCFG$_{f(\text{SURP})}$ vs. PCFG$_{\text{SURP}^{1}}$	27	0.0869	**807**	**0.0006**	-259	—
	PCFG$_{f(\text{SURP})}$ vs. PCFG$_{\text{SURP}^{4/3}}$	-25	—	123	0.1657	-107	—
	PCFG$_{f(\text{SURP})}$ vs. PCFG$_{\text{SURP}^{2}}$	**80**	**0.0006**	**979**	**0.0006**	-439	—
	PCFG$_{\text{PROB}}$ vs. $\emptyset$	**69**	**0.0006**	-518	—	**350**	**0.0005**
	PCFG$_{\text{SURP}^{1/2}}$ vs. $\emptyset$	**118**	**0.0006**	81	0.7112	-29	—
	PCFG$_{\text{SURP}^{3/4}}$ vs. $\emptyset$	**55**	**0.0006**	**226**	**0.0011**	**161**	**0.0005**
	PCFG$_{\text{SURP}^{1}}$ vs. $\emptyset$	**54**	**0.0006**	-399	—	**152**	**0.0005**
	PCFG$_{\text{SURP}^{4/3}}$ vs. $\emptyset$	**106**	**0.0006**	**285**	**0.0006**	0	1.0000
	PCFG$_{\text{SURP}^{2}}$ vs. $\emptyset$	0	1.0000	-571	—	**332**	**0.0005**
	PCFG$_{\text{SURP}^{1/2}}$ vs. PCFG$_{\text{PROB}}$	**49**	**0.0006**	**600**	**0.0006**	**-380**	**0.0005**
	PCFG$_{\text{SURP}^{3/4}}$ vs. PCFG$_{\text{PROB}}$	-13	1.0000	**744**	**0.0006**	**-189**	**0.0005**
	PCFG$_{\text{SURP}^{1}}$ vs. PCFG$_{\text{PROB}}$	-14	1.0000	120	0.1271	**-199**	**0.0005**
	PCFG$_{\text{SURP}^{4/3}}$ vs. PCFG$_{\text{PROB}}$	**37**	**0.0094**	**804**	**0.0006**	**-350**	**0.0005**
	PCFG$_{\text{SURP}^{2}}$ vs. PCFG$_{\text{PROB}}$	**-68**	**0.0006**	-53	1.0000	-18	0.6045
	PCFG$_{\text{SURP}^{3/4}}$ vs. PCFG$_{\text{SURP}^{1/2}}$	**-63**	**0.0006**	**144**	**0.0195**	**190**	**0.0005**
	PCFG$_{\text{SURP}^{1}}$ vs. PCFG$_{\text{SURP}^{1/2}}$	**-64**	**0.0006**	**-480**	**0.0006**	**181**	**0.0005**
	PCFG$_{\text{SURP}^{4/3}}$ vs. PCFG$_{\text{SURP}^{1/2}}$	-13	1.0000	**204**	**0.0011**	29	0.5347
	PCFG$_{\text{SURP}^{2}}$ vs. PCFG$_{\text{SURP}^{1/2}}$	**-118**	**0.0006**	**-653**	**0.0006**	**362**	**0.0005**
	PCFG$_{\text{SURP}^{1}}$ vs. PCFG$_{\text{SURP}^{3/4}}$	-1	1.0000	**-624**	**0.0006**	-9	1.0000
	PCFG$_{\text{SURP}^{4/3}}$ vs. PCFG$_{\text{SURP}^{3/4}}$	**50**	**0.0006**	60	1.0000	**-161**	**0.0005**
	PCFG$_{\text{SURP}^{2}}$ vs. PCFG$_{\text{SURP}^{3/4}}$	**-55**	**0.0006**	**-797**	**0.0006**	**172**	**0.0005**
	PCFG$_{\text{SURP}^{4/3}}$ vs. PCFG$_{\text{SURP}^{1}}$	**51**	**0.0006**	**684**	**0.0006**	**-152**	**0.0005**
	PCFG$_{\text{SURP}^{2}}$ vs. PCFG$_{\text{SURP}^{1}}$	**-54**	**0.0006**	**-172**	**0.0274**	**181**	**0.0005**
	PCFG$_{\text{SURP}^{2}}$ vs. PCFG$_{\text{SURP}^{4/3}}$	**-105**	**0.0006**	**-857**	**0.0006**	**332**	**0.0005**
	PCFG$_{\text{SURP}^{<1}}$ vs. PCFG$_{\text{SURP}^{1}}$	**54**	**0.0006**	**613**	**0.0006**	**-105**	**0.0005**
	PCFG$_{\text{SURP}^{>1}}$ vs. PCFG$_{\text{SURP}^{1}}$	-3	1.0000	**258**	**0.0006**	33	0.0524
	PCFG$_{\text{SURP}^{<1}}$ vs. PCFG$_{\text{SURP}^{\geq 1}}$	**54**	**0.0006**	**536**	**0.0006**	**-105**	**0.0005**
	PCFG$_{\text{SURP}^{>1}}$ vs. PCFG$_{\text{SURP}^{\leq 1}}$	**-41**	**0.0006**	**-153**	**0.0011**	**96**	**0.0005**
GPT-2	GPT-2$_{f(\text{SURP})}$ vs. $\emptyset$	**120**	**0.0006**	87	0.7904	**179**	**0.0004**
	GPT-2$_{f(\text{SURP})}$ vs. GPT-2$_{\text{PROB}}$	**53**	**0.0006**	53	1.0000	**75**	**0.0004**
	GPT-2$_{f(\text{SURP})}$ vs. GPT-2$_{\text{SURP}^{1/2}}$	**54**	**0.0006**	-70	—	-228	—
	GPT-2$_{f(\text{SURP})}$ vs. GPT-2$_{\text{SURP}^{3/4}}$	22	0.2528	-160	—	-276	—
	GPT-2$_{f(\text{SURP})}$ vs. GPT-2$_{\text{SURP}^{1}}$	**101**	**0.0006**	-101	—	-173	—
	GPT-2$_{f(\text{SURP})}$ vs. GPT-2$_{\text{SURP}^{4/3}}$	**39**	**0.0018**	-351	—	-92	—
	GPT-2$_{f(\text{SURP})}$ vs. GPT-2$_{\text{SURP}^{2}}$	**157**	**0.0006**	-8	—	-104	—
	GPT-2$_{\text{PROB}}$ vs. $\emptyset$	**67**	**0.0006**	34	1.0000	**104**	**0.0004**
	GPT-2$_{\text{SURP}^{1/2}}$ vs. $\emptyset$	**66**	**0.0006**	157	0.0510	**407**	**0.0004**
	GPT-2$_{\text{SURP}^{3/4}}$ vs. $\emptyset$	**98**	**0.0006**	**247**	**0.0013**	**456**	**0.0004**
	GPT-2$_{\text{SURP}^{1}}$ vs. $\emptyset$	19	0.4443	**188**	**0.0154**	**352**	**0.0004**
	GPT-2$_{\text{SURP}^{4/3}}$ vs. $\emptyset$	**81**	**0.0006**	**439**	**0.0013**	**272**	**0.0004**
	GPT-2$_{\text{SURP}^{2}}$ vs. $\emptyset$	-37	—	95	0.5895	**283**	**0.0004**
	GPT-2$_{\text{SURP}^{1/2}}$ vs. GPT-2$_{\text{PROB}}$	-1	1.0000	123	0.1498	**303**	**0.0004**
	GPT-2$_{\text{SURP}^{3/4}}$ vs. GPT-2$_{\text{PROB}}$	31	0.0732	**213**	**0.0049**	**352**	**0.0004**
	GPT-2$_{\text{SURP}^{1}}$ vs. GPT-2$_{\text{PROB}}$	**-48**	**0.0012**	154	0.0510	**248**	**0.0004**
	GPT-2$_{\text{SURP}^{4/3}}$ vs. GPT-2$_{\text{PROB}}$	14	1.0000	**405**	**0.0013**	**168**	**0.0004**
	GPT-2$_{\text{SURP}^{2}}$ vs. GPT-2$_{\text{PROB}}$	**-104**	**0.0006**	61	1.0000	**179**	**0.0004**
	GPT-2$_{\text{SURP}^{3/4}}$ vs. GPT-2$_{\text{SURP}^{1/2}}$	**32**	**0.0270**	90	0.5832	**48**	**0.0017**
	GPT-2$_{\text{SURP}^{1}}$ vs. GPT-2$_{\text{SURP}^{1/2}}$	**-47**	**0.0006**	30	1.0000	**-55**	**0.0021**
	GPT-2$_{\text{SURP}^{4/3}}$ vs. GPT-2$_{\text{SURP}^{1/2}}$	15	0.8131	**281**	**0.0013**	**-136**	**0.0004**
	GPT-2$_{\text{SURP}^{2}}$ vs. GPT-2$_{\text{SURP}^{1/2}}$	**-103**	**0.0006**	-63	1.0000	**-124**	**0.0004**
	GPT-2$_{\text{SURP}^{1}}$ vs. GPT-2$_{\text{SURP}^{3/4}}$	**-79**	**0.0006**	-59	1.0000	**-104**	**0.0004**
	GPT-2$_{\text{SURP}^{4/3}}$ vs. GPT-2$_{\text{SURP}^{3/4}}$	-17	0.5974	**191**	**0.0079**	**-184**	**0.0004**
	GPT-2$_{\text{SURP}^{2}}$ vs. GPT-2$_{\text{SURP}^{3/4}}$	**-135**	**0.0006**	**-152**	**0.0369**	**-172**	**0.0004**
	GPT-2$_{\text{SURP}^{4/3}}$ vs. GPT-2$_{\text{SURP}^{1}}$	**62**	**0.0006**	**251**	**0.0013**	**-80**	**0.0004**
	GPT-2$_{\text{SURP}^{2}}$ vs. GPT-2$_{\text{SURP}^{1}}$	**-56**	**0.0006**	-93	0.4761	**-68**	**0.0004**
	GPT-2$_{\text{SURP}^{2}}$ vs. GPT-2$_{\text{SURP}^{4/3}}$	**-118**	**0.0006**	**-344**	**0.0013**	12	1.0000
	GPT-2$_{\text{SURP}^{<1}}$ vs. GPT-2$_{\text{SURP}^{1}}$	**54**	**0.0006**	-26	1.0000	**63**	**0.0004**
	GPT-2$_{\text{SURP}^{>1}}$ vs. GPT-2$_{\text{SURP}^{1}}$	-12	0.9340	**149**	**0.0145**	**-72**	**0.0004**
	GPT-2$_{\text{SURP}^{<1}}$ vs. GPT-2$_{\text{SURP}^{\geq 1}}$	**66**	**0.0006**	**-140**	**0.0013**	**112**	**0.0004**
	GPT-2$_{\text{SURP}^{>1}}$ vs. GPT-2$_{\text{SURP}^{\leq 1}}$	**-50**	**0.0006**	**175**	**0.0013**	**-125**	**0.0004**

Group	Comparison	Brown (SPR) ΔLL	Brown (SPR) p	NatStor (SPR) ΔLL	NatStor (SPR) p	NatStor (Maze) ΔLL	NatStor (Maze) p
GPT-J	GPT-J$_{f(\text{SURP})}$ vs. $\emptyset$	**136**	**0.0006**	**326**	**0.0009**	**276**	**0.0006**
	GPT-J$_{f(\text{SURP})}$ vs. GPT-J$_{\text{PROB}}$	**89**	**0.0006**	-6	—	-176	—
	GPT-J$_{f(\text{SURP})}$ vs. GPT-J$_{\text{SURP}^{1/2}}$	-67	—	**425**	**0.0009**	-27	—
	GPT-J$_{f(\text{SURP})}$ vs. GPT-J$_{\text{SURP}^{3/4}}$	**79**	**0.0006**	**334**	**0.0009**	**259**	**0.0006**
	GPT-J$_{f(\text{SURP})}$ vs. GPT-J$_{\text{SURP}^{1}}$	**135**	**0.0006**	**358**	**0.0009**	**104**	**0.0006**
	GPT-J$_{f(\text{SURP})}$ vs. GPT-J$_{\text{SURP}^{4/3}}$	**114**	**0.0006**	17	1.0000	**153**	**0.0006**
	GPT-J$_{f(\text{SURP})}$ vs. GPT-J$_{\text{SURP}^{2}}$	**161**	**0.0006**	**157**	**0.0352**	**162**	**0.0006**
	GPT-J$_{\text{PROB}}$ vs. $\emptyset$	**47**	**0.0006**	**332**	**0.0009**	**452**	**0.0006**
	GPT-J$_{\text{SURP}^{1/2}}$ vs. $\emptyset$	**203**	**0.0006**	-99	—	**303**	**0.0006**
	GPT-J$_{\text{SURP}^{3/4}}$ vs. $\emptyset$	**57**	**0.0006**	-8	—	17	1.0000
	GPT-J$_{\text{SURP}^{1}}$ vs. $\emptyset$	2	1.0000	-32	—	**172**	**0.0006**
	GPT-J$_{\text{SURP}^{4/3}}$ vs. $\emptyset$	22	0.2740	**309**	**0.0009**	**123**	**0.0006**
	GPT-J$_{\text{SURP}^{2}}$ vs. $\emptyset$	-25	—	**169**	**0.0351**	**114**	**0.0006**
	GPT-J$_{\text{SURP}^{1/2}}$ vs. GPT-J$_{\text{PROB}}$	**156**	**0.0006**	**-431**	**0.0009**	**-149**	**0.0006**
	GPT-J$_{\text{SURP}^{3/4}}$ vs. GPT-J$_{\text{PROB}}$	10	1.0000	**-340**	**0.0009**	**-435**	**0.0006**
	GPT-J$_{\text{SURP}^{1}}$ vs. GPT-J$_{\text{PROB}}$	**-46**	**0.0006**	**-364**	**0.0009**	**-280**	**0.0006**
	GPT-J$_{\text{SURP}^{4/3}}$ vs. GPT-J$_{\text{PROB}}$	-25	0.1852	-23	1.0000	**-329**	**0.0006**
	GPT-J$_{\text{SURP}^{2}}$ vs. GPT-J$_{\text{PROB}}$	**-72**	**0.0006**	**-163**	**0.0246**	**-338**	**0.0006**
	GPT-J$_{\text{SURP}^{3/4}}$ vs. GPT-J$_{\text{SURP}^{1/2}}$	**-146**	**0.0006**	91	0.8235	**-286**	**0.0006**
	GPT-J$_{\text{SURP}^{1}}$ vs. GPT-J$_{\text{SURP}^{1/2}}$	**-201**	**0.0006**	67	1.0000	**-131**	**0.0006**
	GPT-J$_{\text{SURP}^{4/3}}$ vs. GPT-J$_{\text{SURP}^{1/2}}$	**-181**	**0.0006**	**408**	**0.0009**	**-180**	**0.0006**
	GPT-J$_{\text{SURP}^{2}}$ vs. GPT-J$_{\text{SURP}^{1/2}}$	**-228**	**0.0006**	**269**	**0.0009**	**-189**	**0.0006**
	GPT-J$_{\text{SURP}^{1}}$ vs. GPT-J$_{\text{SURP}^{3/4}}$	**-55**	**0.0006**	-24	1.0000	**155**	**0.0006**
	GPT-J$_{\text{SURP}^{4/3}}$ vs. GPT-J$_{\text{SURP}^{3/4}}$	**-35**	**0.0114**	**317**	**0.0009**	**106**	**0.0006**
	GPT-J$_{\text{SURP}^{2}}$ vs. GPT-J$_{\text{SURP}^{3/4}}$	**-82**	**0.0006**	**177**	**0.0144**	**97**	**0.0006**
	GPT-J$_{\text{SURP}^{4/3}}$ vs. GPT-J$_{\text{SURP}^{1}}$	20	0.1952	**341**	**0.0009**	-49	0.0678
	GPT-J$_{\text{SURP}^{2}}$ vs. GPT-J$_{\text{SURP}^{1}}$	-26	0.0748	**201**	**0.0038**	**-58**	**0.0022**
	GPT-J$_{\text{SURP}^{2}}$ vs. GPT-J$_{\text{SURP}^{4/3}}$	**-47**	**0.0006**	-140	0.0767	-9	1.0000
	GPT-J$_{\text{SURP}^{<1}}$ vs. GPT-J$_{\text{SURP}^{1}}$	**139**	**0.0006**	-67	1.0000	**-51**	**0.0083**
	GPT-J$_{\text{SURP}^{>1}}$ vs. GPT-J$_{\text{SURP}^{1}}$	-20	0.1593	**300**	**0.0009**	**-49**	**0.0095**
	GPT-J$_{\text{SURP}^{<1}}$ vs. GPT-J$_{\text{SURP}^{\geq 1}}$	**157**	**0.0006**	**-140**	**0.0038**	-2	1.0000
	GPT-J$_{\text{SURP}^{>1}}$ vs. GPT-J$_{\text{SURP}^{\leq 1}}$	**-78**	**0.0006**	**345**	**0.0009**	2	1.0000
GPT-3	GPT-3$_{f(\text{SURP})}$ vs. $\emptyset$	**119**	**0.0006**	**535**	**0.0006**	**241**	**0.0005**
	GPT-3$_{f(\text{SURP})}$ vs. GPT-3$_{\text{PROB}}$	**84**	**0.0006**	**686**	**0.0006**	-91	—
	GPT-3$_{f(\text{SURP})}$ vs. GPT-3$_{\text{SURP}^{1/2}}$	-93	—	**461**	**0.0006**	9	1.0000
	GPT-3$_{f(\text{SURP})}$ vs. GPT-3$_{\text{SURP}^{3/4}}$	-8	—	**759**	**0.0006**	-10	—
	GPT-3$_{f(\text{SURP})}$ vs. GPT-3$_{\text{SURP}^{1}}$	**33**	**0.0076**	**221**	**0.0012**	**135**	**0.0005**
	GPT-3$_{f(\text{SURP})}$ vs. GPT-3$_{\text{SURP}^{4/3}}$	23	0.1402	**701**	**0.0006**	-108	—
	GPT-3$_{f(\text{SURP})}$ vs. GPT-3$_{\text{SURP}^{2}}$	-32	—	**173**	**0.0171**	-76	—
	GPT-3$_{\text{PROB}}$ vs. $\emptyset$	**36**	**0.0125**	-152	—	**332**	**0.0005**
	GPT-3$_{\text{SURP}^{1/2}}$ vs. $\emptyset$	**212**	**0.0006**	74	1.0000	**232**	**0.0005**
	GPT-3$_{\text{SURP}^{3/4}}$ vs. $\emptyset$	**128**	**0.0006**	-224	—	**251**	**0.0005**
	GPT-3$_{\text{SURP}^{1}}$ vs. $\emptyset$	**86**	**0.0006**	**314**	**0.0006**	**106**	**0.0005**
	GPT-3$_{\text{SURP}^{4/3}}$ vs. $\emptyset$	**96**	**0.0006**	-166	—	**349**	**0.0005**
	GPT-3$_{\text{SURP}^{2}}$ vs. $\emptyset$	**152**	**0.0006**	**361**	**0.0006**	**317**	**0.0005**
	GPT-3$_{\text{SURP}^{1/2}}$ vs. GPT-3$_{\text{PROB}}$	**177**	**0.0006**	**226**	**0.0006**	**-100**	**0.0005**
	GPT-3$_{\text{SURP}^{3/4}}$ vs. GPT-3$_{\text{PROB}}$	**92**	**0.0006**	-73	1.0000	**-81**	**0.0005**
	GPT-3$_{\text{SURP}^{1}}$ vs. GPT-3$_{\text{PROB}}$	**51**	**0.0006**	**466**	**0.0006**	**-226**	**0.0005**
	GPT-3$_{\text{SURP}^{4/3}}$ vs. GPT-3$_{\text{PROB}}$	**60**	**0.0006**	-14	1.0000	17	0.9090
	GPT-3$_{\text{SURP}^{2}}$ vs. GPT-3$_{\text{PROB}}$	**116**	**0.0006**	**513**	**0.0006**	-15	1.0000
	GPT-3$_{\text{SURP}^{3/4}}$ vs. GPT-3$_{\text{SURP}^{1/2}}$	**-85**	**0.0006**	**-298**	**0.0006**	19	0.9090
	GPT-3$_{\text{SURP}^{1}}$ vs. GPT-3$_{\text{SURP}^{1/2}}$	**-126**	**0.0006**	**240**	**0.0006**	**-126**	**0.0005**
	GPT-3$_{\text{SURP}^{4/3}}$ vs. GPT-3$_{\text{SURP}^{1/2}}$	**-116**	**0.0006**	**-240**	**0.0006**	**117**	**0.0005**
	GPT-3$_{\text{SURP}^{2}}$ vs. GPT-3$_{\text{SURP}^{1/2}}$	**-61**	**0.0006**	**288**	**0.0006**	**85**	**0.0005**
	GPT-3$_{\text{SURP}^{1}}$ vs. GPT-3$_{\text{SURP}^{3/4}}$	**-42**	**0.0006**	**538**	**0.0006**	**-145**	**0.0005**
	GPT-3$_{\text{SURP}^{4/3}}$ vs. GPT-3$_{\text{SURP}^{3/4}}$	**-32**	**0.0144**	58	1.0000	**98**	**0.0005**
	GPT-3$_{\text{SURP}^{2}}$ vs. GPT-3$_{\text{SURP}^{3/4}}$	24	0.1169	**586**	**0.0006**	**66**	**0.0005**
	GPT-3$_{\text{SURP}^{4/3}}$ vs. GPT-3$_{\text{SURP}^{1}}$	10	1.0000	**-480**	**0.0006**	**243**	**0.0005**
	GPT-3$_{\text{SURP}^{2}}$ vs. GPT-3$_{\text{SURP}^{1}}$	**66**	**0.0006**	47	1.0000	**211**	**0.0005**
	GPT-3$_{\text{SURP}^{2}}$ vs. GPT-3$_{\text{SURP}^{4/3}}$	**56**	**0.0006**	**527**	**0.0006**	**-32**	**0.0298**
	GPT-3$_{\text{SURP}^{<1}}$ vs. GPT-3$_{\text{SURP}^{1}}$	**86**	**0.0006**	**-362**	**0.0006**	**145**	**0.0005**
	GPT-3$_{\text{SURP}^{>1}}$ vs. GPT-3$_{\text{SURP}^{1}}$	**32**	**0.0028**	**-192**	**0.0006**	**211**	**0.0005**
	GPT-3$_{\text{SURP}^{<1}}$ vs. GPT-3$_{\text{SURP}^{\geq 1}}$	**68**	**0.0006**	**-209**	**0.0006**	**48**	**0.0005**
	GPT-3$_{\text{SURP}^{>1}}$ vs. GPT-3$_{\text{SURP}^{\leq 1}}$	-10	0.8943	21	1.0000	**94**	**0.0005**
All LMs	All-LMs$_{f(\text{SURP})}$ vs. $\emptyset$	**87**	**0.0005**	**228**	**0.0006**	**121**	**0.0006**
	All-LMs$_{f(\text{SURP})}$ vs. All-LMs$_{\text{PROB}}$	**47**	**0.0005**	**194**	**0.0006**	-129	—
	All-LMs$_{f(\text{SURP})}$ vs. All-LMs$_{\text{SURP}^{1/2}}$	-40	—	**305**	**0.0006**	-100	—
	All-LMs$_{f(\text{SURP})}$ vs. All-LMs$_{\text{SURP}^{3/4}}$	-11	—	**162**	**0.0006**	-118	—
	All-LMs$_{f(\text{SURP})}$ vs. All-LMs$_{\text{SURP}^{1}}$	**40**	**0.0005**	**148**	**0.0006**	-110	—
	All-LMs$_{f(\text{SURP})}$ vs. All-LMs$_{\text{SURP}^{4/3}}$	**17**	**0.0157**	-26	—	-146	—
	All-LMs$_{f(\text{SURP})}$ vs. All-LMs$_{\text{SURP}^{2}}$	**87**	**0.0005**	**171**	**0.0006**	-166	—
	All-LMs$_{\text{PROB}}$ vs. $\emptyset$	**41**	**0.0009**	34	1.0000	**250**	**0.0006**
	All-LMs$_{\text{SURP}^{1/2}}$ vs. $\emptyset$	**127**	**0.0005**	-77	—	**220**	**0.0006**
	All-LMs$_{\text{SURP}^{3/4}}$ vs. $\emptyset$	**98**	**0.0005**	66	0.8408	**239**	**0.0006**
	All-LMs$_{\text{SURP}^{1}}$ vs. $\emptyset$	**47**	**0.0005**	81	0.4274	**230**	**0.0006**
	All-LMs$_{\text{SURP}^{4/3}}$ vs. $\emptyset$	**70**	**0.0005**	**254**	**0.0006**	**267**	**0.0006**
	All-LMs$_{\text{SURP}^{2}}$ vs. $\emptyset$	0	1.0000	57	1.0000	**287**	**0.0006**
	All-LMs$_{\text{SURP}^{1/2}}$ vs. All-LMs$_{\text{PROB}}$	**86**	**0.0005**	**-111**	**0.0006**	**-30**	**0.0006**
	All-LMs$_{\text{SURP}^{3/4}}$ vs. All-LMs$_{\text{PROB}}$	**58**	**0.0005**	32	1.0000	-11	0.7599
	All-LMs$_{\text{SURP}^{1}}$ vs. All-LMs$_{\text{PROB}}$	7	1.0000	47	0.4274	**-19**	**0.0320**
	All-LMs$_{\text{SURP}^{4/3}}$ vs. All-LMs$_{\text{PROB}}$	**30**	**0.0005**	**221**	**0.0006**	17	0.0923
	All-LMs$_{\text{SURP}^{2}}$ vs. All-LMs$_{\text{PROB}}$	**-40**	**0.0005**	23	1.0000	**37**	**0.0006**
	All-LMs$_{\text{SURP}^{3/4}}$ vs. All-LMs$_{\text{SURP}^{1/2}}$	**-28**	**0.0009**	**143**	**0.0006**	19	0.1665
	All-LMs$_{\text{SURP}^{1}}$ vs. All-LMs$_{\text{SURP}^{1/2}}$	**-80**	**0.0005**	**157**	**0.0006**	10	0.9333
	All-LMs$_{\text{SURP}^{4/3}}$ vs. All-LMs$_{\text{SURP}^{1/2}}$	**-57**	**0.0005**	**331**	**0.0006**	**47**	**0.0006**
	All-LMs$_{\text{SURP}^{2}}$ vs. All-LMs$_{\text{SURP}^{1/2}}$	**-127**	**0.0005**	**134**	**0.0006**	**66**	**0.0006**
	All-LMs$_{\text{SURP}^{1}}$ vs. All-LMs$_{\text{SURP}^{3/4}}$	**-51**	**0.0005**	15	1.0000	-9	1.0000
	All-LMs$_{\text{SURP}^{4/3}}$ vs. All-LMs$_{\text{SURP}^{3/4}}$	**-28**	**0.0005**	**189**	**0.0006**	**28**	**0.0032**
	All-LMs$_{\text{SURP}^{2}}$ vs. All-LMs$_{\text{SURP}^{3/4}}$	**-98**	**0.0005**	-9	1.0000	**48**	**0.0006**
	All-LMs$_{\text{SURP}^{4/3}}$ vs. All-LMs$_{\text{SURP}^{1}}$	**23**	**0.0009**	**174**	**0.0006**	**37**	**0.0006**
	All-LMs$_{\text{SURP}^{2}}$ vs. All-LMs$_{\text{SURP}^{1}}$	**-47**	**0.0005**	-23	1.0000	**56**	**0.0006**
	All-LMs$_{\text{SURP}^{2}}$ vs. All-LMs$_{\text{SURP}^{4/3}}$	**-70**	**0.0005**	**-197**	**0.0006**	**20**	**0.0249**
	All-LMs$_{\text{SURP}^{<1}}$ vs. All-LMs$_{\text{SURP}^{1}}$	**66**	**0.0005**	**-96**	**0.0006**	8	1.0000
	All-LMs$_{\text{SURP}^{>1}}$ vs. All-LMs$_{\text{SURP}^{1}}$	**-24**	**0.0005**	**103**	**0.0006**	**49**	**0.0006**
	All-LMs$_{\text{SURP}^{<1}}$ vs. All-LMs$_{\text{SURP}^{\geq 1}}$	**75**	**0.0005**	**-149**	**0.0006**	**-25**	**0.0006**
	All-LMs$_{\text{SURP}^{>1}}$ vs. All-LMs$_{\text{SURP}^{\leq 1}}$	**-66**	**0.0005**	**133**	**0.0006**	**41**	**0.0006**

Group	Comparison	Brown (SPR) ΔLL	Brown (SPR) p	NatStor (SPR) ΔLL	NatStor (SPR) p	NatStor (Maze) ΔLL	NatStor (Maze) p
Between LMs	PCFG$_{f(\text{SURP})}$ vs. n-gram$_{f(\text{SURP})}$	**101**	**0.0006**	**365**	**0.0005**	**-122**	**0.0004**
	GPT-2$_{f(\text{SURP})}$ vs. n-gram$_{f(\text{SURP})}$	**141**	**0.0006**	44	1.0000	**164**	**0.0004**
	GPT-J$_{f(\text{SURP})}$ vs. n-gram$_{f(\text{SURP})}$	**157**	**0.0006**	**283**	**0.0005**	**261**	**0.0004**
	GPT-3$_{f(\text{SURP})}$ vs. n-gram$_{f(\text{SURP})}$	**140**	**0.0006**	**492**	**0.0005**	**226**	**0.0004**
	GPT-2$_{f(\text{SURP})}$ vs. PCFG$_{f(\text{SURP})}$	**39**	**0.0071**	**-321**	**0.0005**	**286**	**0.0004**
	GPT-J$_{f(\text{SURP})}$ vs. PCFG$_{f(\text{SURP})}$	**55**	**0.0006**	-82	0.5045	**383**	**0.0004**
	GPT-3$_{f(\text{SURP})}$ vs. PCFG$_{f(\text{SURP})}$	**39**	**0.0057**	127	0.1439	**348**	**0.0004**
	GPT-J$_{f(\text{SURP})}$ vs. GPT-2$_{f(\text{SURP})}$	16	0.6696	**239**	**0.0005**	**96**	**0.0004**
	GPT-3$_{f(\text{SURP})}$ vs. GPT-2$_{f(\text{SURP})}$	-1	1.0000	**448**	**0.0005**	**62**	**0.0004**
	GPT-3$_{f(\text{SURP})}$ vs. GPT-J$_{f(\text{SURP})}$	-17	0.5193	**209**	**0.0017**	**-35**	**0.0047**
	GPT-2PCFG$_{f(\text{SURP})}$ vs. GPT-2$_{f(\text{SURP})}$	-99	—	**267**	**0.0005**	-49	—
GPT-2 (Normal Error)	GPT-2$_{f(\text{SURP})}$ vs. $\emptyset$	85	1.0000	506	0.1911	-26	—
	GPT-2$_{f(\text{SURP})}$ vs. GPT-2$_{\text{PROB}}$	**573**	**0.0019**	522	0.2013	-51	—
	GPT-2$_{f(\text{SURP})}$ vs. GPT-2$_{\text{SURP}^{1/2}}$	54	1.0000	16	1.0000	2	1.0000
	GPT-2$_{f(\text{SURP})}$ vs. GPT-2$_{\text{SURP}^{3/4}}$	152	0.1493	326	0.6829	**95**	**0.0006**
	GPT-2$_{f(\text{SURP})}$ vs. GPT-2$_{\text{SURP}^{1}}$	**219**	**0.0101**	584	0.0563	-61	—
	GPT-2$_{f(\text{SURP})}$ vs. GPT-2$_{\text{SURP}^{4/3}}$	2	1.0000	484	0.2013	**121**	**0.0006**
	GPT-2$_{f(\text{SURP})}$ vs. GPT-2$_{\text{SURP}^{2}}$	95	0.7577	-38	—	**202**	**0.0006**
	GPT-2$_{\text{PROB}}$ vs. $\emptyset$	-488	—	-16	—	25	1.0000
	GPT-2$_{\text{SURP}^{1/2}}$ vs. $\emptyset$	32	1.0000	490	0.1212	-28	—
	GPT-2$_{\text{SURP}^{3/4}}$ vs. $\emptyset$	-67	—	180	1.0000	-121	—
	GPT-2$_{\text{SURP}^{1}}$ vs. $\emptyset$	-134	—	-78	—	35	0.3376
	GPT-2$_{\text{SURP}^{4/3}}$ vs. $\emptyset$	83	1.0000	23	1.0000	-146	—
	GPT-2$_{\text{SURP}^{2}}$ vs. $\emptyset$	-10	—	544	0.1212	-228	—
	GPT-2$_{\text{SURP}^{1/2}}$ vs. GPT-2$_{\text{PROB}}$	**519**	**0.0019**	506	0.2013	**-52**	**0.0022**
	GPT-2$_{\text{SURP}^{3/4}}$ vs. GPT-2$_{\text{PROB}}$	**421**	**0.0019**	196	1.0000	**-146**	**0.0006**
	GPT-2$_{\text{SURP}^{1}}$ vs. GPT-2$_{\text{PROB}}$	**354**	**0.0019**	-62	1.0000	10	1.0000
	GPT-2$_{\text{SURP}^{4/3}}$ vs. GPT-2$_{\text{PROB}}$	**571**	**0.0019**	39	1.0000	**-171**	**0.0006**
	GPT-2$_{\text{SURP}^{2}}$ vs. GPT-2$_{\text{PROB}}$	**478**	**0.0019**	560	0.2013	**-253**	**0.0006**
	GPT-2$_{\text{SURP}^{3/4}}$ vs. GPT-2$_{\text{SURP}^{1/2}}$	-98	0.6688	-310	0.5795	**-94**	**0.0006**
	GPT-2$_{\text{SURP}^{1}}$ vs. GPT-2$_{\text{SURP}^{1/2}}$	-165	0.0708	-568	0.0519	**63**	**0.0011**
	GPT-2$_{\text{SURP}^{4/3}}$ vs. GPT-2$_{\text{SURP}^{1/2}}$	51	1.0000	-468	0.2013	**-119**	**0.0006**
	GPT-2$_{\text{SURP}^{2}}$ vs. GPT-2$_{\text{SURP}^{1/2}}$	-42	1.0000	54	1.0000	**-200**	**0.0006**
	GPT-2$_{\text{SURP}^{1}}$ vs. GPT-2$_{\text{SURP}^{3/4}}$	-67	1.0000	-258	1.0000	**156**	**0.0006**
	GPT-2$_{\text{SURP}^{4/3}}$ vs. GPT-2$_{\text{SURP}^{3/4}}$	150	0.1618	-158	1.0000	-25	0.4050
	GPT-2$_{\text{SURP}^{2}}$ vs. GPT-2$_{\text{SURP}^{3/4}}$	57	1.0000	364	0.5795	**-107**	**0.0006**
	GPT-2$_{\text{SURP}^{4/3}}$ vs. GPT-2$_{\text{SURP}^{1}}$	**217**	**0.0032**	101	1.0000	**-181**	**0.0006**
	GPT-2$_{\text{SURP}^{2}}$ vs. GPT-2$_{\text{SURP}^{1}}$	124	0.1837	622	0.0563	**-263**	**0.0006**
	GPT-2$_{\text{SURP}^{2}}$ vs. GPT-2$_{\text{SURP}^{4/3}}$	-93	0.7539	521	0.2013	**-81**	**0.0006**
	GPT-2$_{\text{SURP}^{<1}}$ vs. GPT-2$_{\text{SURP}^{1}}$	105	0.3720	452	0.0617	**-116**	**0.0006**
	GPT-2$_{\text{SURP}^{>1}}$ vs. GPT-2$_{\text{SURP}^{1}}$	**158**	**0.0195**	274	0.9545	**-235**	**0.0006**
	GPT-2$_{\text{SURP}^{<1}}$ vs. GPT-2$_{\text{SURP}^{\geq 1}}$	-7	1.0000	298	0.2013	**31**	**0.0358**
	GPT-2$_{\text{SURP}^{>1}}$ vs. GPT-2$_{\text{SURP}^{\leq 1}}$	88	0.2581	16	1.0000	**-138**	**0.0006**
	GPT-2$^{(\text{EXGAUSS})}_{f(\text{SURP})}$ vs. GPT-2$^{(\text{NORMAL})}_{f(\text{SURP})}$	**14920**	**0.0006**	**120357**	**0.0013**	**2260**	**0.0004**
Prob vs. Surp	GPT-2$_{\text{PROB}+\text{SURP}^{1}}$ vs. GPT-2$_{\text{PROB}}$	-65	—	**577**	**0.0001**	**384**	**0.0001**
	GPT-2$_{\text{PROB}+\text{SURP}^{1}}$ vs. GPT-2$_{\text{SURP}^{1}}$	-17	—	**423**	**0.0001**	**137**	**0.0001**

Table S5. Testing Results on Self-Paced (Reading and Maze) Datasets. Results of key statistical comparisons based on permutation tests of the difference in average test set likelihood between the alternative and null model in each comparison (ΔLL). Subscripts indicate the assumed functional form of predictability effects: $f(\text{SURP})$ (nonlinear in surprisal), PROB (linear in probability), and SURP^a (linear in surprisal raised to exponent a, such that e.g., SURP^1 is linear in surprisal). Boldface indicates statistical significance. In directional tests where one hypothesis subsumes the other (e.g., $f(\text{SURP})$ vs. SURP^1, since a nonlinear surprisal effect subsumes a linear one), dashes (—) indicate failure of the alternative hypothesis (left) to improve over the null hypothesis (right). Otherwise, cells are color coded using cyan to indicate that the hypothesis on the left outperforms the one on the right, and using magenta to indicate that the hypothesis on the right outperforms the one on the left. All p-values are corrected for false discovery rate (15) within each family of tests (delimited by single horizontal lines). Key comparisons are highlighted.

	Comparison	Scan Path ΔLL	Scan Path p	First Pass ΔLL	First Pass p	Go Past ΔLL	Go Past p
n-gram	n-gram$_{f(\text{SURP})}$ vs. $\emptyset$	**137**	**0.0007**	**113**	**0.0010**	**140**	**0.0009**
	n-gram$_{f(\text{SURP})}$ vs. n-gram$_{\text{PROB}}$	**139**	**0.0007**	**130**	**0.0010**	**145**	**0.0009**
	n-gram$_{f(\text{SURP})}$ vs. n-gram$_{\text{SURP}^{1/2}}$	**29**	**0.0161**	19	0.1695	-28	—
	n-gram$_{f(\text{SURP})}$ vs. n-gram$_{\text{SURP}^{3/4}}$	-9	—	-7	—	-27	—
	n-gram$_{f(\text{SURP})}$ vs. n-gram$_{\text{SURP}^{1}}$	-25	—	-18	—	-42	—
	n-gram$_{f(\text{SURP})}$ vs. n-gram$_{\text{SURP}^{4/3}}$	-19	—	-14	—	-43	—
	n-gram$_{f(\text{SURP})}$ vs. n-gram$_{\text{SURP}^{2}}$	17	0.3926	1	1.0000	4	1.0000
	n-gram$_{\text{PROB}}$ vs. $\emptyset$	-2	—	-17	—	-5	—
	n-gram$_{\text{SURP}^{1/2}}$ vs. $\emptyset$	**108**	**0.0007**	**95**	**0.0010**	**168**	**0.0009**
	n-gram$_{\text{SURP}^{3/4}}$ vs. $\emptyset$	**146**	**0.0007**	**120**	**0.0010**	**167**	**0.0009**
	n-gram$_{\text{SURP}^{1}}$ vs. $\emptyset$	**162**	**0.0007**	**131**	**0.0010**	**181**	**0.0009**
	n-gram$_{\text{SURP}^{4/3}}$ vs. $\emptyset$	**156**	**0.0007**	**127**	**0.0010**	**183**	**0.0009**
	n-gram$_{\text{SURP}^{2}}$ vs. $\emptyset$	**120**	**0.0007**	**112**	**0.0010**	**136**	**0.0009**
	n-gram$_{\text{SURP}^{1/2}}$ vs. n-gram$_{\text{PROB}}$	**110**	**0.0007**	**112**	**0.0010**	**172**	**0.0009**
	n-gram$_{\text{SURP}^{3/4}}$ vs. n-gram$_{\text{PROB}}$	**148**	**0.0007**	**138**	**0.0010**	**172**	**0.0009**
	n-gram$_{\text{SURP}^{1}}$ vs. n-gram$_{\text{PROB}}$	**164**	**0.0007**	**148**	**0.0010**	**186**	**0.0009**
	n-gram$_{\text{SURP}^{4/3}}$ vs. n-gram$_{\text{PROB}}$	**158**	**0.0007**	**144**	**0.0010**	**188**	**0.0009**
	n-gram$_{\text{SURP}^{2}}$ vs. n-gram$_{\text{PROB}}$	**122**	**0.0007**	**130**	**0.0010**	**140**	**0.0009**
	n-gram$_{\text{SURP}^{3/4}}$ vs. n-gram$_{\text{SURP}^{1/2}}$	**38**	**0.0007**	**26**	**0.0043**	0	1.0000
	n-gram$_{\text{SURP}^{1}}$ vs. n-gram$_{\text{SURP}^{1/2}}$	**55**	**0.0007**	**36**	**0.0010**	14	0.9135
	n-gram$_{\text{SURP}^{4/3}}$ vs. n-gram$_{\text{SURP}^{1/2}}$	**49**	**0.0007**	**32**	**0.0019**	15	0.8981
	n-gram$_{\text{SURP}^{2}}$ vs. n-gram$_{\text{SURP}^{1/2}}$	12	0.8008	18	0.1695	**-32**	**0.0390**
	n-gram$_{\text{SURP}^{1}}$ vs. n-gram$_{\text{SURP}^{3/4}}$	17	0.2171	10	0.7597	14	0.8981
	n-gram$_{\text{SURP}^{4/3}}$ vs. n-gram$_{\text{SURP}^{3/4}}$	10	0.9795	7	1.0000	16	0.8875
	n-gram$_{\text{SURP}^{2}}$ vs. n-gram$_{\text{SURP}^{3/4}}$	**-26**	**0.0224**	-8	1.0000	**-32**	**0.0374**
	n-gram$_{\text{SURP}^{4/3}}$ vs. n-gram$_{\text{SURP}^{1}}$	-6	1.0000	-4	1.0000	2	1.0000
	n-gram$_{\text{SURP}^{2}}$ vs. n-gram$_{\text{SURP}^{1}}$	**-42**	**0.0007**	-18	0.1199	**-46**	**0.0009**
	n-gram$_{\text{SURP}^{2}}$ vs. n-gram$_{\text{SURP}^{4/3}}$	**-36**	**0.0007**	-14	0.2916	**-47**	**0.0009**
	n-gram$_{\text{SURP}^{<1}}$ vs. n-gram$_{\text{SURP}^{1}}$	**-41**	**0.0007**	**-21**	**0.0130**	-14	0.7845
	n-gram$_{\text{SURP}^{>1}}$ vs. n-gram$_{\text{SURP}^{1}}$	**-26**	**0.0026**	-13	0.2659	-20	0.2399
	n-gram$_{\text{SURP}^{<1}}$ vs. n-gram$_{\text{SURP}^{\geq 1}}$	**-24**	**0.0007**	-11	0.2213	-1	1.0000
	n-gram$_{\text{SURP}^{>1}}$ vs. n-gram$_{\text{SURP}^{\leq 1}}$	-7	1.0000	0	1.0000	-9	1.0000
PCFG	PCFG$_{f(\text{SURP})}$ vs. $\emptyset$	**47**	**0.0026**	**30**	**0.0195**	**42**	**0.0260**
	PCFG$_{f(\text{SURP})}$ vs. PCFG$_{\text{PROB}}$	24	0.1794	**30**	**0.0173**	32	0.1315
	PCFG$_{f(\text{SURP})}$ vs. PCFG$_{\text{SURP}^{1/2}}$	21	0.3240	18	0.2757	8	1.0000
	PCFG$_{f(\text{SURP})}$ vs. PCFG$_{\text{SURP}^{3/4}}$	28	0.0637	11	1.0000	8	1.0000
	PCFG$_{f(\text{SURP})}$ vs. PCFG$_{\text{SURP}^{1}}$	**38**	**0.0074**	**24**	**0.0427**	3	1.0000
	PCFG$_{f(\text{SURP})}$ vs. PCFG$_{\text{SURP}^{4/3}}$	**42**	**0.0043**	**31**	**0.0173**	-8	—
	PCFG$_{f(\text{SURP})}$ vs. PCFG$_{\text{SURP}^{2}}$	**65**	**0.0026**	**39**	**0.0130**	5	1.0000
	PCFG$_{\text{PROB}}$ vs. $\emptyset$	23	0.0637	-1	—	10	1.0000
	PCFG$_{\text{SURP}^{1/2}}$ vs. $\emptyset$	**26**	**0.0462**	12	0.9718	34	0.0853
	PCFG$_{\text{SURP}^{3/4}}$ vs. $\emptyset$	19	0.2406	19	0.2056	35	0.0649
	PCFG$_{\text{SURP}^{1}}$ vs. $\emptyset$	9	1.0000	6	1.0000	**39**	**0.0325**
	PCFG$_{\text{SURP}^{4/3}}$ vs. $\emptyset$	5	1.0000	-1	—	**50**	**0.0260**
	PCFG$_{\text{SURP}^{2}}$ vs. $\emptyset$	-18	—	-10	—	38	0.0597
	PCFG$_{\text{SURP}^{1/2}}$ vs. PCFG$_{\text{PROB}}$	3	1.0000	13	0.8808	24	0.4242
	PCFG$_{\text{SURP}^{3/4}}$ vs. PCFG$_{\text{PROB}}$	-4	1.0000	20	0.1983	25	0.4242
	PCFG$_{\text{SURP}^{1}}$ vs. PCFG$_{\text{PROB}}$	-14	0.6628	6	1.0000	29	0.2121
	PCFG$_{\text{SURP}^{4/3}}$ vs. PCFG$_{\text{PROB}}$	-18	0.3556	0	1.0000	**40**	**0.0260**
	PCFG$_{\text{SURP}^{2}}$ vs. PCFG$_{\text{PROB}}$	**-41**	**0.0026**	-9	1.0000	28	0.3298
	PCFG$_{\text{SURP}^{3/4}}$ vs. PCFG$_{\text{SURP}^{1/2}}$	-7	1.0000	7	1.0000	1	1.0000
	PCFG$_{\text{SURP}^{1}}$ vs. PCFG$_{\text{SURP}^{1/2}}$	-17	0.3240	-6	1.0000	5	1.0000
	PCFG$_{\text{SURP}^{4/3}}$ vs. PCFG$_{\text{SURP}^{1/2}}$	-21	0.1714	-13	0.8327	16	1.0000
	PCFG$_{\text{SURP}^{2}}$ vs. PCFG$_{\text{SURP}^{1/2}}$	**-44**	**0.0026**	-22	0.1519	3	1.0000
	PCFG$_{\text{SURP}^{1}}$ vs. PCFG$_{\text{SURP}^{3/4}}$	-10	1.0000	-13	0.6805	4	1.0000
	PCFG$_{\text{SURP}^{4/3}}$ vs. PCFG$_{\text{SURP}^{3/4}}$	-14	0.6086	-20	0.1198	16	1.0000
	PCFG$_{\text{SURP}^{2}}$ vs. PCFG$_{\text{SURP}^{3/4}}$	**-37**	**0.0026**	**-28**	**0.0238**	3	1.0000
	PCFG$_{\text{SURP}^{4/3}}$ vs. PCFG$_{\text{SURP}^{1}}$	-4	1.0000	-7	1.0000	11	1.0000
	PCFG$_{\text{SURP}^{2}}$ vs. PCFG$_{\text{SURP}^{1}}$	**-27**	**0.0373**	-15	0.5213	-1	1.0000
	PCFG$_{\text{SURP}^{2}}$ vs. PCFG$_{\text{SURP}^{4/3}}$	-23	0.0918	-8	1.0000	-13	1.0000
	PCFG$_{\text{SURP}^{<1}}$ vs. PCFG$_{\text{SURP}^{1}}$	12	0.6628	9	1.0000	-4	1.0000
	PCFG$_{\text{SURP}^{>1}}$ vs. PCFG$_{\text{SURP}^{1}}$	-5	1.0000	-10	1.0000	9	1.0000
	PCFG$_{\text{SURP}^{<1}}$ vs. PCFG$_{\text{SURP}^{\geq 1}}$	16	0.0918	15	0.0747	-13	0.9387
	PCFG$_{\text{SURP}^{>1}}$ vs. PCFG$_{\text{SURP}^{\leq 1}}$	-16	0.0671	**-18**	**0.0208**	13	0.8371
GPT-2	GPT-2$_{f(\text{SURP})}$ vs. $\emptyset$	**245**	**0.0006**	**211**	**0.0006**	**283**	**0.0007**
	GPT-2$_{f(\text{SURP})}$ vs. GPT-2$_{\text{PROB}}$	**188**	**0.0006**	**170**	**0.0006**	**237**	**0.0007**
	GPT-2$_{f(\text{SURP})}$ vs. GPT-2$_{\text{SURP}^{1/2}}$	**39**	**0.0006**	**35**	**0.0006**	**44**	**0.0020**
	GPT-2$_{f(\text{SURP})}$ vs. GPT-2$_{\text{SURP}^{3/4}}$	-5	—	9	1.0000	11	1.0000
	GPT-2$_{f(\text{SURP})}$ vs. GPT-2$_{\text{SURP}^{1}}$	3	1.0000	12	0.5835	**36**	**0.0141**
	GPT-2$_{f(\text{SURP})}$ vs. GPT-2$_{\text{SURP}^{4/3}}$	12	0.7253	18	0.1292	**33**	**0.0169**
	GPT-2$_{f(\text{SURP})}$ vs. GPT-2$_{\text{SURP}^{2}}$	**87**	**0.0006**	**84**	**0.0006**	**103**	**0.0007**
	GPT-2$_{\text{PROB}}$ vs. $\emptyset$	**57**	**0.0006**	**42**	**0.0006**	**46**	**0.0014**
	GPT-2$_{\text{SURP}^{1/2}}$ vs. $\emptyset$	**206**	**0.0006**	**177**	**0.0006**	**240**	**0.0007**
	GPT-2$_{\text{SURP}^{3/4}}$ vs. $\emptyset$	**250**	**0.0006**	**202**	**0.0006**	**272**	**0.0007**
	GPT-2$_{\text{SURP}^{1}}$ vs. $\emptyset$	**242**	**0.0006**	**199**	**0.0006**	**247**	**0.0007**
	GPT-2$_{\text{SURP}^{4/3}}$ vs. $\emptyset$	**232**	**0.0006**	**194**	**0.0006**	**250**	**0.0007**
	GPT-2$_{\text{SURP}^{2}}$ vs. $\emptyset$	**158**	**0.0006**	**127**	**0.0006**	**180**	**0.0007**
	GPT-2$_{\text{SURP}^{1/2}}$ vs. GPT-2$_{\text{PROB}}$	**150**	**0.0006**	**135**	**0.0006**	**194**	**0.0007**
	GPT-2$_{\text{SURP}^{3/4}}$ vs. GPT-2$_{\text{PROB}}$	**194**	**0.0006**	**161**	**0.0006**	**227**	**0.0007**
	GPT-2$_{\text{SURP}^{1}}$ vs. GPT-2$_{\text{PROB}}$	**186**	**0.0006**	**157**	**0.0006**	**201**	**0.0007**
	GPT-2$_{\text{SURP}^{4/3}}$ vs. GPT-2$_{\text{PROB}}$	**176**	**0.0006**	**152**	**0.0006**	**204**	**0.0007**
	GPT-2$_{\text{SURP}^{2}}$ vs. GPT-2$_{\text{PROB}}$	**102**	**0.0006**	**86**	**0.0006**	**135**	**0.0007**
	GPT-2$_{\text{SURP}^{3/4}}$ vs. GPT-2$_{\text{SURP}^{1/2}}$	**44**	**0.0006**	**26**	**0.0023**	**33**	**0.0104**
	GPT-2$_{\text{SURP}^{1}}$ vs. GPT-2$_{\text{SURP}^{1/2}}$	**36**	**0.0006**	**22**	**0.0216**	7	1.0000
	GPT-2$_{\text{SURP}^{4/3}}$ vs. GPT-2$_{\text{SURP}^{1/2}}$	**26**	**0.0119**	17	0.1409	10	1.0000
	GPT-2$_{\text{SURP}^{2}}$ vs. GPT-2$_{\text{SURP}^{1/2}}$	**-48**	**0.0006**	**-49**	**0.0006**	**-59**	**0.0007**
	GPT-2$_{\text{SURP}^{1}}$ vs. GPT-2$_{\text{SURP}^{3/4}}$	-8	1.0000	-4	1.0000	-25	0.0934
	GPT-2$_{\text{SURP}^{4/3}}$ vs. GPT-2$_{\text{SURP}^{3/4}}$	-18	0.1643	-9	1.0000	-23	0.1764
	GPT-2$_{\text{SURP}^{2}}$ vs. GPT-2$_{\text{SURP}^{3/4}}$	**-92**	**0.0006**	**-75**	**0.0006**	**-92**	**0.0007**
	GPT-2$_{\text{SURP}^{4/3}}$ vs. GPT-2$_{\text{SURP}^{1}}$	-10	1.0000	-5	1.0000	3	1.0000
	GPT-2$_{\text{SURP}^{2}}$ vs. GPT-2$_{\text{SURP}^{1}}$	**-84**	**0.0006**	**-72**	**0.0006**	**-67**	**0.0007**
	GPT-2$_{\text{SURP}^{2}}$ vs. GPT-2$_{\text{SURP}^{4/3}}$	**-74**	**0.0006**	**-66**	**0.0006**	**-69**	**0.0007**
	GPT-2$_{\text{SURP}^{<1}}$ vs. GPT-2$_{\text{SURP}^{1}}$	**-21**	**0.0140**	-9	0.8909	9	1.0000
	GPT-2$_{\text{SURP}^{>1}}$ vs. GPT-2$_{\text{SURP}^{1}}$	**-53**	**0.0006**	**-39**	**0.0006**	-20	0.2467
	GPT-2$_{\text{SURP}^{<1}}$ vs. GPT-2$_{\text{SURP}^{\geq 1}}$	**20**	**0.0043**	**19**	**0.0028**	20	0.0631
	GPT-2$_{\text{SURP}^{>1}}$ vs. GPT-2$_{\text{SURP}^{\leq 1}}$	**-33**	**0.0006**	**-33**	**0.0006**	**-25**	**0.0104**
GPT-J	GPT-J$_{f(\text{SURP})}$ vs. $\emptyset$	**140**	**0.0005**	**114**	**0.0008**	**160**	**0.0011**
	GPT-J$_{f(\text{SURP})}$ vs. GPT-J$_{\text{PROB}}$	**68**	**0.0005**	**62**	**0.0008**	**121**	**0.0011**
	GPT-J$_{f(\text{SURP})}$ vs. GPT-J$_{\text{SURP}^{1/2}}$	-42	—	-24	—	-1	—
	GPT-J$_{f(\text{SURP})}$ vs. GPT-J$_{\text{SURP}^{3/4}}$	20	0.1289	13	0.5209	22	0.4331
	GPT-J$_{f(\text{SURP})}$ vs. GPT-J$_{\text{SURP}^{1}}$	6	1.0000	8	1.0000	17	0.7007
	GPT-J$_{f(\text{SURP})}$ vs. GPT-J$_{\text{SURP}^{4/3}}$	-7	—	2	1.0000	25	0.2583
	GPT-J$_{f(\text{SURP})}$ vs. GPT-J$_{\text{SURP}^{2}}$	**60**	**0.0005**	**35**	**0.0008**	**32**	**0.0427**
	GPT-J$_{\text{PROB}}$ vs. $\emptyset$	**72**	**0.0005**	**52**	**0.0008**	**40**	**0.0080**
	GPT-J$_{\text{SURP}^{1/2}}$ vs. $\emptyset$	**183**	**0.0005**	**138**	**0.0008**	**162**	**0.0011**
	GPT-J$_{\text{SURP}^{3/4}}$ vs. $\emptyset$	**121**	**0.0005**	**101**	**0.0008**	**139**	**0.0011**
	GPT-J$_{\text{SURP}^{1}}$ vs. $\emptyset$	**134**	**0.0005**	**106**	**0.0008**	**143**	**0.0011**
	GPT-J$_{\text{SURP}^{4/3}}$ vs. $\emptyset$	**147**	**0.0005**	**112**	**0.0008**	**136**	**0.0011**
	GPT-J$_{\text{SURP}^{2}}$ vs. $\emptyset$	**81**	**0.0005**	**79**	**0.0008**	**128**	**0.0011**
	GPT-J$_{\text{SURP}^{1/2}}$ vs. GPT-J$_{\text{PROB}}$	**110**	**0.0005**	**86**	**0.0008**	**122**	**0.0011**
	GPT-J$_{\text{SURP}^{3/4}}$ vs. GPT-J$_{\text{PROB}}$	**49**	**0.0005**	**49**	**0.0008**	**99**	**0.0011**
	GPT-J$_{\text{SURP}^{1}}$ vs. GPT-J$_{\text{PROB}}$	**62**	**0.0005**	**54**	**0.0008**	**103**	**0.0011**
	GPT-J$_{\text{SURP}^{4/3}}$ vs. GPT-J$_{\text{PROB}}$	**75**	**0.0005**	**60**	**0.0008**	**96**	**0.0011**
	GPT-J$_{\text{SURP}^{2}}$ vs. GPT-J$_{\text{PROB}}$	9	1.0000	**26**	**0.0400**	**88**	**0.0011**
	GPT-J$_{\text{SURP}^{3/4}}$ vs. GPT-J$_{\text{SURP}^{1/2}}$	**-62**	**0.0005**	**-37**	**0.0008**	-23	0.4073
	GPT-J$_{\text{SURP}^{1}}$ vs. GPT-J$_{\text{SURP}^{1/2}}$	**-49**	**0.0005**	**-32**	**0.0014**	-19	0.6942
	GPT-J$_{\text{SURP}^{4/3}}$ vs. GPT-J$_{\text{SURP}^{1/2}}$	**-36**	**0.0005**	**-26**	**0.0117**	-26	0.2583
	GPT-J$_{\text{SURP}^{2}}$ vs. GPT-J$_{\text{SURP}^{1/2}}$	**-102**	**0.0005**	**-59**	**0.0008**	-34	0.0710
	GPT-J$_{\text{SURP}^{1}}$ vs. GPT-J$_{\text{SURP}^{3/4}}$	13	0.5203	5	1.0000	5	1.0000
	GPT-J$_{\text{SURP}^{4/3}}$ vs. GPT-J$_{\text{SURP}^{3/4}}$	**26**	**0.0135**	11	0.8789	-3	1.0000
	GPT-J$_{\text{SURP}^{2}}$ vs. GPT-J$_{\text{SURP}^{3/4}}$	**-40**	**0.0005**	**-22**	**0.0363**	-10	1.0000
	GPT-J$_{\text{SURP}^{4/3}}$ vs. GPT-J$_{\text{SURP}^{1}}$	13	0.5203	6	1.0000	-8	1.0000
	GPT-J$_{\text{SURP}^{2}}$ vs. GPT-J$_{\text{SURP}^{1}}$	**-53**	**0.0005**	**-27**	**0.0026**	-15	0.9718
	GPT-J$_{\text{SURP}^{2}}$ vs. GPT-J$_{\text{SURP}^{4/3}}$	**-66**	**0.0005**	**-33**	**0.0014**	-8	1.0000
	GPT-J$_{\text{SURP}^{<1}}$ vs. GPT-J$_{\text{SURP}^{1}}$	**29**	**0.0005**	**19**	**0.0248**	10	1.0000
	GPT-J$_{\text{SURP}^{>1}}$ vs. GPT-J$_{\text{SURP}^{1}}$	**-31**	**0.0010**	-15	0.1868	-10	1.0000
	GPT-J$_{\text{SURP}^{<1}}$ vs. GPT-J$_{\text{SURP}^{\geq 1}}$	**42**	**0.0005**	**26**	**0.0008**	18	0.2459
	GPT-J$_{\text{SURP}^{>1}}$ vs. GPT-J$_{\text{SURP}^{\leq 1}}$	**-35**	**0.0005**	**-23**	**0.0008**	-15	0.4428
GPT-3	GPT-3$_{f(\text{SURP})}$ vs. $\emptyset$	**55**	**0.0008**	**49**	**0.0013**	**84**	**0.0016**
	GPT-3$_{f(\text{SURP})}$ vs. GPT-3$_{\text{PROB}}$	-35	—	-12	—	**42**	**0.0069**
	GPT-3$_{f(\text{SURP})}$ vs. GPT-3$_{\text{SURP}^{1/2}}$	-1	—	11	0.9828	12	1.0000
	GPT-3$_{f(\text{SURP})}$ vs. GPT-3$_{\text{SURP}^{3/4}}$	-3	—	2	1.0000	2	1.0000
	GPT-3$_{f(\text{SURP})}$ vs. GPT-3$_{\text{SURP}^{1}}$	-47	—	-19	—	-28	—
	GPT-3$_{f(\text{SURP})}$ vs. GPT-3$_{\text{SURP}^{4/3}}$	8	1.0000	16	0.3432	13	1.0000
	GPT-3$_{f(\text{SURP})}$ vs. GPT-3$_{\text{SURP}^{2}}$	9	1.0000	17	0.1927	9	1.0000
	GPT-3$_{\text{PROB}}$ vs. $\emptyset$	**90**	**0.0008**	**61**	**0.0013**	**42**	**0.0029**
	GPT-3$_{\text{SURP}^{1/2}}$ vs. $\emptyset$	**57**	**0.0008**	**38**	**0.0013**	**72**	**0.0016**
	GPT-3$_{\text{SURP}^{3/4}}$ vs. $\emptyset$	**58**	**0.0008**	**47**	**0.0013**	**82**	**0.0016**
	GPT-3$_{\text{SURP}^{1}}$ vs. $\emptyset$	**103**	**0.0008**	**68**	**0.0013**	**112**	**0.0016**
	GPT-3$_{\text{SURP}^{4/3}}$ vs. $\emptyset$	**47**	**0.0008**	**33**	**0.0020**	**72**	**0.0016**
	GPT-3$_{\text{SURP}^{2}}$ vs. $\emptyset$	**46**	**0.0008**	**32**	**0.0020**	**75**	**0.0016**
	GPT-3$_{\text{SURP}^{1/2}}$ vs. GPT-3$_{\text{PROB}}$	**-34**	**0.0008**	**-23**	**0.0435**	30	0.0866
	GPT-3$_{\text{SURP}^{3/4}}$ vs. GPT-3$_{\text{PROB}}$	**-32**	**0.0014**	-14	0.5172	**40**	**0.0069**
	GPT-3$_{\text{SURP}^{1}}$ vs. GPT-3$_{\text{PROB}}$	12	0.9968	7	1.0000	**70**	**0.0016**
	GPT-3$_{\text{SURP}^{4/3}}$ vs. GPT-3$_{\text{PROB}}$	**-43**	**0.0008**	**-28**	**0.0164**	29	0.1292
	GPT-3$_{\text{SURP}^{2}}$ vs. GPT-3$_{\text{PROB}}$	**-44**	**0.0008**	**-29**	**0.0074**	33	0.0672
	GPT-3$_{\text{SURP}^{3/4}}$ vs. GPT-3$_{\text{SURP}^{1/2}}$	1	1.0000	9	1.0000	10	1.0000
	GPT-3$_{\text{SURP}^{1}}$ vs. GPT-3$_{\text{SURP}^{1/2}}$	**46**	**0.0008**	**30**	**0.0013**	**40**	**0.0054**
	GPT-3$_{\text{SURP}^{4/3}}$ vs. GPT-3$_{\text{SURP}^{1/2}}$	-10	1.0000	-5	1.0000	-1	1.0000
	GPT-3$_{\text{SURP}^{2}}$ vs. GPT-3$_{\text{SURP}^{1/2}}$	-10	1.0000	-7	1.0000	3	1.0000
	GPT-3$_{\text{SURP}^{1}}$ vs. GPT-3$_{\text{SURP}^{3/4}}$	**45**	**0.0008**	**21**	**0.0365**	30	0.0503
	GPT-3$_{\text{SURP}^{4/3}}$ vs. GPT-3$_{\text{SURP}^{3/4}}$	-11	1.0000	-14	0.5172	-10	1.0000
	GPT-3$_{\text{SURP}^{2}}$ vs. GPT-3$_{\text{SURP}^{3/4}}$	-12	1.0000	-16	0.2831	-7	1.0000
	GPT-3$_{\text{SURP}^{4/3}}$ vs. GPT-3$_{\text{SURP}^{1}}$	**-56**	**0.0008**	**-35**	**0.0013**	**-41**	**0.0060**
	GPT-3$_{\text{SURP}^{2}}$ vs. GPT-3$_{\text{SURP}^{1}}$	**-56**	**0.0008**	**-36**	**0.0013**	**-37**	**0.0047**
	GPT-3$_{\text{SURP}^{2}}$ vs. GPT-3$_{\text{SURP}^{4/3}}$	0	1.0000	-2	1.0000	3	1.0000
	GPT-3$_{\text{SURP}^{<1}}$ vs. GPT-3$_{\text{SURP}^{1}}$	**-45**	**0.0008**	**-27**	**0.0013**	**-39**	**0.0016**
	GPT-3$_{\text{SURP}^{>1}}$ vs. GPT-3$_{\text{SURP}^{1}}$	**-56**	**0.0008**	**-36**	**0.0013**	**-38**	**0.0039**
	GPT-3$_{\text{SURP}^{<1}}$ vs. GPT-3$_{\text{SURP}^{\geq 1}}$	-5	1.0000	-3	1.0000	-3	1.0000
	GPT-3$_{\text{SURP}^{>1}}$ vs. GPT-3$_{\text{SURP}^{\leq 1}}$	**-19**	**0.0097**	**-21**	**0.0020**	-19	0.1156
All LMs	All-LMs$_{f(\text{SURP})}$ vs. $\emptyset$	**90**	**0.0005**	**81**	**0.0007**	**122**	**0.0007**
	All-LMs$_{f(\text{SURP})}$ vs. All-LMs$_{\text{PROB}}$	**58**	**0.0005**	**50**	**0.0007**	**94**	**0.0007**
	All-LMs$_{f(\text{SURP})}$ vs. All-LMs$_{\text{SURP}^{1/2}}$	-18	—	-14	—	-37	—
	All-LMs$_{f(\text{SURP})}$ vs. All-LMs$_{\text{SURP}^{3/4}}$	-12	—	-11	—	-16	—
	All-LMs$_{f(\text{SURP})}$ vs. All-LMs$_{\text{SURP}^{1}}$	-44	—	-25	—	-20	—
	All-LMs$_{f(\text{SURP})}$ vs. All-LMs$_{\text{SURP}^{4/3}}$	-29	—	-18	—	-9	—
	All-LMs$_{f(\text{SURP})}$ vs. All-LMs$_{\text{SURP}^{2}}$	11	0.1749	8	0.6166	17	0.0644
	All-LMs$_{\text{PROB}}$ vs. $\emptyset$	**32**	**0.0005**	**31**	**0.0007**	**27**	**0.0233**
	All-LMs$_{\text{SURP}^{1/2}}$ vs. $\emptyset$	**108**	**0.0005**	**95**	**0.0007**	**158**	**0.0007**
	All-LMs$_{\text{SURP}^{3/4}}$ vs. $\emptyset$	**102**	**0.0005**	**92**	**0.0007**	**138**	**0.0007**
	All-LMs$_{\text{SURP}^{1}}$ vs. $\emptyset$	**134**	**0.0005**	**106**	**0.0007**	**141**	**0.0007**
	All-LMs$_{\text{SURP}^{4/3}}$ vs. $\emptyset$	**119**	**0.0005**	**99**	**0.0007**	**130**	**0.0007**
	All-LMs$_{\text{SURP}^{2}}$ vs. $\emptyset$	**79**	**0.0005**	**73**	**0.0007**	**104**	**0.0007**
	All-LMs$_{\text{SURP}^{1/2}}$ vs. All-LMs$_{\text{PROB}}$	**76**	**0.0005**	**64**	**0.0007**	**131**	**0.0007**
	All-LMs$_{\text{SURP}^{3/4}}$ vs. All-LMs$_{\text{PROB}}$	**70**	**0.0005**	**62**	**0.0007**	**111**	**0.0007**
	All-LMs$_{\text{SURP}^{1}}$ vs. All-LMs$_{\text{PROB}}$	**102**	**0.0005**	**75**	**0.0007**	**114**	**0.0007**
	All-LMs$_{\text{SURP}^{4/3}}$ vs. All-LMs$_{\text{PROB}}$	**87**	**0.0005**	**68**	**0.0007**	**103**	**0.0007**
	All-LMs$_{\text{SURP}^{2}}$ vs. All-LMs$_{\text{PROB}}$	**47**	**0.0005**	**42**	**0.0007**	**77**	**0.0007**
	All-LMs$_{\text{SURP}^{3/4}}$ vs. All-LMs$_{\text{SURP}^{1/2}}$	-5	1.0000	-2	1.0000	**-20**	**0.0071**
	All-LMs$_{\text{SURP}^{1}}$ vs. All-LMs$_{\text{SURP}^{1/2}}$	**26**	**0.0005**	11	0.0919	**-17**	**0.0410**
	All-LMs$_{\text{SURP}^{4/3}}$ vs. All-LMs$_{\text{SURP}^{1/2}}$	12	0.1299	5	1.0000	**-28**	**0.0007**
	All-LMs$_{\text{SURP}^{2}}$ vs. All-LMs$_{\text{SURP}^{1/2}}$	**-29**	**0.0005**	**-21**	**0.0007**	**-54**	**0.0007**
	All-LMs$_{\text{SURP}^{1}}$ vs. All-LMs$_{\text{SURP}^{3/4}}$	**32**	**0.0005**	**13**	**0.0237**	3	1.0000
	All-LMs$_{\text{SURP}^{4/3}}$ vs. All-LMs$_{\text{SURP}^{3/4}}$	**17**	**0.0068**	7	0.7119	-8	1.0000
	All-LMs$_{\text{SURP}^{2}}$ vs. All-LMs$_{\text{SURP}^{3/4}}$	**-24**	**0.0005**	**-19**	**0.0013**	**-34**	**0.0007**
	All-LMs$_{\text{SURP}^{4/3}}$ vs. All-LMs$_{\text{SURP}^{1}}$	**-15**	**0.0269**	-6	0.7980	-11	0.4967
	All-LMs$_{\text{SURP}^{2}}$ vs. All-LMs$_{\text{SURP}^{1}}$	**-55**	**0.0005**	**-32**	**0.0007**	**-37**	**0.0007**
	All-LMs$_{\text{SURP}^{2}}$ vs. All-LMs$_{\text{SURP}^{4/3}}$	**-41**	**0.0005**	**-26**	**0.0007**	**-26**	**0.0031**
	All-LMs$_{\text{SURP}^{<1}}$ vs. All-LMs$_{\text{SURP}^{1}}$	**-29**	**0.0005**	**-12**	**0.0183**	8	0.7885
	All-LMs$_{\text{SURP}^{>1}}$ vs. All-LMs$_{\text{SURP}^{1}}$	**-48**	**0.0005**	**-23**	**0.0007**	**-22**	**0.0013**
	All-LMs$_{\text{SURP}^{<1}}$ vs. All-LMs$_{\text{SURP}^{\geq 1}}$	-6	0.3883	3	1.0000	**19**	**0.0007**
	All-LMs$_{\text{SURP}^{>1}}$ vs. All-LMs$_{\text{SURP}^{\leq 1}}$	**-29**	**0.0005**	**-17**	**0.0007**	**-26**	**0.0007**
Between LMs	PCFG$_{f(\text{SURP})}$ vs. n-gram$_{f(\text{SURP})}$	**-90**	**0.0004**	**-84**	**0.0004**	**-98**	**0.0005**
	GPT-2$_{f(\text{SURP})}$ vs. n-gram$_{f(\text{SURP})}$	**108**	**0.0004**	**98**	**0.0004**	**143**	**0.0005**
	GPT-J$_{f(\text{SURP})}$ vs. n-gram$_{f(\text{SURP})}$	4	1.0000	1	1.0000	21	0.5178
	GPT-3$_{f(\text{SURP})}$ vs. n-gram$_{f(\text{SURP})}$	**-82**	**0.0004**	**-64**	**0.0004**	**-56**	**0.0008**
	GPT-2$_{f(\text{SURP})}$ vs. PCFG$_{f(\text{SURP})}$	**198**	**0.0004**	**182**	**0.0004**	**241**	**0.0005**
	GPT-J$_{f(\text{SURP})}$ vs. PCFG$_{f(\text{SURP})}$	**94**	**0.0004**	**84**	**0.0004**	**118**	**0.0005**
	GPT-3$_{f(\text{SURP})}$ vs. PCFG$_{f(\text{SURP})}$	8	1.0000	19	0.1565	**42**	**0.0066**
	GPT-J$_{f(\text{SURP})}$ vs. GPT-2$_{f(\text{SURP})}$	**-104**	**0.0004**	**-97**	**0.0004**	**-123**	**0.0005**
	GPT-3$_{f(\text{SURP})}$ vs. GPT-2$_{f(\text{SURP})}$	**-190**	**0.0004**	**-162**	**0.0004**	**-199**	**0.0005**
	GPT-3$_{f(\text{SURP})}$ vs. GPT-J$_{f(\text{SURP})}$	**-85**	**0.0004**	**-65**	**0.0004**	**-76**	**0.0005**
	GPT-2PCFG$_{f(\text{SURP})}$ vs. GPT-2$_{f(\text{SURP})}$	-18	—	-2	—	-10	—
GPT-2 (Normal Error)	GPT-2$_{f(\text{SURP})}$ vs. $\emptyset$	**436**	**0.0009**	**469**	**0.0010**	**449**	**0.0422**
	GPT-2$_{f(\text{SURP})}$ vs. GPT-2$_{\text{PROB}}$	**447**	**0.0009**	**367**	**0.0010**	182	1.0000
	GPT-2$_{f(\text{SURP})}$ vs. GPT-2$_{\text{SURP}^{1/2}}$	-54	—	30	1.0000	-156	—
	GPT-2$_{f(\text{SURP})}$ vs. GPT-2$_{\text{SURP}^{3/4}}$	-164	—	-28	—	-158	—
	GPT-2$_{f(\text{SURP})}$ vs. GPT-2$_{\text{SURP}^{1}}$	-62	—	-52	—	26	1.0000
	GPT-2$_{f(\text{SURP})}$ vs. GPT-2$_{\text{SURP}^{4/3}}$	-16	—	-20	—	15	1.0000
	GPT-2$_{f(\text{SURP})}$ vs. GPT-2$_{\text{SURP}^{2}}$	**210**	**0.0016**	**97**	**0.0375**	222	0.9454
	GPT-2$_{\text{PROB}}$ vs. $\emptyset$	-12	—	**102**	**0.0244**	267	0.1212
	GPT-2$_{\text{SURP}^{1/2}}$ vs. $\emptyset$	**489**	**0.0009**	**439**	**0.0010**	**605**	**0.0065**
	GPT-2$_{\text{SURP}^{3/4}}$ vs. $\emptyset$	**600**	**0.0009**	**497**	**0.0010**	**608**	**0.0065**
	GPT-2$_{\text{SURP}^{1}}$ vs. $\emptyset$	**497**	**0.0009**	**521**	**0.0010**	**424**	**0.0467**
	GPT-2$_{\text{SURP}^{4/3}}$ vs. $\emptyset$	**451**	**0.0009**	**489**	**0.0010**	**434**	**0.0422**
	GPT-2$_{\text{SURP}^{2}}$ vs. $\emptyset$	**226**	**0.0036**	**372**	**0.0010**	228	0.6333
	GPT-2$_{\text{SURP}^{1/2}}$ vs. GPT-2$_{\text{PROB}}$	**501**	**0.0009**	**337**	**0.0010**	338	0.0685
	GPT-2$_{\text{SURP}^{3/4}}$ vs. GPT-2$_{\text{PROB}}$	**611**	**0.0009**	**395**	**0.0010**	340	0.0685
	GPT-2$_{\text{SURP}^{1}}$ vs. GPT-2$_{\text{PROB}}$	**509**	**0.0009**	**419**	**0.0010**	156	1.0000
	GPT-2$_{\text{SURP}^{4/3}}$ vs. GPT-2$_{\text{PROB}}$	**463**	**0.0009**	**387**	**0.0010**	167	1.0000
	GPT-2$_{\text{SURP}^{2}}$ vs. GPT-2$_{\text{PROB}}$	**237**	**0.0023**	**270**	**0.0010**	-40	1.0000
	GPT-2$_{\text{SURP}^{3/4}}$ vs. GPT-2$_{\text{SURP}^{1/2}}$	110	0.1993	58	0.8041	2	1.0000
	GPT-2$_{\text{SURP}^{1}}$ vs. GPT-2$_{\text{SURP}^{1/2}}$	8	1.0000	82	0.2370	-182	1.0000
	GPT-2$_{\text{SURP}^{4/3}}$ vs. GPT-2$_{\text{SURP}^{1/2}}$	-38	1.0000	50	1.0000	-171	1.0000
	GPT-2$_{\text{SURP}^{2}}$ vs. GPT-2$_{\text{SURP}^{1/2}}$	**-264**	**0.0009**	-67	0.7154	-378	0.0685
	GPT-2$_{\text{SURP}^{1}}$ vs. GPT-2$_{\text{SURP}^{3/4}}$	-102	0.2143	24	1.0000	-184	1.0000
	GPT-2$_{\text{SURP}^{4/3}}$ vs. GPT-2$_{\text{SURP}^{3/4}}$	**-148**	**0.0224**	-8	1.0000	-174	1.0000
	GPT-2$_{\text{SURP}^{2}}$ vs. GPT-2$_{\text{SURP}^{3/4}}$	**-374**	**0.0009**	**-125**	**0.0156**	-380	0.0685
	GPT-2$_{\text{SURP}^{4/3}}$ vs. GPT-2$_{\text{SURP}^{1}}$	-46	1.0000	-32	1.0000	10	1.0000
	GPT-2$_{\text{SURP}^{2}}$ vs. GPT-2$_{\text{SURP}^{1}}$	**-272**	**0.0009**	**-149**	**0.0010**	-196	1.0000
	GPT-2$_{\text{SURP}^{2}}$ vs. GPT-2$_{\text{SURP}^{4/3}}$	**-226**	**0.0009**	**-117**	**0.0056**	-207	0.9454
	GPT-2$_{\text{SURP}^{<1}}$ vs. GPT-2$_{\text{SURP}^{1}}$	51	1.0000	-29	1.0000	182	1.0000
	GPT-2$_{\text{SURP}^{>1}}$ vs. GPT-2$_{\text{SURP}^{1}}$	**-151**	**0.0149**	**-92**	**0.0162**	-82	1.0000
	GPT-2$_{\text{SURP}^{<1}}$ vs. GPT-2$_{\text{SURP}^{\geq 1}}$	**113**	**0.0167**	47	0.4069	265	0.0685
	GPT-2$_{\text{SURP}^{>1}}$ vs. GPT-2$_{\text{SURP}^{\leq 1}}$	**-179**	**0.0009**	-69	0.0519	-253	0.0685
	GPT-2$^{(\text{EXGAUSS})}_{f(\text{SURP})}$ vs. GPT-2$^{(\text{NORMAL})}_{f(\text{SURP})}$	**14600**	**0.0006**	**10615**	**0.0006**	**25051**	**0.0007**
Prob vs. Surp	GPT-2$_{\text{PROB}+\text{SURP}^{1}}$ vs. GPT-2$_{\text{PROB}}$	**161**	**0.0003**	**131**	**0.0003**	**178**	**0.0003**
	GPT-2$_{\text{PROB}+\text{SURP}^{1}}$ vs. GPT-2$_{\text{SURP}^{1}}$	-24	—	-27	—	-23	—

Table S6. Testing Results on Dundee (Eye-Tracking). Results of key statistical comparisons based on permutation tests of the difference in average test set likelihood between the alternative and null model in each comparison (△LL). Subscripts indicate the assumed functional form of predictability effects: $f(\text{SURP})$ (nonlinear in surprisal), PROB (linear in probability), and SURP^{a} (linear in surprisal raised to exponent a, such that e.g., SURP^{1} is linear in surprisal). Boldface indicates statistical significance. In directional tests where one hypothesis subsumes the other (e.g., $f(\text{SURP})$ vs. SURP^{1}, since a nonlinear surprisal effect subsumes a linear one), dashes (—) indicate failure of the alternative hypothesis (left) to improve over the null hypothesis (right). Otherwise, cells are color coded using cyan to indicate that the hypothesis on the left outperforms the one on the right, and using magenta to indicate that the hypothesis on the right outperforms the one on the left. All p-values are corrected for false discovery rate (15) within each family of tests (delimited by single horizontal lines). Key comparisons are highlighted.

Group	Comparison	First Pass ΔLL	First Pass p	Go Past ΔLL	Go Past p
n-gram	n-gram$_{f(\text{SURP})}$ vs. $\emptyset$	77	0.0010	160	0.0010
	n-gram$_{f(\text{SURP})}$ vs. n-gram$_{\text{PROB}}$	47	0.0010	78	0.0010
	n-gram$_{f(\text{SURP})}$ vs. n-gram$_{\text{SURP}^{1/2}}$	-29	—	-18	—
	n-gram$_{f(\text{SURP})}$ vs. n-gram$_{\text{SURP}^{3/4}}$	-2	—	6	1.0000
	n-gram$_{f(\text{SURP})}$ vs. n-gram$_{\text{SURP}^{1}}$	-27	—	-6	—
	n-gram$_{f(\text{SURP})}$ vs. n-gram$_{\text{SURP}^{4/3}}$	-20	—	3	1.0000
	n-gram$_{f(\text{SURP})}$ vs. n-gram$_{\text{SURP}^{2}}$	-17	—	17	0.8602
	n-gram$_{\text{PROB}}$ vs. $\emptyset$	30	0.0010	82	0.0010
	n-gram$_{\text{SURP}^{1/2}}$ vs. $\emptyset$	105	0.0010	178	0.0010
	n-gram$_{\text{SURP}^{3/4}}$ vs. $\emptyset$	78	0.0010	154	0.0010
	n-gram$_{\text{SURP}^{1}}$ vs. $\emptyset$	103	0.0010	166	0.0010
	n-gram$_{\text{SURP}^{4/3}}$ vs. $\emptyset$	96	0.0010	157	0.0010
	n-gram$_{\text{SURP}^{2}}$ vs. $\emptyset$	94	0.0010	143	0.0010
	n-gram$_{\text{SURP}^{1/2}}$ vs. n-gram$_{\text{PROB}}$	76	0.0010	96	0.0010
	n-gram$_{\text{SURP}^{3/4}}$ vs. n-gram$_{\text{PROB}}$	49	0.0010	72	0.0010
	n-gram$_{\text{SURP}^{1}}$ vs. n-gram$_{\text{PROB}}$	74	0.0010	84	0.0010
	n-gram$_{\text{SURP}^{4/3}}$ vs. n-gram$_{\text{PROB}}$	67	0.0010	75	0.0010
	n-gram$_{\text{SURP}^{2}}$ vs. n-gram$_{\text{PROB}}$	64	0.0010	61	0.0010
	n-gram$_{\text{SURP}^{3/4}}$ vs. n-gram$_{\text{SURP}^{1/2}}$	-27	0.0017	-24	0.1887
	n-gram$_{\text{SURP}^{1}}$ vs. n-gram$_{\text{SURP}^{1/2}}$	-2	1.0000	-12	1.0000
	n-gram$_{\text{SURP}^{4/3}}$ vs. n-gram$_{\text{SURP}^{1/2}}$	-9	0.9908	-21	0.4186
	n-gram$_{\text{SURP}^{2}}$ vs. n-gram$_{\text{SURP}^{1/2}}$	-12	0.6538	-35	0.0186
	n-gram$_{\text{SURP}^{1}}$ vs. n-gram$_{\text{SURP}^{3/4}}$	25	0.0046	12	1.0000
	n-gram$_{\text{SURP}^{4/3}}$ vs. n-gram$_{\text{SURP}^{3/4}}$	18	0.0595	3	1.0000
	n-gram$_{\text{SURP}^{2}}$ vs. n-gram$_{\text{SURP}^{3/4}}$	15	0.1783	-11	1.0000
	n-gram$_{\text{SURP}^{4/3}}$ vs. n-gram$_{\text{SURP}^{1}}$	-7	1.0000	-9	1.0000
	n-gram$_{\text{SURP}^{2}}$ vs. n-gram$_{\text{SURP}^{1}}$	-10	0.9908	-23	0.3344
	n-gram$_{\text{SURP}^{2}}$ vs. n-gram$_{\text{SURP}^{4/3}}$	-3	1.0000	-14	1.0000
	n-gram$_{\text{SURP}^{<1}}$ vs. n-gram$_{\text{SURP}^{1}}$	-16	0.0805	-5	1.0000
	n-gram$_{\text{SURP}^{>1}}$ vs. n-gram$_{\text{SURP}^{1}}$	-9	0.7884	-16	0.6025
	n-gram$_{\text{SURP}^{<1}}$ vs. n-gram$_{\text{SURP}^{\geq 1}}$	-6	0.9173	5	1.0000
	n-gram$_{\text{SURP}^{>1}}$ vs. n-gram$_{\text{SURP}^{\leq 1}}$	3	1.0000	-13	0.4718
PCFG	PCFG$_{f(\text{SURP})}$ vs. $\emptyset$	37	0.0006	97	0.0008
	PCFG$_{f(\text{SURP})}$ vs. PCFG$_{\text{PROB}}$	31	0.0006	89	0.0008
	PCFG$_{f(\text{SURP})}$ vs. PCFG$_{\text{SURP}^{1/2}}$	-55	—	-50	—
	PCFG$_{f(\text{SURP})}$ vs. PCFG$_{\text{SURP}^{3/4}}$	-17	—	-10	—
	PCFG$_{f(\text{SURP})}$ vs. PCFG$_{\text{SURP}^{1}}$	-49	—	-25	—
	PCFG$_{f(\text{SURP})}$ vs. PCFG$_{\text{SURP}^{4/3}}$	-27	—	-7	—
	PCFG$_{f(\text{SURP})}$ vs. PCFG$_{\text{SURP}^{2}}$	24	0.0051	29	0.0463
	PCFG$_{\text{PROB}}$ vs. $\emptyset$	7	1.0000	9	1.0000
	PCFG$_{\text{SURP}^{1/2}}$ vs. $\emptyset$	92	0.0006	147	0.0008
	PCFG$_{\text{SURP}^{3/4}}$ vs. $\emptyset$	54	0.0006	108	0.0008
	PCFG$_{\text{SURP}^{1}}$ vs. $\emptyset$	87	0.0006	122	0.0008
	PCFG$_{\text{SURP}^{4/3}}$ vs. $\emptyset$	65	0.0006	104	0.0008
	PCFG$_{\text{SURP}^{2}}$ vs. $\emptyset$	13	0.3279	69	0.0008
	PCFG$_{\text{SURP}^{1/2}}$ vs. PCFG$_{\text{PROB}}$	86	0.0006	138	0.0008
	PCFG$_{\text{SURP}^{3/4}}$ vs. PCFG$_{\text{PROB}}$	47	0.0006	99	0.0008
	PCFG$_{\text{SURP}^{1}}$ vs. PCFG$_{\text{PROB}}$	80	0.0006	114	0.0008
	PCFG$_{\text{SURP}^{4/3}}$ vs. PCFG$_{\text{PROB}}$	58	0.0006	95	0.0008
	PCFG$_{\text{SURP}^{2}}$ vs. PCFG$_{\text{PROB}}$	7	1.0000	60	0.0008
	PCFG$_{\text{SURP}^{3/4}}$ vs. PCFG$_{\text{SURP}^{1/2}}$	-38	0.0006	-39	0.0087
	PCFG$_{\text{SURP}^{1}}$ vs. PCFG$_{\text{SURP}^{1/2}}$	-5	1.0000	-25	0.1293
	PCFG$_{\text{SURP}^{4/3}}$ vs. PCFG$_{\text{SURP}^{1/2}}$	-27	0.0006	-43	0.0021
	PCFG$_{\text{SURP}^{2}}$ vs. PCFG$_{\text{SURP}^{1/2}}$	-79	0.0006	-78	0.0008
	PCFG$_{\text{SURP}^{1}}$ vs. PCFG$_{\text{SURP}^{3/4}}$	33	0.0006	15	0.8369
	PCFG$_{\text{SURP}^{4/3}}$ vs. PCFG$_{\text{SURP}^{3/4}}$	11	0.4948	-4	1.0000
	PCFG$_{\text{SURP}^{2}}$ vs. PCFG$_{\text{SURP}^{3/4}}$	-41	0.0006	-39	0.0039
	PCFG$_{\text{SURP}^{4/3}}$ vs. PCFG$_{\text{SURP}^{1}}$	-22	0.0073	-18	0.4805
	PCFG$_{\text{SURP}^{2}}$ vs. PCFG$_{\text{SURP}^{1}}$	-74	0.0006	-54	0.0008
	PCFG$_{\text{SURP}^{2}}$ vs. PCFG$_{\text{SURP}^{4/3}}$	-52	0.0006	-35	0.0106
	PCFG$_{\text{SURP}^{<1}}$ vs. PCFG$_{\text{SURP}^{1}}$	-18	0.0140	1	1.0000
	PCFG$_{\text{SURP}^{>1}}$ vs. PCFG$_{\text{SURP}^{1}}$	-40	0.0006	-39	0.0008
	PCFG$_{\text{SURP}^{<1}}$ vs. PCFG$_{\text{SURP}^{\geq 1}}$	15	0.0070	32	0.0014
	PCFG$_{\text{SURP}^{>1}}$ vs. PCFG$_{\text{SURP}^{\leq 1}}$	-25	0.0006	-39	0.0008
GPT-2	GPT-2$_{f(\text{SURP})}$ vs. $\emptyset$	246	0.0007	395	0.0006
	GPT-2$_{f(\text{SURP})}$ vs. GPT-2$_{\text{PROB}}$	129	0.0007	217	0.0006
	GPT-2$_{f(\text{SURP})}$ vs. GPT-2$_{\text{SURP}^{1/2}}$	18	0.0675	39	0.0076
	GPT-2$_{f(\text{SURP})}$ vs. GPT-2$_{\text{SURP}^{3/4}}$	-28	—	14	1.0000
	GPT-2$_{f(\text{SURP})}$ vs. GPT-2$_{\text{SURP}^{1}}$	-16	—	-17	—
	GPT-2$_{f(\text{SURP})}$ vs. GPT-2$_{\text{SURP}^{4/3}}$	-1	—	-8	—
	GPT-2$_{f(\text{SURP})}$ vs. GPT-2$_{\text{SURP}^{2}}$	30	0.0026	63	0.0006
	GPT-2$_{\text{PROB}}$ vs. $\emptyset$	117	0.0007	178	0.0006
	GPT-2$_{\text{SURP}^{1/2}}$ vs. $\emptyset$	228	0.0007	356	0.0006
	GPT-2$_{\text{SURP}^{3/4}}$ vs. $\emptyset$	274	0.0007	382	0.0006
	GPT-2$_{\text{SURP}^{1}}$ vs. $\emptyset$	261	0.0007	412	0.0006
	GPT-2$_{\text{SURP}^{4/3}}$ vs. $\emptyset$	247	0.0007	403	0.0006
	GPT-2$_{\text{SURP}^{2}}$ vs. $\emptyset$	216	0.0007	332	0.0006
	GPT-2$_{\text{SURP}^{1/2}}$ vs. GPT-2$_{\text{PROB}}$	111	0.0007	178	0.0006
	GPT-2$_{\text{SURP}^{3/4}}$ vs. GPT-2$_{\text{PROB}}$	157	0.0007	203	0.0006
	GPT-2$_{\text{SURP}^{1}}$ vs. GPT-2$_{\text{PROB}}$	145	0.0007	234	0.0006
	GPT-2$_{\text{SURP}^{4/3}}$ vs. GPT-2$_{\text{PROB}}$	130	0.0007	225	0.0006
	GPT-2$_{\text{SURP}^{2}}$ vs. GPT-2$_{\text{PROB}}$	99	0.0007	154	0.0006
	GPT-2$_{\text{SURP}^{3/4}}$ vs. GPT-2$_{\text{SURP}^{1/2}}$	46	0.0007	25	0.1245
	GPT-2$_{\text{SURP}^{1}}$ vs. GPT-2$_{\text{SURP}^{1/2}}$	34	0.0007	56	0.0006
	GPT-2$_{\text{SURP}^{4/3}}$ vs. GPT-2$_{\text{SURP}^{1/2}}$	19	0.0730	47	0.0006
	GPT-2$_{\text{SURP}^{2}}$ vs. GPT-2$_{\text{SURP}^{1/2}}$	-12	0.5423	-24	0.2548
	GPT-2$_{\text{SURP}^{1}}$ vs. GPT-2$_{\text{SURP}^{3/4}}$	-12	0.3395	30	0.0489
	GPT-2$_{\text{SURP}^{4/3}}$ vs. GPT-2$_{\text{SURP}^{3/4}}$	-27	0.0035	22	0.3079
	GPT-2$_{\text{SURP}^{2}}$ vs. GPT-2$_{\text{SURP}^{3/4}}$	-58	0.0007	-49	0.0006
	GPT-2$_{\text{SURP}^{4/3}}$ vs. GPT-2$_{\text{SURP}^{1}}$	-15	0.1814	-8	1.0000
	GPT-2$_{\text{SURP}^{2}}$ vs. GPT-2$_{\text{SURP}^{1}}$	-46	0.0007	-80	0.0006
	GPT-2$_{\text{SURP}^{2}}$ vs. GPT-2$_{\text{SURP}^{4/3}}$	-31	0.0007	-71	0.0006
	GPT-2$_{\text{SURP}^{<1}}$ vs. GPT-2$_{\text{SURP}^{1}}$	-2	1.0000	-34	0.0031
	GPT-2$_{\text{SURP}^{>1}}$ vs. GPT-2$_{\text{SURP}^{1}}$	-18	0.0236	-39	0.0006
	GPT-2$_{\text{SURP}^{<1}}$ vs. GPT-2$_{\text{SURP}^{\geq 1}}$	13	0.0518	-19	0.0788
	GPT-2$_{\text{SURP}^{>1}}$ vs. GPT-2$_{\text{SURP}^{\leq 1}}$	-18	0.0031	-11	0.7317
GPT-J	GPT-J$_{f(\text{SURP})}$ vs. $\emptyset$	192	0.0009	315	0.0009
	GPT-J$_{f(\text{SURP})}$ vs. GPT-J$_{\text{PROB}}$	76	0.0009	174	0.0009
	GPT-J$_{f(\text{SURP})}$ vs. GPT-J$_{\text{SURP}^{1/2}}$	14	0.3733	28	0.0949
	GPT-J$_{f(\text{SURP})}$ vs. GPT-J$_{\text{SURP}^{3/4}}$	17	0.1839	24	0.1883
	GPT-J$_{f(\text{SURP})}$ vs. GPT-J$_{\text{SURP}^{1}}$	9	1.0000	-9	—
	GPT-J$_{f(\text{SURP})}$ vs. GPT-J$_{\text{SURP}^{4/3}}$	11	0.9291	-13	—
	GPT-J$_{f(\text{SURP})}$ vs. GPT-J$_{\text{SURP}^{2}}$	38	0.0009	41	0.0110
	GPT-J$_{\text{PROB}}$ vs. $\emptyset$	117	0.0009	141	0.0009
	GPT-J$_{\text{SURP}^{1/2}}$ vs. $\emptyset$	178	0.0009	287	0.0009
	GPT-J$_{\text{SURP}^{3/4}}$ vs. $\emptyset$	176	0.0009	290	0.0009
	GPT-J$_{\text{SURP}^{1}}$ vs. $\emptyset$	183	0.0009	324	0.0009
	GPT-J$_{\text{SURP}^{4/3}}$ vs. $\emptyset$	182	0.0009	328	0.0009
	GPT-J$_{\text{SURP}^{2}}$ vs. $\emptyset$	154	0.0009	274	0.0009
	GPT-J$_{\text{SURP}^{1/2}}$ vs. GPT-J$_{\text{PROB}}$	61	0.0009	146	0.0009
	GPT-J$_{\text{SURP}^{3/4}}$ vs. GPT-J$_{\text{PROB}}$	59	0.0009	150	0.0009
	GPT-J$_{\text{SURP}^{1}}$ vs. GPT-J$_{\text{PROB}}$	67	0.0009	183	0.0009
	GPT-J$_{\text{SURP}^{4/3}}$ vs. GPT-J$_{\text{PROB}}$	65	0.0009	187	0.0009
	GPT-J$_{\text{SURP}^{2}}$ vs. GPT-J$_{\text{PROB}}$	38	0.0009	133	0.0009
	GPT-J$_{\text{SURP}^{3/4}}$ vs. GPT-J$_{\text{SURP}^{1/2}}$	-2	1.0000	4	1.0000
	GPT-J$_{\text{SURP}^{1}}$ vs. GPT-J$_{\text{SURP}^{1/2}}$	5	1.0000	37	0.0124
	GPT-J$_{\text{SURP}^{4/3}}$ vs. GPT-J$_{\text{SURP}^{1/2}}$	4	1.0000	41	0.0041
	GPT-J$_{\text{SURP}^{2}}$ vs. GPT-J$_{\text{SURP}^{1/2}}$	-24	0.0244	-12	1.0000
	GPT-J$_{\text{SURP}^{1}}$ vs. GPT-J$_{\text{SURP}^{3/4}}$	8	1.0000	33	0.0266
	GPT-J$_{\text{SURP}^{4/3}}$ vs. GPT-J$_{\text{SURP}^{3/4}}$	6	1.0000	38	0.0082
	GPT-J$_{\text{SURP}^{2}}$ vs. GPT-J$_{\text{SURP}^{3/4}}$	-22	0.0447	-16	1.0000
	GPT-J$_{\text{SURP}^{4/3}}$ vs. GPT-J$_{\text{SURP}^{1}}$	-2	1.0000	4	1.0000
	GPT-J$_{\text{SURP}^{2}}$ vs. GPT-J$_{\text{SURP}^{1}}$	-29	0.0009	-49	0.0017
	GPT-J$_{\text{SURP}^{2}}$ vs. GPT-J$_{\text{SURP}^{4/3}}$	-28	0.0016	-54	0.0009
	GPT-J$_{\text{SURP}^{<1}}$ vs. GPT-J$_{\text{SURP}^{1}}$	-5	1.0000	-36	0.0053
	GPT-J$_{\text{SURP}^{>1}}$ vs. GPT-J$_{\text{SURP}^{1}}$	-11	0.5714	-15	0.7558
	GPT-J$_{\text{SURP}^{<1}}$ vs. GPT-J$_{\text{SURP}^{\geq 1}}$	5	1.0000	-28	0.0072
	GPT-J$_{\text{SURP}^{>1}}$ vs. GPT-J$_{\text{SURP}^{\leq 1}}$	-6	1.0000	-1	1.0000
GPT-3	GPT-3$_{f(\text{SURP})}$ vs. $\emptyset$	141	0.0008	220	0.0011
	GPT-3$_{f(\text{SURP})}$ vs. GPT-3$_{\text{PROB}}$	6	1.0000	77	0.0011
	GPT-3$_{f(\text{SURP})}$ vs. GPT-3$_{\text{SURP}^{1/2}}$	15	0.3444	31	0.0623
	GPT-3$_{f(\text{SURP})}$ vs. GPT-3$_{\text{SURP}^{3/4}}$	-4	—	19	0.5887
	GPT-3$_{f(\text{SURP})}$ vs. GPT-3$_{\text{SURP}^{1}}$	41	0.0008	33	0.0355
	GPT-3$_{f(\text{SURP})}$ vs. GPT-3$_{\text{SURP}^{4/3}}$	-5	—	-2	—
	GPT-3$_{f(\text{SURP})}$ vs. GPT-3$_{\text{SURP}^{2}}$	34	0.0008	19	0.5887
	GPT-3$_{\text{PROB}}$ vs. $\emptyset$	136	0.0008	143	0.0011
	GPT-3$_{\text{SURP}^{1/2}}$ vs. $\emptyset$	127	0.0008	189	0.0011
	GPT-3$_{\text{SURP}^{3/4}}$ vs. $\emptyset$	145	0.0008	201	0.0011
	GPT-3$_{\text{SURP}^{1}}$ vs. $\emptyset$	100	0.0008	187	0.0011
	GPT-3$_{\text{SURP}^{4/3}}$ vs. $\emptyset$	147	0.0008	223	0.0011
	GPT-3$_{\text{SURP}^{2}}$ vs. $\emptyset$	107	0.0008	201	0.0011
	GPT-3$_{\text{SURP}^{1/2}}$ vs. GPT-3$_{\text{PROB}}$	-9	1.0000	46	0.0011
	GPT-3$_{\text{SURP}^{3/4}}$ vs. GPT-3$_{\text{PROB}}$	10	1.0000	58	0.0011
	GPT-3$_{\text{SURP}^{1}}$ vs. GPT-3$_{\text{PROB}}$	-36	0.0008	44	0.0028
	GPT-3$_{\text{SURP}^{4/3}}$ vs. GPT-3$_{\text{PROB}}$	11	0.9723	80	0.0011
	GPT-3$_{\text{SURP}^{2}}$ vs. GPT-3$_{\text{PROB}}$	-28	0.0043	58	0.0011
	GPT-3$_{\text{SURP}^{3/4}}$ vs. GPT-3$_{\text{SURP}^{1/2}}$	18	0.0773	12	1.0000
	GPT-3$_{\text{SURP}^{1}}$ vs. GPT-3$_{\text{SURP}^{1/2}}$	-27	0.0031	-1	1.0000
	GPT-3$_{\text{SURP}^{4/3}}$ vs. GPT-3$_{\text{SURP}^{1/2}}$	20	0.0500	34	0.0281
	GPT-3$_{\text{SURP}^{2}}$ vs. GPT-3$_{\text{SURP}^{1/2}}$	-20	0.0773	12	1.0000
	GPT-3$_{\text{SURP}^{1}}$ vs. GPT-3$_{\text{SURP}^{3/4}}$	-45	0.0008	-13	1.0000
	GPT-3$_{\text{SURP}^{4/3}}$ vs. GPT-3$_{\text{SURP}^{3/4}}$	1	1.0000	22	0.3795
	GPT-3$_{\text{SURP}^{2}}$ vs. GPT-3$_{\text{SURP}^{3/4}}$	-38	0.0008	0	1.0000
	GPT-3$_{\text{SURP}^{4/3}}$ vs. GPT-3$_{\text{SURP}^{1}}$	47	0.0008	35	0.0187
	GPT-3$_{\text{SURP}^{2}}$ vs. GPT-3$_{\text{SURP}^{1}}$	7	1.0000	14	1.0000
	GPT-3$_{\text{SURP}^{2}}$ vs. GPT-3$_{\text{SURP}^{4/3}}$	-39	0.0008	-22	0.3795
	GPT-3$_{\text{SURP}^{<1}}$ vs. GPT-3$_{\text{SURP}^{1}}$	41	0.0008	4	1.0000
	GPT-3$_{\text{SURP}^{>1}}$ vs. GPT-3$_{\text{SURP}^{1}}$	28	0.0008	32	0.0121
	GPT-3$_{\text{SURP}^{<1}}$ vs. GPT-3$_{\text{SURP}^{\geq 1}}$	16	0.0116	-24	0.0214
	GPT-3$_{\text{SURP}^{>1}}$ vs. GPT-3$_{\text{SURP}^{\leq 1}}$	0	1.0000	28	0.0020
All LMs	All-LMs$_{f(\text{SURP})}$ vs. $\emptyset$	141	0.0006	220	0.0008
	All-LMs$_{f(\text{SURP})}$ vs. All-LMs$_{\text{PROB}}$	62	0.0006	110	0.0008
	All-LMs$_{f(\text{SURP})}$ vs. All-LMs$_{\text{SURP}^{1/2}}$	-11	—	16	0.1217
	All-LMs$_{f(\text{SURP})}$ vs. All-LMs$_{\text{SURP}^{3/4}}$	1	1.0000	32	0.0008
	All-LMs$_{f(\text{SURP})}$ vs. All-LMs$_{\text{SURP}^{1}}$	10	0.1443	19	0.0542
	All-LMs$_{f(\text{SURP})}$ vs. All-LMs$_{\text{SURP}^{4/3}}$	-6	—	-3	—
	All-LMs$_{f(\text{SURP})}$ vs. All-LMs$_{\text{SURP}^{2}}$	28	0.0006	19	0.0484
	All-LMs$_{\text{PROB}}$ vs. $\emptyset$	80	0.0006	111	0.0008
	All-LMs$_{\text{SURP}^{1/2}}$ vs. $\emptyset$	152	0.0006	204	0.0008
	All-LMs$_{\text{SURP}^{3/4}}$ vs. $\emptyset$	141	0.0006	188	0.0008
	All-LMs$_{\text{SURP}^{1}}$ vs. $\emptyset$	132	0.0006	202	0.0008
	All-LMs$_{\text{SURP}^{4/3}}$ vs. $\emptyset$	148	0.0006	223	0.0008
	All-LMs$_{\text{SURP}^{2}}$ vs. $\emptyset$	113	0.0006	201	0.0008
	All-LMs$_{\text{SURP}^{1/2}}$ vs. All-LMs$_{\text{PROB}}$	73	0.0006	93	0.0008
	All-LMs$_{\text{SURP}^{3/4}}$ vs. All-LMs$_{\text{PROB}}$	61	0.0006	78	0.0008
	All-LMs$_{\text{SURP}^{1}}$ vs. All-LMs$_{\text{PROB}}$	52	0.0006	91	0.0008
	All-LMs$_{\text{SURP}^{4/3}}$ vs. All-LMs$_{\text{PROB}}$	68	0.0006	112	0.0008
	All-LMs$_{\text{SURP}^{2}}$ vs. All-LMs$_{\text{PROB}}$	33	0.0006	90	0.0008
	All-LMs$_{\text{SURP}^{3/4}}$ vs. All-LMs$_{\text{SURP}^{1/2}}$	-12	0.0287	-15	0.1376
	All-LMs$_{\text{SURP}^{1}}$ vs. All-LMs$_{\text{SURP}^{1/2}}$	-21	0.0006	-2	1.0000
	All-LMs$_{\text{SURP}^{4/3}}$ vs. All-LMs$_{\text{SURP}^{1/2}}$	-5	1.0000	19	0.0439
	All-LMs$_{\text{SURP}^{2}}$ vs. All-LMs$_{\text{SURP}^{1/2}}$	-39	0.0006	-3	1.0000
	All-LMs$_{\text{SURP}^{1}}$ vs. All-LMs$_{\text{SURP}^{3/4}}$	-9	0.1895	13	0.2902
	All-LMs$_{\text{SURP}^{4/3}}$ vs. All-LMs$_{\text{SURP}^{3/4}}$	7	0.4540	35	0.0008
	All-LMs$_{\text{SURP}^{2}}$ vs. All-LMs$_{\text{SURP}^{3/4}}$	-27	0.0006	13	0.3564
	All-LMs$_{\text{SURP}^{4/3}}$ vs. All-LMs$_{\text{SURP}^{1}}$	16	0.0018	21	0.0212
	All-LMs$_{\text{SURP}^{2}}$ vs. All-LMs$_{\text{SURP}^{1}}$	-19	0.0006	0	1.0000
	All-LMs$_{\text{SURP}^{2}}$ vs. All-LMs$_{\text{SURP}^{4/3}}$	-35	0.0006	-22	0.0152
	All-LMs$_{\text{SURP}^{<1}}$ vs. All-LMs$_{\text{SURP}^{1}}$	14	0.0012	-5	1.0000
	All-LMs$_{\text{SURP}^{>1}}$ vs. All-LMs$_{\text{SURP}^{1}}$	2	1.0000	18	0.0286
	All-LMs$_{\text{SURP}^{<1}}$ vs. All-LMs$_{\text{SURP}^{\geq 1}}$	14	0.0006	-21	0.0015
	All-LMs$_{\text{SURP}^{>1}}$ vs. All-LMs$_{\text{SURP}^{\leq 1}}$	-8	0.0220	22	0.0008

Group	Comparison	First Pass ΔLL	First Pass p	Go Past ΔLL	Go Past p
Between LMs	PCFG$_{f(\text{SURP})}$ vs. n-gram$_{f(\text{SURP})}$	-39	0.0003	-63	0.0003
	GPT-2$_{f(\text{SURP})}$ vs. n-gram$_{f(\text{SURP})}$	169	0.0003	235	0.0003
	GPT-J$_{f(\text{SURP})}$ vs. n-gram$_{f(\text{SURP})}$	116	0.0003	155	0.0003
	GPT-3$_{f(\text{SURP})}$ vs. n-gram$_{f(\text{SURP})}$	65	0.0003	60	0.0003
	GPT-2$_{f(\text{SURP})}$ vs. PCFG$_{f(\text{SURP})}$	208	0.0003	298	0.0003
	GPT-J$_{f(\text{SURP})}$ vs. PCFG$_{f(\text{SURP})}$	155	0.0003	217	0.0003
	GPT-3$_{f(\text{SURP})}$ vs. PCFG$_{f(\text{SURP})}$	104	0.0003	123	0.0003
	GPT-J$_{f(\text{SURP})}$ vs. GPT-2$_{f(\text{SURP})}$	-54	0.0003	-80	0.0003
	GPT-3$_{f(\text{SURP})}$ vs. GPT-2$_{f(\text{SURP})}$	-104	0.0003	-175	0.0003
	GPT-3$_{f(\text{SURP})}$ vs. GPT-J$_{f(\text{SURP})}$	-51	0.0003	-95	0.0003
	GPT-2PCFG$_{f(\text{SURP})}$ vs. GPT-2$_{f(\text{SURP})}$	-21	—	-79	—
GPT-2 (Normal Error)	GPT-2$_{f(\text{SURP})}$ vs. $\emptyset$	480	0.0005	607	0.0032
	GPT-2$_{f(\text{SURP})}$ vs. GPT-2$_{\text{PROB}}$	312	0.0005	178	0.9496
	GPT-2$_{f(\text{SURP})}$ vs. GPT-2$_{\text{SURP}^{1/2}}$	-27	—	-251	—
	GPT-2$_{f(\text{SURP})}$ vs. GPT-2$_{\text{SURP}^{3/4}}$	-32	—	-196	—
	GPT-2$_{f(\text{SURP})}$ vs. GPT-2$_{\text{SURP}^{1}}$	5	1.0000	-262	—
	GPT-2$_{f(\text{SURP})}$ vs. GPT-2$_{\text{SURP}^{4/3}}$	115	0.0005	110	1.0000
	GPT-2$_{f(\text{SURP})}$ vs. GPT-2$_{\text{SURP}^{2}}$	126	0.0005	127	1.0000
	GPT-2$_{\text{PROB}}$ vs. $\emptyset$	168	0.0005	430	0.0299
	GPT-2$_{\text{SURP}^{1/2}}$ vs. $\emptyset$	507	0.0005	858	0.0032
	GPT-2$_{\text{SURP}^{3/4}}$ vs. $\emptyset$	512	0.0005	803	0.0032
	GPT-2$_{\text{SURP}^{1}}$ vs. $\emptyset$	475	0.0005	869	0.0032
	GPT-2$_{\text{SURP}^{4/3}}$ vs. $\emptyset$	365	0.0005	497	0.0065
	GPT-2$_{\text{SURP}^{2}}$ vs. $\emptyset$	354	0.0005	480	0.0065
	GPT-2$_{\text{SURP}^{1/2}}$ vs. GPT-2$_{\text{PROB}}$	340	0.0005	429	0.0065
	GPT-2$_{\text{SURP}^{3/4}}$ vs. GPT-2$_{\text{PROB}}$	345	0.0005	374	0.0460
	GPT-2$_{\text{SURP}^{1}}$ vs. GPT-2$_{\text{PROB}}$	307	0.0005	439	0.0065
	GPT-2$_{\text{SURP}^{4/3}}$ vs. GPT-2$_{\text{PROB}}$	197	0.0005	67	1.0000
	GPT-2$_{\text{SURP}^{2}}$ vs. GPT-2$_{\text{PROB}}$	186	0.0005	50	1.0000
	GPT-2$_{\text{SURP}^{3/4}}$ vs. GPT-2$_{\text{SURP}^{1/2}}$	5	1.0000	-55	1.0000
	GPT-2$_{\text{SURP}^{1}}$ vs. GPT-2$_{\text{SURP}^{1/2}}$	-33	0.5570	11	1.0000
	GPT-2$_{\text{SURP}^{4/3}}$ vs. GPT-2$_{\text{SURP}^{1/2}}$	-142	0.0005	-361	0.1731
	GPT-2$_{\text{SURP}^{2}}$ vs. GPT-2$_{\text{SURP}^{1/2}}$	-154	0.0005	-378	0.2292
	GPT-2$_{\text{SURP}^{1}}$ vs. GPT-2$_{\text{SURP}^{3/4}}$	-37	0.3596	66	1.0000
	GPT-2$_{\text{SURP}^{4/3}}$ vs. GPT-2$_{\text{SURP}^{3/4}}$	-147	0.0005	-306	0.2496
	GPT-2$_{\text{SURP}^{2}}$ vs. GPT-2$_{\text{SURP}^{3/4}}$	-158	0.0005	-323	0.3201
	GPT-2$_{\text{SURP}^{4/3}}$ vs. GPT-2$_{\text{SURP}^{1}}$	-110	0.0005	-372	0.1029
	GPT-2$_{\text{SURP}^{2}}$ vs. GPT-2$_{\text{SURP}^{1}}$	-121	0.0005	-389	0.1391
	GPT-2$_{\text{SURP}^{2}}$ vs. GPT-2$_{\text{SURP}^{4/3}}$	-11	1.0000	-17	1.0000
	GPT-2$_{\text{SURP}^{<1}}$ vs. GPT-2$_{\text{SURP}^{1}}$	34	0.3231	-11	1.0000
	GPT-2$_{\text{SURP}^{>1}}$ vs. GPT-2$_{\text{SURP}^{1}}$	-111	0.0005	-372	0.0487
	GPT-2$_{\text{SURP}^{<1}}$ vs. GPT-2$_{\text{SURP}^{\geq 1}}$	144	0.0005	234	0.2118
	GPT-2$_{\text{SURP}^{>1}}$ vs. GPT-2$_{\text{SURP}^{\leq 1}}$	-145	0.0005	-361	0.0072
	GPT-2$^{(\text{EXGAUSS})}_{f(\text{SURP})}$ vs. GPT-2$^{(\text{NORMAL})}_{f(\text{SURP})}$	13461	0.0007	40958	0.0006
Prob vs. Surp	GPT-2$_{\text{PROB}+\text{SURP}^{1}}$ vs. GPT-2$_{\text{PROB}}$	143	0.0003	241	0.0003
	GPT-2$_{\text{PROB}+\text{SURP}^{1}}$ vs. GPT-2$_{\text{SURP}^{1}}$	-2	—	7	0.8030

Table S7. Testing Results on GECO (Eye-Tracking). Results of key statistical comparisons based on permutation tests of the difference in average test set likelihood between the alternative and null model in each comparison (△LL). Subscripts indicate the assumed functional form of predictability effects: $f(\text{SURP})$ (nonlinear in surprisal), PROB (linear in probability), and SURP^{a} (linear in surprisal raised to exponent a, such that e.g., SURP^{1} is linear in surprisal). Boldface indicates statistical significance. In directional tests where one hypothesis subsumes the other (e.g., $f(\text{SURP})$ vs. SURP^{1}, since a nonlinear surprisal effect subsumes a linear one), dashes (—) indicate failure of the alternative hypothesis (left) to improve over the null hypothesis (right). Otherwise, cells are color coded using cyan to indicate that the hypothesis on the left outperforms the one on the right, and using magenta to indicate that the hypothesis on the right outperforms the one on the left. All p-values are corrected for false discovery rate (15) within each family of tests (delimited by single horizontal lines). Key comparisons are highlighted.

Group	Comparison	Scan Path ΔLL	Scan Path p	First Pass ΔLL	First Pass p	Go Past ΔLL	Go Past p
n-gram	n-gram$_{f(\text{SURP})}$ vs. $\emptyset$	-21	—	-7	—	**42**	**0.0013**
	n-gram$_{f(\text{SURP})}$ vs. n-gram$_{\text{PROB}}$	-6	—	8	1.0000	**51**	**0.0013**
	n-gram$_{f(\text{SURP})}$ vs. n-gram$_{\text{SURP}^{1/2}}$	-15	—	-4	—	-1	—
	n-gram$_{f(\text{SURP})}$ vs. n-gram$_{\text{SURP}^{3/4}}$	-11	—	-9	—	-6	—
	n-gram$_{f(\text{SURP})}$ vs. n-gram$_{\text{SURP}^{1}}$	-18	—	1	1.0000	12	0.4250
	n-gram$_{f(\text{SURP})}$ vs. n-gram$_{\text{SURP}^{4/3}}$	-2	—	2	1.0000	15	0.2065
	n-gram$_{f(\text{SURP})}$ vs. n-gram$_{\text{SURP}^{2}}$	-13	—	3	1.0000	**28**	**0.0022**
	n-gram$_{\text{PROB}}$ vs. $\emptyset$	-15	—	-16	—	-9	—
	n-gram$_{\text{SURP}^{1/2}}$ vs. $\emptyset$	-6	—	-3	—	**43**	**0.0013**
	n-gram$_{\text{SURP}^{3/4}}$ vs. $\emptyset$	-10	—	1	1.0000	**48**	**0.0013**
	n-gram$_{\text{SURP}^{1}}$ vs. $\emptyset$	-3	—	-8	—	**30**	**0.0050**
	n-gram$_{\text{SURP}^{4/3}}$ vs. $\emptyset$	-18	—	-9	—	**27**	**0.0065**
	n-gram$_{\text{SURP}^{2}}$ vs. $\emptyset$	-8	—	-11	—	14	0.3750
	n-gram$_{\text{SURP}^{1/2}}$ vs. n-gram$_{\text{PROB}}$	9	1.0000	13	0.4740	**52**	**0.0013**
	n-gram$_{\text{SURP}^{3/4}}$ vs. n-gram$_{\text{PROB}}$	5	1.0000	17	0.1039	**57**	**0.0013**
	n-gram$_{\text{SURP}^{1}}$ vs. n-gram$_{\text{PROB}}$	13	0.6522	7	1.0000	**38**	**0.0013**
	n-gram$_{\text{SURP}^{4/3}}$ vs. n-gram$_{\text{PROB}}$	-3	1.0000	7	1.0000	**36**	**0.0013**
	n-gram$_{\text{SURP}^{2}}$ vs. n-gram$_{\text{PROB}}$	7	1.0000	5	1.0000	**23**	**0.0462**
	n-gram$_{\text{SURP}^{3/4}}$ vs. n-gram$_{\text{SURP}^{1/2}}$	-4	1.0000	4	1.0000	5	1.0000
	n-gram$_{\text{SURP}^{1}}$ vs. n-gram$_{\text{SURP}^{1/2}}$	4	1.0000	-5	1.0000	-14	0.3032
	n-gram$_{\text{SURP}^{4/3}}$ vs. n-gram$_{\text{SURP}^{1/2}}$	-12	0.5779	-6	1.0000	-16	0.1325
	n-gram$_{\text{SURP}^{2}}$ vs. n-gram$_{\text{SURP}^{1/2}}$	-2	1.0000	-8	1.0000	**-29**	**0.0022**
	n-gram$_{\text{SURP}^{1}}$ vs. n-gram$_{\text{SURP}^{3/4}}$	7	1.0000	-9	0.6161	-19	0.0595
	n-gram$_{\text{SURP}^{4/3}}$ vs. n-gram$_{\text{SURP}^{3/4}}$	-9	1.0000	-10	0.5649	**-21**	**0.0190**
	n-gram$_{\text{SURP}^{2}}$ vs. n-gram$_{\text{SURP}^{3/4}}$	2	1.0000	-12	0.4740	**-34**	**0.0013**
	n-gram$_{\text{SURP}^{4/3}}$ vs. n-gram$_{\text{SURP}^{1}}$	-16	0.2110	-1	1.0000	-3	1.0000
	n-gram$_{\text{SURP}^{2}}$ vs. n-gram$_{\text{SURP}^{1}}$	-5	1.0000	-3	1.0000	-15	0.2739
	n-gram$_{\text{SURP}^{2}}$ vs. n-gram$_{\text{SURP}^{4/3}}$	10	0.7957	-2	1.0000	-13	0.3750
	n-gram$_{\text{SURP}^{<1}}$ vs. n-gram$_{\text{SURP}^{1}}$	-6	1.0000	7	1.0000	**18**	**0.0306**
	n-gram$_{\text{SURP}^{>1}}$ vs. n-gram$_{\text{SURP}^{1}}$	-8	1.0000	-2	1.0000	-7	1.0000
	n-gram$_{\text{SURP}^{<1}}$ vs. n-gram$_{\text{SURP}^{\geq 1}}$	3	1.0000	7	0.5432	**23**	**0.0013**
	n-gram$_{\text{SURP}^{>1}}$ vs. n-gram$_{\text{SURP}^{\leq 1}}$	-3	1.0000	-7	0.5432	**-15**	**0.0219**
PCFG	PCFG$_{f(\text{SURP})}$ vs. $\emptyset$	-5	—	9	0.4533	10	1.0000
	PCFG$_{f(\text{SURP})}$ vs. PCFG$_{\text{PROB}}$	**42**	**0.0012**	**47**	**0.0016**	26	0.0909
	PCFG$_{f(\text{SURP})}$ vs. PCFG$_{\text{SURP}^{1/2}}$	**21**	**0.0061**	**21**	**0.0016**	14	0.8311
	PCFG$_{f(\text{SURP})}$ vs. PCFG$_{\text{SURP}^{3/4}}$	11	0.3099	**16**	**0.0071**	9	1.0000
	PCFG$_{f(\text{SURP})}$ vs. PCFG$_{\text{SURP}^{1}}$	-4	—	7	0.8787	16	0.6341
	PCFG$_{f(\text{SURP})}$ vs. PCFG$_{\text{SURP}^{4/3}}$	**28**	**0.0012**	**19**	**0.0039**	25	0.0909
	PCFG$_{f(\text{SURP})}$ vs. PCFG$_{\text{SURP}^{2}}$	4	1.0000	4	1.0000	12	1.0000
	PCFG$_{\text{PROB}}$ vs. $\emptyset$	-46	—	-38	—	-16	—
	PCFG$_{\text{SURP}^{1/2}}$ vs. $\emptyset$	-25	—	-13	—	-3	—
	PCFG$_{\text{SURP}^{3/4}}$ vs. $\emptyset$	-15	—	-7	—	2	1.0000
	PCFG$_{\text{SURP}^{1}}$ vs. $\emptyset$	-1	—	2	1.0000	-5	—
	PCFG$_{\text{SURP}^{4/3}}$ vs. $\emptyset$	-33	—	-11	—	-14	—
	PCFG$_{\text{SURP}^{2}}$ vs. $\emptyset$	-9	—	5	1.0000	-1	—
	PCFG$_{\text{SURP}^{1/2}}$ vs. PCFG$_{\text{PROB}}$	**21**	**0.0056**	**25**	**0.0016**	13	0.9856
	PCFG$_{\text{SURP}^{3/4}}$ vs. PCFG$_{\text{PROB}}$	**31**	**0.0012**	**31**	**0.0016**	18	0.6341
	PCFG$_{\text{SURP}^{1}}$ vs. PCFG$_{\text{PROB}}$	**46**	**0.0012**	**40**	**0.0016**	11	1.0000
	PCFG$_{\text{SURP}^{4/3}}$ vs. PCFG$_{\text{PROB}}$	13	0.3024	**27**	**0.0016**	2	1.0000
	PCFG$_{\text{SURP}^{2}}$ vs. PCFG$_{\text{PROB}}$	**37**	**0.0012**	**43**	**0.0016**	14	0.9856
	PCFG$_{\text{SURP}^{3/4}}$ vs. PCFG$_{\text{SURP}^{1/2}}$	10	0.3382	6	1.0000	5	1.0000
	PCFG$_{\text{SURP}^{1}}$ vs. PCFG$_{\text{SURP}^{1/2}}$	**25**	**0.0012**	**15**	**0.0130**	-2	1.0000
	PCFG$_{\text{SURP}^{4/3}}$ vs. PCFG$_{\text{SURP}^{1/2}}$	-8	0.9819	2	1.0000	-11	1.0000
	PCFG$_{\text{SURP}^{2}}$ vs. PCFG$_{\text{SURP}^{1/2}}$	**16**	**0.0465**	**17**	**0.0039**	2	1.0000
	PCFG$_{\text{SURP}^{1}}$ vs. PCFG$_{\text{SURP}^{3/4}}$	**15**	**0.0346**	9	0.3188	-7	1.0000
	PCFG$_{\text{SURP}^{4/3}}$ vs. PCFG$_{\text{SURP}^{3/4}}$	**-18**	**0.0170**	-4	1.0000	-16	0.6341
	PCFG$_{\text{SURP}^{2}}$ vs. PCFG$_{\text{SURP}^{3/4}}$	7	1.0000	12	0.1183	-3	1.0000
	PCFG$_{\text{SURP}^{4/3}}$ vs. PCFG$_{\text{SURP}^{1}}$	**-32**	**0.0012**	-13	0.0817	-9	1.0000
	PCFG$_{\text{SURP}^{2}}$ vs. PCFG$_{\text{SURP}^{1}}$	-8	0.8342	3	1.0000	4	1.0000
	PCFG$_{\text{SURP}^{2}}$ vs. PCFG$_{\text{SURP}^{4/3}}$	**24**	**0.0012**	**15**	**0.0340**	13	1.0000
	PCFG$_{\text{SURP}^{<1}}$ vs. PCFG$_{\text{SURP}^{1}}$	**-21**	**0.0022**	**-11**	**0.0371**	4	1.0000
	PCFG$_{\text{SURP}^{>1}}$ vs. PCFG$_{\text{SURP}^{1}}$	-12	0.1364	-4	1.0000	-2	1.0000
	PCFG$_{\text{SURP}^{<1}}$ vs. PCFG$_{\text{SURP}^{\geq 1}}$	**-13**	**0.0056**	-8	0.0812	4	1.0000
	PCFG$_{\text{SURP}^{>1}}$ vs. PCFG$_{\text{SURP}^{\leq 1}}$	3	1.0000	5	0.6369	-5	1.0000
GPT-2	GPT-2$_{f(\text{SURP})}$ vs. $\emptyset$	**24**	**0.0104**	**19**	**0.0147**	**55**	**0.0010**
	GPT-2$_{f(\text{SURP})}$ vs. GPT-2$_{\text{PROB}}$	**71**	**0.0011**	**48**	**0.0015**	**80**	**0.0010**
	GPT-2$_{f(\text{SURP})}$ vs. GPT-2$_{\text{SURP}^{1/2}}$	**20**	**0.0193**	2	1.0000	-2	—
	GPT-2$_{f(\text{SURP})}$ vs. GPT-2$_{\text{SURP}^{3/4}}$	-8	—	-9	—	-4	—
	GPT-2$_{f(\text{SURP})}$ vs. GPT-2$_{\text{SURP}^{1}}$	**19**	**0.0252**	8	0.6159	2	1.0000
	GPT-2$_{f(\text{SURP})}$ vs. GPT-2$_{\text{SURP}^{4/3}}$	7	1.0000	1	1.0000	0	1.0000
	GPT-2$_{f(\text{SURP})}$ vs. GPT-2$_{\text{SURP}^{2}}$	**19**	**0.0329**	6	1.0000	1	1.0000
	GPT-2$_{\text{PROB}}$ vs. $\emptyset$	-47	—	-29	—	-25	—
	GPT-2$_{\text{SURP}^{1/2}}$ vs. $\emptyset$	4	1.0000	**17**	**0.0228**	**57**	**0.0010**
	GPT-2$_{\text{SURP}^{3/4}}$ vs. $\emptyset$	**32**	**0.0011**	**28**	**0.0015**	**58**	**0.0010**
	GPT-2$_{\text{SURP}^{1}}$ vs. $\emptyset$	5	1.0000	11	0.3879	**52**	**0.0010**
	GPT-2$_{\text{SURP}^{4/3}}$ vs. $\emptyset$	17	0.1383	**18**	**0.0228**	**55**	**0.0010**
	GPT-2$_{\text{SURP}^{2}}$ vs. $\emptyset$	5	1.0000	13	0.1754	**54**	**0.0010**
	GPT-2$_{\text{SURP}^{1/2}}$ vs. GPT-2$_{\text{PROB}}$	**51**	**0.0011**	**46**	**0.0015**	**82**	**0.0010**
	GPT-2$_{\text{SURP}^{3/4}}$ vs. GPT-2$_{\text{PROB}}$	**78**	**0.0011**	**57**	**0.0015**	**84**	**0.0010**
	GPT-2$_{\text{SURP}^{1}}$ vs. GPT-2$_{\text{PROB}}$	**52**	**0.0011**	**40**	**0.0015**	**78**	**0.0010**
	GPT-2$_{\text{SURP}^{4/3}}$ vs. GPT-2$_{\text{PROB}}$	**63**	**0.0011**	**47**	**0.0015**	**80**	**0.0010**
	GPT-2$_{\text{SURP}^{2}}$ vs. GPT-2$_{\text{PROB}}$	**52**	**0.0011**	**42**	**0.0015**	**79**	**0.0010**
	GPT-2$_{\text{SURP}^{3/4}}$ vs. GPT-2$_{\text{SURP}^{1/2}}$	**28**	**0.0011**	11	0.2039	2	1.0000
	GPT-2$_{\text{SURP}^{1}}$ vs. GPT-2$_{\text{SURP}^{1/2}}$	1	1.0000	-6	1.0000	-5	1.0000
	GPT-2$_{\text{SURP}^{4/3}}$ vs. GPT-2$_{\text{SURP}^{1/2}}$	13	0.2666	1	1.0000	-2	1.0000
	GPT-2$_{\text{SURP}^{2}}$ vs. GPT-2$_{\text{SURP}^{1/2}}$	1	1.0000	-4	1.0000	-3	1.0000
	GPT-2$_{\text{SURP}^{1}}$ vs. GPT-2$_{\text{SURP}^{3/4}}$	**-27**	**0.0011**	**-17**	**0.0054**	-6	1.0000
	GPT-2$_{\text{SURP}^{4/3}}$ vs. GPT-2$_{\text{SURP}^{3/4}}$	-15	0.1108	-10	0.3117	-4	1.0000
	GPT-2$_{\text{SURP}^{2}}$ vs. GPT-2$_{\text{SURP}^{3/4}}$	**-27**	**0.0011**	**-15**	**0.0360**	-4	1.0000
	GPT-2$_{\text{SURP}^{4/3}}$ vs. GPT-2$_{\text{SURP}^{1}}$	12	0.3128	7	0.8129	2	1.0000
	GPT-2$_{\text{SURP}^{2}}$ vs. GPT-2$_{\text{SURP}^{1}}$	0	1.0000	2	1.0000	2	1.0000
	GPT-2$_{\text{SURP}^{2}}$ vs. GPT-2$_{\text{SURP}^{4/3}}$	-12	0.3256	-5	1.0000	-1	1.0000
	GPT-2$_{\text{SURP}^{<1}}$ vs. GPT-2$_{\text{SURP}^{1}}$	14	0.0659	**13**	**0.0318**	5	1.0000
	GPT-2$_{\text{SURP}^{>1}}$ vs. GPT-2$_{\text{SURP}^{1}}$	6	1.0000	7	0.6159	2	1.0000
	GPT-2$_{\text{SURP}^{<1}}$ vs. GPT-2$_{\text{SURP}^{\geq 1}}$	10	0.1079	9	0.0843	3	1.0000
	GPT-2$_{\text{SURP}^{>1}}$ vs. GPT-2$_{\text{SURP}^{\leq 1}}$	-1	1.0000	-1	1.0000	-1	1.0000

Group	Comparison	Scan Path ΔLL	Scan Path p	First Pass ΔLL	First Pass p	Go Past ΔLL	Go Past p
GPT-J	GPT-J$_{f(\text{SURP})}$ vs. $\emptyset$	7	1.0000	8	1.0000	**27**	**0.0406**
	GPT-J$_{f(\text{SURP})}$ vs. GPT-J$_{\text{PROB}}$	**49**	**0.0013**	**49**	**0.0019**	**56**	**0.0019**
	GPT-J$_{f(\text{SURP})}$ vs. GPT-J$_{\text{SURP}^{1/2}}$	-10	—	-1	—	-10	—
	GPT-J$_{f(\text{SURP})}$ vs. GPT-J$_{\text{SURP}^{3/4}}$	**25**	**0.0020**	16	0.0883	4	1.0000
	GPT-J$_{f(\text{SURP})}$ vs. GPT-J$_{\text{SURP}^{1}}$	17	0.0826	9	0.8163	-7	—
	GPT-J$_{f(\text{SURP})}$ vs. GPT-J$_{\text{SURP}^{4/3}}$	**25**	**0.0035**	14	0.1334	13	0.6045
	GPT-J$_{f(\text{SURP})}$ vs. GPT-J$_{\text{SURP}^{2}}$	**26**	**0.0020**	9	0.8611	13	0.6917
	GPT-J$_{\text{PROB}}$ vs. $\emptyset$	-42	—	-41	—	-30	—
	GPT-J$_{\text{SURP}^{1/2}}$ vs. $\emptyset$	17	0.0594	9	0.8611	**37**	**0.0019**
	GPT-J$_{\text{SURP}^{3/4}}$ vs. $\emptyset$	-18	—	-8	—	23	0.0952
	GPT-J$_{\text{SURP}^{1}}$ vs. $\emptyset$	-10	—	-1	—	**34**	**0.0065**
	GPT-J$_{\text{SURP}^{4/3}}$ vs. $\emptyset$	-18	—	-6	—	14	0.6917
	GPT-J$_{\text{SURP}^{2}}$ vs. $\emptyset$	-19	—	-1	—	14	0.6895
	GPT-J$_{\text{SURP}^{1/2}}$ vs. GPT-J$_{\text{PROB}}$	**60**	**0.0013**	**50**	**0.0019**	**67**	**0.0019**
	GPT-J$_{\text{SURP}^{3/4}}$ vs. GPT-J$_{\text{PROB}}$	**24**	**0.0028**	**34**	**0.0019**	**53**	**0.0019**
	GPT-J$_{\text{SURP}^{1}}$ vs. GPT-J$_{\text{PROB}}$	**33**	**0.0013**	**40**	**0.0019**	**63**	**0.0019**
	GPT-J$_{\text{SURP}^{4/3}}$ vs. GPT-J$_{\text{PROB}}$	**24**	**0.0049**	**35**	**0.0019**	**43**	**0.0019**
	GPT-J$_{\text{SURP}^{2}}$ vs. GPT-J$_{\text{PROB}}$	**23**	**0.0099**	**40**	**0.0019**	**44**	**0.0019**
	GPT-J$_{\text{SURP}^{3/4}}$ vs. GPT-J$_{\text{SURP}^{1/2}}$	**-35**	**0.0013**	**-16**	**0.0308**	-14	0.4258
	GPT-J$_{\text{SURP}^{1}}$ vs. GPT-J$_{\text{SURP}^{1/2}}$	**-27**	**0.0013**	-10	0.5054	-3	1.0000
	GPT-J$_{\text{SURP}^{4/3}}$ vs. GPT-J$_{\text{SURP}^{1/2}}$	**-35**	**0.0013**	-15	0.0808	**-23**	**0.0227**
	GPT-J$_{\text{SURP}^{2}}$ vs. GPT-J$_{\text{SURP}^{1/2}}$	**-37**	**0.0013**	-10	0.7012	**-23**	**0.0355**
	GPT-J$_{\text{SURP}^{1}}$ vs. GPT-J$_{\text{SURP}^{3/4}}$	9	0.7638	6	1.0000	11	0.8035
	GPT-J$_{\text{SURP}^{4/3}}$ vs. GPT-J$_{\text{SURP}^{3/4}}$	0	1.0000	2	1.0000	-9	1.0000
	GPT-J$_{\text{SURP}^{2}}$ vs. GPT-J$_{\text{SURP}^{3/4}}$	-1	1.0000	7	1.0000	-9	1.0000
	GPT-J$_{\text{SURP}^{4/3}}$ vs. GPT-J$_{\text{SURP}^{1}}$	-8	0.7459	-5	1.0000	**-20**	**0.0355**
	GPT-J$_{\text{SURP}^{2}}$ vs. GPT-J$_{\text{SURP}^{1}}$	-10	0.6044	0	1.0000	-20	0.0596
	GPT-J$_{\text{SURP}^{2}}$ vs. GPT-J$_{\text{SURP}^{4/3}}$	-2	1.0000	5	1.0000	1	1.0000
	GPT-J$_{\text{SURP}^{<1}}$ vs. GPT-J$_{\text{SURP}^{1}}$	**21**	**0.0020**	0	1.0000	-1	1.0000
	GPT-J$_{\text{SURP}^{>1}}$ vs. GPT-J$_{\text{SURP}^{1}}$	-8	0.6044	-2	1.0000	**-20**	**0.0177**
	GPT-J$_{\text{SURP}^{<1}}$ vs. GPT-J$_{\text{SURP}^{\geq 1}}$	**26**	**0.0013**	1	1.0000	**16**	**0.0250**
	GPT-J$_{\text{SURP}^{>1}}$ vs. GPT-J$_{\text{SURP}^{\leq 1}}$	**-23**	**0.0013**	-1	1.0000	**-20**	**0.0029**
GPT-3	GPT-3$_{f(\text{SURP})}$ vs. $\emptyset$	-21	—	-17	—	5	1.0000
	GPT-3$_{f(\text{SURP})}$ vs. GPT-3$_{\text{PROB}}$	**23**	**0.0199**	**22**	**0.0152**	**51**	**0.0019**
	GPT-3$_{f(\text{SURP})}$ vs. GPT-3$_{\text{SURP}^{1/2}}$	-13	—	-4	—	-3	—
	GPT-3$_{f(\text{SURP})}$ vs. GPT-3$_{\text{SURP}^{3/4}}$	-29	—	-11	—	-21	—
	GPT-3$_{f(\text{SURP})}$ vs. GPT-3$_{\text{SURP}^{1}}$	-5	—	0	1.0000	-12	—
	GPT-3$_{f(\text{SURP})}$ vs. GPT-3$_{\text{SURP}^{4/3}}$	16	0.0903	4	1.0000	3	1.0000
	GPT-3$_{f(\text{SURP})}$ vs. GPT-3$_{\text{SURP}^{2}}$	1	1.0000	-2	—	5	1.0000
	GPT-3$_{\text{PROB}}$ vs. $\emptyset$	-44	—	-39	—	-46	—
	GPT-3$_{\text{SURP}^{1/2}}$ vs. $\emptyset$	-8	—	-13	—	8	1.0000
	GPT-3$_{\text{SURP}^{3/4}}$ vs. $\emptyset$	8	1.0000	-6	—	**26**	**0.0245**
	GPT-3$_{\text{SURP}^{1}}$ vs. $\emptyset$	-16	—	-17	—	17	0.3944
	GPT-3$_{\text{SURP}^{4/3}}$ vs. $\emptyset$	-37	—	-21	—	2	1.0000
	GPT-3$_{\text{SURP}^{2}}$ vs. $\emptyset$	-22	—	-15	—	0	1.0000
	GPT-3$_{\text{SURP}^{1/2}}$ vs. GPT-3$_{\text{PROB}}$	**36**	**0.0014**	**26**	**0.0043**	**54**	**0.0019**
	GPT-3$_{\text{SURP}^{3/4}}$ vs. GPT-3$_{\text{PROB}}$	**52**	**0.0014**	**33**	**0.0043**	**72**	**0.0019**
	GPT-3$_{\text{SURP}^{1}}$ vs. GPT-3$_{\text{PROB}}$	**28**	**0.0022**	**22**	**0.0167**	**63**	**0.0019**
	GPT-3$_{\text{SURP}^{4/3}}$ vs. GPT-3$_{\text{PROB}}$	7	1.0000	**18**	**0.0438**	**48**	**0.0019**
	GPT-3$_{\text{SURP}^{2}}$ vs. GPT-3$_{\text{PROB}}$	**22**	**0.0253**	**24**	**0.0065**	**46**	**0.0019**
	GPT-3$_{\text{SURP}^{3/4}}$ vs. GPT-3$_{\text{SURP}^{1/2}}$	15	0.0834	8	0.8585	18	0.1234
	GPT-3$_{\text{SURP}^{1}}$ vs. GPT-3$_{\text{SURP}^{1/2}}$	-8	1.0000	-4	1.0000	9	1.0000
	GPT-3$_{\text{SURP}^{4/3}}$ vs. GPT-3$_{\text{SURP}^{1/2}}$	**-29**	**0.0014**	-7	1.0000	-6	1.0000
	GPT-3$_{\text{SURP}^{2}}$ vs. GPT-3$_{\text{SURP}^{1/2}}$	-14	0.1970	-1	1.0000	-8	1.0000
	GPT-3$_{\text{SURP}^{1}}$ vs. GPT-3$_{\text{SURP}^{3/4}}$	**-24**	**0.0022**	-11	0.2968	-9	1.0000
	GPT-3$_{\text{SURP}^{4/3}}$ vs. GPT-3$_{\text{SURP}^{3/4}}$	**-45**	**0.0014**	-15	0.0708	**-24**	**0.0247**
	GPT-3$_{\text{SURP}^{2}}$ vs. GPT-3$_{\text{SURP}^{3/4}}$	**-29**	**0.0014**	-9	0.8585	**-26**	**0.0065**
	GPT-3$_{\text{SURP}^{4/3}}$ vs. GPT-3$_{\text{SURP}^{1}}$	**-21**	**0.0130**	-4	1.0000	-15	0.4033
	GPT-3$_{\text{SURP}^{2}}$ vs. GPT-3$_{\text{SURP}^{1}}$	-6	1.0000	2	1.0000	-17	0.2820
	GPT-3$_{\text{SURP}^{2}}$ vs. GPT-3$_{\text{SURP}^{4/3}}$	15	0.1092	6	1.0000	-2	1.0000
	GPT-3$_{\text{SURP}^{<1}}$ vs. GPT-3$_{\text{SURP}^{1}}$	**17**	**0.0227**	6	1.0000	-6	1.0000
	GPT-3$_{\text{SURP}^{>1}}$ vs. GPT-3$_{\text{SURP}^{1}}$	-13	0.1092	-1	1.0000	-15	0.2787
	GPT-3$_{\text{SURP}^{<1}}$ vs. GPT-3$_{\text{SURP}^{\geq 1}}$	**23**	**0.0014**	6	0.6745	4	1.0000
	GPT-3$_{\text{SURP}^{>1}}$ vs. GPT-3$_{\text{SURP}^{\leq 1}}$	**-26**	**0.0014**	-6	0.8585	-11	0.3714
Cloze	Cloze$_{f(\text{SURP})}$ vs. $\emptyset$	14	0.3005	4	1.0000	-19	—
	Cloze$_{f(\text{SURP})}$ vs. Cloze$_{\text{PROB}}$	**51**	**0.0016**	**30**	**0.0019**	16	0.6025
	Cloze$_{f(\text{SURP})}$ vs. Cloze$_{\text{SURP}^{1/2}}$	14	0.2314	3	1.0000	-12	—
	Cloze$_{f(\text{SURP})}$ vs. Cloze$_{\text{SURP}^{3/4}}$	**22**	**0.0043**	10	0.2964	-17	—
	Cloze$_{f(\text{SURP})}$ vs. Cloze$_{\text{SURP}^{1}}$	9	0.9129	1	1.0000	-20	—
	Cloze$_{f(\text{SURP})}$ vs. Cloze$_{\text{SURP}^{4/3}}$	13	0.2787	14	0.0552	-13	—
	Cloze$_{f(\text{SURP})}$ vs. Cloze$_{\text{SURP}^{2}}$	**24**	**0.0016**	**20**	**0.0019**	-12	—
	Cloze$_{\text{PROB}}$ vs. $\emptyset$	-37	—	-26	—	-35	—
	Cloze$_{\text{SURP}^{1/2}}$ vs. $\emptyset$	0	1.0000	1	1.0000	-7	—
	Cloze$_{\text{SURP}^{3/4}}$ vs. $\emptyset$	-8	—	-6	—	-1	—
	Cloze$_{\text{SURP}^{1}}$ vs. $\emptyset$	5	1.0000	2	1.0000	2	1.0000
	Cloze$_{\text{SURP}^{4/3}}$ vs. $\emptyset$	1	1.0000	-11	—	-6	—
	Cloze$_{\text{SURP}^{2}}$ vs. $\emptyset$	-10	—	-16	—	-6	—
	Cloze$_{\text{SURP}^{1/2}}$ vs. Cloze$_{\text{PROB}}$	**36**	**0.0016**	**27**	**0.0019**	**28**	**0.0052**
	Cloze$_{\text{SURP}^{3/4}}$ vs. Cloze$_{\text{PROB}}$	**29**	**0.0016**	**20**	**0.0019**	**34**	**0.0043**
	Cloze$_{\text{SURP}^{1}}$ vs. Cloze$_{\text{PROB}}$	**42**	**0.0016**	**29**	**0.0019**	**37**	**0.0043**
	Cloze$_{\text{SURP}^{4/3}}$ vs. Cloze$_{\text{PROB}}$	**37**	**0.0016**	**16**	**0.0496**	**29**	**0.0052**
	Cloze$_{\text{SURP}^{2}}$ vs. Cloze$_{\text{PROB}}$	**27**	**0.0016**	10	0.4316	**29**	**0.0065**
	Cloze$_{\text{SURP}^{3/4}}$ vs. Cloze$_{\text{SURP}^{1/2}}$	-7	1.0000	-7	0.7166	6	1.0000
	Cloze$_{\text{SURP}^{1}}$ vs. Cloze$_{\text{SURP}^{1/2}}$	5	1.0000	2	1.0000	9	1.0000
	Cloze$_{\text{SURP}^{4/3}}$ vs. Cloze$_{\text{SURP}^{1/2}}$	1	1.0000	-11	0.1713	1	1.0000
	Cloze$_{\text{SURP}^{2}}$ vs. Cloze$_{\text{SURP}^{1/2}}$	-9	0.8701	**-17**	**0.0101**	1	1.0000
	Cloze$_{\text{SURP}^{1}}$ vs. Cloze$_{\text{SURP}^{3/4}}$	13	0.2787	9	0.4259	3	1.0000
	Cloze$_{\text{SURP}^{4/3}}$ vs. Cloze$_{\text{SURP}^{3/4}}$	8	0.9178	-4	1.0000	-5	1.0000
	Cloze$_{\text{SURP}^{2}}$ vs. Cloze$_{\text{SURP}^{3/4}}$	-2	1.0000	-10	0.3593	-5	1.0000
	Cloze$_{\text{SURP}^{4/3}}$ vs. Cloze$_{\text{SURP}^{1}}$	-4	1.0000	-13	0.0909	-8	1.0000
	Cloze$_{\text{SURP}^{2}}$ vs. Cloze$_{\text{SURP}^{1}}$	-15	0.2000	**-19**	**0.0081**	-8	1.0000
	Cloze$_{\text{SURP}^{2}}$ vs. Cloze$_{\text{SURP}^{4/3}}$	-10	0.6692	-5	1.0000	0	1.0000
	Cloze$_{\text{SURP}^{<1}}$ vs. Cloze$_{\text{SURP}^{1}}$	-10	0.4407	-5	1.0000	-8	1.0000
	Cloze$_{\text{SURP}^{>1}}$ vs. Cloze$_{\text{SURP}^{1}}$	-11	0.4164	**-15**	**0.0117**	-8	1.0000
	Cloze$_{\text{SURP}^{<1}}$ vs. Cloze$_{\text{SURP}^{\geq 1}}$	-5	1.0000	8	0.0978	-1	1.0000
	Cloze$_{\text{SURP}^{>1}}$ vs. Cloze$_{\text{SURP}^{\leq 1}}$	-6	0.9129	**-12**	**0.0019**	-2	1.0000

Group	Comparison	Scan Path ΔLL	Scan Path p	First Pass ΔLL	First Pass p	Go Past ΔLL	Go Past p
All LMs	All-LMs$_{f(\text{SURP})}$ vs. $\emptyset$	-5	—	0	1.0000	**32**	**0.0013**
	All-LMs$_{f(\text{SURP})}$ vs. All-LMs$_{\text{PROB}}$	**36**	**0.0013**	**34**	**0.0019**	**56**	**0.0013**
	All-LMs$_{f(\text{SURP})}$ vs. All-LMs$_{\text{SURP}^{1/2}}$	3	1.0000	5	1.0000	3	1.0000
	All-LMs$_{f(\text{SURP})}$ vs. All-LMs$_{\text{SURP}^{3/4}}$	-8	—	2	1.0000	-2	—
	All-LMs$_{f(\text{SURP})}$ vs. All-LMs$_{\text{SURP}^{1}}$	1	1.0000	-1	—	0	1.0000
	All-LMs$_{f(\text{SURP})}$ vs. All-LMs$_{\text{SURP}^{4/3}}$	**13**	**0.0080**	4	1.0000	9	0.3162
	All-LMs$_{f(\text{SURP})}$ vs. All-LMs$_{\text{SURP}^{2}}$	7	0.3117	3	1.0000	**15**	**0.0137**
	All-LMs$_{\text{PROB}}$ vs. $\emptyset$	-41	—	-34	—	-24	—
	All-LMs$_{\text{SURP}^{1/2}}$ vs. $\emptyset$	-8	—	-5	—	**28**	**0.0013**
	All-LMs$_{\text{SURP}^{3/4}}$ vs. $\emptyset$	3	1.0000	-2	—	**33**	**0.0013**
	All-LMs$_{\text{SURP}^{1}}$ vs. $\emptyset$	-6	—	1	1.0000	**31**	**0.0022**
	All-LMs$_{\text{SURP}^{4/3}}$ vs. $\emptyset$	-18	—	-4	—	**22**	**0.0317**
	All-LMs$_{\text{SURP}^{2}}$ vs. $\emptyset$	-12	—	-4	—	16	0.2189
	All-LMs$_{\text{SURP}^{1/2}}$ vs. All-LMs$_{\text{PROB}}$	**33**	**0.0013**	**29**	**0.0019**	**52**	**0.0013**
	All-LMs$_{\text{SURP}^{3/4}}$ vs. All-LMs$_{\text{PROB}}$	**44**	**0.0013**	**33**	**0.0019**	**58**	**0.0013**
	All-LMs$_{\text{SURP}^{1}}$ vs. All-LMs$_{\text{PROB}}$	**35**	**0.0013**	**35**	**0.0019**	**55**	**0.0013**
	All-LMs$_{\text{SURP}^{4/3}}$ vs. All-LMs$_{\text{PROB}}$	**23**	**0.0013**	**30**	**0.0019**	**46**	**0.0013**
	All-LMs$_{\text{SURP}^{2}}$ vs. All-LMs$_{\text{PROB}}$	**28**	**0.0013**	**31**	**0.0019**	**40**	**0.0013**
	All-LMs$_{\text{SURP}^{3/4}}$ vs. All-LMs$_{\text{SURP}^{1/2}}$	**11**	**0.0043**	4	1.0000	5	1.0000
	All-LMs$_{\text{SURP}^{1}}$ vs. All-LMs$_{\text{SURP}^{1/2}}$	2	1.0000	6	0.6331	3	1.0000
	All-LMs$_{\text{SURP}^{4/3}}$ vs. All-LMs$_{\text{SURP}^{1/2}}$	**-10**	**0.0211**	1	1.0000	-6	1.0000
	All-LMs$_{\text{SURP}^{2}}$ vs. All-LMs$_{\text{SURP}^{1/2}}$	-5	0.9474	2	1.0000	-12	0.0642
	All-LMs$_{\text{SURP}^{1}}$ vs. All-LMs$_{\text{SURP}^{3/4}}$	-9	0.0504	2	1.0000	-2	1.0000
	All-LMs$_{\text{SURP}^{4/3}}$ vs. All-LMs$_{\text{SURP}^{3/4}}$	**-21**	**0.0013**	-3	1.0000	-11	0.1013
	All-LMs$_{\text{SURP}^{2}}$ vs. All-LMs$_{\text{SURP}^{3/4}}$	**-16**	**0.0013**	-2	1.0000	**-17**	**0.0022**
	All-LMs$_{\text{SURP}^{4/3}}$ vs. All-LMs$_{\text{SURP}^{1}}$	**-12**	**0.0093**	-5	1.0000	-9	0.2928
	All-LMs$_{\text{SURP}^{2}}$ vs. All-LMs$_{\text{SURP}^{1}}$	-6	0.4486	-4	1.0000	**-15**	**0.0089**
	All-LMs$_{\text{SURP}^{2}}$ vs. All-LMs$_{\text{SURP}^{4/3}}$	6	0.5189	1	1.0000	-6	1.0000
	All-LMs$_{\text{SURP}^{<1}}$ vs. All-LMs$_{\text{SURP}^{1}}$	4	1.0000	-3	1.0000	1	1.0000
	All-LMs$_{\text{SURP}^{>1}}$ vs. All-LMs$_{\text{SURP}^{1}}$	-8	0.0931	-4	1.0000	**-14**	**0.0060**
	All-LMs$_{\text{SURP}^{<1}}$ vs. All-LMs$_{\text{SURP}^{\geq 1}}$	**10**	**0.0013**	0	1.0000	**10**	**0.0078**
	All-LMs$_{\text{SURP}^{>1}}$ vs. All-LMs$_{\text{SURP}^{\leq 1}}$	**-8**	**0.0043**	-1	1.0000	**-15**	**0.0013**
Between LMs	n-gram$_{f(\text{SURP})}$ vs. Cloze$_{f(\text{SURP})}$	**-35**	**0.0014**	-11	0.3434	**61**	**0.0011**
	PCFG$_{f(\text{SURP})}$ vs. Cloze$_{f(\text{SURP})}$	**-19**	**0.0439**	5	1.0000	**29**	**0.0095**
	GPT-2$_{f(\text{SURP})}$ vs. Cloze$_{f(\text{SURP})}$	10	0.6695	16	0.0680	**73**	**0.0011**
	GPT-J$_{f(\text{SURP})}$ vs. Cloze$_{f(\text{SURP})}$	-7	1.0000	4	1.0000	**45**	**0.0011**
	GPT-3$_{f(\text{SURP})}$ vs. Cloze$_{f(\text{SURP})}$	**-35**	**0.0014**	**-21**	**0.0097**	24	0.0561
	PCFG$_{f(\text{SURP})}$ vs. n-gram$_{f(\text{SURP})}$	16	0.0787	**16**	**0.0406**	**-32**	**0.0027**
	GPT-2$_{f(\text{SURP})}$ vs. n-gram$_{f(\text{SURP})}$	**45**	**0.0014**	**27**	**0.0014**	13	0.6638
	GPT-J$_{f(\text{SURP})}$ vs. n-gram$_{f(\text{SURP})}$	**28**	**0.0018**	15	0.1116	-15	0.5080
	GPT-3$_{f(\text{SURP})}$ vs. n-gram$_{f(\text{SURP})}$	0	1.0000	-10	0.5652	**-37**	**0.0039**
	GPT-2$_{f(\text{SURP})}$ vs. PCFG$_{f(\text{SURP})}$	**29**	**0.0018**	11	0.4686	**44**	**0.0011**
	GPT-J$_{f(\text{SURP})}$ vs. PCFG$_{f(\text{SURP})}$	12	0.5001	-1	1.0000	16	0.4152
	GPT-3$_{f(\text{SURP})}$ vs. PCFG$_{f(\text{SURP})}$	-16	0.1293	**-26**	**0.0014**	-5	1.0000
	GPT-J$_{f(\text{SURP})}$ vs. GPT-2$_{f(\text{SURP})}$	-17	0.1125	-11	0.3347	**-28**	**0.0156**
	GPT-3$_{f(\text{SURP})}$ vs. GPT-2$_{f(\text{SURP})}$	**-45**	**0.0014**	**-37**	**0.0014**	**-49**	**0.0011**
	GPT-3$_{f(\text{SURP})}$ vs. GPT-J$_{f(\text{SURP})}$	**-28**	**0.0031**	**-25**	**0.0014**	-21	0.0865
	GPT-2PCFG$_{f(\text{SURP})}$ vs. GPT-2$_{f(\text{SURP})}$	-13	—	-2	—	**22**	**0.0206**
GPT-2 (Normal Error)	GPT-2$_{f(\text{SURP})}$ vs. $\emptyset$	**200**	**0.0442**	**127**	**0.0035**	76	1.0000
	GPT-2$_{f(\text{SURP})}$ vs. GPT-2$_{\text{PROB}}$	**217**	**0.0087**	**115**	**0.0087**	**269**	**0.0022**
	GPT-2$_{f(\text{SURP})}$ vs. GPT-2$_{\text{SURP}^{1/2}}$	16	1.0000	-29	—	-70	—
	GPT-2$_{f(\text{SURP})}$ vs. GPT-2$_{\text{SURP}^{3/4}}$	38	1.0000	-1	—	-100	—
	GPT-2$_{f(\text{SURP})}$ vs. GPT-2$_{\text{SURP}^{1}}$	-16	—	-17	—	-38	—
	GPT-2$_{f(\text{SURP})}$ vs. GPT-2$_{\text{SURP}^{4/3}}$	-34	—	-29	—	-15	—
	GPT-2$_{f(\text{SURP})}$ vs. GPT-2$_{\text{SURP}^{2}}$	2	1.0000	5	1.0000	-36	—
	GPT-2$_{\text{PROB}}$ vs. $\emptyset$	-17	—	12	1.0000	-193	—
	GPT-2$_{\text{SURP}^{1/2}}$ vs. $\emptyset$	**184**	**0.0496**	**156**	**0.0016**	146	0.2035
	GPT-2$_{\text{SURP}^{3/4}}$ vs. $\emptyset$	163	0.1277	**128**	**0.0016**	176	0.0941
	GPT-2$_{\text{SURP}^{1}}$ vs. $\emptyset$	**216**	**0.0032**	**144**	**0.0016**	113	0.8098
	GPT-2$_{\text{SURP}^{4/3}}$ vs. $\emptyset$	**234**	**0.0032**	**156**	**0.0016**	90	1.0000
	GPT-2$_{\text{SURP}^{2}}$ vs. $\emptyset$	**198**	**0.0186**	**122**	**0.0016**	111	0.8561
	GPT-2$_{\text{SURP}^{1/2}}$ vs. GPT-2$_{\text{PROB}}$	**200**	**0.0195**	**144**	**0.0016**	**339**	**0.0022**
	GPT-2$_{\text{SURP}^{3/4}}$ vs. GPT-2$_{\text{PROB}}$	**179**	**0.0361**	**116**	**0.0026**	**369**	**0.0022**
	GPT-2$_{\text{SURP}^{1}}$ vs. GPT-2$_{\text{PROB}}$	**233**	**0.0032**	**132**	**0.0016**	**307**	**0.0022**
	GPT-2$_{\text{SURP}^{4/3}}$ vs. GPT-2$_{\text{PROB}}$	**251**	**0.0032**	**144**	**0.0016**	**284**	**0.0022**
	GPT-2$_{\text{SURP}^{2}}$ vs. GPT-2$_{\text{PROB}}$	**215**	**0.0078**	**110**	**0.0026**	**305**	**0.0022**
	GPT-2$_{\text{SURP}^{3/4}}$ vs. GPT-2$_{\text{SURP}^{1/2}}$	-21	1.0000	-28	1.0000	30	1.0000
	GPT-2$_{\text{SURP}^{1}}$ vs. GPT-2$_{\text{SURP}^{1/2}}$	32	1.0000	-12	1.0000	-33	1.0000
	GPT-2$_{\text{SURP}^{4/3}}$ vs. GPT-2$_{\text{SURP}^{1/2}}$	51	1.0000	0	1.0000	-56	1.0000
	GPT-2$_{\text{SURP}^{2}}$ vs. GPT-2$_{\text{SURP}^{1/2}}$	15	1.0000	-34	1.0000	-35	1.0000
	GPT-2$_{\text{SURP}^{1}}$ vs. GPT-2$_{\text{SURP}^{3/4}}$	53	1.0000	15	1.0000	-62	1.0000
	GPT-2$_{\text{SURP}^{4/3}}$ vs. GPT-2$_{\text{SURP}^{3/4}}$	72	1.0000	28	1.0000	-85	0.8561
	GPT-2$_{\text{SURP}^{2}}$ vs. GPT-2$_{\text{SURP}^{3/4}}$	36	1.0000	-6	1.0000	-64	1.0000
	GPT-2$_{\text{SURP}^{4/3}}$ vs. GPT-2$_{\text{SURP}^{1}}$	18	1.0000	12	1.0000	-23	1.0000
	GPT-2$_{\text{SURP}^{2}}$ vs. GPT-2$_{\text{SURP}^{1}}$	-18	1.0000	-22	1.0000	-2	1.0000
	GPT-2$_{\text{SURP}^{2}}$ vs. GPT-2$_{\text{SURP}^{4/3}}$	-36	1.0000	-34	1.0000	21	1.0000
	GPT-2$_{\text{SURP}^{<1}}$ vs. GPT-2$_{\text{SURP}^{1}}$	-46	1.0000	-15	1.0000	48	1.0000
	GPT-2$_{\text{SURP}^{>1}}$ vs. GPT-2$_{\text{SURP}^{1}}$	0	1.0000	-2	1.0000	-2	1.0000
	GPT-2$_{\text{SURP}^{<1}}$ vs. GPT-2$_{\text{SURP}^{\geq 1}}$	-46	1.0000	-14	1.0000	50	1.0000
	GPT-2$_{\text{SURP}^{>1}}$ vs. GPT-2$_{\text{SURP}^{\leq 1}}$	36	1.0000	14	1.0000	-38	1.0000
	GPT-2$^{(\text{EXGAUSS})}_{f(\text{SURP})}$ vs. GPT-2$^{(\text{NORMAL})}_{f(\text{SURP})}$	**14699**	**0.0011**	**9470**	**0.0015**	**17950**	**0.0010**
Prob vs. Surp	GPT-2$_{\text{PROB}+\text{SURP}^{1}}$ vs. GPT-2$_{\text{PROB}}$	**33**	**0.0006**	**30**	**0.0003**	**49**	**0.0003**
	GPT-2$_{\text{PROB}+\text{SURP}^{1}}$ vs. GPT-2$_{\text{SURP}^{1}}$	-19	—	-10	—	-28	—

Table S8. Testing Results on Provo (Eye-Tracking). Results of key statistical comparisons based on permutation tests of the difference in average test set likelihood between the alternative and null model in each comparison (ΔLL). Subscripts indicate the assumed functional form of predictability effects: $f(\text{SURP})$ (nonlinear in surprisal), PROB (linear in probability), and SURP^a (linear in surprisal raised to exponent a, such that e.g., SURP^1 is linear in surprisal). Boldface indicates statistical significance. In directional tests where one hypothesis subsumes the other (e.g., $f(\text{SURP})$ vs. SURP^1, since a nonlinear surprisal effect subsumes a linear one), dashes (—) indicate failure of the alternative hypothesis (left) to improve over the null hypothesis (right). Otherwise, cells are color coded using cyan to indicate that the hypothesis on the left outperforms the one on the right, and using magenta to indicate that the hypothesis on the right outperforms the one on the left. All p-values are corrected for false discovery rate (15) within each family of tests (delimited by single horizontal lines). Key comparisons are highlighted.

	Comparison	Combined Datasets ΔLL	p
n-gram	n-gram$_{f(\text{SURP})}$ vs. $\emptyset$	**941**	**0.0005**
	n-gram$_{f(\text{SURP})}$ vs. n-gram$_{\text{PROB}}$	**405**	**0.0005**
	n-gram$_{f(\text{SURP})}$ vs. n-gram$_{\text{SURP}^{1/2}}$	-203	—
	n-gram$_{f(\text{SURP})}$ vs. n-gram$_{\text{SURP}^{3/4}}$	-67	—
	n-gram$_{f(\text{SURP})}$ vs. n-gram$_{\text{SURP}^{1}}$	-788	—
	n-gram$_{f(\text{SURP})}$ vs. n-gram$_{\text{SURP}^{4/3}}$	-662	—
	n-gram$_{f(\text{SURP})}$ vs. n-gram$_{\text{SURP}^{2}}$	-536	—
	n-gram$_{\text{PROB}}$ vs. $\emptyset$	**535**	**0.0005**
	n-gram$_{\text{SURP}^{1/2}}$ vs. $\emptyset$	**1144**	**0.0005**
	n-gram$_{\text{SURP}^{3/4}}$ vs. $\emptyset$	**1008**	**0.0005**
	n-gram$_{\text{SURP}^{1}}$ vs. $\emptyset$	**1729**	**0.0005**
	n-gram$_{\text{SURP}^{4/3}}$ vs. $\emptyset$	**1603**	**0.0005**
	n-gram$_{\text{SURP}^{2}}$ vs. $\emptyset$	**1477**	**0.0005**
	n-gram$_{\text{SURP}^{1/2}}$ vs. n-gram$_{\text{PROB}}$	**608**	**0.0005**
	n-gram$_{\text{SURP}^{3/4}}$ vs. n-gram$_{\text{PROB}}$	**472**	**0.0005**
	n-gram$_{\text{SURP}^{1}}$ vs. n-gram$_{\text{PROB}}$	**1194**	**0.0005**
	n-gram$_{\text{SURP}^{4/3}}$ vs. n-gram$_{\text{PROB}}$	**1067**	**0.0005**
	n-gram$_{\text{SURP}^{2}}$ vs. n-gram$_{\text{PROB}}$	**941**	**0.0005**
	n-gram$_{\text{SURP}^{3/4}}$ vs. n-gram$_{\text{SURP}^{1/2}}$	-136	0.1910
	n-gram$_{\text{SURP}^{1}}$ vs. n-gram$_{\text{SURP}^{1/2}}$	**586**	**0.0005**
	n-gram$_{\text{SURP}^{4/3}}$ vs. n-gram$_{\text{SURP}^{1/2}}$	**459**	**0.0005**
	n-gram$_{\text{SURP}^{2}}$ vs. n-gram$_{\text{SURP}^{1/2}}$	**333**	**0.0005**
	n-gram$_{\text{SURP}^{1}}$ vs. n-gram$_{\text{SURP}^{3/4}}$	**721**	**0.0005**
	n-gram$_{\text{SURP}^{4/3}}$ vs. n-gram$_{\text{SURP}^{3/4}}$	**595**	**0.0005**
	n-gram$_{\text{SURP}^{2}}$ vs. n-gram$_{\text{SURP}^{3/4}}$	**469**	**0.0005**
	n-gram$_{\text{SURP}^{4/3}}$ vs. n-gram$_{\text{SURP}^{1}}$	-126	0.1910
	n-gram$_{\text{SURP}^{2}}$ vs. n-gram$_{\text{SURP}^{1}}$	**-252**	**0.0005**
	n-gram$_{\text{SURP}^{2}}$ vs. n-gram$_{\text{SURP}^{4/3}}$	-126	0.1910
	n-gram$_{\text{SURP}^{<1}}$ vs. n-gram$_{\text{SURP}^{1}}$	**-652**	**0.0005**
	n-gram$_{\text{SURP}^{>1}}$ vs. n-gram$_{\text{SURP}^{1}}$	-126	0.0881
	n-gram$_{\text{SURP}^{<1}}$ vs. n-gram$_{\text{SURP}^{\geq 1}}$	**-571**	**0.0005**
	n-gram$_{\text{SURP}^{>1}}$ vs. n-gram$_{\text{SURP}^{\leq 1}}$	**251**	**0.0005**
PCFG	PCFG$_{f(\text{SURP})}$ vs. $\emptyset$	**774**	**0.0009**
	PCFG$_{f(\text{SURP})}$ vs. PCFG$_{\text{PROB}}$	**712**	**0.0009**
	PCFG$_{f(\text{SURP})}$ vs. PCFG$_{\text{SURP}^{1/2}}$	**232**	**0.0144**
	PCFG$_{f(\text{SURP})}$ vs. PCFG$_{\text{SURP}^{3/4}}$	106	0.4935
	PCFG$_{f(\text{SURP})}$ vs. PCFG$_{\text{SURP}^{1}}$	**198**	**0.0279**
	PCFG$_{f(\text{SURP})}$ vs. PCFG$_{\text{SURP}^{4/3}}$	**241**	**0.0017**
	PCFG$_{f(\text{SURP})}$ vs. PCFG$_{\text{SURP}^{2}}$	**402**	**0.0009**
	PCFG$_{\text{PROB}}$ vs. $\emptyset$	62	1.0000
	PCFG$_{\text{SURP}^{1/2}}$ vs. $\emptyset$	**542**	**0.0009**
	PCFG$_{\text{SURP}^{3/4}}$ vs. $\emptyset$	**668**	**0.0009**
	PCFG$_{\text{SURP}^{1}}$ vs. $\emptyset$	**576**	**0.0009**
	PCFG$_{\text{SURP}^{4/3}}$ vs. $\emptyset$	**533**	**0.0009**
	PCFG$_{\text{SURP}^{2}}$ vs. $\emptyset$	**372**	**0.0009**
	PCFG$_{\text{SURP}^{1/2}}$ vs. PCFG$_{\text{PROB}}$	**480**	**0.0009**
	PCFG$_{\text{SURP}^{3/4}}$ vs. PCFG$_{\text{PROB}}$	**606**	**0.0009**
	PCFG$_{\text{SURP}^{1}}$ vs. PCFG$_{\text{PROB}}$	**514**	**0.0009**
	PCFG$_{\text{SURP}^{4/3}}$ vs. PCFG$_{\text{PROB}}$	**471**	**0.0009**
	PCFG$_{\text{SURP}^{2}}$ vs. PCFG$_{\text{PROB}}$	**310**	**0.0009**
	PCFG$_{\text{SURP}^{3/4}}$ vs. PCFG$_{\text{SURP}^{1/2}}$	126	0.2603
	PCFG$_{\text{SURP}^{1}}$ vs. PCFG$_{\text{SURP}^{1/2}}$	34	1.0000
	PCFG$_{\text{SURP}^{4/3}}$ vs. PCFG$_{\text{SURP}^{1/2}}$	-9	1.0000
	PCFG$_{\text{SURP}^{2}}$ vs. PCFG$_{\text{SURP}^{1/2}}$	-170	0.1405
	PCFG$_{\text{SURP}^{1}}$ vs. PCFG$_{\text{SURP}^{3/4}}$	-92	0.6532
	PCFG$_{\text{SURP}^{4/3}}$ vs. PCFG$_{\text{SURP}^{3/4}}$	-135	0.1570
	PCFG$_{\text{SURP}^{2}}$ vs. PCFG$_{\text{SURP}^{3/4}}$	**-296**	**0.0009**
	PCFG$_{\text{SURP}^{4/3}}$ vs. PCFG$_{\text{SURP}^{1}}$	-43	1.0000
	PCFG$_{\text{SURP}^{2}}$ vs. PCFG$_{\text{SURP}^{1}}$	**-204**	**0.0106**
	PCFG$_{\text{SURP}^{2}}$ vs. PCFG$_{\text{SURP}^{4/3}}$	-161	0.1255
	PCFG$_{\text{SURP}^{<1}}$ vs. PCFG$_{\text{SURP}^{1}}$	28	1.0000
	PCFG$_{\text{SURP}^{>1}}$ vs. PCFG$_{\text{SURP}^{1}}$	-109	0.3444
	PCFG$_{\text{SURP}^{<1}}$ vs. PCFG$_{\text{SURP}^{\geq 1}}$	**137**	**0.0157**
	PCFG$_{\text{SURP}^{>1}}$ vs. PCFG$_{\text{SURP}^{\leq 1}}$	**-137**	**0.0144**
GPT-2	GPT-2$_{f(\text{SURP})}$ vs. $\emptyset$	**2119**	**0.0005**
	GPT-2$_{f(\text{SURP})}$ vs. GPT-2$_{\text{PROB}}$	**1466**	**0.0005**
	GPT-2$_{f(\text{SURP})}$ vs. GPT-2$_{\text{SURP}^{1/2}}$	3	1.0000
	GPT-2$_{f(\text{SURP})}$ vs. GPT-2$_{\text{SURP}^{3/4}}$	-564	—
	GPT-2$_{f(\text{SURP})}$ vs. GPT-2$_{\text{SURP}^{1}}$	-380	—
	GPT-2$_{f(\text{SURP})}$ vs. GPT-2$_{\text{SURP}^{4/3}}$	-164	—
	GPT-2$_{f(\text{SURP})}$ vs. GPT-2$_{\text{SURP}^{2}}$	**429**	**0.0005**
	GPT-2$_{\text{PROB}}$ vs. $\emptyset$	**654**	**0.0005**
	GPT-2$_{\text{SURP}^{1/2}}$ vs. $\emptyset$	**2116**	**0.0005**
	GPT-2$_{\text{SURP}^{3/4}}$ vs. $\emptyset$	**2684**	**0.0005**
	GPT-2$_{\text{SURP}^{1}}$ vs. $\emptyset$	**2499**	**0.0005**
	GPT-2$_{\text{SURP}^{4/3}}$ vs. $\emptyset$	**2283**	**0.0005**
	GPT-2$_{\text{SURP}^{2}}$ vs. $\emptyset$	**1690**	**0.0005**
	GPT-2$_{\text{SURP}^{1/2}}$ vs. GPT-2$_{\text{PROB}}$	**1462**	**0.0005**
	GPT-2$_{\text{SURP}^{3/4}}$ vs. GPT-2$_{\text{PROB}}$	**2030**	**0.0005**
	GPT-2$_{\text{SURP}^{1}}$ vs. GPT-2$_{\text{PROB}}$	**1846**	**0.0005**
	GPT-2$_{\text{SURP}^{4/3}}$ vs. GPT-2$_{\text{PROB}}$	**1630**	**0.0005**
	GPT-2$_{\text{SURP}^{2}}$ vs. GPT-2$_{\text{PROB}}$	**1036**	**0.0005**
	GPT-2$_{\text{SURP}^{3/4}}$ vs. GPT-2$_{\text{SURP}^{1/2}}$	**568**	**0.0005**
	GPT-2$_{\text{SURP}^{1}}$ vs. GPT-2$_{\text{SURP}^{1/2}}$	**383**	**0.0005**
	GPT-2$_{\text{SURP}^{4/3}}$ vs. GPT-2$_{\text{SURP}^{1/2}}$	**167**	**0.0193**
	GPT-2$_{\text{SURP}^{2}}$ vs. GPT-2$_{\text{SURP}^{1/2}}$	**-426**	**0.0005**
	GPT-2$_{\text{SURP}^{1}}$ vs. GPT-2$_{\text{SURP}^{3/4}}$	**-184**	**0.0126**
	GPT-2$_{\text{SURP}^{4/3}}$ vs. GPT-2$_{\text{SURP}^{3/4}}$	**-400**	**0.0005**
	GPT-2$_{\text{SURP}^{2}}$ vs. GPT-2$_{\text{SURP}^{3/4}}$	**-994**	**0.0005**
	GPT-2$_{\text{SURP}^{4/3}}$ vs. GPT-2$_{\text{SURP}^{1}}$	**-216**	**0.0029**
	GPT-2$_{\text{SURP}^{2}}$ vs. GPT-2$_{\text{SURP}^{1}}$	**-809**	**0.0005**
	GPT-2$_{\text{SURP}^{2}}$ vs. GPT-2$_{\text{SURP}^{4/3}}$	**-593**	**0.0005**
	GPT-2$_{\text{SURP}^{<1}}$ vs. GPT-2$_{\text{SURP}^{1}}$	56	1.0000
	GPT-2$_{\text{SURP}^{>1}}$ vs. GPT-2$_{\text{SURP}^{1}}$	**-535**	**0.0005**
	GPT-2$_{\text{SURP}^{<1}}$ vs. GPT-2$_{\text{SURP}^{\geq 1}}$	**352**	**0.0005**
	GPT-2$_{\text{SURP}^{>1}}$ vs. GPT-2$_{\text{SURP}^{\leq 1}}$	**-580**	**0.0005**

	Comparison	Combined Datasets ΔLL	p
GPT-J	GPT-J$_{f(\text{SURP})}$ vs. $\emptyset$	**1998**	**0.0006**
	GPT-J$_{f(\text{SURP})}$ vs. GPT-J$_{\text{PROB}}$	**671**	**0.0006**
	GPT-J$_{f(\text{SURP})}$ vs. GPT-J$_{\text{SURP}^{1/2}}$	**358**	**0.0006**
	GPT-J$_{f(\text{SURP})}$ vs. GPT-J$_{\text{SURP}^{3/4}}$	**608**	**0.0006**
	GPT-J$_{f(\text{SURP})}$ vs. GPT-J$_{\text{SURP}^{1}}$	**761**	**0.0006**
	GPT-J$_{f(\text{SURP})}$ vs. GPT-J$_{\text{SURP}^{4/3}}$	**236**	**0.0012**
	GPT-J$_{f(\text{SURP})}$ vs. GPT-J$_{\text{SURP}^{2}}$	**733**	**0.0006**
	GPT-J$_{\text{PROB}}$ vs. $\emptyset$	**1328**	**0.0006**
	GPT-J$_{\text{SURP}^{1/2}}$ vs. $\emptyset$	**1641**	**0.0006**
	GPT-J$_{\text{SURP}^{3/4}}$ vs. $\emptyset$	**1390**	**0.0006**
	GPT-J$_{\text{SURP}^{1}}$ vs. $\emptyset$	**1237**	**0.0006**
	GPT-J$_{\text{SURP}^{4/3}}$ vs. $\emptyset$	**1762**	**0.0006**
	GPT-J$_{\text{SURP}^{2}}$ vs. $\emptyset$	**1266**	**0.0006**
	GPT-J$_{\text{SURP}^{1/2}}$ vs. GPT-J$_{\text{PROB}}$	**313**	**0.0006**
	GPT-J$_{\text{SURP}^{3/4}}$ vs. GPT-J$_{\text{PROB}}$	63	1.0000
	GPT-J$_{\text{SURP}^{1}}$ vs. GPT-J$_{\text{PROB}}$	-91	0.7791
	GPT-J$_{\text{SURP}^{4/3}}$ vs. GPT-J$_{\text{PROB}}$	**434**	**0.0006**
	GPT-J$_{\text{SURP}^{2}}$ vs. GPT-J$_{\text{PROB}}$	-62	1.0000
	GPT-J$_{\text{SURP}^{3/4}}$ vs. GPT-J$_{\text{SURP}^{1/2}}$	**-250**	**0.0017**
	GPT-J$_{\text{SURP}^{1}}$ vs. GPT-J$_{\text{SURP}^{1/2}}$	**-404**	**0.0006**
	GPT-J$_{\text{SURP}^{4/3}}$ vs. GPT-J$_{\text{SURP}^{1/2}}$	121	0.4180
	GPT-J$_{\text{SURP}^{2}}$ vs. GPT-J$_{\text{SURP}^{1/2}}$	**-375**	**0.0006**
	GPT-J$_{\text{SURP}^{1}}$ vs. GPT-J$_{\text{SURP}^{3/4}}$	-153	0.1082
	GPT-J$_{\text{SURP}^{4/3}}$ vs. GPT-J$_{\text{SURP}^{3/4}}$	**372**	**0.0006**
	GPT-J$_{\text{SURP}^{2}}$ vs. GPT-J$_{\text{SURP}^{3/4}}$	-125	0.2992
	GPT-J$_{\text{SURP}^{4/3}}$ vs. GPT-J$_{\text{SURP}^{1}}$	**525**	**0.0006**
	GPT-J$_{\text{SURP}^{2}}$ vs. GPT-J$_{\text{SURP}^{1}}$	29	1.0000
	GPT-J$_{\text{SURP}^{2}}$ vs. GPT-J$_{\text{SURP}^{4/3}}$	**-496**	**0.0006**
	GPT-J$_{\text{SURP}^{<1}}$ vs. GPT-J$_{\text{SURP}^{1}}$	**279**	**0.0006**
	GPT-J$_{\text{SURP}^{>1}}$ vs. GPT-J$_{\text{SURP}^{1}}$	**358**	**0.0006**
	GPT-J$_{\text{SURP}^{<1}}$ vs. GPT-J$_{\text{SURP}^{\geq 1}}$	-63	0.7504
	GPT-J$_{\text{SURP}^{>1}}$ vs. GPT-J$_{\text{SURP}^{\leq 1}}$	79	0.4180
GPT-3	GPT-3$_{f(\text{SURP})}$ vs. $\emptyset$	**1443**	**0.0006**
	GPT-3$_{f(\text{SURP})}$ vs. GPT-3$_{\text{PROB}}$	**740**	**0.0006**
	GPT-3$_{f(\text{SURP})}$ vs. GPT-3$_{\text{SURP}^{1/2}}$	**241**	**0.0011**
	GPT-3$_{f(\text{SURP})}$ vs. GPT-3$_{\text{SURP}^{3/4}}$	164	0.0828
	GPT-3$_{f(\text{SURP})}$ vs. GPT-3$_{\text{SURP}^{1}}$	-4	—
	GPT-3$_{f(\text{SURP})}$ vs. GPT-3$_{\text{SURP}^{4/3}}$	**393**	**0.0006**
	GPT-3$_{f(\text{SURP})}$ vs. GPT-3$_{\text{SURP}^{2}}$	-425	—
	GPT-3$_{\text{PROB}}$ vs. $\emptyset$	**703**	**0.0006**
	GPT-3$_{\text{SURP}^{1/2}}$ vs. $\emptyset$	**1202**	**0.0006**
	GPT-3$_{\text{SURP}^{3/4}}$ vs. $\emptyset$	**1279**	**0.0006**
	GPT-3$_{\text{SURP}^{1}}$ vs. $\emptyset$	**1447**	**0.0006**
	GPT-3$_{\text{SURP}^{4/3}}$ vs. $\emptyset$	**1050**	**0.0006**
	GPT-3$_{\text{SURP}^{2}}$ vs. $\emptyset$	**1868**	**0.0006**
	GPT-3$_{\text{SURP}^{1/2}}$ vs. GPT-3$_{\text{PROB}}$	**499**	**0.0006**
	GPT-3$_{\text{SURP}^{3/4}}$ vs. GPT-3$_{\text{PROB}}$	**576**	**0.0006**
	GPT-3$_{\text{SURP}^{1}}$ vs. GPT-3$_{\text{PROB}}$	**743**	**0.0006**
	GPT-3$_{\text{SURP}^{4/3}}$ vs. GPT-3$_{\text{PROB}}$	**347**	**0.0006**
	GPT-3$_{\text{SURP}^{2}}$ vs. GPT-3$_{\text{PROB}}$	**1164**	**0.0006**
	GPT-3$_{\text{SURP}^{3/4}}$ vs. GPT-3$_{\text{SURP}^{1/2}}$	77	1.0000
	GPT-3$_{\text{SURP}^{1}}$ vs. GPT-3$_{\text{SURP}^{1/2}}$	**245**	**0.0011**
	GPT-3$_{\text{SURP}^{4/3}}$ vs. GPT-3$_{\text{SURP}^{1/2}}$	**-152**	**0.0445**
	GPT-3$_{\text{SURP}^{2}}$ vs. GPT-3$_{\text{SURP}^{1/2}}$	**666**	**0.0006**
	GPT-3$_{\text{SURP}^{1}}$ vs. GPT-3$_{\text{SURP}^{3/4}}$	**167**	**0.0445**
	GPT-3$_{\text{SURP}^{4/3}}$ vs. GPT-3$_{\text{SURP}^{3/4}}$	**-229**	**0.0049**
	GPT-3$_{\text{SURP}^{2}}$ vs. GPT-3$_{\text{SURP}^{3/4}}$	**588**	**0.0006**
	GPT-3$_{\text{SURP}^{4/3}}$ vs. GPT-3$_{\text{SURP}^{1}}$	**-397**	**0.0006**
	GPT-3$_{\text{SURP}^{2}}$ vs. GPT-3$_{\text{SURP}^{1}}$	**421**	**0.0006**
	GPT-3$_{\text{SURP}^{2}}$ vs. GPT-3$_{\text{SURP}^{4/3}}$	**818**	**0.0006**
	GPT-3$_{\text{SURP}^{<1}}$ vs. GPT-3$_{\text{SURP}^{1}}$	**-220**	**0.0011**
	GPT-3$_{\text{SURP}^{>1}}$ vs. GPT-3$_{\text{SURP}^{1}}$	**-144**	**0.0317**
	GPT-3$_{\text{SURP}^{<1}}$ vs. GPT-3$_{\text{SURP}^{\geq 1}}$	**-111**	**0.0390**
	GPT-3$_{\text{SURP}^{>1}}$ vs. GPT-3$_{\text{SURP}^{\leq 1}}$	49	1.0000
All LMs	All-LMs$_{f(\text{SURP})}$ vs. $\emptyset$	**1452**	**0.0005**
	All-LMs$_{f(\text{SURP})}$ vs. All-LMs$_{\text{PROB}}$	**773**	**0.0005**
	All-LMs$_{f(\text{SURP})}$ vs. All-LMs$_{\text{SURP}^{1/2}}$	**251**	**0.0005**
	All-LMs$_{f(\text{SURP})}$ vs. All-LMs$_{\text{SURP}^{3/4}}$	**232**	**0.0005**
	All-LMs$_{f(\text{SURP})}$ vs. All-LMs$_{\text{SURP}^{1}}$	-190	—
	All-LMs$_{f(\text{SURP})}$ vs. All-LMs$_{\text{SURP}^{4/3}}$	-51	—
	All-LMs$_{f(\text{SURP})}$ vs. All-LMs$_{\text{SURP}^{2}}$	**184**	**0.0005**
	All-LMs$_{\text{PROB}}$ vs. $\emptyset$	**680**	**0.0005**
	All-LMs$_{\text{SURP}^{1/2}}$ vs. $\emptyset$	**1202**	**0.0005**
	All-LMs$_{\text{SURP}^{3/4}}$ vs. $\emptyset$	**1221**	**0.0005**
	All-LMs$_{\text{SURP}^{1}}$ vs. $\emptyset$	**1643**	**0.0005**
	All-LMs$_{\text{SURP}^{4/3}}$ vs. $\emptyset$	**1504**	**0.0005**
	All-LMs$_{\text{SURP}^{2}}$ vs. $\emptyset$	**1268**	**0.0005**
	All-LMs$_{\text{SURP}^{1/2}}$ vs. All-LMs$_{\text{PROB}}$	**522**	**0.0005**
	All-LMs$_{\text{SURP}^{3/4}}$ vs. All-LMs$_{\text{PROB}}$	**541**	**0.0005**
	All-LMs$_{\text{SURP}^{1}}$ vs. All-LMs$_{\text{PROB}}$	**963**	**0.0005**
	All-LMs$_{\text{SURP}^{4/3}}$ vs. All-LMs$_{\text{PROB}}$	**824**	**0.0005**
	All-LMs$_{\text{SURP}^{2}}$ vs. All-LMs$_{\text{PROB}}$	**589**	**0.0005**
	All-LMs$_{\text{SURP}^{3/4}}$ vs. All-LMs$_{\text{SURP}^{1/2}}$	19	1.0000
	All-LMs$_{\text{SURP}^{1}}$ vs. All-LMs$_{\text{SURP}^{1/2}}$	**441**	**0.0005**
	All-LMs$_{\text{SURP}^{4/3}}$ vs. All-LMs$_{\text{SURP}^{1/2}}$	**302**	**0.0005**
	All-LMs$_{\text{SURP}^{2}}$ vs. All-LMs$_{\text{SURP}^{1/2}}$	67	0.2217
	All-LMs$_{\text{SURP}^{1}}$ vs. All-LMs$_{\text{SURP}^{3/4}}$	**422**	**0.0005**
	All-LMs$_{\text{SURP}^{4/3}}$ vs. All-LMs$_{\text{SURP}^{3/4}}$	**283**	**0.0005**
	All-LMs$_{\text{SURP}^{2}}$ vs. All-LMs$_{\text{SURP}^{3/4}}$	47	0.6588
	All-LMs$_{\text{SURP}^{4/3}}$ vs. All-LMs$_{\text{SURP}^{1}}$	**-139**	**0.0005**
	All-LMs$_{\text{SURP}^{2}}$ vs. All-LMs$_{\text{SURP}^{1}}$	**-375**	**0.0005**
	All-LMs$_{\text{SURP}^{2}}$ vs. All-LMs$_{\text{SURP}^{4/3}}$	**-235**	**0.0005**
	All-LMs$_{\text{SURP}^{<1}}$ vs. All-LMs$_{\text{SURP}^{1}}$	**-422**	**0.0005**
	All-LMs$_{\text{SURP}^{>1}}$ vs. All-LMs$_{\text{SURP}^{1}}$	**-315**	**0.0005**
	All-LMs$_{\text{SURP}^{<1}}$ vs. All-LMs$_{\text{SURP}^{\geq 1}}$	**-240**	**0.0005**
	All-LMs$_{\text{SURP}^{>1}}$ vs. All-LMs$_{\text{SURP}^{\leq 1}}$	-16	1.0000

	Comparison	Combined Datasets ΔLL	p
Between LMs	PCFG$_{f(\text{SURP})}$ vs. n-gram$_{f(\text{SURP})}$	-167	0.0554
	GPT-2$_{f(\text{SURP})}$ vs. n-gram$_{f(\text{SURP})}$	**1178**	**0.0004**
	GPT-J$_{f(\text{SURP})}$ vs. n-gram$_{f(\text{SURP})}$	**1057**	**0.0004**
	GPT-3$_{f(\text{SURP})}$ vs. n-gram$_{f(\text{SURP})}$	**502**	**0.0004**
	GPT-2$_{f(\text{SURP})}$ vs. PCFG$_{f(\text{SURP})}$	**1345**	**0.0004**
	GPT-J$_{f(\text{SURP})}$ vs. PCFG$_{f(\text{SURP})}$	**1224**	**0.0004**
	GPT-3$_{f(\text{SURP})}$ vs. PCFG$_{f(\text{SURP})}$	**669**	**0.0004**
	GPT-J$_{f(\text{SURP})}$ vs. GPT-2$_{f(\text{SURP})}$	-121	0.2212
	GPT-3$_{f(\text{SURP})}$ vs. GPT-2$_{f(\text{SURP})}$	**-676**	**0.0004**
	GPT-3$_{f(\text{SURP})}$ vs. GPT-J$_{f(\text{SURP})}$	**-555**	**0.0004**
	GPT-2PCFG$_{f(\text{SURP})}$ vs. GPT-2$_{f(\text{SURP})}$	17	1.0000
GPT-2 (Normal Error)	GPT-2$_{f(\text{SURP})}$ vs. $\emptyset$	**3088**	**0.0011**
	GPT-2$_{f(\text{SURP})}$ vs. GPT-2$_{\text{PROB}}$	**3305**	**0.0011**
	GPT-2$_{f(\text{SURP})}$ vs. GPT-2$_{\text{SURP}^{1/2}}$	-184	—
	GPT-2$_{f(\text{SURP})}$ vs. GPT-2$_{\text{SURP}^{3/4}}$	82	1.0000
	GPT-2$_{f(\text{SURP})}$ vs. GPT-2$_{\text{SURP}^{1}}$	391	1.0000
	GPT-2$_{f(\text{SURP})}$ vs. GPT-2$_{\text{SURP}^{4/3}}$	524	0.8036
	GPT-2$_{f(\text{SURP})}$ vs. GPT-2$_{\text{SURP}^{2}}$	138	1.0000
	GPT-2$_{\text{PROB}}$ vs. $\emptyset$	-217	—
	GPT-2$_{\text{SURP}^{1/2}}$ vs. $\emptyset$	**3272**	**0.0011**
	GPT-2$_{\text{SURP}^{3/4}}$ vs. $\emptyset$	**3005**	**0.0011**
	GPT-2$_{\text{SURP}^{1}}$ vs. $\emptyset$	**2697**	**0.0011**
	GPT-2$_{\text{SURP}^{4/3}}$ vs. $\emptyset$	**2563**	**0.0011**
	GPT-2$_{\text{SURP}^{2}}$ vs. $\emptyset$	**2950**	**0.0011**
	GPT-2$_{\text{SURP}^{1/2}}$ vs. GPT-2$_{\text{PROB}}$	**3489**	**0.0011**
	GPT-2$_{\text{SURP}^{3/4}}$ vs. GPT-2$_{\text{PROB}}$	**3222**	**0.0011**
	GPT-2$_{\text{SURP}^{1}}$ vs. GPT-2$_{\text{PROB}}$	**2914**	**0.0011**
	GPT-2$_{\text{SURP}^{4/3}}$ vs. GPT-2$_{\text{PROB}}$	**2780**	**0.0011**
	GPT-2$_{\text{SURP}^{2}}$ vs. GPT-2$_{\text{PROB}}$	**3167**	**0.0011**
	GPT-2$_{\text{SURP}^{3/4}}$ vs. GPT-2$_{\text{SURP}^{1/2}}$	-267	1.0000
	GPT-2$_{\text{SURP}^{1}}$ vs. GPT-2$_{\text{SURP}^{1/2}}$	-575	0.3404
	GPT-2$_{\text{SURP}^{4/3}}$ vs. GPT-2$_{\text{SURP}^{1/2}}$	-708	0.2477
	GPT-2$_{\text{SURP}^{2}}$ vs. GPT-2$_{\text{SURP}^{1/2}}$	-322	1.0000
	GPT-2$_{\text{SURP}^{1}}$ vs. GPT-2$_{\text{SURP}^{3/4}}$	-309	1.0000
	GPT-2$_{\text{SURP}^{4/3}}$ vs. GPT-2$_{\text{SURP}^{3/4}}$	-442	1.0000
	GPT-2$_{\text{SURP}^{2}}$ vs. GPT-2$_{\text{SURP}^{3/4}}$	-56	1.0000
	GPT-2$_{\text{SURP}^{4/3}}$ vs. GPT-2$_{\text{SURP}^{1}}$	-133	1.0000
	GPT-2$_{\text{SURP}^{2}}$ vs. GPT-2$_{\text{SURP}^{1}}$	253	1.0000
	GPT-2$_{\text{SURP}^{2}}$ vs. GPT-2$_{\text{SURP}^{4/3}}$	386	1.0000
	GPT-2$_{\text{SURP}^{<1}}$ vs. GPT-2$_{\text{SURP}^{1}}$	425	0.6558
	GPT-2$_{\text{SURP}^{>1}}$ vs. GPT-2$_{\text{SURP}^{1}}$	128	1.0000
	GPT-2$_{\text{SURP}^{<1}}$ vs. GPT-2$_{\text{SURP}^{\geq 1}}$	397	0.4839
	GPT-2$_{\text{SURP}^{>1}}$ vs. GPT-2$_{\text{SURP}^{\leq 1}}$	-216	1.0000
	GPT-2$^{(\text{EXGAUSS})}_{f(\text{SURP})}$ vs. GPT-2$^{(\text{NORMAL})}_{f(\text{SURP})}$	**284442**	**0.0005**
Prob vs. Surp	GPT-2$_{\text{PROB}+\text{SURP}^{1}}$ vs. GPT-2$_{\text{PROB}}$	**2240**	**0.0001**
	GPT-2$_{\text{PROB}+\text{SURP}^{1}}$ vs. GPT-2$_{\text{SURP}^{1}}$	**394**	**0.0001**

Table S9. Testing Results Across All Datasets. Results of key statistical comparisons based on permutation tests of the difference in average test set likelihood between the alternative and null model in each comparison (ΔLL), aggregating all dependent variables considered in this study in each test (see Supplementary Information 13 for details). Subscripts indicate the assumed functional form of predictability effects: $f(\text{SURP})$ (nonlinear in surprisal), PROB (linear in probability), and SURP^{a} (linear in surprisal raised to exponent a, such that e.g., SURP^{1} is linear in surprisal). Boldface indicates statistical significance. In directional tests where one hypothesis subsumes the other (e.g., $f(\text{SURP})$ vs. SURP^{1}, since a nonlinear surprisal effect subsumes a linear one), dashes (—) indicate failure of the alternative hypothesis (left) to improve over the null hypothesis (right). Otherwise, cells are color coded using cyan to indicate that the hypothesis on the left outperforms the one on the right, and using magenta to indicate that the hypothesis on the right outperforms the one on the left. All p-values are corrected for false discovery rate (15) within each family of tests (delimited by single horizontal lines). Key comparisons are highlighted.

5. Full IRF Surface Plots

Figures S5–**S6** provide detailed IRF estimates across language models and datasets. In these three-dimensional surface plots, the change in the dependent variable (e.g., reading time) is shown on the z-axis as a function of both surprisal (x-axis) and delay (in seconds) from stimulus onset (y-axis). These surface plots provide timecourse details that are important for interpretation of some results. For example, **Figure 2** of the main article shows a null effect (with a puzzling negative slope) of PCFG surprisal on first pass reading times of the GECO corpus. This finding becomes less puzzling in the context of **Figure S5**, which makes clear that the estimated effect of PCFG surprisal on first pass reading times in GECO is still positive overall, but simply late-onset, peaking around 250ms after the word is first fixated. This estimated timecourse is an outlier: for most datasets, language models, and response types, the instantaneous surprisal effect (at delay 0) is positive. We avoid further interpretation of this finding in light of the fact that the PCFG is a weaker language model than the others considered in this study, both in terms of perplexity (**Table S10**) and psychometric performance (**Figure 3** of the main article). Related work has explored better-performing incremental parsers as language models (56), a research question that was not a central focus of this study.

Figure S7 plots the estimated IRFs for GPT-2 probability (**Figure S7a**) vs. GPT-2 surprisal (**Figure S7b**) in a model containing strictly linear terms for both of these predictors. This configuration addresses the hypothesis that linear and logarithmic effects (deriving from distinct cognitive processes) may be superposed (57) by allowing the regression model to find a mixture between the linear (probability) and logarithmic (surprisal) terms. As shown, the estimated mixture heavily favors surprisal over probability. GPT-2 surprisal effect estimates (**Figure S7b**) have relatively large magnitude and show the expected positive association predicted by the COST view. By comparison, GPT-2 probability effect estimates (**Figure S7a**) have small magnitude and high uncertainty, and they do not consistently show the expected negative association predicted by the FACILITATION view; indeed more often than not effects trend numerically in the wrong direction, with higher probability words eliciting longer reading times. We thus find no systematic support for superposed linear and logarithmic predictability effects.

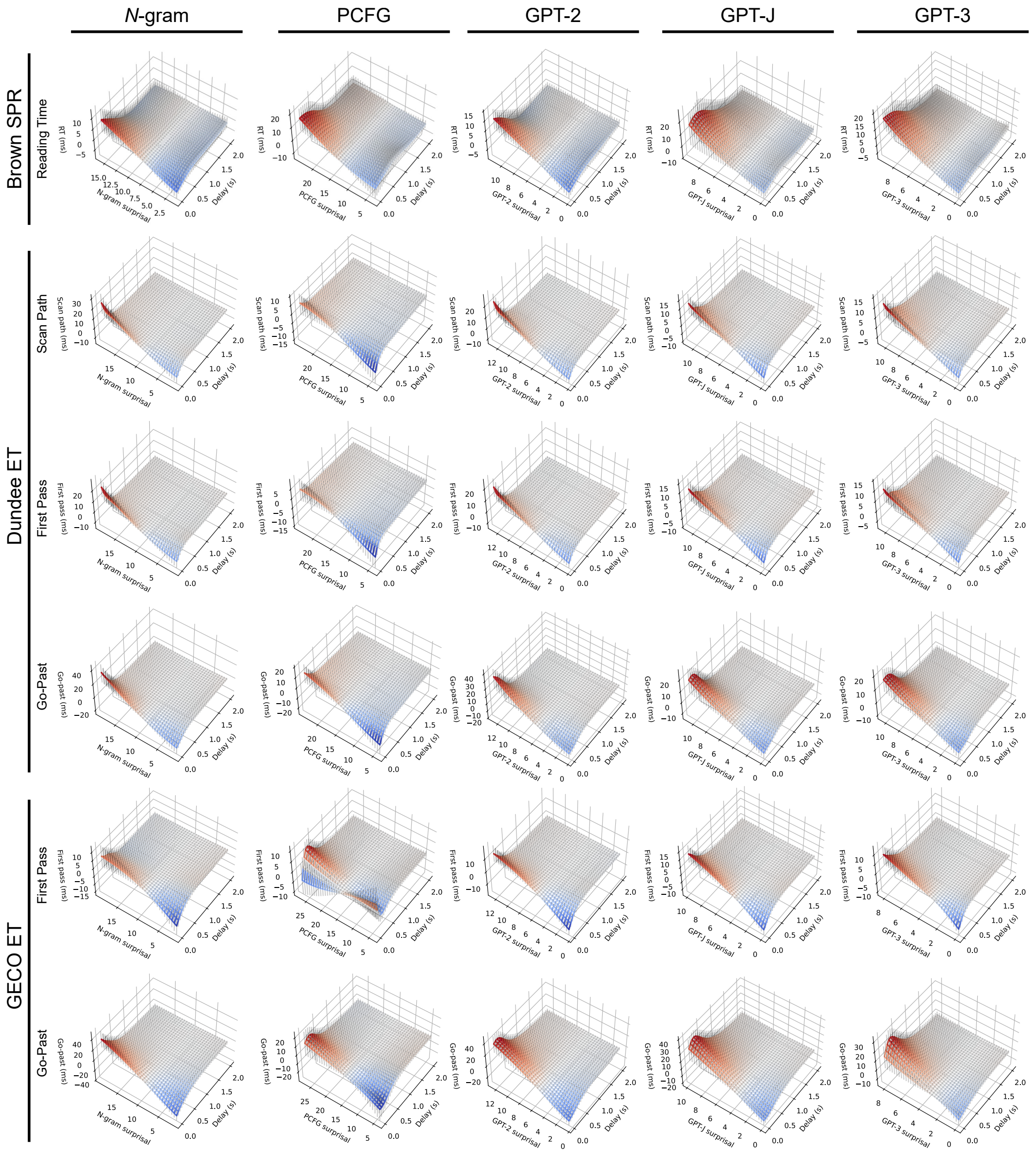

Fig. S5. CDRNN-estimated functional form of effect estimates in the Brown, Dundee, and GECO datasets across language models over a 2s interval following initial fixation. Each subplot shows the estimated change in the dependent variable (vertical axis) from observing a word with a given surprisal value (left axis) at a given delay (in seconds) from word onset (right axis). Gray error bars indicate 95% variational Bayesian credible intervals.

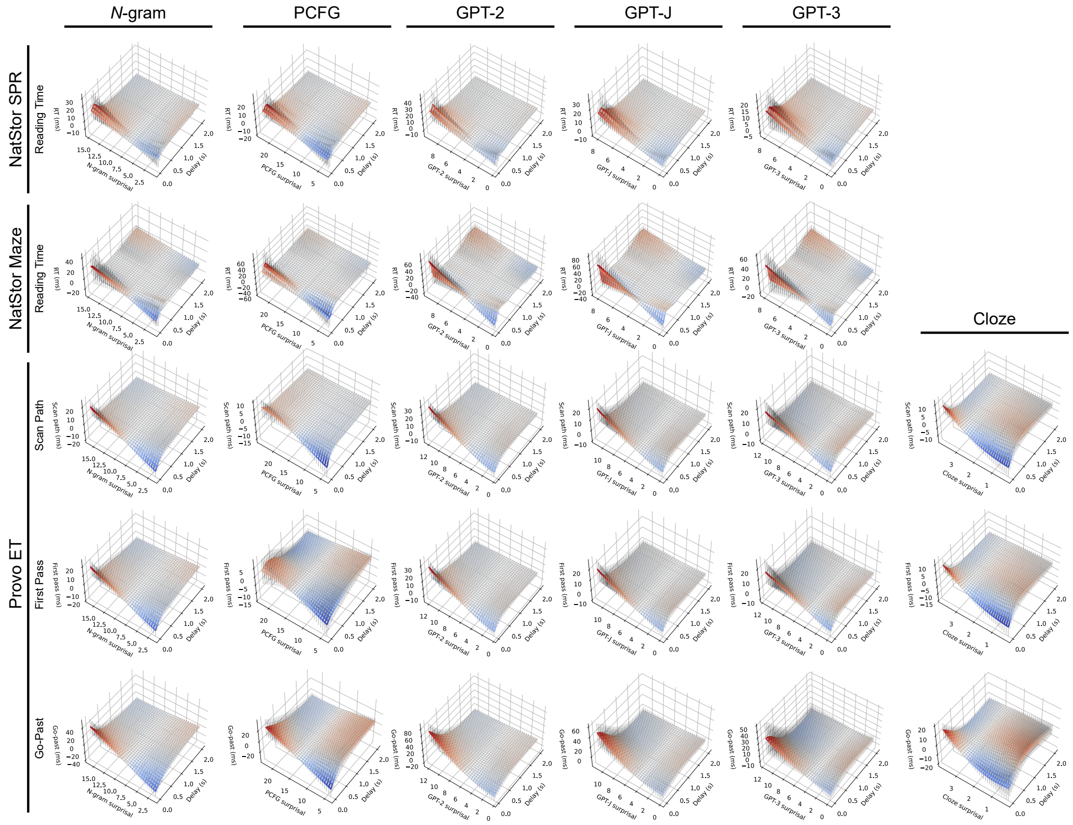

Fig. S6. CDRNN-estimated functional form of effect estimates in the Natural Stories SPR, Natural Stories Maze, and Provo daetasets across language models over a 2s interval following initial fixation. Each subplot shows the estimated change in the dependent variable (vertical axis) from observing a word with a given surprisal value (left axis) at a given delay (in seconds) from word onset (right axis). Gray error bars indicate 95% variational Bayesian credible intervals.

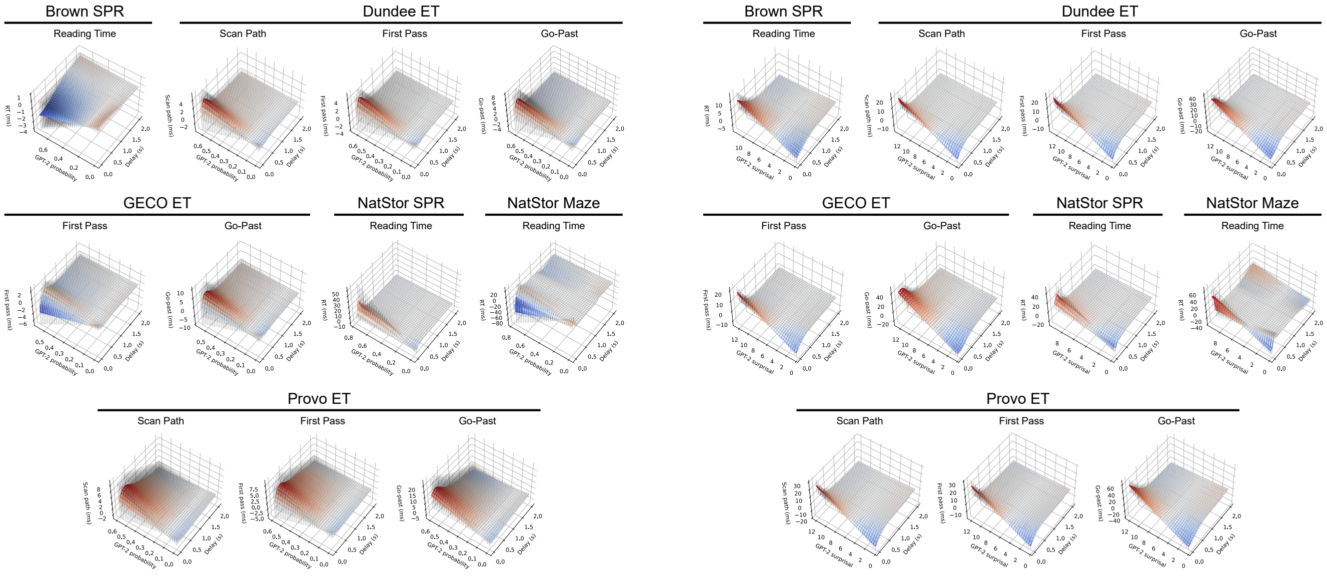

(a) Estimated GPT-2 probability effects across datasets

(b) Estimated GPT-2 surprisal effects across datasets

Fig. S7. CDRNN-estimated impulse responses to GPT-2 probability (a) vs. GPT-2 surprisal (b) across datasets, under a model containing strictly linear terms for both GPT-2 probability and GPT-2 surprisal. Each subplot shows the estimated change in the dependent variable (vertical axis) from observing a word with a given probability/surprisal value (left axis) at a given delay (in seconds) from word onset (right axis). Gray error bars indicate 95% variational Bayesian credible intervals. Note that, whereas reading times consistently increase on word surprisal near word onset in panel (b), they do not consistently decrease on word predictability in panel (a). Instead, in some datasets (e.g., Natural Stories SPR), more probable words are estimated to lead to an *increase* in reading time.

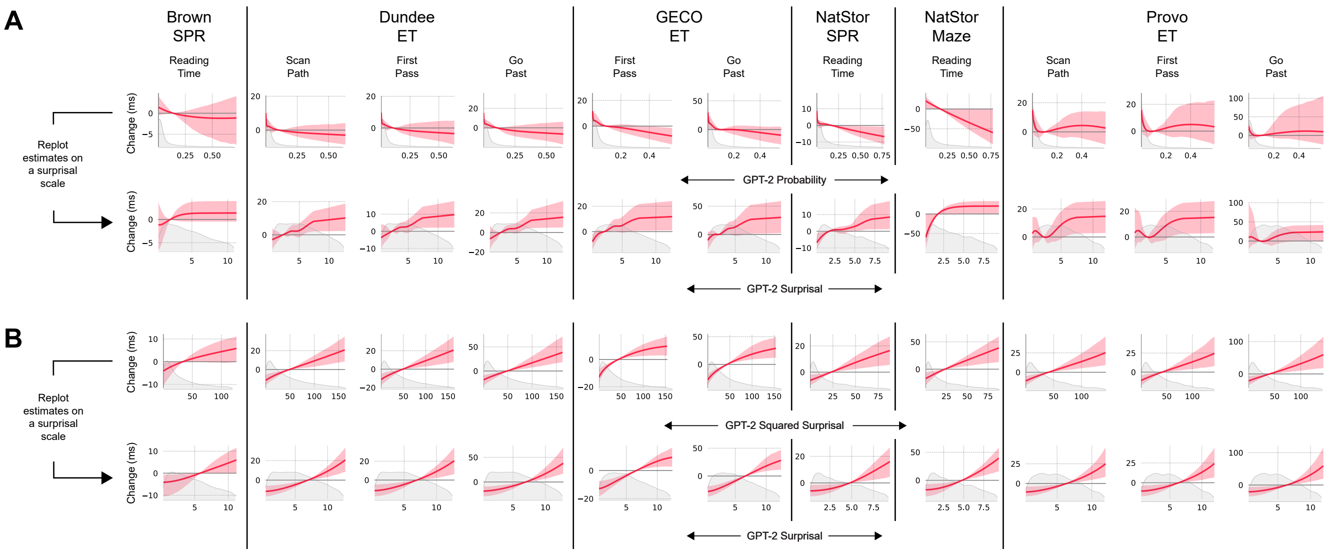

Fig. S8. Inversion of predictability effects toward surprisal. The top row of each panel shows CDRNN estimated predictability effects on a (non-logarithmic) source scale (probability or squared surprisal) from which the CDRNN model estimates the shape and scale of a continuous function relating word predictability to processing cost (reading time). The bottom row of each panel shows the same estimated function (from the same models) replotted on a surprisal scale. Effects are shown with no delay (i.e. at the surprising word). **A.** Estimates from models using GPT-2 probability to represent word predictability. A logarithmic effect should be realized as a sharp increase in reading time as probability drops toward zero in the top row of the panel. When the resulting estimated function is replotted on a surprisal scale (bottom row of the panel), it should resemble a straight line. This prediction is largely borne out visually, especially in the largest datasets (Dundee ET, GECO ET, and Natural Stories SPR). With the exception of Natural Stories Maze, models do not find the linear effect (straight line with a negative slope in the top row of the panel) predicted by the faciilitation view. **B.** Estimates from models using squared GPT-2 surprisal to represent word predictability. A logarithmic effect should be realized as decreasing slope the reading time effect as squared surprisal increases in the top row of the panel. When the resulting estimated function is replotted on a surprisal scale (bottom row of the panel), it should resemble a straight line. This prediction is borne out in some cases (e.g., Brown SPR and GECO ET), but not in others. In other words, a logarithmic effect is generally not (fully) recovered from a superlogarithmic source scale. Nonetheless, direct statistical comparisons favor a logarithmic effect over a superlogarithmic one overall (**Figure 3**).

6. Direct Comparison between GPT-2 and PCFG Language Models

We show in the main article that GPT-2 substantially outperforms PCFG language models an estimator of human processing difficulty. Nonetheless, recent evidence indicates that surprisal derived from incremental generative parsers like our PCFG may contain signal about human language processing that is not fully captured by GPT-2 surprisal (56), despite the capacity of large neural network language models to acquire some syntactic information (e.g., 58, 59). To test this possibility, we compared our GPT-2 $f(\textsc{surp})$ CDRNN models to comparable models augmented with PCFG surprisal, which allows us to quantify the contribution of PCFG surprisal to model fit over GPT-2 surprisal alone. Results do not clearly support a generalized contribution of PCFGs over GPT-2. Adding PCFG surprisal to a model containing GPT-2 surprisal does not provide a significant performance boost across datasets (**Table S9**) and is only significant in 2/11 comparisons on individual datasets. Notably, one of these is the Natural Stories SPR dataset, which has previously been used to argue for complementary PCFG effects over GPT-2 (56). Although we do not find evidence in these analyses of unique contributions of PCFGs over transformer networks as psychometric models, more sophisticated generative incremental parsers are known to afford substantial psychometric gains over GPT-2 models (56). Our lack of findings in favor of unique PCFG effects should therefore not be construed as evidence against the sensitivity of human surprisal to syntactic factors (see also e.g., 60, 61).

7. Are Logarithmic Predictability Effects Recovered from a Non-Logarithmic Scale?

If CDRNN models learn to *invert* a predictability measure defined on a non-logarithmic scale into one that is logarithmic, this would provide convergent evidence for logarithmic predictability effects. To investigate this, we fit nonlinear CDRNNs equipped with either GPT-2 probability (linear in predictability) or squared GPT-2 surprisal (superlogarithmic in predictability) and evaluate the degree to which the model pushes the estimated predictability effect toward a logarithmic shape (i.e., linear in surprisal). We refer to these models respectively as $f(\textsc{prob})$ and $f(\textsc{surp}^2)$. Note that recovering a logarithmic surprisal scale from a linear probability scale is a challenging estimation problem for neural networks (like CDRNNs), which repeatedly modify their weights in small increments during estimation, subject to penalties on large-magnitude weights. This is because the model must find a probability-cost curve that rises sharply (to asymptotic infinity) over a small numerical range (probabilities approximately in the range $0 < p < 0.05$). The converse is not true: recovering a linear probability scale from a logarithmic surprisal scale is straightforward, since the model must simply find a "plateau" whereby cost increases little at large values of surprisal (**Figure 1**). To address this asymmetry, we substantially reduce the regularizer strength in these analyses (by a factor of 500), which harms generalization performance but permits more flexible estimation of nonlinearities. Results are plotted in **Figure S8**. As shown, CDRNNs tend to estimate a highly nonlinear effect of GPT-2 probability that suggests an inordinate cost associated with low-probability items across datasets, contrary to the FACILITATION view. When these estimates

are replotted on a surprisal scale, they look roughly linear in the Dundee, GECO, and Natural Stories SPR datasets, which are substantially larger than the others and plausibly provide more reliable estimates.

Results using squared surprisal are less clear. In some cases, the squaring function is inverted to an effect that is linear on surprisal (Brown and GECO), and in others not. Note however that this is a similar distribution of models that found visually linear vs. superlinear estimates for GPT-2 surprisal in Figure 2, and, as discussed above, these estimated superlinearities do not lead systematically to improved fit. In sum, models tend to find a logarithmic predictability effect, even when starting from predictability measures that do not represent predictability logarithmically.

8. Comparison to Hoover et al. (2023)

Here we provide an extended discussion of Hoover et al. (5), a closely-related study that reached qualitatively different conclusions about the functional form of predictability effects. Hoover et al. make two key contributions, one theoretical and one empirical. Their key theoretical contribution is to derive superlogarithmic predictability effects from sampling algorithms as models of language comprehension, thus providing a mechanistic account for how UID pressures might arise from processing constraints. Their key empirical contribution is to show superlogarithmic estimates of word predictability effects (consistent with UID) in the Natural Stories SPR dataset (also analyzed in this study), especially for large language models like GPT-3.

The methods in Hoover et al. are rigorous, using generalized additive mixed models for location and scale (11, 62, 63) with spillover regressors from preceding words (64) to estimate and control for delayed, nonlinear, and heteroscedastic patterns of influence of word predictability on reading times, and using 6-fold cross-validation to insure against high-leverage outliers. Nevertheless, our approach offers some advantages. *First*, we consider a broader range of datasets (six) and reading modalities (three), which allows us to avoid drawing conclusions from patterns that may be idiosyncratic to a given dataset or modality. *Second*, our exGaussian CDRNN models are less assumption-laden, allowing for continuous-time effect delays (rather than indexical delays over word positions, which are implausible for naturalistic reading; 65, 66), arbitrary predictor interactions, nonstationarity in the response function, and control over skewness in the distribution over reading times, which plays a major role in the Natural Stories SPR dataset (our exGaussian model improves over a comparable Normal model on Natural Stories SPR by over 120,000 log likelihood points; **Table S9**). *Third*, our claims are based on the generalization performance of pretrained regression models, rather than on visual estimates (or descriptive statistics derived from visual estimates, as in Hoover et al.). This aspect of our design offers critical insurance against non-replication, since in-sample tests from highly expressive models applied to large datasets may be prone to finding small but significant effects that may not hold in general, especially when analyses are concentrated on estimates from the empirical tail (a small number of high-surprisal words). Although Hoover et al.'s cross-validation procedure offers some protection against this concern, generalization performance is not evaluated, and 5/6 of the training data (used to estimate the critical surprisal effects) is shared across any pair of folds.

As discussed in the main article, our procedures nonetheless replicate key aspects of the Hoover et al. findings on Natural Stories SPR. *First*, the effect estimates tend to be superlogarithmic across language models (**Figure 2** of the main article). This impression is even more pronounced when considering the full empirical range of surprisal values in the training data (**Figure S9**), as reported in Hoover et al., rather than the interdecile range reported in the main article. Natural Stories SPR shows a visually pronounced superlogarithmic pattern at the highest levels of surprisal. *Second*, these superlogarithmic estimates in Natural Stories SPR confer performance advantages: in aggregate, f(SURP) outperforms SURP^{1}, and SURP$^{4/3}$ (but not SURP^{2}) also outperforms SURP^{1}. This outcome is reassuring, since different research teams using different methods converge in large part on the same result when analyzing the same dataset.

However, our approach allows us to zoom out to a broader picture that is considerably less favorable to superlogarithmic effects, and which suggests that the Natural Stories SPR dataset may be an outlier. The other datasets show little evidence of superlogarithmic patterns, even at the highest values of surprisal (**Figure S9**). In addition, as reported in the main article, over all datasets, SURP^{1} outperforms SURP$^{4/3}$, SURP^{2}, and SURP$^{>1}$ (the ensemble of SURP$^{4/3}$ and SURP^{2}), and SURP$^{>1}$ does not outperform SURP$^{\leq 1}$ (the ensemble of SURP$^{1/2}$ and SURP$^{3/4}$, and SURP^{1}). This pattern holds both when we consider all language models in aggregate and when we focus on the best-performing model overall (GPT-2). Furthermore, this general picture is unchanged when we revisit our key questions using GAMs, as in Hoover et al., instead of CDRNNs (**SI 14**).

One could potentially argue that Natural Stories SPR has a privileged status relative to the other datasets, and that results using it should be given greater weight. In particular, it is the largest of the datasets considered here (>1M datapoints), and it was specifically designed to tax the language processing system using rare words and syntactic constructions that plausibly increase overall surprisal (67). Perhaps this makes it a better testbed for questions about predictability effects relative to the five other datasets we considered (which used naturally occurring written texts, and which may therefore under-represent effects in the high surprisal regime). We find the following problems with this argument. *First*, the texts in Natural Stories are not in fact more surprising overall than those in the other datasets. Across language models, surprisal values in Natural Stories are among the lowest in our sample, whether considering overall model perplexity (i.e., exponentiated average surprisal, **Table S10**), the interdecile range (**Figure 2** of the main article), the total range (**Figure S9**), or the surprisal densities within either of these intervals. There is thus little empirical support for the notion that these materials are better for investigating the extremes of the surprisal continuum. *Second*, the Natural Stories Maze dataset used the same materials in a different sample of participants under a different reading modality, but Natural Stories Maze shows no evidence of superlogarithmicity, either in terms of estimates (**Figure S9**) or model performance (if anything, the best-performing models are sublogarithmic; **Figure 3** of the main article). Thus, the Natural Stories SPR patterns do not appear to derive from the textual materials, but are instead specific to the particular sample and/or modality (self-paced reading). *Third*, it is known that responses in

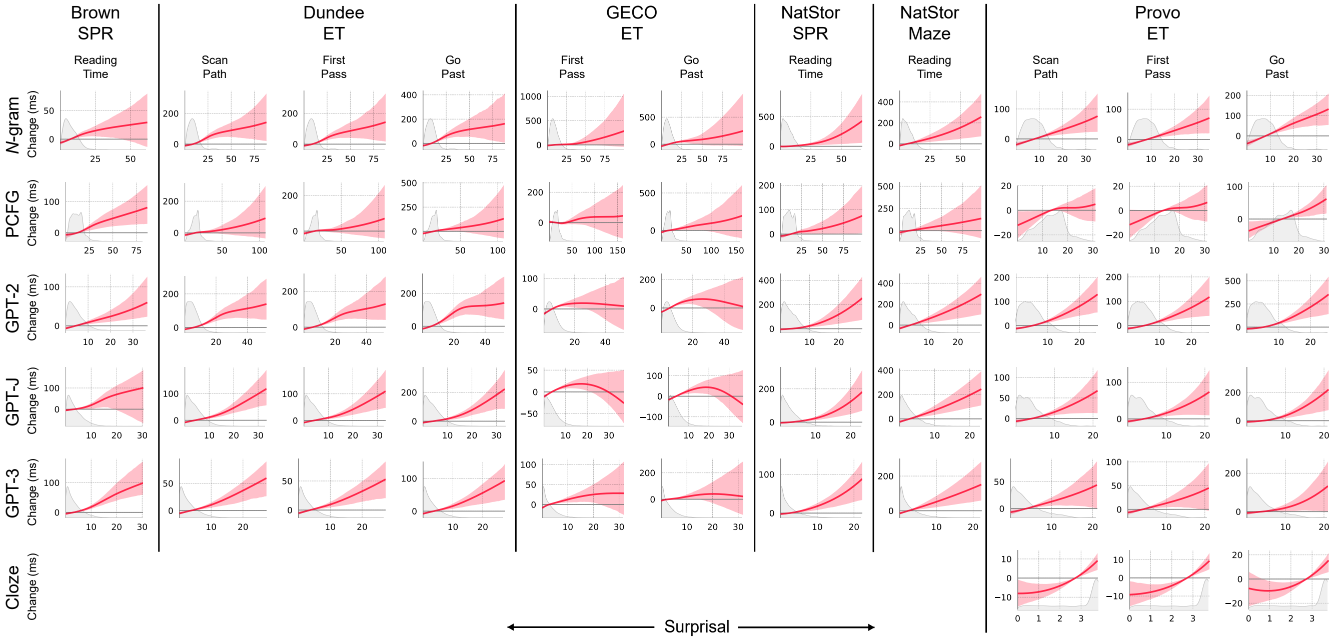

Fig. S9. CDRNN-estimated functional form of effects across language model types (*n*-gram, PCFG, GPT-2, GPT-J, GPT-3, and human cloze) with no delay (i.e. at the surprising word). Plots cover the full empirical range of surprisal values in each training dataset. Kernel density plots show the distribution of surprisal values in the training data over the plotted range.

Natural Stories SPR show strong effects of continuous reading rate that are much larger in magnitude than linguistic effects like word frequency or surprisal (65, 66). These rate effects are plausibly driven by motor habituation, rather than language processing demand (65, 66), and they cannot be estimated using discrete-time methods like those in Hoover et al. Studies that control for rate effects in Natural Stories SPR show that linguistic effects can be still detected (65, 66), but that they are faint relative to more ecologically valid modalities like eye-tracking. *Fourth*, unlike the other datasets (except Natural Stories Maze), Natural Stories SPR was crowdsourced on Amazon Mechanical Turk and may consequently have issues with quality control that may not be present in the other data. For example, one participant fixated a word for over an hour, and another participant had an average reaction time of 35ms, far faster than the time generally needed to develop and execute a motor plan (for button pressing), let alone process the meanings of words. Although these extreme cases are removed through outlier filtering, it is unclear how indicative they may be about broader quality issues in crowd-sourced data that may not always be caught by outlier filters. For example, the participant with the 35ms average reading time still managed to get an average of 6/8 comprehension questions correct, enough to pass quality checks for any of their reading times that happen to exceed the 100ms minimum filter. These issues are unlikely to arise in an in-person laboratory setting.

Consistent with the third and fourth concerns above, our results show large variation in model performance on Natural Stories SPR relative to the other datasets, despite its size (**Figure 3** of the main article), which suggests weak signal. This is not to say Natural Stories SPR is bad data; as we said above, it has major advantages, and prior work has supported the existence of linguistic effects in that dataset. For these reasons, we have chosen to include it in our own analyses. Our point is only that, like all datasets, Natural Stories SPR has both strengths and weaknesses, and we do not think that it should be privileged relative to the other datasets in our sample, especially given the clearer signals about predictive processing that emerge using other datasets. In summary, we believe the full pattern of results in our study suggests that the superlogarithmic effects reported by Hoover et al. may be idiosyncratic to the dataset they analyzed, rather than being characteristic of reading in general.

Up to this point, we have focused on points of disagreement between our study and Hoover et al., but these points should be considered against a larger context of similarity. Both studies concur that predictability effects in natural reading are *at least* logarithmic and thus agree with the COST (cf., FACILITATION) view that probabilistic inference is a major concern of the human language processing system. Where they differ is with respect to the presence or absence of additional pressures favoring uniform information density, over and above a set of shared assumptions about the underlying inferential processes. Furthermore, as discussed in the main article, our design cannot falsify the UID view, only constrain the strength of superlogarithmic patterns. For example, our results indicate that SURP^2 may be too strongly superlogarithmic. Nonetheless, there remains an infinite space of superlogarithmic functions that are consistent with our pattern of results. We hope this work will encourage the development of experimental and analytical innovations that could shed additional light on this question.

9. Language Model Perplexity

Table S10 presents language model perplexities by dataset.

Dataset	Perplexity				
	n-gram	PCFG	GPT-2	GPT-J	GPT-3
Brown	1560	6379	98	32	23
Dundee	1142	4028	74	28	20
GECO	1702	6444	86	23	10
Natural Stories	645	4216	43	18	12
Provo	796	3172	72	32	23

Table S10. Language model perplexity (exponentiated average surprisal) by model type and dataset.

10. Statistical Controls

We include the following control predictors in all models:

- **Rate.** A "deconvolutional intercept" (65) describing the average response to a word, independent of its properties. *Rate* is so named because its influence on the response depends solely on stimulus timing.
- **Word length.** The length of the word (in characters).
- **Unigram surprisal.** A "context-free" surprisal driven by the relative frequency of the word. Unigram surprisal was obtained from a KenLM unigram model (68) trained on the Gigaword 3 corpus (20).
- **End of sentence.** Whether a word ends a sentence (binary indicator), designed to capture diffuse effects of sentence boundaries (e.g., 69, 70), even though the final words of sentences themselves are excluded from analysis (see above).

Models of eye-tracking datasets additionally contained the following control predictors that are specifically relevant to the eye-tracking modality:

- **Saccade length.** Length in words of incoming saccade (eye movement).
- **Regression.** Whether the fixation is part of a regressive (backward) eye movement (binary indicator).

Since the Dundee corpus additionally provides annotations for screen and line boundaries, we included these as regressors in Dundee models only:

- **End of line.** Whether a word ends a line of text on the display (binary indicator).
- **End of screen.** Whether a word ends a screen on the display (binary indicator).

Finally, the Maze task used in Natural Stories Maze involves a potentially errorful word-by-word forced choice task. Therefore, for this dataset alone, we modeled the possibility of effects from task errors using the following regressor:

- **Incorrect.** Whether the incorrect continuation was chosen in the A-Maze task (binary indicator).

The CDRNN models used here flexibly capture interactions between any combinations of these variables (66). Thus, by including e.g., the *regression* predictor, the models can learn not only overall differences in response to regressive vs. non-regressive fixations, but also e.g., differences in surprisal or word length effects between regressive vs. non-regressive fixations. This ability is important because fixations during regressive eye movements plausibly differ in their processing demands, since they involve material that was likely already viewed either foveally or parafoveally.

11. Model Formulae

For ease of reference, here we present the CDRNN model formulae used throughout this study. Variable names are changed from those used in our codebase for readability. For full software implementation details, see https://github.com/coryshain/cdr. Because the sets of control predictors differ across datasets, we abbreviate "$\text{control}_1 + \ldots + \text{control}_n$" (e.g., "WordLength + SaccadeLength + EndOfSentence + ...") as "CONTROLS". Control variables used for each dataset are described in **SI 10**. The keywords "Surprisal" and "Probability" respectively represent surprisal- and probability-scale estimates from a given language model.

- **∅:** y ∼ C(CONTROLS, NN()) + (C(CONTROLS, NN(ran=T)) | Participant) + (1 | DocumentID:SentenceID:WordPositionInSentence)
- **f(surp):** y ∼ C(CONTROLS + Surprisal, NN()) + (C(CONTROLS + Surprisal, NN(ran=T)) | Participant) + (1 | DocumentID:SentenceID:WordPositionInSentence)
- **f(prob):** y ∼ C(CONTROLS + Probability, NN()) + (C(CONTROLS + Probability, NN(ran=T)) | Participant) + (1 | DocumentID:SentenceID:WordPositionInSentence)
- **$f(\text{surp}^2)$:** y ∼ C(CONTROLS + Surprisal^2, NN()) + (C(CONTROLS + Surprisal^2, NN(ran=T)) | Participant) + (1 | DocumentID:SentenceID:WordPositionInSentence)
- **prob:** y ∼ C(CONTROLS + Probability, NN(inputs_to_drop=[Probability])) + (C(CONTROLS + Probability, NN(inputs_to_drop=[Probability], ran=T)) | Participant) + (1 | DocumentID:SentenceID:WordPositionInSentence)
- **$\text{surp}^{1/2}$:** y ∼ C(CONTROLS + $\text{Surprisal}^{1/2}$, NN(inputs_to_drop=[$\text{Surprisal}^{1/2}$])) + (C(CONTROLS + $\text{Surprisal}^{1/2}$, NN(inputs_to_drop=[$\text{Surprisal}^{1/2}$], ran=T)) | Participant) + (1 | DocumentID:SentenceID:WordPositionInSentence)
- **$\text{surp}^{3/4}$:** y ∼ C(CONTROLS + $\text{Surprisal}^{3/4}$, NN(inputs_to_drop=[$\text{Surprisal}^{3/4}$])) + (C(CONTROLS + $\text{Surprisal}^{3/4}$, NN(inputs_to_drop=[$\text{Surprisal}^{3/4}$], ran=T)) | Participant) + (1 | DocumentID:SentenceID:WordPositionInSentence)

- **surp^{1}:** y ~ C(CONTROLS + Surprisal^1, NN(inputs_to_drop=[Surprisal^1])) + (C(CONTROLS + Surprisal^1, NN(inputs_to_drop=[Surprisal^1], ran=T)) | Participant) + (1 | DocumentID:SentenceID:WordPositionInSentence)
- **surp$^{4/3}$:** y ~ C(CONTROLS + Surprisal$^{4/3}$, NN(inputs_to_drop=[Surprisal$^{4/3}$])) + (C(CONTROLS + Surprisal$^{4/3}$, NN(inputs_to_drop=[Surprisal$^{4/3}$], ran=T)) | Participant) + (1 | DocumentID:SentenceID:WordPositionInSentence)
- **surp^{2}:** y ~ C(CONTROLS + Surprisal^2, NN(inputs_to_drop=[Surprisal^2])) + (C(CONTROLS + Surprisal^2, NN(inputs_to_drop=[Surprisal^2], ran=T)) | Participant) + (1 | DocumentID:SentenceID:WordPositionInSentence)
- **GPT-2+PCFG$_{f(\text{surp})}$:** C(CONTROLS + GPT2Surprisal + PCFGSurprisal, NN()) + (C(CONTROLS + GPT2Surprisal + PCFGSurprisal, NN(ran=T)) | Participant) + (1 | DocumentID:SentenceID:WordPositionInSentence)

12. CDRNN Model Definition

For ease of reference, here we reproduce the formal definition of the CDRNN model from ref. (66). Let $\mathbf{y} \in \mathbb{R}^Y$ be a Y-dimensional random variable that we seek to model (the response). Let $\mathcal{F}$ be a probability distribution with S-dimensional parameter vector $\mathbf{s} \in \mathbb{R}^S$ such that $\mathbf{y} \sim \mathcal{F}(\mathbf{s})$. Let $\mathbf{X} \in \mathbb{R}^{N\times K}$ be a matrix of N K-dimensional predictor vectors $\mathbf{x}_n, 1 \le n \le N$. Let $t_\mathbf{y} \in \mathbb{R}$ be the timestamp of $\mathbf{y}$, and let $\mathbf{t} \in \mathbb{R}^N$ be the vector of predictor timestamps $t_{\mathbf{x}_1}, ..., t_{\mathbf{x}_n}$ such that $t_{\mathbf{x}_n}$ is the timestamp of $\mathbf{x}_n$. Let $\mathbf{d} \in \mathbb{R}^N$ then be the vector of temporal offsets $d_{\mathbf{x}_1}, ..., d_{\mathbf{x}_n}$ such that $d_{\mathbf{x}_n} = t_\mathbf{y} - t_{\mathbf{x}_n}$, i.e. the signed distance in time between $\mathbf{y}$ and $\mathbf{x}_n$.*

The timestamps $\mathbf{t}$ are horizontally concatenated with $\mathbf{X}$ to yield the inputs to $f_\text{in} \in \mathbb{R}^{N\times(K+1)} \to \mathbb{R}^{N\times J}$ with parameters $\mathbf{u}_\text{in}$. f_in is an input processing function that yields $\mathbf{X}' \in \mathbb{R}^{N\times J}$, a matrix of J-dimensional impulse vectors $\mathbf{x}'_n, 1 \le n \le N$:

$$\mathbf{X}' \stackrel{\text{def}}{=} f_\text{in}\left(\begin{bmatrix}\mathbf{t} & \mathbf{X}\end{bmatrix}; \mathbf{u}_\text{in}\right) \quad [1]$$

$\mathbf{X}'$ is horizontally concatenated with $\mathbf{d}$ and $\mathbf{t}$ to yield the inputs to IRF $f_\text{IRF} \in \mathbb{R}^{N\times(J+2)} \to \mathbb{R}^{N\times S\times(J+1)}$ with parameters $\mathbf{u}_\text{IRF}$. The output of the IRF is a sequence of convolution weight matrices $\mathbf{G}_n \in \mathbb{R}^{S\times(J+1)}, 1 \le n \le N$:

$$\mathbf{G}_1, \ldots, \mathbf{G}_N \stackrel{\text{def}}{=} f_\text{IRF}\left(\begin{bmatrix}\mathbf{d} & \mathbf{t} & \mathbf{X}'\end{bmatrix}; \mathbf{u}_\text{IRF}\right) \quad [2]$$

The final outputs of the model—parameters $\mathbf{s}$ of $\mathcal{F}$—are computed as the sum of (i) the temporal convolution of $\mathbf{X}'$ with $\mathbf{G}_1, \ldots, \mathbf{G}_N$ and (ii) learned bias vector $\mathbf{s_0}$, where each transposed row $\mathbf{x}'_n, 1 \le n \le N$ of $\mathbf{X}'$ is vertically concatenated with a bias† and weighted by learned coefficient vector $\mathbf{b} \in \mathbb{R}^{J+1}$:

$$\mathbf{s} \stackrel{\text{def}}{=} \mathbf{s_0} + \sum_{n=1}^{N} \mathbf{G}_n \text{diag}(\mathbf{b}) \begin{bmatrix} 1 \\ \mathbf{x}'_n \end{bmatrix} \quad [3]$$

Letting $\mathbf{v} \in \mathbb{R}^V$ represent the concatenation of $\mathbf{u}_\text{in}$, $\mathbf{u}_\text{IRF}$, $\mathbf{b}$, and $\mathbf{s_0}$

$$\mathbf{v} \stackrel{\text{def}}{=} \begin{bmatrix} \mathbf{u}_\text{in} \\ \mathbf{u}_\text{IRF} \\ \mathbf{b} \\ \mathbf{s_0} \end{bmatrix} \quad [4]$$

mixed effects models can be defined by letting $\mathbf{v}$ be the sum of a fixed part $\mathbf{v}_0 \in \mathbb{R}^V$ and random part, where $\mathbf{V} \in \mathbb{R}^{V\times Z}$ is a random effects matrix whose rows sum to 0 and $\mathbf{z} \in \{0,1\}^Z$ indicates which of Z random effects levels apply to $\mathbf{y}$:

$$\mathbf{v} = \mathbf{v}_0 + \mathbf{V}\mathbf{z} \quad [5]$$

The parameters of the model are therefore $\mathbf{v}_0$ and $\mathbf{V}$, which may be fitted via maximum likelihood or (given priors) Bayesian inference. This definition assumes a singleton dataset $\mathcal{D} = \{\langle \mathbf{X}, \mathbf{t}, \mathbf{y}, t_\mathbf{y}\rangle\}$, but it extends without loss of generality to any finite dataset by applying eq. 3 independently to each of M elements in $\mathcal{D} = \{\langle \mathbf{X}_m, \mathbf{t}_m, \mathbf{y}_m, t_{\mathbf{y}_m}\rangle \mid 1 \le m \le M\}$.

In this study, following ref. (66), f_in is identity, and f_IRF is a feedforward neural network; i.e., a network consisting solely of linear transformations followed by a nonlinearity—in our case, the GELU function (71). To enforce a linear effect for the kth predictor dimension, f_IRF is preceded by a mask $\mathbf{f}^{(k)} \in \{0,1\}^K$ such that each of its rows is defined as:

$$\mathbf{f}_i^{(k)} \stackrel{\text{def}}{=} \begin{cases} 1 & i \ne k \\ 0 & \text{otherwise} \end{cases}$$

This removes the kth predictor from the inputs to the IRF, but retains it in the convolution defined in eq. 3, thereby preventing the IRF from conditioning on the predictor's value and enforcing a linear effect.

*We note that, in this work, we never use future stimuli for predicting $\mathbf{y}$, so in practice $t_\mathbf{y} \ge t_{\mathbf{x}_n}$ and all $d_{\mathbf{x}_n}$ will be non-negative.

†The bias term, referred to here and in ref. (65) as *rate*, serves as a deconvolutional "intercept" capturing general effects of event timing.

13. CDRNN Implementation and Statistical Procedure

Unless otherwise indicated, all CDRNNs were implemented as described in ref. (66) with the following parameter settings:

- Feedforward IRF with two hidden layers of 32 units each.
- Full random effects (zero-centered deviations in the model intercepts, linear coefficients, and layerwise bias terms permitting variation in IRF shape) by subject and random intercepts by token.
- Dropout rate (72) of 0.1 on (a) all hidden layers and (b) random grouping factor variables.
- L2 weight regularization constant of 5.
- L2 regularization constant of 10 on random IRF bias terms.
- To speed convergence, prior to fitting, response variables are z-scored and predictor variables are rescaled by their standard deviations.
- Fixed intercepts and coefficients assume a standard normal prior, and, following ref. (65), random intercepts and coefficients assume a normal prior with mean 0 and standard deviation 0.1. Variational posteriors over these parameters are estimated using variational expectation maximization.
- For computational efficiency, histories are truncated at 32 words or 60s into the past, whichever is shorter.
- Convergence is diagnosed based on a time-loss correlation criterion, where the loss is the validation set likelihood evaluated every 10 epochs and the correlation is computed over a window of 250 consecutive epochs. Thus, convergence is declared whenever the validation set likelihood is statistically non-increasing at $\alpha = 0.5$ for at least 13 of the preceding 25 evaluations. For full details about this procedure, see ref. (65). Following convergence, the model state with the best validation set performance is used for all evaluation and visualization.

Full code and model configuration files needed for reproduction are provided at https://github.com/coryshain/cdr. Hypotheses are statistically evaluated on the test set, with separate tests for each response variable (scan path, first pass, and go-past durations in Dundee and reading time in Natural Stories).

In this study, null hypotheses assume a linear effect of some fixed function of word predictability (e.g., surprisal), and the alternative hypothesis is a non-linear effect. To enforce linearity, dependencies to predictors are removed from impulse response functions, which prevents the network from adapting its convolution weights to the value of the predictor, resulting in a strictly linear effect. We present the specific formulae used to define our CRDNN models in **SI 11**. For formal definition of the CDRNN model, see **SI 12**.

Following ref. (66), in order to account for optimization noise in the statistical tests, we statistically compare *ensembles* of 10 model replicates per hypothesis, using a hierarchical paired permutation test inspired by ref. (73). In some cases, we combine ensembles to test *composite* hypotheses that encompass multiple simple hypotheses in our design. For example, given our design spanning five different exponents on surprisal (1/2, 3/4, 1, 4/3, and 2) for each language model, the hypothesis that predictability effects are superlogarithmic is a composite test in which exponents 1/2, 3/4, and 1 together define the composite null hypothesis and exponents 4/3 and 2 together define the composite alternative hypothesis. For such questions, we ensemble all models fitted using each composite hypothesis, resulting (in the example above) in a null model with 30 replicates (3×10) and an alternative model with 20 replicates (2×10).

Tests use the following procedure, in which A is the ensemble size for hypothesis $\mathcal{A}$ and B is the ensemble size for hypothesis $\mathcal{B}$:

1. For each of the N evaluation items $1 \leq n \leq N$, repartition the $A + B$ log-likelihood statistics into two random sets of likelihoods $\hat{\mathcal{A}}_n \in \mathbb{R}^A$, $\hat{\mathcal{B}}_n \in \mathbb{R}^B$.
2. Compute the resampled dataset likelihood as the median of summed likelihoods within the resampled partition: $\mathcal{L}_{\hat{\mathcal{A}}} = \underset{1 \leq a \leq A}{\text{med}} \left[\sum_{n=1}^{N} \hat{\mathcal{A}}_{n,a}\right]$, $\mathcal{L}_{\hat{\mathcal{B}}} = \underset{1 \leq b \leq B}{\text{med}} \left[\sum_{n=1}^{N} \hat{\mathcal{B}}_{n,b}\right]$
3. Compute and store the absolute difference $|\mathcal{L}_{\mathcal{A}} - \mathcal{L}_{\mathcal{B}}|$.

This process is repeated many (10,000) times to construct an empirical null distribution over the likelihood differences between ensembles, which is then compared to the observed difference in mean likelihood between ensembles in order to compute a p value. Because tests are based on out-of-sample performance, alternative models are not guaranteed to outperform null models. Thus, for directional hypotheses in which the alternative subsumes the null (e.g., testing $f(\text{SURP})$ against SURP, since the former's solution space includes the latter), cases where test set likelihood *degrades* in the alternative model relative to the null model are assigned a default p value of 1.

In some cases, we combine all response variables from all datasets in order to test comparisons across the entire set. To do so, given D dataset-response pairs (in this study, $D = 11$) with M total datapoints between them, we vertically concatenate the item-wise likelihood matrices into joint likelihood matrices $\mathcal{A}^{(\text{all})} \in \mathbb{R}^{M\times A}, \mathcal{B}^{(\text{all})} \in \mathbb{R}^{M\times B}$ as follows:

$$\mathcal{A}^{(\text{all})} = \begin{bmatrix} \mathcal{A}^{(1)} \\ \vdots \\ \mathcal{A}^{(M)} \end{bmatrix}, \mathcal{B}^{(\text{all})} = \begin{bmatrix} \mathcal{B}^{(1)} \\ \vdots \\ \mathcal{B}^{(M)} \end{bmatrix}$$

These combined likelihood matrices serve as inputs to the testing procedure outlined above.

All visualizations aggregate across the entire ensemble using 1,000 bootstrap resampling iterations. In each iteration, an ensemble component (i.e., a CDRNN fit) is sampled uniformly, then a model is sampled from that component's variational posterior, then the sampled model is queried with respect to the estimate of interest. This procedure jointly takes into account uncertainty in the posterior of each CDRNN fit as well as uncertainty across the ensemble.

14. Revisiting Our Main Findings Using Generalized Additive Models (GAMs)

The generalized additive model (GAM; 11, 62) is the statistical method of choice in related work on the functional form of predictability effects in reading (e.g., 1–3, 5) because it permits inference about the functional form of the predictability-cost relationship, rather than requiring an assumed form. However, for reasons discussed in the **Introduction** to the main article and elaborated in ref. (66), GAMs as standardly implemented (e.g., in the `mgcv` package, 11) retain a number of problematic simplifying assumptions for the reading domain, including discrete-time (spillover) rather than continuous effect delays, constant error, and (barring multidimensional interaction smooths that are difficult to estimate in practice) additive effects. For these reasons, we have chosen to use continuous-time deconvolutional regressive neural network (CDRNN) models for our key analyses, since they allow us to relax all of these assumptions in a data-driven manner (66), thereby reducing the likelihood that results depend critically on poor correspondence between aspects of model design and aspects of the underlying cognitive process. However, to clarify the extent to which results depend on this modeling choice, here we revisit our key analyses using GAMs instead of CDRNNs.

In so doing, we attempt to match the design of the GAM models to that of our main CDRNN models as closely as possible. With some exceptions noted below, all predictors and random effects from a given CDRNN model were included in the corresponding GAM model. In addition, although GAMs cannot estimate continuous-time impulse response functions, we provide them with some ability to detect effect delays by including two additional spillover positions for each predictor. For example, to model word length effects, GAM models include as predictors both the length of the current word and the lengths of the two preceding words. Given that predictability effects are represented by three distinct predictors in GAM models, all three of these predictors are removed from the baseline ($\emptyset$) GAM model (since this corresponds most closely to the ablation used in the main CDRNN analyses, where predictability effects are not modeled in the baseline at any delay). All predictors are modeled using thin-plate splines with default bases except for boolean indicators like *End of sentence* (which only take two values and thus do not support nonlinear regression) and constrained terms like $GPT\text{-}2_{\text{PROB}}$, both of which are modeled as linear. We apply the same hypothesis testing paradigm to GAM models as we do to CDRNNs (permutation testing of the likelihood difference statistic on the held-out test set). We also apply the same exclusion criteria to the training and evaluation data for GAMs as we did for CDRNNs.

That said, practical considerations led to the following deviations from the CDRNN design:

- Both by-token random intercepts and by-participant random splines led to out-of-memory errors on our compute resource, and, as a result, models with these terms could not be fitted. Therefore, GAM models only contain by-participant random intercepts and by-participant random slopes for each fixed effect in a given model, allowing the magnitude (but not the shape) of the response to each variable to vary by participant.

- Although CDRNNs implicitly estimate interactions between all subsets of variables, estimating such rich multidimensional smooths using GAMs (via tensor-product smooths) is intractable for these datasets. Therefore, predictors are assumed to combine additively in all GAM models.

- Whenever sentence, line, and/or screen starts and ends are excluded from analysis, the *End of sentence*, *End of line*, and/or *End of screen* predictors have no variance both *in situ* and in spillover position 1 (which corresponds to the start of the next sentence, line, or screen, hence also excluded). Therefore, only spillover position 2 is considered for these boundary predictors.

- Because items with incorrect responses are excluded from analysis of the Natural Stories Maze dataset, the *Incorrect* predictor has no variance *in situ*. Therefore, only spilled over variants of *Incorrect* are considered.

- Because first pass and go-past durations by definition exclude words fixated as part of a regressive eye movement, the *Regression* predictor has no variance (in any spillover position) for first pass and go-past durations and is therefore excluded from all models.

- Because GAMs lack the capacity for continuous-time deconvolution, they cannot estimate the *Rate* predictor (65, 66). Therefore, *Rate* is excluded from all models.
- Because (unlike our CDRNN models) GAMs do not rely on early stopping for convergence, no validation set is needed. Therefore, GAMs are fitted to the training and validation sets together. The test set remains the same as that used for CDRNNs.
- In rare cases, numerical imprecision led to `NaN` likelihoods for some datapoints during test-set prediction from GAMs (this never occurred with CDRNN models). Any datapoint assigned such a likelihood by either model in a given statistical comparison was excluded prior to performing the permutation test. This led to exclusion of at most two datapoints in any given test.
- Unlike CDRNNs, GAM fitting is deterministic. Therefore, only a single GAM is fitted to each model configuration (rather than the ensemble of 10 CDRNN models used in the main analyses).

The GAM-estimated predictability-cost functions at no delay (i.e., at the surprising word) are plotted in **Figure S10**. Although estimates differ those produced by CDRNNs (**Figure S9**), this is to be expected given important design differences between the two modeling approaches. Nonetheless, the overall visual impression remains similar to that supported by our main finding: estimates are generally consistent with a logarithmic predictability (linear surprisal) effect (especially at lower surprisal values where the datapoints are concentrated) without a systematic trend either toward the plateau predicted by the FACILITATION view or the superlogarithmic pattern predicted by the UID view: some estimates look more sublogarithmic (e.g., the GPT-2 surprisal effect on Dundee scan path durations), others look more superlogarithmic (e.g., the GPT-2 surprisal effect on Provo scan path durations), and others simply look logarithmic (e.g., the GPT-J surprisal effect on GECO go-past durations). Thus, replacing CDRNNs with GAMs does not lead to systematic differences in the theoretical conclusions suggested visually by model estimates.

Full statistical testing results for our main comparisons using GAM models are given in **Tables S11–S15**. In general, comparisons using GAM models reject the null hypothesis less frequently than comparisons using CDRNN models, suggesting lower sensitivity. For example, no comparison is significant in the Provo dataset (**Table S14**). However, in aggregate, comparisons using GAM models on the held-out test set largely accord with those of our main CDRNN-based findings (**Table S15**): across all datasets and language models, (i) models containing predictability effects significantly outperform the baseline containing no predictability effect, (ii) the strictly logarithmic SURP^1 model is one of the best performing models, (iii) the logarithmic SURP^1 model significantly outperforms the linear PROB model, and (iv) the superlogarithmic $\text{SURP}^{4/3}$ and SURP^2 models do not significantly outperform the SURP^1 model. The key difference from our main findings is that GAM models do not show a significant improvement of the SURP^1 model over the $\text{SURP}^{3/4}$ and SURP^2 models, whereas CDRNN models show significant improvements over both. Thus, the GAM results decide less clearly between the predictions of the COST and UID views of predictability effects. This difference aside, these reanalyses show that, despite their limitations, GAMs largely reproduce our key findings, suggesting that the conclusions we advocate are not critically dependent on the use of CDRNNs rather than GAMs.

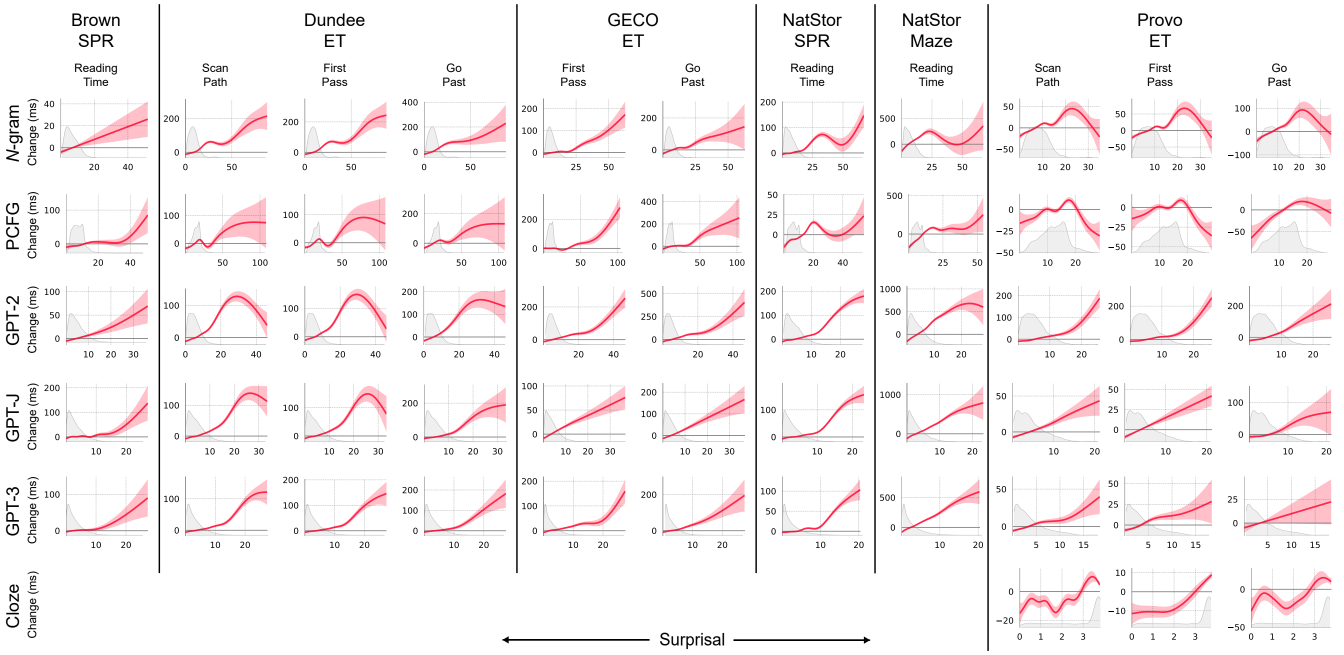

Fig. S10. GAM-estimated functional form of effects across language model types (*n*-gram, PCFG, GPT-2, GPT-J, GPT-3, and human cloze) with no delay (i.e. at the surprising word). Plots cover the full empirical range of surprisal values in each training dataset (and are thus most comparable to the CDRNN plots in **Figure S9**, rather than **Figure 2** of the main article, which uses the interdecile range). Kernel density plots show the distribution of surprisal values in the training data over the plotted range. Uncertainty intervals show the $\pm$ 2 standard errors used as a default in plots from `mgcv` (unlike CDRNN plots, which show 95% credible intervals).

	Comparison	Brown (SPR)		NatStor (SPR)		NatStor (Maze)	
		ΔLL	p	ΔLL	p	ΔLL	p
n-gram	n-gram$_{f(\text{SURP})}$ vs. $\emptyset$	14	0.8920	**324**	**0.0008**	**136**	**0.0008**
	n-gram$_{f(\text{SURP})}$ vs. n-gram$_{\text{PROB}}$	13	0.9155	**257**	**0.0008**	**73**	**0.0032**
	n-gram$_{f(\text{SURP})}$ vs. n-gram$_{\text{SURP}^{1/2}}$	6	0.8920	**91**	**0.0071**	7	1.0000
	n-gram$_{f(\text{SURP})}$ vs. n-gram$_{\text{SURP}^{3/4}}$	3	0.8920	**78**	**0.0015**	18	0.1716
	n-gram$_{f(\text{SURP})}$ vs. n-gram$_{\text{SURP}^{1}}$	0	0.9155	**78**	**0.0008**	**34**	**0.0027**
	n-gram$_{f(\text{SURP})}$ vs. n-gram$_{\text{SURP}^{4/3}}$	-3	—	**96**	**0.0008**	**60**	**0.0008**
	n-gram$_{f(\text{SURP})}$ vs. n-gram$_{\text{SURP}^{2}}$	-2	—	**183**	**0.0008**	**129**	**0.0008**
	n-gram$_{\text{PROB}}$ vs. $\emptyset$	2	1.0000	**67**	**0.0008**	**63**	**0.0008**
	n-gram$_{\text{SURP}^{1/2}}$ vs. $\emptyset$	9	1.0000	**233**	**0.0008**	**129**	**0.0008**
	n-gram$_{\text{SURP}^{3/4}}$ vs. $\emptyset$	11	0.9155	**246**	**0.0008**	**118**	**0.0008**
	n-gram$_{\text{SURP}^{1}}$ vs. $\emptyset$	14	0.8920	**246**	**0.0008**	**102**	**0.0016**
	n-gram$_{\text{SURP}^{4/3}}$ vs. $\emptyset$	17	0.8920	**228**	**0.0008**	**76**	**0.0082**
	n-gram$_{\text{SURP}^{2}}$ vs. $\emptyset$	17	0.8920	141	0.0833	6	1.0000
	n-gram$_{\text{SURP}^{1/2}}$ vs. n-gram$_{\text{PROB}}$	7	1.0000	**166**	**0.0008**	**67**	**0.0037**
	n-gram$_{\text{SURP}^{3/4}}$ vs. n-gram$_{\text{PROB}}$	10	1.0000	**180**	**0.0008**	**55**	**0.0298**
	n-gram$_{\text{SURP}^{1}}$ vs. n-gram$_{\text{PROB}}$	13	0.8920	**179**	**0.0008**	39	0.2960
	n-gram$_{\text{SURP}^{4/3}}$ vs. n-gram$_{\text{PROB}}$	16	0.8920	**161**	**0.0008**	13	1.0000
	n-gram$_{\text{SURP}^{2}}$ vs. n-gram$_{\text{PROB}}$	15	0.8920	74	1.0000	-56	0.2378
	n-gram$_{\text{SURP}^{3/4}}$ vs. n-gram$_{\text{SURP}^{1/2}}$	3	0.8920	13	0.6592	**-12**	**0.0390**
	n-gram$_{\text{SURP}^{1}}$ vs. n-gram$_{\text{SURP}^{1/2}}$	6	0.8920	13	1.0000	**-27**	**0.0069**
	n-gram$_{\text{SURP}^{4/3}}$ vs. n-gram$_{\text{SURP}^{1/2}}$	9	0.8920	-5	1.0000	**-53**	**0.0008**
	n-gram$_{\text{SURP}^{2}}$ vs. n-gram$_{\text{SURP}^{1/2}}$	8	1.0000	-93	0.4058	**-123**	**0.0008**
	n-gram$_{\text{SURP}^{1}}$ vs. n-gram$_{\text{SURP}^{3/4}}$	3	0.8920	0	1.0000	**-16**	**0.0027**
	n-gram$_{\text{SURP}^{4/3}}$ vs. n-gram$_{\text{SURP}^{3/4}}$	6	0.8920	-18	1.0000	**-42**	**0.0008**
	n-gram$_{\text{SURP}^{2}}$ vs. n-gram$_{\text{SURP}^{3/4}}$	5	1.0000	-106	0.1105	**-111**	**0.0008**
	n-gram$_{\text{SURP}^{4/3}}$ vs. n-gram$_{\text{SURP}^{1}}$	3	0.8920	-18	0.7056	**-26**	**0.0008**
	n-gram$_{\text{SURP}^{2}}$ vs. n-gram$_{\text{SURP}^{1}}$	2	1.0000	**-105**	**0.0195**	**-96**	**0.0008**
	n-gram$_{\text{SURP}^{2}}$ vs. n-gram$_{\text{SURP}^{4/3}}$	-1	1.0000	**-87**	**0.0021**	**-70**	**0.0008**
PCFG	PCFG$_{f(\text{SURP})}$ vs. $\emptyset$	**58**	**0.0037**	**125**	**0.0011**	**77**	**0.0008**
	PCFG$_{f(\text{SURP})}$ vs. PCFG$_{\text{PROB}}$	**56**	**0.0037**	**117**	**0.0011**	**64**	**0.0008**
	PCFG$_{f(\text{SURP})}$ vs. PCFG$_{\text{SURP}^{1/2}}$	**28**	**0.0037**	**77**	**0.0011**	14	0.2406
	PCFG$_{f(\text{SURP})}$ vs. PCFG$_{\text{SURP}^{3/4}}$	**25**	**0.0063**	**71**	**0.0011**	**23**	**0.0159**
	PCFG$_{f(\text{SURP})}$ vs. PCFG$_{\text{SURP}^{1}}$	**25**	**0.0037**	**66**	**0.0011**	**33**	**0.0014**
	PCFG$_{f(\text{SURP})}$ vs. PCFG$_{\text{SURP}^{4/3}}$	**26**	**0.0037**	**64**	**0.0011**	**45**	**0.0008**
	PCFG$_{f(\text{SURP})}$ vs. PCFG$_{\text{SURP}^{2}}$	**35**	**0.0037**	**75**	**0.0011**	**60**	**0.0008**
	PCFG$_{\text{PROB}}$ vs. $\emptyset$	2	0.5367	**8**	**0.0195**	**13**	**0.0008**
	PCFG$_{\text{SURP}^{1/2}}$ vs. $\emptyset$	**30**	**0.0086**	**47**	**0.0034**	**63**	**0.0008**
	PCFG$_{\text{SURP}^{3/4}}$ vs. $\emptyset$	**33**	**0.0090**	**54**	**0.0034**	**54**	**0.0014**
	PCFG$_{\text{SURP}^{1}}$ vs. $\emptyset$	**33**	**0.0110**	**59**	**0.0011**	**44**	**0.0185**
	PCFG$_{\text{SURP}^{4/3}}$ vs. $\emptyset$	**31**	**0.0173**	**61**	**0.0011**	32	0.1563
	PCFG$_{\text{SURP}^{2}}$ vs. $\emptyset$	22	0.1686	**50**	**0.0011**	16	0.7371
	PCFG$_{\text{SURP}^{1/2}}$ vs. PCFG$_{\text{PROB}}$	**28**	**0.0090**	**40**	**0.0110**	**50**	**0.0014**
	PCFG$_{\text{SURP}^{3/4}}$ vs. PCFG$_{\text{PROB}}$	**31**	**0.0069**	**46**	**0.0051**	**41**	**0.0183**
	PCFG$_{\text{SURP}^{1}}$ vs. PCFG$_{\text{PROB}}$	**31**	**0.0144**	**51**	**0.0051**	31	0.1645
	PCFG$_{\text{SURP}^{4/3}}$ vs. PCFG$_{\text{PROB}}$	**29**	**0.0268**	**53**	**0.0034**	19	0.7977
	PCFG$_{\text{SURP}^{2}}$ vs. PCFG$_{\text{PROB}}$	21	0.2627	**42**	**0.0089**	3	1.0000
	PCFG$_{\text{SURP}^{3/4}}$ vs. PCFG$_{\text{SURP}^{1/2}}$	2	0.6107	6	0.1539	**-9**	**0.0079**
	PCFG$_{\text{SURP}^{1}}$ vs. PCFG$_{\text{SURP}^{1/2}}$	3	1.0000	12	0.1545	**-19**	**0.0008**
	PCFG$_{\text{SURP}^{4/3}}$ vs. PCFG$_{\text{SURP}^{1/2}}$	1	1.0000	13	0.4656	**-31**	**0.0008**
	PCFG$_{\text{SURP}^{2}}$ vs. PCFG$_{\text{SURP}^{1/2}}$	-8	1.0000	2	1.0000	**-46**	**0.0008**
	PCFG$_{\text{SURP}^{1}}$ vs. PCFG$_{\text{SURP}^{3/4}}$	0	1.0000	5	0.2809	**-10**	**0.0008**
	PCFG$_{\text{SURP}^{4/3}}$ vs. PCFG$_{\text{SURP}^{3/4}}$	-1	1.0000	7	1.0000	**-22**	**0.0008**
	PCFG$_{\text{SURP}^{2}}$ vs. PCFG$_{\text{SURP}^{3/4}}$	-10	0.4491	-4	1.0000	**-37**	**0.0008**
	PCFG$_{\text{SURP}^{4/3}}$ vs. PCFG$_{\text{SURP}^{1}}$	-2	1.0000	2	1.0000	**-12**	**0.0008**
	PCFG$_{\text{SURP}^{2}}$ vs. PCFG$_{\text{SURP}^{1}}$	-11	0.1675	-10	1.0000	**-27**	**0.0019**
	PCFG$_{\text{SURP}^{2}}$ vs. PCFG$_{\text{SURP}^{4/3}}$	**-9**	**0.0268**	-11	0.2283	**-15**	**0.0110**

	Comparison	Brown (SPR)		NatStor (SPR)		NatStor (Maze)	
		ΔLL	p	ΔLL	p	ΔLL	p
GPT-2	GPT-2$_{f(\text{SURP})}$ vs. $\emptyset$	**75**	**0.0007**	**708**	**0.0005**	**715**	**0.0006**
	GPT-2$_{f(\text{SURP})}$ vs. GPT-2$_{\text{PROB}}$	**72**	**0.0007**	**578**	**0.0005**	**462**	**0.0006**
	GPT-2$_{f(\text{SURP})}$ vs. GPT-2$_{\text{SURP}^{1/2}}$	**29**	**0.0007**	**263**	**0.0005**	**83**	**0.0006**
	GPT-2$_{f(\text{SURP})}$ vs. GPT-2$_{\text{SURP}^{3/4}}$	**18**	**0.0031**	**188**	**0.0005**	26	0.0607
	GPT-2$_{f(\text{SURP})}$ vs. GPT-2$_{\text{SURP}^{1}}$	9	0.0760	**132**	**0.0005**	1	1.0000
	GPT-2$_{f(\text{SURP})}$ vs. GPT-2$_{\text{SURP}^{4/3}}$	0	1.0000	**83**	**0.0005**	10	1.0000
	GPT-2$_{f(\text{SURP})}$ vs. GPT-$_{\text{SURP}^{2}}$	-6	—	**74**	**0.0084**	**140**	**0.0006**
	GPT-2$_{\text{PROB}}$ vs. $\emptyset$	3	1.0000	**130**	**0.0005**	**253**	**0.0006**
	GPT-2$_{\text{SURP}^{1/2}}$ vs. $\emptyset$	**46**	**0.0007**	**445**	**0.0005**	**632**	**0.0006**
	GPT-2$_{\text{SURP}^{3/4}}$ vs. $\emptyset$	**58**	**0.0007**	**519**	**0.0005**	**689**	**0.0006**
	GPT-2$_{\text{SURP}^{1}}$ vs. $\emptyset$	**67**	**0.0007**	**576**	**0.0005**	**714**	**0.0006**
	GPT-2$_{\text{SURP}^{4/3}}$ vs. $\emptyset$	**75**	**0.0007**	**624**	**0.0005**	**705**	**0.0006**
	GPT-$_{\text{SURP}^{2}}$ vs. $\emptyset$	**82**	**0.0007**	**634**	**0.0005**	**575**	**0.0006**
	GPT-2$_{\text{SURP}^{1/2}}$ vs. GPT-2$_{\text{PROB}}$	**43**	**0.0007**	**315**	**0.0005**	**379**	**0.0006**
	GPT-2$_{\text{SURP}^{3/4}}$ vs. GPT-2$_{\text{PROB}}$	**54**	**0.0007**	**390**	**0.0005**	**436**	**0.0006**
	GPT-2$_{\text{SURP}^{1}}$ vs. GPT-2$_{\text{PROB}}$	**63**	**0.0007**	**446**	**0.0005**	**461**	**0.0006**
	GPT-2$_{\text{SURP}^{4/3}}$ vs. GPT-2$_{\text{PROB}}$	**72**	**0.0007**	**495**	**0.0005**	**452**	**0.0006**
	GPT-$_{\text{SURP}^{2}}$ vs. GPT-2$_{\text{PROB}}$	**79**	**0.0007**	**504**	**0.0005**	**322**	**0.0006**
	GPT-2$_{\text{SURP}^{3/4}}$ vs. GPT-2$_{\text{SURP}^{1/2}}$	**12**	**0.0007**	**75**	**0.0005**	**58**	**0.0006**
	GPT-2$_{\text{SURP}^{1}}$ vs. GPT-2$_{\text{SURP}^{1/2}}$	**21**	**0.0007**	**131**	**0.0005**	**82**	**0.0006**
	GPT-2$_{\text{SURP}^{4/3}}$ vs. GPT-2$_{\text{SURP}^{1/2}}$	**29**	**0.0013**	**180**	**0.0005**	74	0.0560
	GPT-$_{\text{SURP}^{2}}$ vs. GPT-2$_{\text{SURP}^{1/2}}$	**36**	**0.0462**	**189**	**0.0009**	-56	1.0000
	GPT-2$_{\text{SURP}^{1}}$ vs. GPT-2$_{\text{SURP}^{3/4}}$	**9**	**0.0013**	**57**	**0.0005**	**25**	**0.0423**
	GPT-2$_{\text{SURP}^{4/3}}$ vs. GPT-2$_{\text{SURP}^{3/4}}$	**17**	**0.0046**	**105**	**0.0005**	16	1.0000
	GPT-$_{\text{SURP}^{2}}$ vs. GPT-2$_{\text{SURP}^{3/4}}$	24	0.3308	**114**	**0.0237**	**-114**	**0.0461**
	GPT-2$_{\text{SURP}^{4/3}}$ vs. GPT-2$_{\text{SURP}^{1}}$	8	0.0592	**48**	**0.0005**	-9	1.0000
	GPT-$_{\text{SURP}^{2}}$ vs. GPT-2$_{\text{SURP}^{1}}$	15	1.0000	58	0.3543	**-139**	**0.0012**
	GPT-$_{\text{SURP}^{2}}$ vs. GPT-2$_{\text{SURP}^{4/3}}$	7	1.0000	9	1.0000	**-130**	**0.0006**
GPT-J	GPT-J$_{f(\text{SURP})}$ vs. $\emptyset$	**142**	**0.0007**	**521**	**0.0005**	**588**	**0.0006**
	GPT-J$_{f(\text{SURP})}$ vs. GPT-J$_{\text{PROB}}$	**120**	**0.0007**	**397**	**0.0005**	**323**	**0.0006**
	GPT-J$_{f(\text{SURP})}$ vs. GPT-J$_{\text{SURP}^{1/2}}$	**54**	**0.0013**	**206**	**0.0005**	**66**	**0.0006**
	GPT-J$_{f(\text{SURP})}$ vs. GPT-J$_{\text{SURP}^{3/4}}$	**31**	**0.0198**	**158**	**0.0005**	23	0.0594
	GPT-J$_{f(\text{SURP})}$ vs. GPT-J$_{\text{SURP}^{1}}$	13	0.2696	**122**	**0.0005**	7	0.1658
	GPT-J$_{f(\text{SURP})}$ vs. GPT-J$_{\text{SURP}^{4/3}}$	-3	—	**90**	**0.0005**	14	0.1365
	GPT-J$_{f(\text{SURP})}$ vs. GPT-J$_{\text{SURP}^{2}}$	-8	—	**65**	**0.0019**	**84**	**0.0006**
	GPT-J$_{\text{PROB}}$ vs. $\emptyset$	**22**	**0.0086**	**124**	**0.0005**	**266**	**0.0006**
	GPT-J$_{\text{SURP}^{1/2}}$ vs. $\emptyset$	**89**	**0.0007**	**315**	**0.0005**	**523**	**0.0006**
	GPT-J$_{\text{SURP}^{3/4}}$ vs. $\emptyset$	**112**	**0.0007**	**363**	**0.0005**	**565**	**0.0006**
	GPT-J$_{\text{SURP}^{1}}$ vs. $\emptyset$	**130**	**0.0007**	**399**	**0.0005**	**581**	**0.0006**
	GPT-J$_{\text{SURP}^{4/3}}$ vs. $\emptyset$	**145**	**0.0007**	**431**	**0.0005**	**574**	**0.0006**
	GPT-J$_{\text{SURP}^{2}}$ vs. $\emptyset$	**150**	**0.0007**	**456**	**0.0005**	**505**	**0.0006**
	GPT-J$_{\text{SURP}^{1/2}}$ vs. GPT-J$_{\text{PROB}}$	**66**	**0.0007**	**191**	**0.0005**	**257**	**0.0006**
	GPT-J$_{\text{SURP}^{3/4}}$ vs. GPT-J$_{\text{PROB}}$	**89**	**0.0007**	**239**	**0.0005**	**299**	**0.0006**
	GPT-J$_{\text{SURP}^{1}}$ vs. GPT-J$_{\text{PROB}}$	**107**	**0.0007**	**275**	**0.0005**	**316**	**0.0006**
	GPT-J$_{\text{SURP}^{4/3}}$ vs. GPT-J$_{\text{PROB}}$	**123**	**0.0007**	**307**	**0.0005**	**308**	**0.0006**
	GPT-J$_{\text{SURP}^{2}}$ vs. GPT-J$_{\text{PROB}}$	**128**	**0.0007**	**332**	**0.0005**	**239**	**0.0006**
	GPT-J$_{\text{SURP}^{3/4}}$ vs. GPT-J$_{\text{SURP}^{1/2}}$	**23**	**0.0007**	**48**	**0.0005**	**43**	**0.0006**
	GPT-J$_{\text{SURP}^{1}}$ vs. GPT-J$_{\text{SURP}^{1/2}}$	**41**	**0.0007**	**83**	**0.0005**	**59**	**0.0023**
	GPT-J$_{\text{SURP}^{4/3}}$ vs. GPT-J$_{\text{SURP}^{1/2}}$	**56**	**0.0007**	**116**	**0.0005**	52	0.1189
	GPT-J$_{\text{SURP}^{2}}$ vs. GPT-J$_{\text{SURP}^{1/2}}$	62	0.0600	**141**	**0.0027**	-18	1.0000
	GPT-J$_{\text{SURP}^{1}}$ vs. GPT-J$_{\text{SURP}^{3/4}}$	**18**	**0.0013**	**36**	**0.0005**	16	0.1238
	GPT-J$_{\text{SURP}^{4/3}}$ vs. GPT-J$_{\text{SURP}^{3/4}}$	**33**	**0.0104**	**68**	**0.0010**	9	1.0000
	GPT-J$_{\text{SURP}^{2}}$ vs. GPT-J$_{\text{SURP}^{3/4}}$	39	0.3573	**93**	**0.0271**	-60	0.1238
	GPT-J$_{\text{SURP}^{4/3}}$ vs. GPT-J$_{\text{SURP}^{1}}$	15	0.0571	**32**	**0.0048**	-7	1.0000
	GPT-J$_{\text{SURP}^{2}}$ vs. GPT-J$_{\text{SURP}^{1}}$	21	1.0000	57	0.2028	**-76**	**0.0012**
	GPT-J$_{\text{SURP}^{2}}$ vs. GPT-J$_{\text{SURP}^{4/3}}$	5	1.0000	25	0.8627	**-69**	**0.0006**

	Comparison	Brown (SPR)		NatStor (SPR)		NatStor (Maze)	
		ΔLL	p	ΔLL	p	ΔLL	p
GPT-3	GPT-3$_{f(\text{SURP})}$ vs. $\emptyset$	**127**	**0.0009**	**466**	**0.0008**	**380**	**0.0008**
	GPT-3$_{f(\text{SURP})}$ vs. GPT-3$_{\text{PROB}}$	**102**	**0.0009**	**248**	**0.0008**	**195**	**0.0008**
	GPT-3$_{f(\text{SURP})}$ vs. GPT-3$_{\text{SURP}^{1/2}}$	**51**	**0.0009**	**156**	**0.0008**	**44**	**0.0110**
	GPT-3$_{f(\text{SURP})}$ vs. GPT-3$_{\text{SURP}^{3/4}}$	**31**	**0.0086**	**146**	**0.0008**	19	0.1056
	GPT-3$_{f(\text{SURP})}$ vs. GPT-3$_{\text{SURP}^{1}}$	15	0.0686	**136**	**0.0008**	7	0.1120
	GPT-3$_{f(\text{SURP})}$ vs. GPT-3$_{\text{SURP}^{4/3}}$	-1	—	**124**	**0.0008**	10	0.3445
	GPT-3$_{f(\text{SURP})}$ vs. GPT-3$_{\text{SURP}^{2}}$	-14	—	**114**	**0.0008**	**51**	**0.0069**
	GPT-3$_{\text{PROB}}$ vs. $\emptyset$	**25**	**0.0203**	**218**	**0.0008**	**185**	**0.0008**
	GPT-3$_{\text{SURP}^{1/2}}$ vs. $\emptyset$	**77**	**0.0009**	**310**	**0.0008**	**336**	**0.0008**
	GPT-3$_{\text{SURP}^{3/4}}$ vs. $\emptyset$	**96**	**0.0009**	**321**	**0.0008**	**361**	**0.0008**
	GPT-3$_{\text{SURP}^{1}}$ vs. $\emptyset$	**113**	**0.0009**	**331**	**0.0008**	**373**	**0.0008**
	GPT-3$_{\text{SURP}^{4/3}}$ vs. $\emptyset$	**129**	**0.0009**	**342**	**0.0008**	**370**	**0.0008**
	GPT-3$_{\text{SURP}^{2}}$ vs. $\emptyset$	**142**	**0.0009**	**352**	**0.0008**	**330**	**0.0008**
	GPT-3$_{\text{SURP}^{1/2}}$ vs. GPT-3$_{\text{PROB}}$	**51**	**0.0009**	**92**	**0.0008**	**151**	**0.0008**
	GPT-3$_{\text{SURP}^{3/4}}$ vs. GPT-3$_{\text{PROB}}$	**71**	**0.0009**	**103**	**0.0015**	**177**	**0.0008**
	GPT-3$_{\text{SURP}^{1}}$ vs. GPT-3$_{\text{PROB}}$	**87**	**0.0009**	**113**	**0.0055**	**188**	**0.0008**
	GPT-3$_{\text{SURP}^{4/3}}$ vs. GPT-3$_{\text{PROB}}$	**103**	**0.0009**	**124**	**0.0110**	**186**	**0.0008**
	GPT-3$_{\text{SURP}^{2}}$ vs. GPT-3$_{\text{PROB}}$	**116**	**0.0024**	**134**	**0.0299**	**145**	**0.0008**
	GPT-3$_{\text{SURP}^{3/4}}$ vs. GPT-3$_{\text{SURP}^{1/2}}$	**19**	**0.0017**	11	1.0000	**26**	**0.0024**
	GPT-3$_{\text{SURP}^{1}}$ vs. GPT-3$_{\text{SURP}^{1/2}}$	**36**	**0.0029**	21	0.9633	**37**	**0.0202**
	GPT-3$_{\text{SURP}^{4/3}}$ vs. GPT-3$_{\text{SURP}^{1/2}}$	**52**	**0.0065**	32	0.9633	35	0.2983
	GPT-3$_{\text{SURP}^{2}}$ vs. GPT-3$_{\text{SURP}^{1/2}}$	65	0.0856	42	1.0000	-6	1.0000
	GPT-3$_{\text{SURP}^{1}}$ vs. GPT-3$_{\text{SURP}^{3/4}}$	**16**	**0.0048**	10	0.9633	11	0.2709
	GPT-3$_{\text{SURP}^{4/3}}$ vs. GPT-3$_{\text{SURP}^{3/4}}$	**32**	**0.0275**	22	0.9633	9	1.0000
	GPT-3$_{\text{SURP}^{2}}$ vs. GPT-3$_{\text{SURP}^{3/4}}$	45	0.3074	32	1.0000	-32	0.6052
	GPT-3$_{\text{SURP}^{4/3}}$ vs. GPT-3$_{\text{SURP}^{1}}$	16	0.0755	12	0.9633	-2	1.0000
	GPT-3$_{\text{SURP}^{2}}$ vs. GPT-3$_{\text{SURP}^{1}}$	29	0.7388	22	1.0000	-43	0.0613
	GPT-3$_{\text{SURP}^{2}}$ vs. GPT-3$_{\text{SURP}^{4/3}}$	13	1.0000	10	1.0000	**-41**	**0.0037**
All LMs	All-LMs$_{f(\text{SURP})}$ vs. $\emptyset$	**75**	**0.0010**	**466**	**0.0006**	**380**	**0.0008**
	All-LMs$_{f(\text{SURP})}$ vs. All-LMs$_{\text{PROB}}$	**72**	**0.0010**	**342**	**0.0006**	**195**	**0.0008**
	All-LMs$_{f(\text{SURP})}$ vs. All-LMs$_{\text{SURP}^{1/2}}$	**29**	**0.0195**	**156**	**0.0006**	44	0.1924
	All-LMs$_{f(\text{SURP})}$ vs. All-LMs$_{\text{SURP}^{3/4}}$	18	0.4746	**146**	**0.0006**	19	1.0000
	All-LMs$_{f(\text{SURP})}$ vs. All-LMs$_{\text{SURP}^{1}}$	9	1.0000	**136**	**0.0006**	7	1.0000
	All-LMs$_{f(\text{SURP})}$ vs. All-LMs$_{\text{SURP}^{4/3}}$	0	1.0000	**124**	**0.0006**	10	1.0000
	All-LMs$_{f(\text{SURP})}$ vs. All-LMs$_{\text{SURP}^{2}}$	-6	—	**114**	**0.0006**	51	0.2932
	All-LMs$_{\text{PROB}}$ vs. $\emptyset$	3	1.0000	**124**	**0.0006**	**185**	**0.0008**
	All-LMs$_{\text{SURP}^{1/2}}$ vs. $\emptyset$	**46**	**0.0010**	**310**	**0.0006**	**336**	**0.0008**
	All-LMs$_{\text{SURP}^{3/4}}$ vs. $\emptyset$	**58**	**0.0010**	**321**	**0.0006**	**361**	**0.0008**
	All-LMs$_{\text{SURP}^{1}}$ vs. $\emptyset$	**67**	**0.0010**	**331**	**0.0006**	**373**	**0.0008**
	All-LMs$_{\text{SURP}^{4/3}}$ vs. $\emptyset$	**75**	**0.0010**	**342**	**0.0006**	**370**	**0.0008**
	All-LMs$_{\text{SURP}^{2}}$ vs. $\emptyset$	**82**	**0.0018**	**352**	**0.0006**	**330**	**0.0008**
	All-LMs$_{\text{SURP}^{1/2}}$ vs. All-LMs$_{\text{PROB}}$	**43**	**0.0010**	**186**	**0.0006**	**151**	**0.0008**
	All-LMs$_{\text{SURP}^{3/4}}$ vs. All-LMs$_{\text{PROB}}$	**54**	**0.0010**	**196**	**0.0006**	**177**	**0.0008**
	All-LMs$_{\text{SURP}^{1}}$ vs. All-LMs$_{\text{PROB}}$	**63**	**0.0010**	**206**	**0.0006**	**188**	**0.0008**
	All-LMs$_{\text{SURP}^{4/3}}$ vs. All-LMs$_{\text{PROB}}$	**72**	**0.0010**	**218**	**0.0006**	**186**	**0.0008**
	All-LMs$_{\text{SURP}^{2}}$ vs. All-LMs$_{\text{PROB}}$	**79**	**0.0010**	**228**	**0.0006**	**145**	**0.0008**
	All-LMs$_{\text{SURP}^{3/4}}$ vs. All-LMs$_{\text{SURP}^{1/2}}$	12	0.7499	11	1.0000	26	0.8702
	All-LMs$_{\text{SURP}^{1}}$ vs. All-LMs$_{\text{SURP}^{1/2}}$	21	0.1416	21	1.0000	37	0.3917
	All-LMs$_{\text{SURP}^{4/3}}$ vs. All-LMs$_{\text{SURP}^{1/2}}$	**29**	**0.0196**	32	0.7735	35	0.5675
	All-LMs$_{\text{SURP}^{2}}$ vs. All-LMs$_{\text{SURP}^{1/2}}$	**36**	**0.0301**	42	0.6643	-6	1.0000
	All-LMs$_{\text{SURP}^{1}}$ vs. All-LMs$_{\text{SURP}^{3/4}}$	9	1.0000	10	1.0000	11	1.0000
	All-LMs$_{\text{SURP}^{4/3}}$ vs. All-LMs$_{\text{SURP}^{3/4}}$	17	0.5486	22	1.0000	9	1.0000
	All-LMs$_{\text{SURP}^{2}}$ vs. All-LMs$_{\text{SURP}^{3/4}}$	24	0.3693	32	1.0000	-32	0.9634
	All-LMs$_{\text{SURP}^{4/3}}$ vs. All-LMs$_{\text{SURP}^{1}}$	8	1.0000	12	1.0000	-2	1.0000
	All-LMs$_{\text{SURP}^{2}}$ vs. All-LMs$_{\text{SURP}^{1}}$	15	1.0000	22	1.0000	-43	0.5278
	All-LMs$_{\text{SURP}^{2}}$ vs. All-LMs$_{\text{SURP}^{4/3}}$	7	1.0000	10	1.0000	-41	0.6476

Table S11. Testing Results on Self-Paced (Reading and Maze) Datasets Using Generalized Additive Models (GAMs). Results of key statistical comparisons based on permutation tests of the difference in average test set likelihood between the alternative and null GAM model in each comparison (△LL). Subscripts indicate the assumed functional form of predictability effects: $f(\text{SURP})$ (nonlinear in surprisal), PROB (linear in probability), and SURP^{a} (linear in surprisal raised to exponent a, such that e.g., SURP^{1} is linear in surprisal). Boldface indicates statistical significance. In directional tests where one hypothesis subsumes the other (e.g., $f(\text{SURP})$ vs. SURP^{1}, since a nonlinear surprisal effect subsumes a linear one), dashes (—) indicate failure of the alternative hypothesis (left) to improve over the null hypothesis (right). Otherwise, cells are color coded using cyan to indicate that the hypothesis on the left outperforms the one on the right, and using magenta to indicate that the hypothesis on the right outperforms the one on the left. All p-values are corrected for false discovery rate (15) within each family of tests (delimited by single horizontal lines). Key comparisons are highlighted.

	Comparison	Scan Path ΔLL	Scan Path p	First Pass ΔLL	First Pass p	Go Past ΔLL	Go Past p
n-gram	n-gram$_{f(\text{SURP})}$ vs. $\emptyset$	**181**	**0.0007**	**177**	**0.0007**	52	0.3055
	n-gram$_{f(\text{SURP})}$ vs. n-gram$_{\text{PROB}}$	**160**	**0.0007**	**157**	**0.0007**	**349**	**0.0010**
	n-gram$_{f(\text{SURP})}$ vs. n-gram$_{\text{SURP}^{1/2}}$	34	0.0740	35	0.0555	-13	—
	n-gram$_{f(\text{SURP})}$ vs. n-gram$_{\text{SURP}^{3/4}}$	19	0.4050	16	0.5937	-8	—
	n-gram$_{f(\text{SURP})}$ vs. n-gram$_{\text{SURP}^{1}}$	17	0.4622	8	1.0000	5	1.0000
	n-gram$_{f(\text{SURP})}$ vs. n-gram$_{\text{SURP}^{4/3}}$	30	0.0539	15	0.5238	**26**	**0.0039**
	n-gram$_{f(\text{SURP})}$ vs. n-gram$_{\text{SURP}^{2}}$	**84**	**0.0007**	**64**	**0.0007**	**63**	**0.0010**
	n-gram$_{\text{PROB}}$ vs. $\emptyset$	**21**	**0.0007**	**20**	**0.0007**	-297	—
	n-gram$_{\text{SURP}^{1/2}}$ vs. $\emptyset$	**147**	**0.0007**	**142**	**0.0007**	**65**	**0.0025**
	n-gram$_{\text{SURP}^{3/4}}$ vs. $\emptyset$	**161**	**0.0007**	**161**	**0.0007**	**61**	**0.0183**
	n-gram$_{\text{SURP}^{1}}$ vs. $\emptyset$	**164**	**0.0007**	**169**	**0.0007**	48	0.3870
	n-gram$_{\text{SURP}^{4/3}}$ vs. $\emptyset$	**151**	**0.0007**	**162**	**0.0007**	26	1.0000
	n-gram$_{\text{SURP}^{2}}$ vs. $\emptyset$	**97**	**0.0007**	**113**	**0.0007**	-10	—
	n-gram$_{\text{SURP}^{1/2}}$ vs. n-gram$_{\text{PROB}}$	**126**	**0.0007**	**123**	**0.0007**	**362**	**0.0010**
	n-gram$_{\text{SURP}^{3/4}}$ vs. n-gram$_{\text{PROB}}$	**141**	**0.0007**	**142**	**0.0007**	**357**	**0.0010**
	n-gram$_{\text{SURP}^{1}}$ vs. n-gram$_{\text{PROB}}$	**143**	**0.0007**	**149**	**0.0007**	**344**	**0.0010**
	n-gram$_{\text{SURP}^{4/3}}$ vs. n-gram$_{\text{PROB}}$	**130**	**0.0007**	**142**	**0.0007**	**323**	**0.0010**
	n-gram$_{\text{SURP}^{2}}$ vs. n-gram$_{\text{PROB}}$	**76**	**0.0039**	**94**	**0.0013**	286	0.0744
	n-gram$_{\text{SURP}^{3/4}}$ vs. n-gram$_{\text{SURP}^{1/2}}$	**15**	**0.0043**	**19**	**0.0007**	-4	1.0000
	n-gram$_{\text{SURP}^{1}}$ vs. n-gram$_{\text{SURP}^{1/2}}$	17	0.1343	**27**	**0.0069**	-17	0.9091
	n-gram$_{\text{SURP}^{4/3}}$ vs. n-gram$_{\text{SURP}^{1/2}}$	4	1.0000	20	0.4307	-39	0.0687
	n-gram$_{\text{SURP}^{2}}$ vs. n-gram$_{\text{SURP}^{1/2}}$	**-50**	**0.0087**	-29	0.3104	**-76**	**0.0010**
	n-gram$_{\text{SURP}^{1}}$ vs. n-gram$_{\text{SURP}^{3/4}}$	2	1.0000	8	0.2655	-13	0.0922
	n-gram$_{\text{SURP}^{4/3}}$ vs. n-gram$_{\text{SURP}^{3/4}}$	-11	0.7286	1	1.0000	**-34**	**0.0018**
	n-gram$_{\text{SURP}^{2}}$ vs. n-gram$_{\text{SURP}^{3/4}}$	**-65**	**0.0007**	**-48**	**0.0018**	**-71**	**0.0010**
	n-gram$_{\text{SURP}^{4/3}}$ vs. n-gram$_{\text{SURP}^{1}}$	**-13**	**0.0132**	-7	0.5238	**-22**	**0.0010**
	n-gram$_{\text{SURP}^{2}}$ vs. n-gram$_{\text{SURP}^{1}}$	**-67**	**0.0007**	**-56**	**0.0007**	**-58**	**0.0010**
	n-gram$_{\text{SURP}^{2}}$ vs. n-gram$_{\text{SURP}^{4/3}}$	**-54**	**0.0007**	**-49**	**0.0007**	**-37**	**0.0010**
PCFG	PCFG$_{f(\text{SURP})}$ vs. $\emptyset$	**87**	**0.0016**	**71**	**0.0016**	5	1.0000
	PCFG$_{f(\text{SURP})}$ vs. PCFG$_{\text{PROB}}$	**85**	**0.0016**	**67**	**0.0016**	4	1.0000
	PCFG$_{f(\text{SURP})}$ vs. PCFG$_{\text{SURP}^{1/2}}$	**57**	**0.0016**	**36**	**0.0027**	11	1.0000
	PCFG$_{f(\text{SURP})}$ vs. PCFG$_{\text{SURP}^{3/4}}$	**59**	**0.0016**	**39**	**0.0016**	7	1.0000
	PCFG$_{f(\text{SURP})}$ vs. PCFG$_{\text{SURP}^{1}}$	**63**	**0.0016**	**42**	**0.0016**	5	1.0000
	PCFG$_{f(\text{SURP})}$ vs. PCFG$_{\text{SURP}^{4/3}}$	**67**	**0.0016**	**46**	**0.0016**	4	1.0000
	PCFG$_{f(\text{SURP})}$ vs. PCFG$_{\text{SURP}^{2}}$	**75**	**0.0016**	**56**	**0.0016**	3	1.0000
	PCFG$_{\text{PROB}}$ vs. $\emptyset$	2	1.0000	4	0.1339	2	1.0000
	PCFG$_{\text{SURP}^{1/2}}$ vs. $\emptyset$	**30**	**0.0069**	**35**	**0.0016**	-5	—
	PCFG$_{\text{SURP}^{3/4}}$ vs. $\emptyset$	**28**	**0.0286**	**33**	**0.0061**	-1	—
	PCFG$_{\text{SURP}^{1}}$ vs. $\emptyset$	24	0.0763	**30**	**0.0090**	1	1.0000
	PCFG$_{\text{SURP}^{4/3}}$ vs. $\emptyset$	19	0.2379	**25**	**0.0287**	1	1.0000
	PCFG$_{\text{SURP}^{2}}$ vs. $\emptyset$	11	0.8800	15	0.2929	2	1.0000
	PCFG$_{\text{SURP}^{1/2}}$ vs. PCFG$_{\text{PROB}}$	**28**	**0.0098**	**31**	**0.0066**	-7	1.0000
	PCFG$_{\text{SURP}^{3/4}}$ vs. PCFG$_{\text{PROB}}$	**26**	**0.0499**	**29**	**0.0165**	-3	1.0000
	PCFG$_{\text{SURP}^{1}}$ vs. PCFG$_{\text{PROB}}$	23	0.0983	**26**	**0.0252**	-1	1.0000
	PCFG$_{\text{SURP}^{4/3}}$ vs. PCFG$_{\text{PROB}}$	18	0.3279	21	0.1120	-1	1.0000
	PCFG$_{\text{SURP}^{2}}$ vs. PCFG$_{\text{PROB}}$	10	1.0000	11	0.6774	0	1.0000
	PCFG$_{\text{SURP}^{3/4}}$ vs. PCFG$_{\text{SURP}^{1/2}}$	-2	0.6421	-2	0.6060	4	1.0000
	PCFG$_{\text{SURP}^{1}}$ vs. PCFG$_{\text{SURP}^{1/2}}$	-6	0.3349	-5	0.2969	6	1.0000
	PCFG$_{\text{SURP}^{4/3}}$ vs. PCFG$_{\text{SURP}^{1/2}}$	-11	0.2034	-10	0.1339	6	1.0000
	PCFG$_{\text{SURP}^{2}}$ vs. PCFG$_{\text{SURP}^{1/2}}$	-19	0.0605	**-20**	**0.0213**	7	1.0000
	PCFG$_{\text{SURP}^{1}}$ vs. PCFG$_{\text{SURP}^{3/4}}$	-3	0.2141	-3	0.1663	2	1.0000
	PCFG$_{\text{SURP}^{4/3}}$ vs. PCFG$_{\text{SURP}^{3/4}}$	-8	0.1186	-8	0.1099	2	1.0000
	PCFG$_{\text{SURP}^{2}}$ vs. PCFG$_{\text{SURP}^{3/4}}$	**-16**	**0.0499**	**-18**	**0.0213**	3	1.0000
	PCFG$_{\text{SURP}^{4/3}}$ vs. PCFG$_{\text{SURP}^{1}}$	-5	0.0821	-5	0.0700	1	1.0000
	PCFG$_{\text{SURP}^{2}}$ vs. PCFG$_{\text{SURP}^{1}}$	**-13**	**0.0499**	**-15**	**0.0203**	1	1.0000
	PCFG$_{\text{SURP}^{2}}$ vs. PCFG$_{\text{SURP}^{4/3}}$	-8	0.0638	**-10**	**0.0213**	1	1.0000
GPT-2	GPT-2$_{f(\text{SURP})}$ vs. $\emptyset$	**340**	**0.0006**	**286**	**0.0006**	**104**	**0.0068**
	GPT-2$_{f(\text{SURP})}$ vs. GPT-2$_{\text{PROB}}$	**287**	**0.0006**	**240**	**0.0006**	**393**	**0.0012**
	GPT-2$_{f(\text{SURP})}$ vs. GPT-2$_{\text{SURP}^{1/2}}$	**100**	**0.0006**	**73**	**0.0058**	7	1.0000
	GPT-2$_{f(\text{SURP})}$ vs. GPT-2$_{\text{SURP}^{3/4}}$	**62**	**0.0075**	42	0.0994	-3	—
	GPT-2$_{f(\text{SURP})}$ vs. GPT-2$_{\text{SURP}^{1}}$	**41**	**0.0325**	27	0.3145	-1	—
	GPT-2$_{f(\text{SURP})}$ vs. GPT-2$_{\text{SURP}^{4/3}}$	**40**	**0.0220**	33	0.1077	15	0.2186
	GPT-2$_{f(\text{SURP})}$ vs. GPT-$_{\text{SURP}^{2}}$	**117**	**0.0006**	**113**	**0.0006**	**68**	**0.0012**
	GPT-2$_{\text{PROB}}$ vs. $\emptyset$	**53**	**0.0006**	**46**	**0.0006**	-288	—
	GPT-2$_{\text{SURP}^{1/2}}$ vs. $\emptyset$	**240**	**0.0006**	**213**	**0.0006**	**97**	**0.0012**
	GPT-2$_{\text{SURP}^{3/4}}$ vs. $\emptyset$	**278**	**0.0006**	**244**	**0.0006**	**108**	**0.0012**
	GPT-2$_{\text{SURP}^{1}}$ vs. $\emptyset$	**299**	**0.0006**	**258**	**0.0006**	**105**	**0.0030**
	GPT-2$_{\text{SURP}^{4/3}}$ vs. $\emptyset$	**299**	**0.0006**	**253**	**0.0006**	89	0.0535
	GPT-$_{\text{SURP}^{2}}$ vs. $\emptyset$	**223**	**0.0006**	**173**	**0.0006**	36	1.0000
	GPT-2$_{\text{SURP}^{1/2}}$ vs. GPT-2$_{\text{PROB}}$	**187**	**0.0006**	**167**	**0.0006**	**385**	**0.0012**
	GPT-2$_{\text{SURP}^{3/4}}$ vs. GPT-2$_{\text{PROB}}$	**224**	**0.0006**	**198**	**0.0006**	**396**	**0.0012**
	GPT-2$_{\text{SURP}^{1}}$ vs. GPT-2$_{\text{PROB}}$	**245**	**0.0006**	**213**	**0.0006**	**394**	**0.0012**
	GPT-2$_{\text{SURP}^{4/3}}$ vs. GPT-2$_{\text{PROB}}$	**246**	**0.0006**	**207**	**0.0006**	**377**	**0.0012**
	GPT-$_{\text{SURP}^{2}}$ vs. GPT-2$_{\text{PROB}}$	**170**	**0.0006**	**127**	**0.0012**	**325**	**0.0037**
	GPT-2$_{\text{SURP}^{3/4}}$ vs. GPT-2$_{\text{SURP}^{1/2}}$	**38**	**0.0006**	**31**	**0.0006**	10	0.3918
	GPT-2$_{\text{SURP}^{1}}$ vs. GPT-2$_{\text{SURP}^{1/2}}$	**59**	**0.0006**	**45**	**0.0006**	8	1.0000
	GPT-2$_{\text{SURP}^{4/3}}$ vs. GPT-2$_{\text{SURP}^{1/2}}$	**60**	**0.0068**	40	0.0950	-8	1.0000
	GPT-$_{\text{SURP}^{2}}$ vs. GPT-2$_{\text{SURP}^{1/2}}$	-17	1.0000	-40	0.5591	-61	0.1766
	GPT-2$_{\text{SURP}^{1}}$ vs. GPT-2$_{\text{SURP}^{3/4}}$	**21**	**0.0022**	**15**	**0.0366**	-2	1.0000
	GPT-2$_{\text{SURP}^{4/3}}$ vs. GPT-2$_{\text{SURP}^{3/4}}$	22	0.4102	9	1.0000	-19	1.0000
	GPT-$_{\text{SURP}^{2}}$ vs. GPT-2$_{\text{SURP}^{3/4}}$	-55	0.0796	**-71**	**0.0093**	**-71**	**0.0071**
	GPT-2$_{\text{SURP}^{4/3}}$ vs. GPT-2$_{\text{SURP}^{1}}$	1	1.0000	-6	1.0000	-17	0.2468
	GPT-$_{\text{SURP}^{2}}$ vs. GPT-2$_{\text{SURP}^{1}}$	**-76**	**0.0012**	**-85**	**0.0006**	**-69**	**0.0022**
	GPT-$_{\text{SURP}^{2}}$ vs. GPT-2$_{\text{SURP}^{4/3}}$	**-77**	**0.0006**	**-79**	**0.0006**	**-52**	**0.0012**
GPT-J	GPT-J$_{f(\text{SURP})}$ vs. $\emptyset$	**195**	**0.0008**	**185**	**0.0008**	**72**	**0.0126**
	GPT-J$_{f(\text{SURP})}$ vs. GPT-J$_{\text{PROB}}$	**135**	**0.0008**	**131**	**0.0008**	43	0.2529
	GPT-J$_{f(\text{SURP})}$ vs. GPT-J$_{\text{SURP}^{1/2}}$	47	0.0625	**50**	**0.0348**	9	1.0000
	GPT-J$_{f(\text{SURP})}$ vs. GPT-J$_{\text{SURP}^{3/4}}$	30	0.2628	34	0.1369	-1	—
	GPT-J$_{f(\text{SURP})}$ vs. GPT-J$_{\text{SURP}^{1}}$	19	0.5183	25	0.2459	-6	—
	GPT-J$_{f(\text{SURP})}$ vs. GPT-J$_{\text{SURP}^{4/3}}$	14	0.5526	17	0.4059	-5	—
	GPT-J$_{f(\text{SURP})}$ vs. GPT-J$_{\text{SURP}^{2}}$	**24**	**0.0213**	**29**	**0.0137**	11	0.3243
	GPT-J$_{\text{PROB}}$ vs. $\emptyset$	**61**	**0.0008**	**54**	**0.0008**	**29**	**0.0044**
	GPT-J$_{\text{SURP}^{1/2}}$ vs. $\emptyset$	**148**	**0.0008**	**135**	**0.0008**	**62**	**0.0044**
	GPT-J$_{\text{SURP}^{3/4}}$ vs. $\emptyset$	**166**	**0.0008**	**151**	**0.0008**	**73**	**0.0044**
	GPT-J$_{\text{SURP}^{1}}$ vs. $\emptyset$	**176**	**0.0008**	**160**	**0.0008**	**77**	**0.0044**
	GPT-J$_{\text{SURP}^{4/3}}$ vs. $\emptyset$	**182**	**0.0008**	**168**	**0.0008**	**77**	**0.0044**
	GPT-J$_{\text{SURP}^{2}}$ vs. $\emptyset$	**172**	**0.0008**	**156**	**0.0008**	61	0.0504
	GPT-J$_{\text{SURP}^{1/2}}$ vs. GPT-J$_{\text{PROB}}$	**88**	**0.0008**	**80**	**0.0008**	**33**	**0.0231**
	GPT-J$_{\text{SURP}^{3/4}}$ vs. GPT-J$_{\text{PROB}}$	**105**	**0.0008**	**97**	**0.0008**	**44**	**0.0151**
	GPT-J$_{\text{SURP}^{1}}$ vs. GPT-J$_{\text{PROB}}$	**115**	**0.0008**	**106**	**0.0008**	**49**	**0.0159**
	GPT-J$_{\text{SURP}^{4/3}}$ vs. GPT-J$_{\text{PROB}}$	**121**	**0.0008**	**114**	**0.0008**	48	0.0575
	GPT-J$_{\text{SURP}^{2}}$ vs. GPT-J$_{\text{PROB}}$	**111**	**0.0008**	**102**	**0.0008**	32	0.7367
	GPT-J$_{\text{SURP}^{3/4}}$ vs. GPT-J$_{\text{SURP}^{1/2}}$	**17**	**0.0008**	**16**	**0.0008**	**10**	**0.0126**
	GPT-J$_{\text{SURP}^{1}}$ vs. GPT-J$_{\text{SURP}^{1/2}}$	**27**	**0.0029**	**26**	**0.0051**	15	0.0504
	GPT-J$_{\text{SURP}^{4/3}}$ vs. GPT-J$_{\text{SURP}^{1/2}}$	**33**	**0.0317**	**33**	**0.0246**	15	0.5069
	GPT-J$_{\text{SURP}^{2}}$ vs. GPT-J$_{\text{SURP}^{1/2}}$	23	0.9544	22	1.0000	-2	1.0000
	GPT-J$_{\text{SURP}^{1}}$ vs. GPT-J$_{\text{SURP}^{3/4}}$	**10**	**0.0476**	**10**	**0.0370**	5	0.5069
	GPT-J$_{\text{SURP}^{4/3}}$ vs. GPT-J$_{\text{SURP}^{3/4}}$	16	0.2953	17	0.1545	4	1.0000
	GPT-J$_{\text{SURP}^{2}}$ vs. GPT-J$_{\text{SURP}^{3/4}}$	6	1.0000	6	1.0000	-12	1.0000
	GPT-J$_{\text{SURP}^{4/3}}$ vs. GPT-J$_{\text{SURP}^{1}}$	6	0.9544	7	0.4613	-1	1.0000
	GPT-J$_{\text{SURP}^{2}}$ vs. GPT-J$_{\text{SURP}^{1}}$	-4	1.0000	-4	1.0000	-17	0.5069
	GPT-J$_{\text{SURP}^{2}}$ vs. GPT-J$_{\text{SURP}^{4/3}}$	-10	1.0000	-12	0.7767	-16	0.1366
GPT-3	GPT-3$_{f(\text{SURP})}$ vs. $\emptyset$	**116**	**0.0012**	**117**	**0.0010**	**53**	**0.0055**
	GPT-3$_{f(\text{SURP})}$ vs. GPT-3$_{\text{PROB}}$	**65**	**0.0156**	**68**	**0.0046**	**38**	**0.0165**
	GPT-3$_{f(\text{SURP})}$ vs. GPT-3$_{\text{SURP}^{1/2}}$	18	1.0000	20	0.8652	14	0.4135
	GPT-3$_{f(\text{SURP})}$ vs. GPT-3$_{\text{SURP}^{3/4}}$	11	1.0000	11	1.0000	4	1.0000
	GPT-3$_{f(\text{SURP})}$ vs. GPT-3$_{\text{SURP}^{1}}$	8	1.0000	7	1.0000	-3	—
	GPT-3$_{f(\text{SURP})}$ vs. GPT-3$_{\text{SURP}^{4/3}}$	8	1.0000	5	1.0000	-7	—
	GPT-3$_{f(\text{SURP})}$ vs. GPT-3$_{\text{SURP}^{2}}$	**19**	**0.0313**	13	0.4948	-5	—
	GPT-3$_{\text{PROB}}$ vs. $\emptyset$	**52**	**0.0012**	**49**	**0.0010**	15	0.1574
	GPT-3$_{\text{SURP}^{1/2}}$ vs. $\emptyset$	**98**	**0.0012**	**97**	**0.0010**	**39**	**0.0066**
	GPT-3$_{\text{SURP}^{3/4}}$ vs. $\emptyset$	**106**	**0.0012**	**106**	**0.0010**	**49**	**0.0055**
	GPT-3$_{\text{SURP}^{1}}$ vs. $\emptyset$	**109**	**0.0012**	**110**	**0.0010**	**56**	**0.0055**
	GPT-3$_{\text{SURP}^{4/3}}$ vs. $\emptyset$	**108**	**0.0012**	**112**	**0.0010**	**60**	**0.0055**
	GPT-3$_{\text{SURP}^{2}}$ vs. $\emptyset$	**98**	**0.0012**	**104**	**0.0010**	**58**	**0.0110**
	GPT-3$_{\text{SURP}^{1/2}}$ vs. GPT-3$_{\text{PROB}}$	**46**	**0.0012**	**48**	**0.0010**	**24**	**0.0165**
	GPT-3$_{\text{SURP}^{3/4}}$ vs. GPT-3$_{\text{PROB}}$	**54**	**0.0022**	**57**	**0.0010**	**35**	**0.0110**
	GPT-3$_{\text{SURP}^{1}}$ vs. GPT-3$_{\text{PROB}}$	**57**	**0.0012**	**61**	**0.0010**	**41**	**0.0110**
	GPT-3$_{\text{SURP}^{4/3}}$ vs. GPT-3$_{\text{PROB}}$	**57**	**0.0040**	**63**	**0.0010**	**45**	**0.0165**
	GPT-3$_{\text{SURP}^{2}}$ vs. GPT-3$_{\text{PROB}}$	46	0.1576	**55**	**0.0353**	43	0.0584
	GPT-3$_{\text{SURP}^{3/4}}$ vs. GPT-3$_{\text{SURP}^{1/2}}$	7	0.1374	**9**	**0.0237**	**11**	**0.0165**
	GPT-3$_{\text{SURP}^{1}}$ vs. GPT-3$_{\text{SURP}^{1/2}}$	11	0.5236	14	0.1136	17	0.0636
	GPT-3$_{\text{SURP}^{4/3}}$ vs. GPT-3$_{\text{SURP}^{1/2}}$	10	1.0000	15	0.5045	21	0.1554
	GPT-3$_{\text{SURP}^{2}}$ vs. GPT-3$_{\text{SURP}^{1/2}}$	0	1.0000	7	1.0000	19	0.6383
	GPT-3$_{\text{SURP}^{1}}$ vs. GPT-3$_{\text{SURP}^{3/4}}$	3	1.0000	5	0.5375	6	0.3098
	GPT-3$_{\text{SURP}^{4/3}}$ vs. GPT-3$_{\text{SURP}^{3/4}}$	3	1.0000	6	1.0000	11	0.5411
	GPT-3$_{\text{SURP}^{2}}$ vs. GPT-3$_{\text{SURP}^{3/4}}$	-8	1.0000	-2	1.0000	8	1.0000
	GPT-3$_{\text{SURP}^{4/3}}$ vs. GPT-3$_{\text{SURP}^{1}}$	0	1.0000	1	1.0000	4	0.9649
	GPT-3$_{\text{SURP}^{2}}$ vs. GPT-3$_{\text{SURP}^{1}}$	-11	1.0000	-6	1.0000	2	1.0000
	GPT-3$_{\text{SURP}^{2}}$ vs. GPT-3$_{\text{SURP}^{4/3}}$	-10	0.5459	-8	1.0000	-2	1.0000
All LMs	All-LMs$_{f(\text{SURP})}$ vs. $\emptyset$	**181**	**0.0007**	**177**	**0.0007**	53	0.1034
	All-LMs$_{f(\text{SURP})}$ vs. All-LMs$_{\text{PROB}}$	**129**	**0.0007**	**131**	**0.0007**	51	1.0000
	All-LMs$_{f(\text{SURP})}$ vs. All-LMs$_{\text{SURP}^{1/2}}$	**34**	**0.0446**	**42**	**0.0032**	-9	—
	All-LMs$_{f(\text{SURP})}$ vs. All-LMs$_{\text{SURP}^{3/4}}$	19	0.6219	26	0.1476	-8	—
	All-LMs$_{f(\text{SURP})}$ vs. All-LMs$_{\text{SURP}^{1}}$	17	0.9135	16	0.8356	-3	—
	All-LMs$_{f(\text{SURP})}$ vs. All-LMs$_{\text{SURP}^{4/3}}$	30	0.1655	15	1.0000	-7	—
	All-LMs$_{f(\text{SURP})}$ vs. All-LMs$_{\text{SURP}^{2}}$	**83**	**0.0007**	**64**	**0.0007**	17	1.0000
	All-LMs$_{\text{PROB}}$ vs. $\emptyset$	**52**	**0.0007**	**46**	**0.0007**	2	1.0000
	All-LMs$_{\text{SURP}^{1/2}}$ vs. $\emptyset$	**147**	**0.0007**	**135**	**0.0007**	**62**	**0.0110**
	All-LMs$_{\text{SURP}^{3/4}}$ vs. $\emptyset$	**161**	**0.0007**	**151**	**0.0007**	**61**	**0.0110**
	All-LMs$_{\text{SURP}^{1}}$ vs. $\emptyset$	**164**	**0.0007**	**160**	**0.0007**	**56**	**0.0403**
	All-LMs$_{\text{SURP}^{4/3}}$ vs. $\emptyset$	**151**	**0.0007**	**162**	**0.0007**	**60**	**0.0467**
	All-LMs$_{\text{SURP}^{2}}$ vs. $\emptyset$	**98**	**0.0007**	**113**	**0.0007**	36	1.0000
	All-LMs$_{\text{SURP}^{1/2}}$ vs. All-LMs$_{\text{PROB}}$	**95**	**0.0007**	**89**	**0.0007**	61	1.0000
	All-LMs$_{\text{SURP}^{3/4}}$ vs. All-LMs$_{\text{PROB}}$	**110**	**0.0007**	**105**	**0.0007**	59	1.0000
	All-LMs$_{\text{SURP}^{1}}$ vs. All-LMs$_{\text{PROB}}$	**112**	**0.0007**	**115**	**0.0007**	54	1.0000
	All-LMs$_{\text{SURP}^{4/3}}$ vs. All-LMs$_{\text{PROB}}$	**99**	**0.0007**	**116**	**0.0007**	58	1.0000
	All-LMs$_{\text{SURP}^{2}}$ vs. All-LMs$_{\text{PROB}}$	**46**	**0.0012**	**67**	**0.0007**	35	1.0000
	All-LMs$_{\text{SURP}^{3/4}}$ vs. All-LMs$_{\text{SURP}^{1/2}}$	15	0.4733	16	0.2754	-2	1.0000
	All-LMs$_{\text{SURP}^{1}}$ vs. All-LMs$_{\text{SURP}^{1/2}}$	17	0.4408	**26**	**0.0272**	-6	1.0000
	All-LMs$_{\text{SURP}^{4/3}}$ vs. All-LMs$_{\text{SURP}^{1/2}}$	4	1.0000	27	0.0506	-2	1.0000
	All-LMs$_{\text{SURP}^{2}}$ vs. All-LMs$_{\text{SURP}^{1/2}}$	**-49**	**0.0012**	-21	0.3236	-26	0.8136
	All-LMs$_{\text{SURP}^{1}}$ vs. All-LMs$_{\text{SURP}^{3/4}}$	2	1.0000	10	1.0000	-5	1.0000
	All-LMs$_{\text{SURP}^{4/3}}$ vs. All-LMs$_{\text{SURP}^{3/4}}$	-11	1.0000	11	1.0000	-1	1.0000
	All-LMs$_{\text{SURP}^{2}}$ vs. All-LMs$_{\text{SURP}^{3/4}}$	**-64**	**0.0007**	**-37**	**0.0073**	-24	0.8922
	All-LMs$_{\text{SURP}^{4/3}}$ vs. All-LMs$_{\text{SURP}^{1}}$	-13	1.0000	2	1.0000	4	1.0000
	All-LMs$_{\text{SURP}^{2}}$ vs. All-LMs$_{\text{SURP}^{1}}$	**-66**	**0.0007**	**-47**	**0.0007**	-19	1.0000
	All-LMs$_{\text{SURP}^{2}}$ vs. All-LMs$_{\text{SURP}^{4/3}}$	**-53**	**0.0012**	**-49**	**0.0032**	-24	1.0000

Table S12. Testing Results on Dundee (Eye-Tracking) using generalized additive models (GAMs). Results of key statistical comparisons based on permutation tests of the difference in average test set likelihood between the alternative and null GAM model in each comparison (△LL). Subscripts indicate the assumed functional form of predictability effects: $f(\text{SURP})$ (nonlinear in surprisal), PROB (linear in probability), and SURP^a (linear in surprisal raised to exponent a, such that e.g., SURP^1 is linear in surprisal). Boldface indicates statistical significance. In directional tests where one hypothesis subsumes the other (e.g., $f(\text{SURP})$ vs. SURP^1, since a nonlinear surprisal effect subsumes a linear one), dashes (—) indicate failure of the alternative hypothesis (left) to improve over the null hypothesis (right). Otherwise, cells are color coded using cyan to indicate that the hypothesis on the left outperforms the one on the right, and using magenta to indicate that the hypothesis on the right outperforms the one on the left. All p-values are corrected for false discovery rate (15) within each family of tests (delimited by single horizontal lines). Key comparisons are highlighted.

	Comparison	First Pass ΔLL	First Pass p	Go Past ΔLL	Go Past p
n-gram	n-gram$_{f(\text{SURP})}$ vs. $\emptyset$	**117**	**0.0014**	**82**	**0.0012**
	n-gram$_{f(\text{SURP})}$ vs. n-gram$_{\text{PROB}}$	**105**	**0.0014**	**66**	**0.0012**
	n-gram$_{f(\text{SURP})}$ vs. n-gram$_{\text{SURP}^{1/2}}$	**62**	**0.0014**	12	0.5045
	n-gram$_{f(\text{SURP})}$ vs. n-gram$_{\text{SURP}^{3/4}}$	**49**	**0.0029**	7	0.7874
	n-gram$_{f(\text{SURP})}$ vs. n-gram$_{\text{SURP}^{1}}$	**39**	**0.0260**	5	1.0000
	n-gram$_{f(\text{SURP})}$ vs. n-gram$_{\text{SURP}^{4/3}}$	31	0.0865	8	0.7083
	n-gram$_{f(\text{SURP})}$ vs. n-gram$_{\text{SURP}^{2}}$	26	0.1356	**25**	**0.0203**
	n-gram$_{\text{PROB}}$ vs. $\emptyset$	**12**	**0.0313**	**16**	**0.0236**
	n-gram$_{\text{SURP}^{1/2}}$ vs. $\emptyset$	**55**	**0.0014**	**70**	**0.0012**
	n-gram$_{\text{SURP}^{3/4}}$ vs. $\emptyset$	**68**	**0.0014**	**75**	**0.0012**
	n-gram$_{\text{SURP}^{1}}$ vs. $\emptyset$	**78**	**0.0014**	**77**	**0.0012**
	n-gram$_{\text{SURP}^{4/3}}$ vs. $\emptyset$	**87**	**0.0018**	**74**	**0.0012**
	n-gram$_{\text{SURP}^{2}}$ vs. $\emptyset$	**92**	**0.0014**	**57**	**0.0020**
	n-gram$_{\text{SURP}^{1/2}}$ vs. n-gram$_{\text{PROB}}$	**43**	**0.0029**	**54**	**0.0012**
	n-gram$_{\text{SURP}^{3/4}}$ vs. n-gram$_{\text{PROB}}$	**56**	**0.0018**	**59**	**0.0012**
	n-gram$_{\text{SURP}^{1}}$ vs. n-gram$_{\text{PROB}}$	**66**	**0.0018**	**61**	**0.0012**
	n-gram$_{\text{SURP}^{4/3}}$ vs. n-gram$_{\text{PROB}}$	**75**	**0.0014**	**58**	**0.0020**
	n-gram$_{\text{SURP}^{2}}$ vs. n-gram$_{\text{PROB}}$	**80**	**0.0018**	41	0.0572
	n-gram$_{\text{SURP}^{3/4}}$ vs. n-gram$_{\text{SURP}^{1/2}}$	**13**	**0.0025**	5	0.7295
	n-gram$_{\text{SURP}^{1}}$ vs. n-gram$_{\text{SURP}^{1/2}}$	**23**	**0.0082**	7	1.0000
	n-gram$_{\text{SURP}^{4/3}}$ vs. n-gram$_{\text{SURP}^{1/2}}$	**31**	**0.0122**	4	1.0000
	n-gram$_{\text{SURP}^{2}}$ vs. n-gram$_{\text{SURP}^{1/2}}$	36	0.1109	-13	1.0000
	n-gram$_{\text{SURP}^{1}}$ vs. n-gram$_{\text{SURP}^{3/4}}$	**10**	**0.0122**	2	1.0000
	n-gram$_{\text{SURP}^{4/3}}$ vs. n-gram$_{\text{SURP}^{3/4}}$	18	0.0534	-1	1.0000
	n-gram$_{\text{SURP}^{2}}$ vs. n-gram$_{\text{SURP}^{3/4}}$	23	0.3912	-18	0.3945
	n-gram$_{\text{SURP}^{4/3}}$ vs. n-gram$_{\text{SURP}^{1}}$	8	0.1786	-3	1.0000
	n-gram$_{\text{SURP}^{2}}$ vs. n-gram$_{\text{SURP}^{1}}$	13	0.9700	-20	0.0667
	n-gram$_{\text{SURP}^{2}}$ vs. n-gram$_{\text{SURP}^{4/3}}$	5	1.0000	**-17**	**0.0027**
PCFG	PCFG$_{f(\text{SURP})}$ vs. $\emptyset$	**177**	**0.0007**	**48**	**0.0128**
	PCFG$_{f(\text{SURP})}$ vs. PCFG$_{\text{PROB}}$	**177**	**0.0007**	**47**	**0.0128**
	PCFG$_{f(\text{SURP})}$ vs. PCFG$_{\text{SURP}^{1/2}}$	**111**	**0.0007**	9	0.7972
	PCFG$_{f(\text{SURP})}$ vs. PCFG$_{\text{SURP}^{3/4}}$	**101**	**0.0007**	9	0.4484
	PCFG$_{f(\text{SURP})}$ vs. PCFG$_{\text{SURP}^{1}}$	**98**	**0.0007**	8	0.3324
	PCFG$_{f(\text{SURP})}$ vs. PCFG$_{\text{SURP}^{4/3}}$	**102**	**0.0007**	10	0.2250
	PCFG$_{f(\text{SURP})}$ vs. PCFG$_{\text{SURP}^{2}}$	**123**	**0.0007**	**19**	**0.0293**
	PCFG$_{\text{PROB}}$ vs. $\emptyset$	1	1.0000	1	1.0000
	PCFG$_{\text{SURP}^{1/2}}$ vs. $\emptyset$	**67**	**0.0007**	**38**	**0.0128**
	PCFG$_{\text{SURP}^{3/4}}$ vs. $\emptyset$	**77**	**0.0007**	**39**	**0.0128**
	PCFG$_{\text{SURP}^{1}}$ vs. $\emptyset$	**80**	**0.0007**	**39**	**0.0220**
	PCFG$_{\text{SURP}^{4/3}}$ vs. $\emptyset$	**75**	**0.0007**	**38**	**0.0297**
	PCFG$_{\text{SURP}^{2}}$ vs. $\emptyset$	**55**	**0.0226**	29	0.1920
	PCFG$_{\text{SURP}^{1/2}}$ vs. PCFG$_{\text{PROB}}$	**66**	**0.0007**	**37**	**0.0128**
	PCFG$_{\text{SURP}^{3/4}}$ vs. PCFG$_{\text{PROB}}$	**76**	**0.0007**	**38**	**0.0128**
	PCFG$_{\text{SURP}^{1}}$ vs. PCFG$_{\text{PROB}}$	**79**	**0.0007**	**38**	**0.0204**
	PCFG$_{\text{SURP}^{4/3}}$ vs. PCFG$_{\text{PROB}}$	**74**	**0.0007**	**37**	**0.0330**
	PCFG$_{\text{SURP}^{2}}$ vs. PCFG$_{\text{PROB}}$	**54**	**0.0324**	27	0.2250
	PCFG$_{\text{SURP}^{3/4}}$ vs. PCFG$_{\text{SURP}^{1/2}}$	**10**	**0.0226**	1	1.0000
	PCFG$_{\text{SURP}^{1}}$ vs. PCFG$_{\text{SURP}^{1/2}}$	13	0.2408	1	1.0000
	PCFG$_{\text{SURP}^{4/3}}$ vs. PCFG$_{\text{SURP}^{1/2}}$	8	1.0000	-1	1.0000
	PCFG$_{\text{SURP}^{2}}$ vs. PCFG$_{\text{SURP}^{1/2}}$	-12	1.0000	-10	1.0000
	PCFG$_{\text{SURP}^{1}}$ vs. PCFG$_{\text{SURP}^{3/4}}$	3	1.0000	0	1.0000
	PCFG$_{\text{SURP}^{4/3}}$ vs. PCFG$_{\text{SURP}^{3/4}}$	-2	1.0000	-1	1.0000
	PCFG$_{\text{SURP}^{2}}$ vs. PCFG$_{\text{SURP}^{3/4}}$	-22	0.6717	-11	0.6956
	PCFG$_{\text{SURP}^{4/3}}$ vs. PCFG$_{\text{SURP}^{1}}$	-5	1.0000	-2	1.0000
	PCFG$_{\text{SURP}^{2}}$ vs. PCFG$_{\text{SURP}^{1}}$	-25	0.2408	-11	0.3292
	PCFG$_{\text{SURP}^{2}}$ vs. PCFG$_{\text{SURP}^{4/3}}$	-20	0.1499	-9	0.1164

	Comparison	First Pass ΔLL	First Pass p	Go Past ΔLL	Go Past p
GPT-2	GPT-2$_{f(\text{SURP})}$ vs. $\emptyset$	**314**	**0.0006**	**151**	**0.0008**
	GPT-2$_{f(\text{SURP})}$ vs. GPT-2$_{\text{PROB}}$	**247**	**0.0006**	**108**	**0.0008**
	GPT-2$_{f(\text{SURP})}$ vs. GPT-2$_{\text{SURP}^{1/2}}$	**85**	**0.0006**	20	0.0782
	GPT-2$_{f(\text{SURP})}$ vs. GPT-2$_{\text{SURP}^{3/4}}$	**52**	**0.0023**	9	0.2785
	GPT-2$_{f(\text{SURP})}$ vs. GPT-2$_{\text{SURP}^{1}}$	29	0.1408	4	0.7935
	GPT-2$_{f(\text{SURP})}$ vs. GPT-2$_{\text{SURP}^{4/3}}$	13	1.0000	7	0.6457
	GPT-2$_{f(\text{SURP})}$ vs. GPT-$_{\text{SURP}^{2}}$	21	0.9179	**36**	**0.0022**
	GPT-2$_{\text{PROB}}$ vs. $\emptyset$	**67**	**0.0006**	**43**	**0.0008**
	GPT-2$_{\text{SURP}^{1/2}}$ vs. $\emptyset$	**229**	**0.0006**	**131**	**0.0008**
	GPT-2$_{\text{SURP}^{3/4}}$ vs. $\emptyset$	**261**	**0.0006**	**142**	**0.0008**
	GPT-2$_{\text{SURP}^{1}}$ vs. $\emptyset$	**285**	**0.0006**	**147**	**0.0008**
	GPT-2$_{\text{SURP}^{4/3}}$ vs. $\emptyset$	**301**	**0.0006**	**144**	**0.0008**
	GPT-$_{\text{SURP}^{2}}$ vs. $\emptyset$	**293**	**0.0006**	**115**	**0.0008**
	GPT-2$_{\text{SURP}^{1/2}}$ vs. GPT-2$_{\text{PROB}}$	**162**	**0.0006**	**88**	**0.0008**
	GPT-2$_{\text{SURP}^{3/4}}$ vs. GPT-2$_{\text{PROB}}$	**194**	**0.0006**	**99**	**0.0008**
	GPT-2$_{\text{SURP}^{1}}$ vs. GPT-2$_{\text{PROB}}$	**218**	**0.0006**	**104**	**0.0008**
	GPT-2$_{\text{SURP}^{4/3}}$ vs. GPT-2$_{\text{PROB}}$	**234**	**0.0006**	**101**	**0.0008**
	GPT-$_{\text{SURP}^{2}}$ vs. GPT-2$_{\text{PROB}}$	**226**	**0.0006**	**72**	**0.0022**
	GPT-2$_{\text{SURP}^{3/4}}$ vs. GPT-2$_{\text{SURP}^{1/2}}$	**33**	**0.0006**	11	0.0530
	GPT-2$_{\text{SURP}^{1}}$ vs. GPT-2$_{\text{SURP}^{1/2}}$	**56**	**0.0006**	15	0.1886
	GPT-2$_{\text{SURP}^{4/3}}$ vs. GPT-2$_{\text{SURP}^{1/2}}$	**72**	**0.0012**	13	0.9270
	GPT-$_{\text{SURP}^{2}}$ vs. GPT-2$_{\text{SURP}^{1/2}}$	64	0.1489	-16	0.9270
	GPT-2$_{\text{SURP}^{1}}$ vs. GPT-2$_{\text{SURP}^{3/4}}$	**23**	**0.0006**	5	0.7118
	GPT-2$_{\text{SURP}^{4/3}}$ vs. GPT-2$_{\text{SURP}^{3/4}}$	**39**	**0.0187**	2	1.0000
	GPT-$_{\text{SURP}^{2}}$ vs. GPT-2$_{\text{SURP}^{3/4}}$	31	0.9179	-27	0.0799
	GPT-2$_{\text{SURP}^{4/3}}$ vs. GPT-2$_{\text{SURP}^{1}}$	16	0.1501	-3	1.0000
	GPT-$_{\text{SURP}^{2}}$ vs. GPT-2$_{\text{SURP}^{1}}$	8	1.0000	**-32**	**0.0027**
	GPT-$_{\text{SURP}^{2}}$ vs. GPT-2$_{\text{SURP}^{4/3}}$	-8	1.0000	**-29**	**0.0008**
GPT-J	GPT-J$_{f(\text{SURP})}$ vs. $\emptyset$	**199**	**0.0008**	**105**	**0.0009**
	GPT-J$_{f(\text{SURP})}$ vs. GPT-J$_{\text{PROB}}$	**111**	**0.0008**	**77**	**0.0009**
	GPT-J$_{f(\text{SURP})}$ vs. GPT-J$_{\text{SURP}^{1/2}}$	19	0.1782	**22**	**0.0103**
	GPT-J$_{f(\text{SURP})}$ vs. GPT-J$_{\text{SURP}^{3/4}}$	6	0.8056	10	0.1204
	GPT-J$_{f(\text{SURP})}$ vs. GPT-J$_{\text{SURP}^{1}}$	3	1.0000	3	0.6038
	GPT-J$_{f(\text{SURP})}$ vs. GPT-J$_{\text{SURP}^{4/3}}$	10	0.8056	2	1.0000
	GPT-J$_{f(\text{SURP})}$ vs. GPT-J$_{\text{SURP}^{2}}$	**48**	**0.0139**	**23**	**0.0183**
	GPT-J$_{\text{PROB}}$ vs. $\emptyset$	**88**	**0.0008**	**29**	**0.0059**
	GPT-J$_{\text{SURP}^{1/2}}$ vs. $\emptyset$	**180**	**0.0008**	**84**	**0.0009**
	GPT-J$_{\text{SURP}^{3/4}}$ vs. $\emptyset$	**193**	**0.0008**	**96**	**0.0009**
	GPT-J$_{\text{SURP}^{1}}$ vs. $\emptyset$	**196**	**0.0008**	**103**	**0.0009**
	GPT-J$_{\text{SURP}^{4/3}}$ vs. $\emptyset$	**190**	**0.0008**	**103**	**0.0009**
	GPT-J$_{\text{SURP}^{2}}$ vs. $\emptyset$	**152**	**0.0008**	**82**	**0.0017**
	GPT-J$_{\text{SURP}^{1/2}}$ vs. GPT-J$_{\text{PROB}}$	**92**	**0.0008**	**55**	**0.0009**
	GPT-J$_{\text{SURP}^{3/4}}$ vs. GPT-J$_{\text{PROB}}$	**105**	**0.0008**	**67**	**0.0009**
	GPT-J$_{\text{SURP}^{1}}$ vs. GPT-J$_{\text{PROB}}$	**108**	**0.0008**	**74**	**0.0009**
	GPT-J$_{\text{SURP}^{4/3}}$ vs. GPT-J$_{\text{PROB}}$	**102**	**0.0008**	**74**	**0.0009**
	GPT-J$_{\text{SURP}^{2}}$ vs. GPT-J$_{\text{PROB}}$	64	0.1782	**53**	**0.0149**
	GPT-J$_{\text{SURP}^{3/4}}$ vs. GPT-J$_{\text{SURP}^{1/2}}$	13	0.0612	**12**	**0.0009**
	GPT-J$_{\text{SURP}^{1}}$ vs. GPT-J$_{\text{SURP}^{1/2}}$	16	0.4700	**19**	**0.0039**
	GPT-J$_{\text{SURP}^{4/3}}$ vs. GPT-J$_{\text{SURP}^{1/2}}$	10	1.0000	19	0.1209
	GPT-J$_{\text{SURP}^{2}}$ vs. GPT-J$_{\text{SURP}^{1/2}}$	-28	0.8844	-1	1.0000
	GPT-J$_{\text{SURP}^{1}}$ vs. GPT-J$_{\text{SURP}^{3/4}}$	3	1.0000	7	0.0862
	GPT-J$_{\text{SURP}^{4/3}}$ vs. GPT-J$_{\text{SURP}^{3/4}}$	-3	1.0000	7	0.9988
	GPT-J$_{\text{SURP}^{2}}$ vs. GPT-J$_{\text{SURP}^{3/4}}$	-41	0.1429	-14	0.8617
	GPT-J$_{\text{SURP}^{4/3}}$ vs. GPT-J$_{\text{SURP}^{1}}$	-7	1.0000	0	1.0000
	GPT-J$_{\text{SURP}^{2}}$ vs. GPT-J$_{\text{SURP}^{1}}$	**-45**	**0.0139**	-20	0.0874
	GPT-J$_{\text{SURP}^{2}}$ vs. GPT-J$_{\text{SURP}^{4/3}}$	**-38**	**0.0008**	**-21**	**0.0009**

	Comparison	First Pass ΔLL	First Pass p	Go Past ΔLL	Go Past p
GPT-3	GPT-3$_{f(\text{SURP})}$ vs. $\emptyset$	**159**	**0.0012**	**65**	**0.0022**
	GPT-3$_{f(\text{SURP})}$ vs. GPT-3$_{\text{PROB}}$	**61**	**0.0220**	**53**	**0.0022**
	GPT-3$_{f(\text{SURP})}$ vs. GPT-3$_{\text{SURP}^{1/2}}$	19	0.5553	**28**	**0.0022**
	GPT-3$_{f(\text{SURP})}$ vs. GPT-3$_{\text{SURP}^{3/4}}$	16	0.5179	**19**	**0.0065**
	GPT-3$_{f(\text{SURP})}$ vs. GPT-3$_{\text{SURP}^{1}}$	19	0.0836	**11**	**0.0272**
	GPT-3$_{f(\text{SURP})}$ vs. GPT-3$_{\text{SURP}^{4/3}}$	**27**	**0.0022**	4	0.5920
	GPT-3$_{f(\text{SURP})}$ vs. GPT-3$_{\text{SURP}^{2}}$	**53**	**0.0012**	1	1.0000
	GPT-3$_{\text{PROB}}$ vs. $\emptyset$	**98**	**0.0012**	12	0.5397
	GPT-3$_{\text{SURP}^{1/2}}$ vs. $\emptyset$	**139**	**0.0012**	**37**	**0.0139**
	GPT-3$_{\text{SURP}^{3/4}}$ vs. $\emptyset$	**143**	**0.0012**	**47**	**0.0027**
	GPT-3$_{\text{SURP}^{1}}$ vs. $\emptyset$	**140**	**0.0012**	**55**	**0.0022**
	GPT-3$_{\text{SURP}^{4/3}}$ vs. $\emptyset$	**132**	**0.0012**	**62**	**0.0022**
	GPT-3$_{\text{SURP}^{2}}$ vs. $\emptyset$	**106**	**0.0012**	**64**	**0.0022**
	GPT-3$_{\text{SURP}^{1/2}}$ vs. GPT-3$_{\text{PROB}}$	**41**	**0.0012**	**25**	**0.0022**
	GPT-3$_{\text{SURP}^{3/4}}$ vs. GPT-3$_{\text{PROB}}$	**45**	**0.0220**	**35**	**0.0022**
	GPT-3$_{\text{SURP}^{1}}$ vs. GPT-3$_{\text{PROB}}$	42	0.1031	**43**	**0.0022**
	GPT-3$_{\text{SURP}^{4/3}}$ vs. GPT-3$_{\text{PROB}}$	34	0.5553	**50**	**0.0022**
	GPT-3$_{\text{SURP}^{2}}$ vs. GPT-3$_{\text{PROB}}$	8	1.0000	**52**	**0.0022**
	GPT-3$_{\text{SURP}^{3/4}}$ vs. GPT-3$_{\text{SURP}^{1/2}}$	3	1.0000	**10**	**0.0022**
	GPT-3$_{\text{SURP}^{1}}$ vs. GPT-3$_{\text{SURP}^{1/2}}$	1	1.0000	**18**	**0.0022**
	GPT-3$_{\text{SURP}^{4/3}}$ vs. GPT-3$_{\text{SURP}^{1/2}}$	-8	1.0000	**25**	**0.0022**
	GPT-3$_{\text{SURP}^{2}}$ vs. GPT-3$_{\text{SURP}^{1/2}}$	-33	0.5139	**27**	**0.0315**
	GPT-3$_{\text{SURP}^{1}}$ vs. GPT-3$_{\text{SURP}^{3/4}}$	-3	1.0000	**8**	**0.0022**
	GPT-3$_{\text{SURP}^{4/3}}$ vs. GPT-3$_{\text{SURP}^{3/4}}$	-11	0.9154	**15**	**0.0067**
	GPT-3$_{\text{SURP}^{2}}$ vs. GPT-3$_{\text{SURP}^{3/4}}$	-37	0.1630	17	0.1950
	GPT-3$_{\text{SURP}^{4/3}}$ vs. GPT-3$_{\text{SURP}^{1}}$	-9	0.4642	**7**	**0.0258**
	GPT-3$_{\text{SURP}^{2}}$ vs. GPT-3$_{\text{SURP}^{1}}$	-34	0.0778	9	0.6195
	GPT-3$_{\text{SURP}^{2}}$ vs. GPT-3$_{\text{SURP}^{4/3}}$	**-25**	**0.0296**	2	1.0000
All LMs	All-LMs$_{f(\text{SURP})}$ vs. $\emptyset$	**177**	**0.0009**	**82**	**0.0009**
	All-LMs$_{f(\text{SURP})}$ vs. All-LMs$_{\text{PROB}}$	**111**	**0.0009**	**66**	**0.0009**
	All-LMs$_{f(\text{SURP})}$ vs. All-LMs$_{\text{SURP}^{1/2}}$	38	0.0612	12	1.0000
	All-LMs$_{f(\text{SURP})}$ vs. All-LMs$_{\text{SURP}^{3/4}}$	35	0.1186	7	1.0000
	All-LMs$_{f(\text{SURP})}$ vs. All-LMs$_{\text{SURP}^{1}}$	37	0.0812	5	1.0000
	All-LMs$_{f(\text{SURP})}$ vs. All-LMs$_{\text{SURP}^{4/3}}$	**46**	**0.0275**	8	1.0000
	All-LMs$_{f(\text{SURP})}$ vs. All-LMs$_{\text{SURP}^{2}}$	**71**	**0.0009**	18	0.4249
	All-LMs$_{\text{PROB}}$ vs. $\emptyset$	**67**	**0.0009**	16	0.0761
	All-LMs$_{\text{SURP}^{1/2}}$ vs. $\emptyset$	**139**	**0.0009**	**70**	**0.0009**
	All-LMs$_{\text{SURP}^{3/4}}$ vs. $\emptyset$	**143**	**0.0009**	**75**	**0.0009**
	All-LMs$_{\text{SURP}^{1}}$ vs. $\emptyset$	**140**	**0.0009**	**77**	**0.0009**
	All-LMs$_{\text{SURP}^{4/3}}$ vs. $\emptyset$	**132**	**0.0009**	**74**	**0.0009**
	All-LMs$_{\text{SURP}^{2}}$ vs. $\emptyset$	**106**	**0.0017**	**64**	**0.0009**
	All-LMs$_{\text{SURP}^{1/2}}$ vs. All-LMs$_{\text{PROB}}$	**73**	**0.0009**	**54**	**0.0009**
	All-LMs$_{\text{SURP}^{3/4}}$ vs. All-LMs$_{\text{PROB}}$	**76**	**0.0009**	**59**	**0.0009**
	All-LMs$_{\text{SURP}^{1}}$ vs. All-LMs$_{\text{PROB}}$	**73**	**0.0009**	**61**	**0.0009**
	All-LMs$_{\text{SURP}^{4/3}}$ vs. All-LMs$_{\text{PROB}}$	**65**	**0.0009**	**58**	**0.0009**
	All-LMs$_{\text{SURP}^{2}}$ vs. All-LMs$_{\text{PROB}}$	**39**	**0.0476**	**48**	**0.0009**
	All-LMs$_{\text{SURP}^{3/4}}$ vs. All-LMs$_{\text{SURP}^{1/2}}$	3	1.0000	5	1.0000
	All-LMs$_{\text{SURP}^{1}}$ vs. All-LMs$_{\text{SURP}^{1/2}}$	1	1.0000	7	1.0000
	All-LMs$_{\text{SURP}^{4/3}}$ vs. All-LMs$_{\text{SURP}^{1/2}}$	-8	1.0000	4	1.0000
	All-LMs$_{\text{SURP}^{2}}$ vs. All-LMs$_{\text{SURP}^{1/2}}$	-33	0.1351	-6	1.0000
	All-LMs$_{\text{SURP}^{1}}$ vs. All-LMs$_{\text{SURP}^{3/4}}$	-3	1.0000	2	1.0000
	All-LMs$_{\text{SURP}^{4/3}}$ vs. All-LMs$_{\text{SURP}^{3/4}}$	-11	1.0000	-1	1.0000
	All-LMs$_{\text{SURP}^{2}}$ vs. All-LMs$_{\text{SURP}^{3/4}}$	-37	0.0812	-11	1.0000
	All-LMs$_{\text{SURP}^{4/3}}$ vs. All-LMs$_{\text{SURP}^{1}}$	-9	1.0000	-3	1.0000
	All-LMs$_{\text{SURP}^{2}}$ vs. All-LMs$_{\text{SURP}^{1}}$	-34	0.1351	-13	1.0000
	All-LMs$_{\text{SURP}^{2}}$ vs. All-LMs$_{\text{SURP}^{4/3}}$	-25	0.4968	-10	1.0000

Table S13. Testing Results on GECO (Eye-Tracking) using generalized additive models (GAMs). Results of key statistical comparisons based on permutation tests of the difference in average test set likelihood between the alternative and null GAM model in each comparison (△LL). Subscripts indicate the assumed functional form of predictability effects: $f(\text{SURP})$ (nonlinear in surprisal), PROB (linear in probability), and SURP^a (linear in surprisal raised to exponent a, such that e.g., SURP^1 is linear in surprisal). Boldface indicates statistical significance. In directional tests where one hypothesis subsumes the other (e.g., $f(\text{SURP})$ vs. SURP^1, since a nonlinear surprisal effect subsumes a linear one), dashes (—) indicate failure of the alternative hypothesis (left) to improve over the null hypothesis (right). Otherwise, cells are color coded using cyan to indicate that the hypothesis on the left outperforms the one on the right, and using magenta to indicate that the hypothesis on the right outperforms the one on the left. All p-values are corrected for false discovery rate (15) within each family of tests (delimited by single horizontal lines). Key comparisons are highlighted.

	Comparison	Scan Path		First Pass		Go Past	
		ΔLL	p	ΔLL	p	ΔLL	p
Cloze	Cloze$_{f(\text{SURP})}$ vs. ∅	25	0.2309	19	0.4545	-2	—
	Cloze$_{f(\text{SURP})}$ vs. Cloze$_{\text{PROB}}$	14	0.2529	7	1.0000	0	1.0000
	Cloze$_{f(\text{SURP})}$ vs. Cloze$_{\text{SURP}^{1/2}}$	9	0.3290	0	1.0000	1	1.0000
	Cloze$_{f(\text{SURP})}$ vs. Cloze$_{\text{SURP}^{3/4}}$	9	0.2529	0	1.0000	3	1.0000
	Cloze$_{f(\text{SURP})}$ vs. Cloze$_{\text{SURP}^{1}}$	8	0.2529	1	1.0000	3	1.0000
	Cloze$_{f(\text{SURP})}$ vs. Cloze$_{\text{SURP}^{4/3}}$	8	0.2529	1	1.0000	3	1.0000
	Cloze$_{f(\text{SURP})}$ vs. Cloze$_{\text{SURP}^{2}}$	7	0.2529	1	1.0000	2	1.0000
	Cloze$_{\text{PROB}}$ vs. ∅	11	0.3793	12	0.6803	-3	—
	Cloze$_{\text{SURP}^{1/2}}$ vs. ∅	16	0.2529	20	0.4535	-4	—
	Cloze$_{\text{SURP}^{3/4}}$ vs. ∅	16	0.2529	20	0.4535	-5	—
	Cloze$_{\text{SURP}^{1}}$ vs. ∅	16	0.3793	19	0.6803	-6	—
	Cloze$_{\text{SURP}^{4/3}}$ vs. ∅	17	0.2529	18	0.4545	-5	—
	Cloze$_{\text{SURP}^{2}}$ vs. ∅	18	0.2529	18	0.6047	-4	—
	Cloze$_{\text{SURP}^{1/2}}$ vs. Cloze$_{\text{PROB}}$	5	0.6032	8	0.9923	-1	1.0000
	Cloze$_{\text{SURP}^{3/4}}$ vs. Cloze$_{\text{PROB}}$	5	0.9661	8	1.0000	-2	1.0000
	Cloze$_{\text{SURP}^{1}}$ vs. Cloze$_{\text{PROB}}$	5	1.0000	6	1.0000	-3	1.0000
	Cloze$_{\text{SURP}^{4/3}}$ vs. Cloze$_{\text{PROB}}$	6	1.0000	6	1.0000	-3	1.0000
	Cloze$_{\text{SURP}^{2}}$ vs. Cloze$_{\text{PROB}}$	7	1.0000	6	1.0000	-1	1.0000
	Cloze$_{\text{SURP}^{3/4}}$ vs. Cloze$_{\text{SURP}^{1/2}}$	0	1.0000	0	1.0000	-2	1.0000
	Cloze$_{\text{SURP}^{1}}$ vs. Cloze$_{\text{SURP}^{1/2}}$	1	1.0000	-1	1.0000	-2	1.0000
	Cloze$_{\text{SURP}^{4/3}}$ vs. Cloze$_{\text{SURP}^{1/2}}$	1	1.0000	-2	1.0000	-2	1.0000
	Cloze$_{\text{SURP}^{2}}$ vs. Cloze$_{\text{SURP}^{1/2}}$	2	1.0000	-2	1.0000	-1	1.0000
	Cloze$_{\text{SURP}^{1}}$ vs. Cloze$_{\text{SURP}^{3/4}}$	0	1.0000	-1	1.0000	0	1.0000
	Cloze$_{\text{SURP}^{4/3}}$ vs. Cloze$_{\text{SURP}^{3/4}}$	1	1.0000	-2	1.0000	0	1.0000
	Cloze$_{\text{SURP}^{2}}$ vs. Cloze$_{\text{SURP}^{3/4}}$	2	1.0000	-2	1.0000	1	1.0000
	Cloze$_{\text{SURP}^{4/3}}$ vs. Cloze$_{\text{SURP}^{1}}$	0	1.0000	0	1.0000	0	1.0000
	Cloze$_{\text{SURP}^{2}}$ vs. Cloze$_{\text{SURP}^{1}}$	1	1.0000	0	1.0000	1	1.0000
	Cloze$_{\text{SURP}^{2}}$ vs. Cloze$_{\text{SURP}^{4/3}}$	1	1.0000	0	1.0000	1	1.0000
n-gram	*n*-gram$_{f(\text{SURP})}$ vs. ∅	15	0.8153	8	1.0000	24	0.0990
	n-gram$_{f(\text{SURP})}$ vs. *n*-gram$_{\text{PROB}}$	9	1.0000	6	1.0000	24	0.0990
	n-gram$_{f(\text{SURP})}$ vs. *n*-gram$_{\text{SURP}^{1/2}}$	4	1.0000	3	1.0000	9	0.5358
	n-gram$_{f(\text{SURP})}$ vs. *n*-gram$_{\text{SURP}^{3/4}}$	7	0.8153	8	0.8735	10	0.2822
	n-gram$_{f(\text{SURP})}$ vs. *n*-gram$_{\text{SURP}^{1}}$	9	0.7298	10	0.7165	10	0.2822
	n-gram$_{f(\text{SURP})}$ vs. *n*-gram$_{\text{SURP}^{4/3}}$	13	0.7298	13	0.7165	11	0.3028
	n-gram$_{f(\text{SURP})}$ vs. *n*-gram$_{\text{SURP}^{2}}$	21	0.7298	21	0.7165	15	0.3028
	n-gram$_{\text{PROB}}$ vs. ∅	5	0.7298	2	1.0000	0	1.0000
	n-gram$_{\text{SURP}^{1/2}}$ vs. ∅	10	1.0000	5	1.0000	15	0.2822
	n-gram$_{\text{SURP}^{3/4}}$ vs. ∅	8	1.0000	-1	—	14	0.2822
	n-gram$_{\text{SURP}^{1}}$ vs. ∅	5	1.0000	-2	—	14	0.2822
	n-gram$_{\text{SURP}^{4/3}}$ vs. ∅	2	1.0000	-5	—	13	0.3028
	n-gram$_{\text{SURP}^{2}}$ vs. ∅	-7	—	-13	—	9	0.7496
	n-gram$_{\text{SURP}^{1/2}}$ vs. *n*-gram$_{\text{PROB}}$	5	1.0000	2	1.0000	14	0.2822
	n-gram$_{\text{SURP}^{3/4}}$ vs. *n*-gram$_{\text{PROB}}$	2	1.0000	-3	1.0000	14	0.2822
	n-gram$_{\text{SURP}^{1}}$ vs. *n*-gram$_{\text{PROB}}$	0	1.0000	-4	1.0000	14	0.3028
	n-gram$_{\text{SURP}^{4/3}}$ vs. *n*-gram$_{\text{PROB}}$	-4	1.0000	-7	1.0000	13	0.5358
	n-gram$_{\text{SURP}^{2}}$ vs. *n*-gram$_{\text{PROB}}$	-12	1.0000	-16	0.8735	9	1.0000
	n-gram$_{\text{SURP}^{3/4}}$ vs. *n*-gram$_{\text{SURP}^{1/2}}$	-2	1.0000	-5	1.0000	-1	1.0000
	n-gram$_{\text{SURP}^{1}}$ vs. *n*-gram$_{\text{SURP}^{1/2}}$	-5	1.0000	-6	1.0000	-1	1.0000
	n-gram$_{\text{SURP}^{4/3}}$ vs. *n*-gram$_{\text{SURP}^{1/2}}$	-9	0.8153	-9	1.0000	-2	1.0000
	n-gram$_{\text{SURP}^{2}}$ vs. *n*-gram$_{\text{SURP}^{1/2}}$	-17	0.7298	-18	0.7165	-6	1.0000
	n-gram$_{\text{SURP}^{1}}$ vs. *n*-gram$_{\text{SURP}^{3/4}}$	-2	1.0000	-1	1.0000	0	1.0000
	n-gram$_{\text{SURP}^{4/3}}$ vs. *n*-gram$_{\text{SURP}^{3/4}}$	-6	0.8153	-4	1.0000	-1	1.0000
	n-gram$_{\text{SURP}^{2}}$ vs. *n*-gram$_{\text{SURP}^{3/4}}$	-15	0.7298	-13	0.8735	-5	1.0000
	n-gram$_{\text{SURP}^{4/3}}$ vs. *n*-gram$_{\text{SURP}^{1}}$	-4	0.8153	-3	1.0000	-1	1.0000
	n-gram$_{\text{SURP}^{2}}$ vs. *n*-gram$_{\text{SURP}^{1}}$	-12	0.7298	-12	0.7165	-5	1.0000
	n-gram$_{\text{SURP}^{2}}$ vs. *n*-gram$_{\text{SURP}^{4/3}}$	-8	0.7298	-9	0.7165	-4	0.7496
PCFG	PCFG$_{f(\text{SURP})}$ vs. ∅	**28**	**0.0110**	20	0.0638	-2	—
	PCFG$_{f(\text{SURP})}$ vs. PCFG$_{\text{PROB}}$	**27**	**0.0154**	19	0.0586	1	1.0000
	PCFG$_{f(\text{SURP})}$ vs. PCFG$_{\text{SURP}^{1/2}}$	**23**	**0.0267**	16	0.2114	1	1.0000
	PCFG$_{f(\text{SURP})}$ vs. PCFG$_{\text{SURP}^{3/4}}$	**24**	**0.0154**	17	0.0861	5	1.0000
	PCFG$_{f(\text{SURP})}$ vs. PCFG$_{\text{SURP}^{1}}$	**24**	**0.0154**	16	0.1003	3	1.0000
	PCFG$_{f(\text{SURP})}$ vs. PCFG$_{\text{SURP}^{4/3}}$	**23**	**0.0154**	14	0.2114	-1	—
	PCFG$_{f(\text{SURP})}$ vs. PCFG$_{\text{SURP}^{2}}$	18	0.1209	8	1.0000	-2	—
	PCFG$_{\text{PROB}}$ vs. ∅	2	0.6377	1	1.0000	-3	—
	PCFG$_{\text{SURP}^{1/2}}$ vs. ∅	6	0.5270	4	1.0000	-3	—
	PCFG$_{\text{SURP}^{3/4}}$ vs. ∅	4	0.6921	3	1.0000	-7	—
	PCFG$_{\text{SURP}^{1}}$ vs. ∅	4	0.5151	4	0.2694	-5	—
	PCFG$_{\text{SURP}^{4/3}}$ vs. ∅	6	0.1836	6	0.0638	-1	—
	PCFG$_{\text{SURP}^{2}}$ vs. ∅	**10**	**0.0220**	**12**	**0.0275**	0	1.0000
	PCFG$_{\text{SURP}^{1/2}}$ vs. PCFG$_{\text{PROB}}$	4	1.0000	3	1.0000	1	1.0000
	PCFG$_{\text{SURP}^{3/4}}$ vs. PCFG$_{\text{PROB}}$	3	1.0000	2	1.0000	-3	1.0000
	PCFG$_{\text{SURP}^{1}}$ vs. PCFG$_{\text{PROB}}$	3	1.0000	4	0.6315	-1	1.0000
	PCFG$_{\text{SURP}^{4/3}}$ vs. PCFG$_{\text{PROB}}$	4	0.6921	6	0.1197	2	1.0000
	PCFG$_{\text{SURP}^{2}}$ vs. PCFG$_{\text{PROB}}$	9	0.1307	**12**	**0.0275**	3	1.0000
	PCFG$_{\text{SURP}^{3/4}}$ vs. PCFG$_{\text{SURP}^{1/2}}$	-1	1.0000	-1	1.0000	-4	1.0000
	PCFG$_{\text{SURP}^{1}}$ vs. PCFG$_{\text{SURP}^{1/2}}$	-1	1.0000	0	1.0000	-2	1.0000
	PCFG$_{\text{SURP}^{4/3}}$ vs. PCFG$_{\text{SURP}^{1/2}}$	0	1.0000	2	1.0000	1	1.0000
	PCFG$_{\text{SURP}^{2}}$ vs. PCFG$_{\text{SURP}^{1/2}}$	4	1.0000	8	0.6662	2	1.0000
	PCFG$_{\text{SURP}^{1}}$ vs. PCFG$_{\text{SURP}^{3/4}}$	0	1.0000	1	1.0000	2	1.0000
	PCFG$_{\text{SURP}^{4/3}}$ vs. PCFG$_{\text{SURP}^{3/4}}$	1	1.0000	4	1.0000	6	1.0000
	PCFG$_{\text{SURP}^{2}}$ vs. PCFG$_{\text{SURP}^{3/4}}$	6	0.5673	10	0.1220	7	1.0000
	PCFG$_{\text{SURP}^{4/3}}$ vs. PCFG$_{\text{SURP}^{1}}$	1	1.0000	2	0.5388	4	1.0000
	PCFG$_{\text{SURP}^{2}}$ vs. PCFG$_{\text{SURP}^{1}}$	6	0.3349	8	0.0895	5	1.0000
	PCFG$_{\text{SURP}^{2}}$ vs. PCFG$_{\text{SURP}^{4/3}}$	5	0.3867	6	0.1759	1	1.0000

	Comparison	Scan Path		First Pass		Go Past	
		ΔLL	p	ΔLL	p	ΔLL	p
GPT-2	GPT-2$_{f(\text{SURP})}$ vs. ∅	52	0.0838	38	1.0000	14	1.0000
	GPT-2$_{f(\text{SURP})}$ vs. GPT-2$_{\text{PROB}}$	46	0.0838	29	1.0000	13	1.0000
	GPT-2$_{f(\text{SURP})}$ vs. GPT-2$_{\text{SURP}^{1/2}}$	25	0.2470	15	1.0000	6	1.0000
	GPT-2$_{f(\text{SURP})}$ vs. GPT-2$_{\text{SURP}^{3/4}}$	21	0.3440	14	1.0000	4	1.0000
	GPT-2$_{f(\text{SURP})}$ vs. GPT-2$_{\text{SURP}^{1}}$	18	0.3636	14	1.0000	1	1.0000
	GPT-2$_{f(\text{SURP})}$ vs. GPT-2$_{\text{SURP}^{4/3}}$	15	0.4689	14	1.0000	-1	—
	GPT-2$_{f(\text{SURP})}$ vs. GPT-$_{\text{SURP}^{2}}$	8	1.0000	13	1.0000	-4	—
	GPT-2$_{\text{PROB}}$ vs. ∅	6	0.4689	9	0.9620	1	1.0000
	GPT-2$_{\text{SURP}^{1/2}}$ vs. ∅	27	0.0838	23	0.9620	8	1.0000
	GPT-2$_{\text{SURP}^{3/4}}$ vs. ∅	31	0.0838	24	1.0000	10	1.0000
	GPT-2$_{\text{SURP}^{1}}$ vs. ∅	34	0.0838	24	1.0000	13	1.0000
	GPT-2$_{\text{SURP}^{4/3}}$ vs. ∅	37	0.0838	24	1.0000	16	1.0000
	GPT-$_{\text{SURP}^{2}}$ vs. ∅	43	0.0838	25	1.0000	18	1.0000
	GPT-2$_{\text{SURP}^{1/2}}$ vs. GPT-2$_{\text{PROB}}$	21	0.0838	14	1.0000	7	1.0000
	GPT-2$_{\text{SURP}^{3/4}}$ vs. GPT-2$_{\text{PROB}}$	25	0.1099	15	1.0000	9	1.0000
	GPT-2$_{\text{SURP}^{1}}$ vs. GPT-2$_{\text{PROB}}$	28	0.1399	15	1.0000	12	1.0000
	GPT-2$_{\text{SURP}^{4/3}}$ vs. GPT-2$_{\text{PROB}}$	31	0.1750	15	1.0000	14	1.0000
	GPT-$_{\text{SURP}^{2}}$ vs. GPT-2$_{\text{PROB}}$	37	0.1374	16	1.0000	17	1.0000
	GPT-2$_{\text{SURP}^{3/4}}$ vs. GPT-2$_{\text{SURP}^{1/2}}$	4	0.9149	1	1.0000	2	1.0000
	GPT-2$_{\text{SURP}^{1}}$ vs. GPT-2$_{\text{SURP}^{1/2}}$	7	0.9818	1	1.0000	5	1.0000
	GPT-2$_{\text{SURP}^{4/3}}$ vs. GPT-2$_{\text{SURP}^{1/2}}$	10	0.9922	1	1.0000	8	1.0000
	GPT-$_{\text{SURP}^{2}}$ vs. GPT-2$_{\text{SURP}^{1/2}}$	16	0.8863	2	1.0000	10	1.0000
	GPT-2$_{\text{SURP}^{1}}$ vs. GPT-2$_{\text{SURP}^{3/4}}$	3	1.0000	0	1.0000	2	1.0000
	GPT-2$_{\text{SURP}^{4/3}}$ vs. GPT-2$_{\text{SURP}^{3/4}}$	6	1.0000	0	1.0000	5	1.0000
	GPT-$_{\text{SURP}^{2}}$ vs. GPT-2$_{\text{SURP}^{3/4}}$	12	0.9945	1	1.0000	8	1.0000
	GPT-2$_{\text{SURP}^{4/3}}$ vs. GPT-2$_{\text{SURP}^{1}}$	3	1.0000	0	1.0000	3	1.0000
	GPT-$_{\text{SURP}^{2}}$ vs. GPT-2$_{\text{SURP}^{1}}$	9	1.0000	1	1.0000	5	1.0000
	GPT-$_{\text{SURP}^{2}}$ vs. GPT-2$_{\text{SURP}^{4/3}}$	7	1.0000	1	1.0000	2	1.0000
GPT-J	GPT-J$_{f(\text{SURP})}$ vs. ∅	18	1.0000	17	1.0000	2	1.0000
	GPT-J$_{f(\text{SURP})}$ vs. GPT-J$_{\text{PROB}}$	17	1.0000	10	1.0000	9	1.0000
	GPT-J$_{f(\text{SURP})}$ vs. GPT-J$_{\text{SURP}^{1/2}}$	9	1.0000	3	1.0000	4	1.0000
	GPT-J$_{f(\text{SURP})}$ vs. GPT-J$_{\text{SURP}^{3/4}}$	7	1.0000	3	1.0000	3	1.0000
	GPT-J$_{f(\text{SURP})}$ vs. GPT-J$_{\text{SURP}^{1}}$	5	0.2859	4	0.7587	2	1.0000
	GPT-J$_{f(\text{SURP})}$ vs. GPT-J$_{\text{SURP}^{4/3}}$	3	1.0000	5	1.0000	0	1.0000
	GPT-J$_{f(\text{SURP})}$ vs. GPT-J$_{\text{SURP}^{2}}$	0	1.0000	8	1.0000	-3	—
	GPT-J$_{\text{PROB}}$ vs. ∅	1	1.0000	6	1.0000	-7	—
	GPT-J$_{\text{SURP}^{1/2}}$ vs. ∅	9	1.0000	13	1.0000	-2	—
	GPT-J$_{\text{SURP}^{3/4}}$ vs. ∅	11	1.0000	14	1.0000	-1	—
	GPT-J$_{\text{SURP}^{1}}$ vs. ∅	13	1.0000	13	1.0000	0	1.0000
	GPT-J$_{\text{SURP}^{4/3}}$ vs. ∅	15	1.0000	11	1.0000	2	1.0000
	GPT-J$_{\text{SURP}^{2}}$ vs. ∅	18	1.0000	9	1.0000	5	1.0000
	GPT-J$_{\text{SURP}^{1/2}}$ vs. GPT-J$_{\text{PROB}}$	8	1.0000	7	1.0000	5	1.0000
	GPT-J$_{\text{SURP}^{3/4}}$ vs. GPT-J$_{\text{PROB}}$	10	1.0000	8	1.0000	6	1.0000
	GPT-J$_{\text{SURP}^{1}}$ vs. GPT-J$_{\text{PROB}}$	12	1.0000	6	1.0000	7	1.0000
	GPT-J$_{\text{SURP}^{4/3}}$ vs. GPT-J$_{\text{PROB}}$	14	1.0000	5	1.0000	10	1.0000
	GPT-J$_{\text{SURP}^{2}}$ vs. GPT-J$_{\text{PROB}}$	16	1.0000	3	1.0000	12	1.0000
	GPT-J$_{\text{SURP}^{3/4}}$ vs. GPT-J$_{\text{SURP}^{1/2}}$	2	1.0000	1	1.0000	1	1.0000
	GPT-J$_{\text{SURP}^{1}}$ vs. GPT-J$_{\text{SURP}^{1/2}}$	4	1.0000	-1	1.0000	2	1.0000
	GPT-J$_{\text{SURP}^{4/3}}$ vs. GPT-J$_{\text{SURP}^{1/2}}$	6	1.0000	-2	1.0000	4	1.0000
	GPT-J$_{\text{SURP}^{2}}$ vs. GPT-J$_{\text{SURP}^{1/2}}$	8	1.0000	-4	1.0000	7	1.0000
	GPT-J$_{\text{SURP}^{1}}$ vs. GPT-J$_{\text{SURP}^{3/4}}$	2	1.0000	-1	1.0000	1	1.0000
	GPT-J$_{\text{SURP}^{4/3}}$ vs. GPT-J$_{\text{SURP}^{3/4}}$	4	1.0000	-3	1.0000	4	1.0000
	GPT-J$_{\text{SURP}^{2}}$ vs. GPT-J$_{\text{SURP}^{3/4}}$	6	1.0000	-5	1.0000	6	1.0000
	GPT-J$_{\text{SURP}^{4/3}}$ vs. GPT-J$_{\text{SURP}^{1}}$	2	1.0000	-2	1.0000	2	1.0000
	GPT-J$_{\text{SURP}^{2}}$ vs. GPT-J$_{\text{SURP}^{1}}$	4	1.0000	-4	1.0000	5	1.0000
	GPT-J$_{\text{SURP}^{2}}$ vs. GPT-J$_{\text{SURP}^{4/3}}$	2	1.0000	-2	1.0000	3	1.0000
GPT-3	GPT-3$_{f(\text{SURP})}$ vs. ∅	16	1.0000	20	1.0000	1	1.0000
	GPT-3$_{f(\text{SURP})}$ vs. GPT-3$_{\text{PROB}}$	11	1.0000	10	1.0000	1	1.0000
	GPT-3$_{f(\text{SURP})}$ vs. GPT-3$_{\text{SURP}^{1/2}}$	3	1.0000	1	1.0000	-1	—
	GPT-3$_{f(\text{SURP})}$ vs. GPT-3$_{\text{SURP}^{3/4}}$	0	1.0000	0	1.0000	1	1.0000
	GPT-3$_{f(\text{SURP})}$ vs. GPT-3$_{\text{SURP}^{1}}$	-2	—	0	1.0000	2	1.0000
	GPT-3$_{f(\text{SURP})}$ vs. GPT-3$_{\text{SURP}^{4/3}}$	-3	—	3	1.0000	3	1.0000
	GPT-3$_{f(\text{SURP})}$ vs. GPT-3$_{\text{SURP}^{2}}$	-6	—	7	1.0000	3	1.0000
	GPT-3$_{\text{PROB}}$ vs. ∅	6	1.0000	10	1.0000	0	1.0000
	GPT-3$_{\text{SURP}^{1/2}}$ vs. ∅	13	1.0000	19	1.0000	2	1.0000
	GPT-3$_{\text{SURP}^{3/4}}$ vs. ∅	16	1.0000	20	1.0000	0	1.0000
	GPT-3$_{\text{SURP}^{1}}$ vs. ∅	18	1.0000	20	1.0000	-1	—
	GPT-3$_{\text{SURP}^{4/3}}$ vs. ∅	20	1.0000	16	1.0000	-1	—
	GPT-3$_{\text{SURP}^{2}}$ vs. ∅	22	1.0000	13	1.0000	-1	—
	GPT-3$_{\text{SURP}^{1/2}}$ vs. GPT-3$_{\text{PROB}}$	7	1.0000	9	1.0000	2	1.0000
	GPT-3$_{\text{SURP}^{3/4}}$ vs. GPT-3$_{\text{PROB}}$	11	1.0000	10	1.0000	0	1.0000
	GPT-3$_{\text{SURP}^{1}}$ vs. GPT-3$_{\text{PROB}}$	13	1.0000	10	1.0000	-1	1.0000
	GPT-3$_{\text{SURP}^{4/3}}$ vs. GPT-3$_{\text{PROB}}$	14	1.0000	6	1.0000	-1	1.0000
	GPT-3$_{\text{SURP}^{2}}$ vs. GPT-3$_{\text{PROB}}$	17	1.0000	3	1.0000	-1	1.0000
	GPT-3$_{\text{SURP}^{3/4}}$ vs. GPT-3$_{\text{SURP}^{1/2}}$	3	1.0000	1	1.0000	-2	1.0000
	GPT-3$_{\text{SURP}^{1}}$ vs. GPT-3$_{\text{SURP}^{1/2}}$	5	1.0000	1	1.0000	-3	1.0000
	GPT-3$_{\text{SURP}^{4/3}}$ vs. GPT-3$_{\text{SURP}^{1/2}}$	7	1.0000	-3	1.0000	-4	1.0000
	GPT-3$_{\text{SURP}^{2}}$ vs. GPT-3$_{\text{SURP}^{1/2}}$	9	1.0000	-6	1.0000	-4	1.0000
	GPT-3$_{\text{SURP}^{1}}$ vs. GPT-3$_{\text{SURP}^{3/4}}$	2	1.0000	0	1.0000	-1	1.0000
	GPT-3$_{\text{SURP}^{4/3}}$ vs. GPT-3$_{\text{SURP}^{3/4}}$	3	1.0000	-3	1.0000	-2	1.0000
	GPT-3$_{\text{SURP}^{2}}$ vs. GPT-3$_{\text{SURP}^{3/4}}$	6	1.0000	-7	1.0000	-2	1.0000
	GPT-3$_{\text{SURP}^{4/3}}$ vs. GPT-3$_{\text{SURP}^{1}}$	2	1.0000	-4	1.0000	-1	1.0000
	GPT-3$_{\text{SURP}^{2}}$ vs. GPT-3$_{\text{SURP}^{1}}$	4	1.0000	-7	1.0000	-1	1.0000
	GPT-3$_{\text{SURP}^{2}}$ vs. GPT-3$_{\text{SURP}^{4/3}}$	3	1.0000	-3	1.0000	0	1.0000

	Comparison	Scan Path		First Pass		Go Past	
		ΔLL	p	ΔLL	p	ΔLL	p
All LMs	All-LMs$_{f(\text{SURP})}$ vs. ∅	18	1.0000	20	1.0000	2	1.0000
	All-LMs$_{f(\text{SURP})}$ vs. All-LMs$_{\text{PROB}}$	13	1.0000	13	1.0000	2	1.0000
	All-LMs$_{f(\text{SURP})}$ vs. All-LMs$_{\text{SURP}^{1/2}}$	8	1.0000	6	1.0000	-1	—
	All-LMs$_{f(\text{SURP})}$ vs. All-LMs$_{\text{SURP}^{3/4}}$	7	1.0000	6	1.0000	1	1.0000
	All-LMs$_{f(\text{SURP})}$ vs. All-LMs$_{\text{SURP}^{1}}$	5	1.0000	7	1.0000	2	1.0000
	All-LMs$_{f(\text{SURP})}$ vs. All-LMs$_{\text{SURP}^{4/3}}$	3	1.0000	9	1.0000	0	1.0000
	All-LMs$_{f(\text{SURP})}$ vs. All-LMs$_{\text{SURP}^{2}}$	0	1.0000	7	1.0000	-3	—
	All-LMs$_{\text{PROB}}$ vs. ∅	5	1.0000	6	1.0000	0	1.0000
	All-LMs$_{\text{SURP}^{1/2}}$ vs. ∅	10	1.0000	13	1.0000	2	1.0000
	All-LMs$_{\text{SURP}^{3/4}}$ vs. ∅	11	1.0000	14	1.0000	0	1.0000
	All-LMs$_{\text{SURP}^{1}}$ vs. ∅	13	1.0000	13	1.0000	0	1.0000
	All-LMs$_{\text{SURP}^{4/3}}$ vs. ∅	15	1.0000	11	1.0000	2	1.0000
	All-LMs$_{\text{SURP}^{2}}$ vs. ∅	18	1.0000	12	1.0000	5	1.0000
	All-LMs$_{\text{SURP}^{1/2}}$ vs. All-LMs$_{\text{PROB}}$	5	1.0000	7	1.0000	2	1.0000
	All-LMs$_{\text{SURP}^{3/4}}$ vs. All-LMs$_{\text{PROB}}$	6	1.0000	8	1.0000	0	1.0000
	All-LMs$_{\text{SURP}^{1}}$ vs. All-LMs$_{\text{PROB}}$	8	1.0000	6	1.0000	0	1.0000
	All-LMs$_{\text{SURP}^{4/3}}$ vs. All-LMs$_{\text{PROB}}$	10	1.0000	5	1.0000	2	1.0000
	All-LMs$_{\text{SURP}^{2}}$ vs. All-LMs$_{\text{PROB}}$	12	1.0000	6	1.0000	5	1.0000
	All-LMs$_{\text{SURP}^{3/4}}$ vs. All-LMs$_{\text{SURP}^{1/2}}$	1	1.0000	1	1.0000	-2	1.0000
	All-LMs$_{\text{SURP}^{1}}$ vs. All-LMs$_{\text{SURP}^{1/2}}$	3	1.0000	-1	1.0000	-2	1.0000
	All-LMs$_{\text{SURP}^{4/3}}$ vs. All-LMs$_{\text{SURP}^{1/2}}$	5	1.0000	-2	1.0000	0	1.0000
	All-LMs$_{\text{SURP}^{2}}$ vs. All-LMs$_{\text{SURP}^{1/2}}$	7	1.0000	-1	1.0000	2	1.0000
	All-LMs$_{\text{SURP}^{1}}$ vs. All-LMs$_{\text{SURP}^{3/4}}$	2	1.0000	-1	1.0000	0	1.0000
	All-LMs$_{\text{SURP}^{4/3}}$ vs. All-LMs$_{\text{SURP}^{3/4}}$	4	1.0000	-3	1.0000	2	1.0000
	All-LMs$_{\text{SURP}^{2}}$ vs. All-LMs$_{\text{SURP}^{3/4}}$	6	1.0000	-2	1.0000	4	1.0000
	All-LMs$_{\text{SURP}^{4/3}}$ vs. All-LMs$_{\text{SURP}^{1}}$	2	1.0000	-2	1.0000	2	1.0000
	All-LMs$_{\text{SURP}^{2}}$ vs. All-LMs$_{\text{SURP}^{1}}$	4	1.0000	0	1.0000	5	1.0000
	All-LMs$_{\text{SURP}^{2}}$ vs. All-LMs$_{\text{SURP}^{4/3}}$	2	1.0000	1	1.0000	3	1.0000

Table S14. Testing Results on Provo (Eye-Tracking) using generalized additive models (GAMs). Results of key statistical comparisons based on permutation tests of the difference in average test set likelihood between the alternative and null GAM model in each comparison (△LL). Subscripts indicate the assumed functional form of predictability effects: $f(\text{SURP})$ (nonlinear in surprisal), PROB (linear in probability), and SURP^a (linear in surprisal raised to exponent a, such that e.g., SURP^1 is linear in surprisal). Boldface indicates statistical significance. In directional tests where one hypothesis subsumes the other (e.g., $f(\text{SURP})$ vs. SURP^1, since a nonlinear surprisal effect subsumes a linear one), dashes (—) indicate failure of the alternative hypothesis (left) to improve over the null hypothesis (right). Otherwise, cells are color coded using cyan to indicate that the hypothesis on the left outperforms the one on the right, and using magenta to indicate that the hypothesis on the right outperforms the one on the left. All p-values are corrected for false discovery rate (15) within each family of tests (delimited by single horizontal lines). Key comparisons are highlighted.

Group	Comparison	Combined Datasets ΔLL	p
n-gram	*n*-gram$_{f(\text{SURP})}$ vs. $\emptyset$	**1130**	**0.0005**
n-gram	*n*-gram$_{f(\text{SURP})}$ vs. *n*-gram$_{\text{PROB}}$	**1219**	**0.0005**
n-gram	*n*-gram$_{f(\text{SURP})}$ vs. *n*-gram$_{\text{SURP}^{1/2}}$	**250**	**0.0005**
n-gram	*n*-gram$_{f(\text{SURP})}$ vs. *n*-gram$_{\text{SURP}^{3/4}}$	**206**	**0.0005**
n-gram	*n*-gram$_{f(\text{SURP})}$ vs. *n*-gram$_{\text{SURP}^{1}}$	**214**	**0.0005**
n-gram	*n*-gram$_{f(\text{SURP})}$ vs. *n*-gram$_{\text{SURP}^{4/3}}$	**299**	**0.0005**
n-gram	*n*-gram$_{f(\text{SURP})}$ vs. *n*-gram$_{\text{SURP}^{2}}$	**629**	**0.0005**
n-gram	*n*-gram$_{\text{PROB}}$ vs. $\emptyset$	-89	—
n-gram	*n*-gram$_{\text{SURP}^{1/2}}$ vs. $\emptyset$	**880**	**0.0005**
n-gram	*n*-gram$_{\text{SURP}^{3/4}}$ vs. $\emptyset$	**924**	**0.0005**
n-gram	*n*-gram$_{\text{SURP}^{1}}$ vs. $\emptyset$	**916**	**0.0005**
n-gram	*n*-gram$_{\text{SURP}^{4/3}}$ vs. $\emptyset$	**831**	**0.0005**
n-gram	*n*-gram$_{\text{SURP}^{2}}$ vs. $\emptyset$	**500**	**0.0005**
n-gram	*n*-gram$_{\text{SURP}^{1/2}}$ vs. *n*-gram$_{\text{PROB}}$	**969**	**0.0005**
n-gram	*n*-gram$_{\text{SURP}^{3/4}}$ vs. *n*-gram$_{\text{PROB}}$	**1013**	**0.0005**
n-gram	*n*-gram$_{\text{SURP}^{1}}$ vs. *n*-gram$_{\text{PROB}}$	**1005**	**0.0005**
n-gram	*n*-gram$_{\text{SURP}^{4/3}}$ vs. *n*-gram$_{\text{PROB}}$	**920**	**0.0005**
n-gram	*n*-gram$_{\text{SURP}^{2}}$ vs. *n*-gram$_{\text{PROB}}$	**590**	**0.0005**
n-gram	*n*-gram$_{\text{SURP}^{3/4}}$ vs. *n*-gram$_{\text{SURP}^{1/2}}$	**44**	**0.0137**
n-gram	*n*-gram$_{\text{SURP}^{1}}$ vs. *n*-gram$_{\text{SURP}^{1/2}}$	36	0.8809
n-gram	*n*-gram$_{\text{SURP}^{4/3}}$ vs. *n*-gram$_{\text{SURP}^{1/2}}$	-49	1.0000
n-gram	*n*-gram$_{\text{SURP}^{2}}$ vs. *n*-gram$_{\text{SURP}^{1/2}}$	**-379**	**0.0005**
n-gram	*n*-gram$_{\text{SURP}^{1}}$ vs. *n*-gram$_{\text{SURP}^{3/4}}$	-8	1.0000
n-gram	*n*-gram$_{\text{SURP}^{4/3}}$ vs. *n*-gram$_{\text{SURP}^{3/4}}$	**-93**	**0.0086**
n-gram	*n*-gram$_{\text{SURP}^{2}}$ vs. *n*-gram$_{\text{SURP}^{3/4}}$	**-423**	**0.0005**
n-gram	*n*-gram$_{\text{SURP}^{4/3}}$ vs. *n*-gram$_{\text{SURP}^{1}}$	**-85**	**0.0005**
n-gram	*n*-gram$_{\text{SURP}^{2}}$ vs. *n*-gram$_{\text{SURP}^{1}}$	**-415**	**0.0005**
n-gram	*n*-gram$_{\text{SURP}^{2}}$ vs. *n*-gram$_{\text{SURP}^{4/3}}$	**-330**	**0.0005**
PCFG	PCFG$_{f(\text{SURP})}$ vs. $\emptyset$	**694**	**0.0005**
PCFG	PCFG$_{f(\text{SURP})}$ vs. PCFG$_{\text{PROB}}$	**663**	**0.0005**
PCFG	PCFG$_{f(\text{SURP})}$ vs. PCFG$_{\text{SURP}^{1/2}}$	**382**	**0.0005**
PCFG	PCFG$_{f(\text{SURP})}$ vs. PCFG$_{\text{SURP}^{3/4}}$	**378**	**0.0005**
PCFG	PCFG$_{f(\text{SURP})}$ vs. PCFG$_{\text{SURP}^{1}}$	**381**	**0.0005**
PCFG	PCFG$_{f(\text{SURP})}$ vs. PCFG$_{\text{SURP}^{4/3}}$	**401**	**0.0005**
PCFG	PCFG$_{f(\text{SURP})}$ vs. PCFG$_{\text{SURP}^{2}}$	**472**	**0.0005**
PCFG	PCFG$_{\text{PROB}}$ vs. $\emptyset$	**30**	**0.0005**
PCFG	PCFG$_{\text{SURP}^{1/2}}$ vs. $\emptyset$	**312**	**0.0005**
PCFG	PCFG$_{\text{SURP}^{3/4}}$ vs. $\emptyset$	**315**	**0.0005**
PCFG	PCFG$_{\text{SURP}^{1}}$ vs. $\emptyset$	**313**	**0.0005**
PCFG	PCFG$_{\text{SURP}^{4/3}}$ vs. $\emptyset$	**293**	**0.0005**
PCFG	PCFG$_{\text{SURP}^{2}}$ vs. $\emptyset$	**222**	**0.0005**
PCFG	PCFG$_{\text{SURP}^{1/2}}$ vs. PCFG$_{\text{PROB}}$	**281**	**0.0005**
PCFG	PCFG$_{\text{SURP}^{3/4}}$ vs. PCFG$_{\text{PROB}}$	**285**	**0.0005**
PCFG	PCFG$_{\text{SURP}^{1}}$ vs. PCFG$_{\text{PROB}}$	**282**	**0.0005**
PCFG	PCFG$_{\text{SURP}^{4/3}}$ vs. PCFG$_{\text{PROB}}$	**262**	**0.0005**
PCFG	PCFG$_{\text{SURP}^{2}}$ vs. PCFG$_{\text{PROB}}$	**192**	**0.0005**
PCFG	PCFG$_{\text{SURP}^{3/4}}$ vs. PCFG$_{\text{SURP}^{1/2}}$	4	1.0000
PCFG	PCFG$_{\text{SURP}^{1}}$ vs. PCFG$_{\text{SURP}^{1/2}}$	1	1.0000
PCFG	PCFG$_{\text{SURP}^{4/3}}$ vs. PCFG$_{\text{SURP}^{1/2}}$	-19	1.0000
PCFG	PCFG$_{\text{SURP}^{2}}$ vs. PCFG$_{\text{SURP}^{1/2}}$	**-90**	**0.0135**
PCFG	PCFG$_{\text{SURP}^{1}}$ vs. PCFG$_{\text{SURP}^{3/4}}$	-3	1.0000
PCFG	PCFG$_{\text{SURP}^{4/3}}$ vs. PCFG$_{\text{SURP}^{3/4}}$	-23	0.4714
PCFG	PCFG$_{\text{SURP}^{2}}$ vs. PCFG$_{\text{SURP}^{3/4}}$	**-93**	**0.0010**
PCFG	PCFG$_{\text{SURP}^{4/3}}$ vs. PCFG$_{\text{SURP}^{1}}$	**-20**	**0.0416**
PCFG	PCFG$_{\text{SURP}^{2}}$ vs. PCFG$_{\text{SURP}^{1}}$	**-91**	**0.0005**
PCFG	PCFG$_{\text{SURP}^{2}}$ vs. PCFG$_{\text{SURP}^{4/3}}$	**-71**	**0.0005**
GPT-2	GPT-2$_{f(\text{SURP})}$ vs. $\emptyset$	**2797**	**0.0005**
GPT-2	GPT-2$_{f(\text{SURP})}$ vs. GPT-2$_{\text{PROB}}$	**2473**	**0.0005**
GPT-2	GPT-2$_{f(\text{SURP})}$ vs. GPT-2$_{\text{SURP}^{1/2}}$	**706**	**0.0005**
GPT-2	GPT-2$_{f(\text{SURP})}$ vs. GPT-2$_{\text{SURP}^{3/4}}$	**433**	**0.0005**
GPT-2	GPT-2$_{f(\text{SURP})}$ vs. GPT-2$_{\text{SURP}^{1}}$	**276**	**0.0005**
GPT-2	GPT-2$_{f(\text{SURP})}$ vs. GPT-2$_{\text{SURP}^{4/3}}$	**231**	**0.0005**
GPT-2	GPT-2$_{f(\text{SURP})}$ vs. GPT-$_{\text{SURP}^{2}}$	**580**	**0.0005**
GPT-2	GPT-2$_{\text{PROB}}$ vs. $\emptyset$	324	0.1944
GPT-2	GPT-2$_{\text{SURP}^{1/2}}$ vs. $\emptyset$	**2091**	**0.0005**
GPT-2	GPT-2$_{\text{SURP}^{3/4}}$ vs. $\emptyset$	**2364**	**0.0005**
GPT-2	GPT-2$_{\text{SURP}^{1}}$ vs. $\emptyset$	**2521**	**0.0005**
GPT-2	GPT-2$_{\text{SURP}^{4/3}}$ vs. $\emptyset$	**2567**	**0.0005**
GPT-2	GPT-$_{\text{SURP}^{2}}$ vs. $\emptyset$	**2218**	**0.0005**
GPT-2	GPT-2$_{\text{SURP}^{1/2}}$ vs. GPT-2$_{\text{PROB}}$	**1767**	**0.0005**
GPT-2	GPT-2$_{\text{SURP}^{3/4}}$ vs. GPT-2$_{\text{PROB}}$	**2041**	**0.0005**
GPT-2	GPT-2$_{\text{SURP}^{1}}$ vs. GPT-2$_{\text{PROB}}$	**2198**	**0.0005**
GPT-2	GPT-2$_{\text{SURP}^{4/3}}$ vs. GPT-2$_{\text{PROB}}$	**2243**	**0.0005**
GPT-2	GPT-$_{\text{SURP}^{2}}$ vs. GPT-2$_{\text{PROB}}$	**1894**	**0.0005**
GPT-2	GPT-2$_{\text{SURP}^{3/4}}$ vs. GPT-2$_{\text{SURP}^{1/2}}$	**273**	**0.0005**
GPT-2	GPT-2$_{\text{SURP}^{1}}$ vs. GPT-2$_{\text{SURP}^{1/2}}$	**431**	**0.0005**
GPT-2	GPT-2$_{\text{SURP}^{4/3}}$ vs. GPT-2$_{\text{SURP}^{1/2}}$	**476**	**0.0005**
GPT-2	GPT-$_{\text{SURP}^{2}}$ vs. GPT-2$_{\text{SURP}^{1/2}}$	127	0.7025
GPT-2	GPT-2$_{\text{SURP}^{1}}$ vs. GPT-2$_{\text{SURP}^{3/4}}$	**157**	**0.0005**
GPT-2	GPT-2$_{\text{SURP}^{4/3}}$ vs. GPT-2$_{\text{SURP}^{3/4}}$	**202**	**0.0005**
GPT-2	GPT-$_{\text{SURP}^{2}}$ vs. GPT-2$_{\text{SURP}^{3/4}}$	-147	0.2993
GPT-2	GPT-2$_{\text{SURP}^{4/3}}$ vs. GPT-2$_{\text{SURP}^{1}}$	45	0.2279
GPT-2	GPT-$_{\text{SURP}^{2}}$ vs. GPT-2$_{\text{SURP}^{1}}$	**-304**	**0.0005**
GPT-2	GPT-$_{\text{SURP}^{2}}$ vs. GPT-2$_{\text{SURP}^{4/3}}$	**-349**	**0.0005**
GPT-J	GPT-J$_{f(\text{SURP})}$ vs. $\emptyset$	**2045**	**0.0005**
GPT-J	GPT-J$_{f(\text{SURP})}$ vs. GPT-J$_{\text{PROB}}$	**1372**	**0.0005**
GPT-J	GPT-J$_{f(\text{SURP})}$ vs. GPT-J$_{\text{SURP}^{1/2}}$	**489**	**0.0005**
GPT-J	GPT-J$_{f(\text{SURP})}$ vs. GPT-J$_{\text{SURP}^{3/4}}$	**303**	**0.0005**
GPT-J	GPT-J$_{f(\text{SURP})}$ vs. GPT-J$_{\text{SURP}^{1}}$	**197**	**0.0005**
GPT-J	GPT-J$_{f(\text{SURP})}$ vs. GPT-J$_{\text{SURP}^{4/3}}$	**147**	**0.0005**
GPT-J	GPT-J$_{f(\text{SURP})}$ vs. GPT-J$_{\text{SURP}^{2}}$	**280**	**0.0005**
GPT-J	GPT-J$_{\text{PROB}}$ vs. $\emptyset$	**672**	**0.0005**
GPT-J	GPT-J$_{\text{SURP}^{1/2}}$ vs. $\emptyset$	**1556**	**0.0005**
GPT-J	GPT-J$_{\text{SURP}^{3/4}}$ vs. $\emptyset$	**1742**	**0.0005**
GPT-J	GPT-J$_{\text{SURP}^{1}}$ vs. $\emptyset$	**1848**	**0.0005**
GPT-J	GPT-J$_{\text{SURP}^{4/3}}$ vs. $\emptyset$	**1898**	**0.0005**
GPT-J	GPT-J$_{\text{SURP}^{2}}$ vs. $\emptyset$	**1765**	**0.0005**
GPT-J	GPT-J$_{\text{SURP}^{1/2}}$ vs. GPT-J$_{\text{PROB}}$	**883**	**0.0005**
GPT-J	GPT-J$_{\text{SURP}^{3/4}}$ vs. GPT-J$_{\text{PROB}}$	**1069**	**0.0005**
GPT-J	GPT-J$_{\text{SURP}^{1}}$ vs. GPT-J$_{\text{PROB}}$	**1176**	**0.0005**
GPT-J	GPT-J$_{\text{SURP}^{4/3}}$ vs. GPT-J$_{\text{PROB}}$	**1225**	**0.0005**
GPT-J	GPT-J$_{\text{SURP}^{2}}$ vs. GPT-J$_{\text{PROB}}$	**1093**	**0.0005**
GPT-J	GPT-J$_{\text{SURP}^{3/4}}$ vs. GPT-J$_{\text{SURP}^{1/2}}$	**186**	**0.0005**
GPT-J	GPT-J$_{\text{SURP}^{1}}$ vs. GPT-J$_{\text{SURP}^{1/2}}$	**293**	**0.0005**
GPT-J	GPT-J$_{\text{SURP}^{4/3}}$ vs. GPT-J$_{\text{SURP}^{1/2}}$	**342**	**0.0005**
GPT-J	GPT-J$_{\text{SURP}^{2}}$ vs. GPT-J$_{\text{SURP}^{1/2}}$	**209**	**0.0207**
GPT-J	GPT-J$_{\text{SURP}^{1}}$ vs. GPT-J$_{\text{SURP}^{3/4}}$	**106**	**0.0005**
GPT-J	GPT-J$_{\text{SURP}^{4/3}}$ vs. GPT-J$_{\text{SURP}^{3/4}}$	**156**	**0.0005**
GPT-J	GPT-J$_{\text{SURP}^{2}}$ vs. GPT-J$_{\text{SURP}^{3/4}}$	23	1.0000
GPT-J	GPT-J$_{\text{SURP}^{4/3}}$ vs. GPT-J$_{\text{SURP}^{1}}$	**49**	**0.0279**
GPT-J	GPT-J$_{\text{SURP}^{2}}$ vs. GPT-J$_{\text{SURP}^{1}}$	-83	0.3710
GPT-J	GPT-J$_{\text{SURP}^{2}}$ vs. GPT-J$_{\text{SURP}^{4/3}}$	**-133**	**0.0005**
GPT-3	GPT-3$_{f(\text{SURP})}$ vs. $\emptyset$	**1522**	**0.0005**
GPT-3	GPT-3$_{f(\text{SURP})}$ vs. GPT-3$_{\text{PROB}}$	**853**	**0.0005**
GPT-3	GPT-3$_{f(\text{SURP})}$ vs. GPT-3$_{\text{SURP}^{1/2}}$	**355**	**0.0005**
GPT-3	GPT-3$_{f(\text{SURP})}$ vs. GPT-3$_{\text{SURP}^{3/4}}$	**256**	**0.0005**
GPT-3	GPT-3$_{f(\text{SURP})}$ vs. GPT-3$_{\text{SURP}^{1}}$	**199**	**0.0005**
GPT-3	GPT-3$_{f(\text{SURP})}$ vs. GPT-3$_{\text{SURP}^{4/3}}$	**173**	**0.0005**
GPT-3	GPT-3$_{f(\text{SURP})}$ vs. GPT-3$_{\text{SURP}^{2}}$	**234**	**0.0005**
GPT-3	GPT-3$_{\text{PROB}}$ vs. $\emptyset$	**669**	**0.0005**
GPT-3	GPT-3$_{\text{SURP}^{1/2}}$ vs. $\emptyset$	**1166**	**0.0005**
GPT-3	GPT-3$_{\text{SURP}^{3/4}}$ vs. $\emptyset$	**1265**	**0.0005**
GPT-3	GPT-3$_{\text{SURP}^{1}}$ vs. $\emptyset$	**1322**	**0.0005**
GPT-3	GPT-3$_{\text{SURP}^{4/3}}$ vs. $\emptyset$	**1349**	**0.0005**
GPT-3	GPT-3$_{\text{SURP}^{2}}$ vs. $\emptyset$	**1287**	**0.0005**
GPT-3	GPT-3$_{\text{SURP}^{1/2}}$ vs. GPT-3$_{\text{PROB}}$	**498**	**0.0005**
GPT-3	GPT-3$_{\text{SURP}^{3/4}}$ vs. GPT-3$_{\text{PROB}}$	**596**	**0.0005**
GPT-3	GPT-3$_{\text{SURP}^{1}}$ vs. GPT-3$_{\text{PROB}}$	**654**	**0.0005**
GPT-3	GPT-3$_{\text{SURP}^{4/3}}$ vs. GPT-3$_{\text{PROB}}$	**680**	**0.0005**
GPT-3	GPT-3$_{\text{SURP}^{2}}$ vs. GPT-3$_{\text{PROB}}$	**618**	**0.0005**
GPT-3	GPT-3$_{\text{SURP}^{3/4}}$ vs. GPT-3$_{\text{SURP}^{1/2}}$	**99**	**0.0005**
GPT-3	GPT-3$_{\text{SURP}^{1}}$ vs. GPT-3$_{\text{SURP}^{1/2}}$	**156**	**0.0005**
GPT-3	GPT-3$_{\text{SURP}^{4/3}}$ vs. GPT-3$_{\text{SURP}^{1/2}}$	**183**	**0.0005**
GPT-3	GPT-3$_{\text{SURP}^{2}}$ vs. GPT-3$_{\text{SURP}^{1/2}}$	121	0.3153
GPT-3	GPT-3$_{\text{SURP}^{1}}$ vs. GPT-3$_{\text{SURP}^{3/4}}$	**57**	**0.0005**
GPT-3	GPT-3$_{\text{SURP}^{4/3}}$ vs. GPT-3$_{\text{SURP}^{3/4}}$	**84**	**0.0210**
GPT-3	GPT-3$_{\text{SURP}^{2}}$ vs. GPT-3$_{\text{SURP}^{3/4}}$	22	1.0000
GPT-3	GPT-3$_{\text{SURP}^{4/3}}$ vs. GPT-3$_{\text{SURP}^{1}}$	27	0.4453
GPT-3	GPT-3$_{\text{SURP}^{2}}$ vs. GPT-3$_{\text{SURP}^{1}}$	-35	1.0000
GPT-3	GPT-3$_{\text{SURP}^{2}}$ vs. GPT-3$_{\text{SURP}^{4/3}}$	-62	0.1063
All LMs	All-LMs$_{f(\text{SURP})}$ vs. $\emptyset$	**1522**	**0.0006**
All LMs	All-LMs$_{f(\text{SURP})}$ vs. All-LMs$_{\text{PROB}}$	**1198**	**0.0006**
All LMs	All-LMs$_{f(\text{SURP})}$ vs. All-LMs$_{\text{SURP}^{1/2}}$	**355**	**0.0006**
All LMs	All-LMs$_{f(\text{SURP})}$ vs. All-LMs$_{\text{SURP}^{3/4}}$	**256**	**0.0006**
All LMs	All-LMs$_{f(\text{SURP})}$ vs. All-LMs$_{\text{SURP}^{1}}$	**199**	**0.0006**
All LMs	All-LMs$_{f(\text{SURP})}$ vs. All-LMs$_{\text{SURP}^{4/3}}$	**173**	**0.0017**
All LMs	All-LMs$_{f(\text{SURP})}$ vs. All-LMs$_{\text{SURP}^{2}}$	**234**	**0.0006**
All LMs	All-LMs$_{\text{PROB}}$ vs. $\emptyset$	**324**	**0.0033**
All LMs	All-LMs$_{\text{SURP}^{1/2}}$ vs. $\emptyset$	**1166**	**0.0006**
All LMs	All-LMs$_{\text{SURP}^{3/4}}$ vs. $\emptyset$	**1265**	**0.0006**
All LMs	All-LMs$_{\text{SURP}^{1}}$ vs. $\emptyset$	**1322**	**0.0006**
All LMs	All-LMs$_{\text{SURP}^{4/3}}$ vs. $\emptyset$	**1349**	**0.0006**
All LMs	All-LMs$_{\text{SURP}^{2}}$ vs. $\emptyset$	**1287**	**0.0006**
All LMs	All-LMs$_{\text{SURP}^{1/2}}$ vs. All-LMs$_{\text{PROB}}$	**843**	**0.0006**
All LMs	All-LMs$_{\text{SURP}^{3/4}}$ vs. All-LMs$_{\text{PROB}}$	**941**	**0.0006**
All LMs	All-LMs$_{\text{SURP}^{1}}$ vs. All-LMs$_{\text{PROB}}$	**999**	**0.0006**
All LMs	All-LMs$_{\text{SURP}^{4/3}}$ vs. All-LMs$_{\text{PROB}}$	**1025**	**0.0006**
All LMs	All-LMs$_{\text{SURP}^{2}}$ vs. All-LMs$_{\text{PROB}}$	**963**	**0.0006**
All LMs	All-LMs$_{\text{SURP}^{3/4}}$ vs. All-LMs$_{\text{SURP}^{1/2}}$	**99**	**0.0288**
All LMs	All-LMs$_{\text{SURP}^{1}}$ vs. All-LMs$_{\text{SURP}^{1/2}}$	**156**	**0.0012**
All LMs	All-LMs$_{\text{SURP}^{4/3}}$ vs. All-LMs$_{\text{SURP}^{1/2}}$	**183**	**0.0006**
All LMs	All-LMs$_{\text{SURP}^{2}}$ vs. All-LMs$_{\text{SURP}^{1/2}}$	121	0.0600
All LMs	All-LMs$_{\text{SURP}^{1}}$ vs. All-LMs$_{\text{SURP}^{3/4}}$	57	0.6917
All LMs	All-LMs$_{\text{SURP}^{4/3}}$ vs. All-LMs$_{\text{SURP}^{3/4}}$	84	0.2476
All LMs	All-LMs$_{\text{SURP}^{2}}$ vs. All-LMs$_{\text{SURP}^{3/4}}$	22	1.0000
All LMs	All-LMs$_{\text{SURP}^{4/3}}$ vs. All-LMs$_{\text{SURP}^{1}}$	27	1.0000
All LMs	All-LMs$_{\text{SURP}^{2}}$ vs. All-LMs$_{\text{SURP}^{1}}$	-35	1.0000
All LMs	All-LMs$_{\text{SURP}^{2}}$ vs. All-LMs$_{\text{SURP}^{4/3}}$	-62	1.0000

Table S15. Testing Results Across All Datasets using generalized additive models (GAMs). Results of key statistical comparisons based on permutation tests of the difference in average test set likelihood between the alternative and null GAM model in each comparison (ΔLL), aggregating all dependent variables considered in this study in each test (see Supplementary Information 13 for details). Subscripts indicate the assumed functional form of predictability effects: $f(\text{SURP})$ (nonlinear in surprisal), PROB (linear in probability), and SURP^{a} (linear in surprisal raised to exponent a, such that e.g., SURP^{1} is linear in surprisal). Boldface indicates statistical significance. In directional tests where one hypothesis subsumes the other (e.g., $f(\text{SURP})$ vs. SURP^{1}, since a nonlinear surprisal effect subsumes a linear one), dashes (—) indicate failure of the alternative hypothesis (left) to improve over the null hypothesis (right). Otherwise, cells are color coded using cyan to indicate that the hypothesis on the left outperforms the one on the right, and using magenta to indicate that the hypothesis on the right outperforms the one on the left. All p-values are corrected for false discovery rate (15) within each family of tests (delimited by single horizontal lines). Key comparisons are highlighted.

15. Reanalysis Using Normal Error

To better understand the potential impact on results of our assumed exGaussian distribution over reading times—which is motivated by prior considerations from psycholinguistic research (e.g., 74, 75) but deviates from the more widely assumed normal distribution, we re-ran the GPT-2 subset of our main analyses assuming a normal rather than exGaussian distribution. The estimated form of GPT-2 surprisal effects under both distributions are plotted in **Figure S11**. As shown, estimates are highly stable across distributions.

Tables S5–S9 report key results of testing GPT-2 models under a normal distribution. In general, a similar qualitative pattern of results holds across both distributions, and most of the key results survive (e.g., $f(\textsc{surp})$ outperforms both $\emptyset$ and PROB, and linear or sublogarithmic models outperform superlinear ones). Nonetheless, fewer of these results are significant, suggesting that the exGaussian analyses may be more sensitive. In any case, the exGaussian results are more credible on the basis of generalization performance: as shown in the final row of each table, the exGaussian GPT-2 $f(\textsc{surp})$ model obtains a test set log likelihood improvement of several thousand points over the normal GPT-2 $f(\textsc{surp})$ model in every comparison (all $p < 0.0001$), accumulating to an improvement of over 280,000 log likelihood points in the combined dataset (**Table S9**). Thus, our results suggest that better models of the underlying data distribution not only improve fit, but also the statistical power of critical tests. This outcome could be of relevance to future analysis design for similarly skewed response time data.

References

1. T Brothers, GR Kuperberg, Word predictability effects are linear, not logarithmic: Implications for probabilistic models of sentence comprehension. *J. Mem. Lang.* **116**, 104174 (2021).
2. NJ Smith, R Levy, The effect of word predictability on reading time is logarithmic. *Cognition* **128**, 302–319 (2013).
3. EG Wilcox, J Gauthier, J Hu, P Qian, R Levy, On the Predictive Power of Neural Language Models for Human Real-Time Comprehension Behavior in *Proceedings of the 42nd Annual Meeting of the Cognitive Science Society.* p. 1707–1713 (2020).
4. C Meister, et al., Revisiting the Uniform Information Density Hypothesis in *Proceedings of the 2021 Conference on Empirical Methods in Natural Language Processing.* pp. 963–980 (2021).
5. JL Hoover, M Sonderegger, ST Piantadosi, TJ O'Donnell, The Plausibility of Sampling as an Algorithmic Theory of Sentence Processing. *Open Mind* **7**, 350–391 (2023).
6. SG Luke, K Christianson, Limits on lexical prediction during reading. *Cogn. Psychol.* **88**, 22–60 (2016).
7. K Rayner, A Pollatsek, D Drieghe, TJ Slattery, ED Reichle, Tracking the mind during reading via eye movements: Comments on {Kliegl, Nuthmann, and Engbert} (2006). *J. Exp. Psychol.* **136**, 520–529 (2007).
8. D Drieghe, Parafoveal-on-foveal effects on eye movements during reading in *The Oxford Handbook of Eye Movements.* (Oxford University Press), (2011).
9. B Angele, et al., Do successor effects in reading reflect lexical parafoveal processing? Evidence from corpus-based and experimental eye movement data. *J. Mem. Lang.* **79**, 76–96 (2015).
10. T Brothers, LJ Hoversten, MJ Traxler, Looking back on reading ahead: No evidence for lexical parafoveal-on-foveal effects. *J. Mem. Lang.* **96**, 9–22 (2017).
11. SN Wood, *Generalized Additive Models: An Introduction with R.* (Chapman and Hall/CRC, Boca Raton), (2006).
12. D Bates, M Mächler, B Bolker, S Walker, Fitting linear mixed-effects models using lme4. *J. Stat. Softw.* **67**, 1–48 (2015).
13. NJ Smith, R Levy, Cloze but no cigar: The complex relationship between cloze, corpus, and subjective probabilities in language processing in *Proceedings of the 33rd CogSci Conference.* (2011).
14. S Frade, A Santi, A Raposo, Filling the gap: Cloze probability and sentence constraint norms for 807 European Portuguese sentences. *Behav. Res. Methods* pp. 1–10 (2023).
15. Y Benjamini, D Yekutieli, The control of the false discovery rate in multiple testing under dependency. *Annals statistics* **29**, 1165–1188 (2001).
16. MJ Hofmann, S Remus, C Biemann, R Radach, L Kuchinke, Language models explain word reading times better than empirical predictability. *Front. Artif. Intell.* **4** (2021).
17. JA Michaelov, S Coulson, BK Bergen, So Cloze yet so Far: N400 amplitude is better predicted by distributional information than human predictability judgements. *IEEE Transactions on Cogn. Dev. Syst.* (2022).
18. A Staub, M Grant, L Astheimer, A Cohen, The influence of cloze probability and item constraint on cloze task response time. *J. Mem. Lang.* **82**, 1–17 (2015).
19. DA Balota, et al., The English lexicon project. *Behav. research methods* **39**, 445–459 (2007).
20. D Graff, J Kong, K Chen, K Maeda, English Gigaword Third Edition LDC2007T07 (2007).
21. C Shain, Word Frequency and Predictability Dissociate in Naturalistic Reading. *PsyArXiv* (2023).
22. A Gokaslan, V Cohen, OpenWebText Corpus (year?).
23. A Staub, The effect of lexical predictability on eye movements in reading: Critical review and theoretical interpretation. *Lang. Linguist. Compass* **9**, 311–327 (2015).
24. EG Wilcox, T Pimentel, C Meister, R Cotterell, RP Levy, Testing the Predictions of Surprisal Theory in 11 Languages. *arXiv e-prints* pp. arXiv–2307 (2023).
25. HH Clark, The language-as-fixed-effect fallacy: A critique of language statistics in psychological research. *J. verbal learning verbal behavior* **12**, 335–359 (1973).
26. U Hasson, CJ Honey, Future trends in Neuroimaging: Neural processes as expressed within real-life contexts. *NeuroImage* **62**, 1272–1278 (2012).

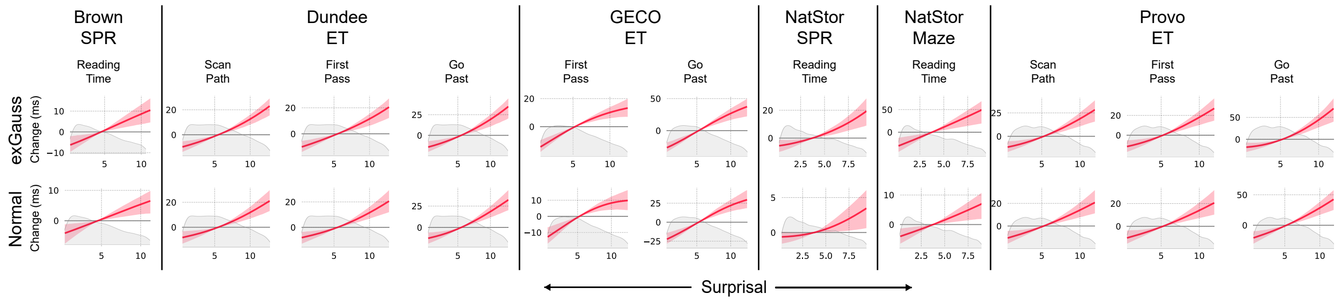

Fig. S11. CDRNN-estimated functional form of GPT-2 surprisal effects using the ExGaussian predictive distribution assumed in our main analyses (top row, reproduced from Figure 2 for convenience) vs. the more widely used Normal predictive distribution (bottom row). Plots cover the interdecile range of surprisal values in each training dataset. Estimates are highly similar across distributions.

27. DJ Barr, R Levy, C Scheepers, HJ Tily, Random effects structure for confirmatory hypothesis testing: Keep it maximal. *J. Mem. Lang.* **68**, 255–278 (2013).
28. U Hasson, G Egidi, M Marelli, RM Willems, Grounding the neurobiology of language in first principles: The necessity of non-language-centric explanations for language comprehension. *Cognition* **180**, 135–157 (2018).
29. KL Campbell, LK Tyler, Language-related domain-specific and domain-general systems in the human brain. *Curr. Opin. Behav. Sci.* **21**, 132–137 (2018).
30. LS Hamilton, AG Huth, The revolution will not be controlled: natural stimuli in speech neuroscience. *Lang. cognition neuroscience* **35**, 573–582 (2020).
31. S Jain, VA Vo, L Wehbe, AG Huth, Computational language modeling and the promise of in silico experimentation. *Neurobiol. Lang.* pp. 1–65 (2023).
32. E Diachek, I Blank, M Siegelman, E Fedorenko, The domain-general multiple demand (MD) network does not support core aspects of language comprehension: A large-scale fMRI investigation. *J. Neurosci.* **40**, 4536–4550 (2020).
33. DG Mook, In defense of external invalidity. *Am. psychologist* **38**, 379 (1983).
34. PA Carpenter, MA Just, What your eyes do while your mind is reading in *Eye movements in reading: Perceptual and language processes*, ed. K Rayner. (Academic Press), pp. 275–307 (1983).
35. M Brysbaert, F Vitu, Word skipping: Implications for theories of eye movement control in reading in *Eye guidance in reading and scene perception.* (Elsevier), pp. 125–147 (1998).
36. K Rayner, Eye Movements in Reading and Information Processing: 20 Years of Research. *Psychol. Bull.* **124**, 372–422 (1998).
37. K Rayner, TJ Slattery, D Drieghe, SP Liversedge, Eye movements and word skipping during reading: effects of word length and predictability. *J. Exp. Psychol. Hum. Percept. Perform.* **37**, 514 (2011).
38. Y Duan, K Bicknell, A rational model of word skipping in reading: ideal integration of visual and linguistic information. *Top. cognitive science* **12**, 387–401 (2020).
39. SF Ehrlich, K Rayner, Contextual effects on word perception and eye movements during reading. *J. verbal learning verbal behavior* **20**, 641–655 (1981).
40. S Frisson, K Rayner, MJ Pickering, Effects of contextual predictability and transitional probability on eye movements during reading. *J. Exp. Psychol. Learn. Mem. Cogn.* **31**, 862 (2005).
41. G Fitzsimmons, D Drieghe, How fast can predictability influence word skipping during reading? *J. Exp. Psychol. Learn. Mem. Cogn.* **39**, 1054 (2013).
42. K Rayner, GW McConkie, What guides a reader's eye movements? *Vis. research* **16**, 829–837 (1976).
43. A Veldre, ED Reichle, R Wong, S Andrews, The effect of contextual plausibility on word skipping during reading. *Cognition* **197**, 104184 (2020).
44. ER Schotter, B Angele, K Rayner, Parafoveal processing in reading. *Attention, Perception, & Psychophys.* **74**, 5–35 (2012).
45. GW McConkie, K Rayner, The span of the effective stimulus during a fixation in reading. *Percept. & Psychophys.* **17**, 578–586 (1975).
46. RK Morris, K Rayner, A Pollatsek, Eye movement guidance in reading: the role of parafoveal letter and space information. *J. Exp. Psychol. Hum. Percept. Perform.* **16**, 268 (1990).
47. HE Blanchard, A Pollatsek, K Rayner, The acquisition of parafoveal word information in reading. *Percept. & Psychophys.* **46**, 85–94 (1989).
48. A Staub, K Goddard, The role of preview validity in predictability and frequency effects on eye movements in reading. *J. Exp. Psychol. Learn. Mem. Cogn.* **45**, 110 (2019).
49. M van Schijndel, W Schuler, Addressing surprisal deficiencies in reading time models in *Proceedings of the workshop on computational linguistics for linguistic complexity (CL4LC).* pp. 32–37 (2016).
50. R Kliegl, A Nuthmann, R Engbert, Tracking the mind during reading: The influence of past, present, and future words on fixation durations. *J. experimental psychology: Gen.* **135**, 12 (2006).
51. M van Schijndel, T Linzen, Can Entropy Explain Successor Surprisal Effects in Reading? in *Proceedings of the Society for Computation in Linguistics (SCiL) 2019.* pp. 1–7 (2019).
52. T Pimentel, C Meister, EG Wilcox, R Levy, R Cotterell, On the Effect of Anticipation on Reading Times. *arXiv preprint arXiv:2211.14301* (2022).
53. JJS Barton, HM Hanif, L Eklinder Björnström, C Hills, The word-length effect in reading: A review. *Cogn. neuropsychology* **31**, 378–412 (2014).
54. M Brysbaert, et al., The word frequency effect. *Exp. psychology* (2011).
55. A Staub, Do effects of visual contrast and font difficulty on readers' eye movements interact with effects of word frequency or predictability? *J. Exp. Psychol. Hum. Percept. Perform.* **46**, 1235 (2020).
56. BD Oh, C Clark, W Schuler, Comparison of Structural Parsers and Neural Language Models as Surprisal Estimators. *Front. Artif. Intell.* **5** (2022).
57. JM Szewczyk, KD Federmeier, Context-based facilitation of semantic access follows both logarithmic and linear functions of stimulus probability. *J. Mem. Lang.* **123**, 104311 (2022).
58. I Tenney, D Das, E Pavlick, BERT rediscovers the classical NLP pipeline. *ACL19* (2019).
59. J Hewitt, CD Manning, A structural probe for finding syntax in word representations in *Proceedings of the 2019 Conference of the North American Chapter of the Association for Computational Linguistics: Human Language Technologies, Volume*

1 (Long and Short Papers). pp. 4129–4138 (2019).

60. J Brennan, EP Stabler, SE Van Wagenen, WM Luh, JT Hale, Abstract linguistic structure correlates with temporal activity during naturalistic comprehension. *Brain language* **157**, 81–94 (2016).
61. C Shain, I Blank, M van Schijndel, W Schuler, E Fedorenko, fMRI reveals language-specific predictive coding during naturalistic sentence comprehension. *Neuropsychologia* **138**, 107307 (2020).
62. T Hastie, R Tibshirani, Generalized additive models. *Stat. Sci.* **1**, 297–310 (1986).
63. RA Rigby, DM Stasinopoulos, Generalized additive models for location, scale and shape. *Appl. Stat.* **54**, 507–554 (2005).
64. DC Mitchell, An evaluation of subject-paced reading tasks and other methods for investigating immediate processes in reading. *New methods reading comprehension research* pp. 69–89 (1984).
65. C Shain, W Schuler, Continuous-Time Deconvolutional Regression for Psycholinguistic Modeling. *Cognition* **215**, 104735 (2021).
66. C Shain, W Schuler, A Deep Learning Approach to Analyzing Continuous-Time Systems. *arXiv preprint arXiv:2209.12128* (2022).
67. R Futrell, et al., The Natural Stories corpus: a reading-time corpus of English texts containing rare syntactic constructions. *Lang. Resour. Eval.* pp. 1–15 (2020).
68. K Heafield, I Pouzyrevsky, JH Clark, P Koehn, Scalable modified Kneser-Ney language model estimation in *Proceedings of the 51st Annual Meeting of the Association for Computational Linguistics*. (Sofia, Bulgaria), pp. 690–696 (2013).
69. M Breen, Empirical investigations of the role of implicit prosody in sentence processing. *Lang. Linguist. Compass* **8**, 37–50 (2014).
70. MJ Nelson, et al., Neurophysiological dynamics of phrase-structure building during sentence processing. *Proc. Natl. Acad. Sci.* **114**, E3669–E3678 (2017).
71. D Hendrycks, K Gimpel, Gaussian error linear units (GELUs). *arXiv preprint arXiv:1606.08415* (2016).
72. N Srivastava, G Hinton, A Krizhevsky, I Sutskever, R Salakhutdinov, Dropout: A simple way to prevent neural networks from overfitting. *The J. Mach. Learn. Res.* **15**, 1929–1958 (2014).
73. AM Winkler, GR Ridgway, MA Webster, SM Smith, TE Nichols, Permutation inference for the general linear model. *Neuroimage* **92**, 381–397 (2014).
74. A Staub, SJ White, D Drieghe, EC Hollway, K Rayner, Distributional effects of word frequency on eye fixation durations. *J. Exp. Psychol. Hum. Percept. Perform.* **36**, 1280 (2010).
75. A Staub, The effect of lexical predictability on distributions of eye fixation durations. *Psychon. bulletin & review* **18**, 371–376 (2011).
